# Supplementary material for: Transition between cell states of sensitivity reveals molecular vulnerability of drug-tolerant cells
Source: Mol Syst Biol. 2025 Oct 1;21(12):4. doi: 10.1038/s44320-025-00150-0 (PMC12673137; doi:10.1038/s44320-025-00150-0)
Supplement: Supplementary file 1 — Appendix [file 44320_2025_150_MOESM1_ESM.pdf]

Appendix for Transition between cell states of sensitivity reveals molecular vulnerability of drug-tolerant cells.

Ludovic Peyre, Marielle Péré, Mickael Meyer, Benjamin Bian, Marina Moureau-Barbato, Walid Djema, Bernard Mari, Georges Vassaux, Jérémie Roux

Contents

1 Mathematical modeling process **2**
- 1.1 Capturing proliferation dynamics . . . 2
- 1.2 Phenotypic switch model for one drug (PSM1D) . . . 2
- 1.3 Cytotoxics and cytostatics effects of one drug . . . 4
- 1.4 Modeling plasticity effects and how cell populations adapt to drug . . . 5
- 1.5 Drug memory and integro differential equations . . . 6
- 1.6 Drugs synergy - PSM2D . . . 6

2 Calibration **9**
- 2.1 Proliferation . . . 9
- 2.2 PSM1D fitting . . . 9
- 2.3 PSM2D fitting . . . 11

3 Appendix - Python Code **11**

4 Appendix - Simulations **13**

5 Appendix - Figures **13**

List of Tables

Appendix Table S1 Phenotypic Switch Model (PSM) configurations with parameter settings 5
Appendix Table S2 Weights used during calibration of PSM1D according to drug regimen. . 10
Appendix Table S3 Initial guess and parameters boundaries used during calibration after a first manual fit. . . . 10
Appendix Table S4 Values of fixed parameters for PSM1D . . . 11

List of Figures

Appendix Figure S1 A HeLa-RIPK3-Cherry cell line to monitor necroptosis induction in live-cell experiments. . . . 15
Appendix Figure S2 Protein markers of apoptosis and necroptosis signaling pathways activation in HeLa-RIPK3 and HT-29 cells lines. . . . 16

1 Mathematical modeling process

Our models provide a in silico platform to investigate the reversible nature of the drug-tolerant phenotype in isogenic cancer cell populations, along with the drug impact on the cell-state transitions during pro-apoptotic and pro-necroptotic sequential treatments. To capture drug regimens specificities and identify critical mechanisms of drug effects, several models' configurations are calibrated using our experimental data. Essentially, we first model naive cell proliferation in the cell culture dish (without drug). We then evaluate different model topologies for each cytotoxic drug regimen (TRAIL and TBQ). Finally, we simply couple the model with the best topology solution for each treatment regimen (the topology with the lowest Root Mean Square Error (RMSE)), to measure the ability of the coupled model to recapitulate specific behaviors observed during alternated treatment regimen (Fig. 5). Both PSM1D and PSM2D equilibrium point studies are also provided (Murray, 2007).

1.1 Capturing proliferation dynamics

Modeling cell proliferation is the first key-step to be able to measure cytostatic and cytotoxic effects of a drug (Cotner et al., 2023). Here, we use the simplest form of ordinary differential equation model to capture proliferation dynamics of the total cell population N in a dish:

$$\dot{N} = P(N) \cdot N \qquad (\textbf{Total Cell Proliferation})$$

with $P(N)$ denotes one of the following candidate functions defined below, each of them including a saturation term when 100% of the area of the dish $N_{\max}$ is occupied.

$$P_A(N) = (\beta + b.N)(1 - \frac{N}{N_{\max}}) \qquad (\textbf{Allee effect})$$

$$P_G(N) = \beta.\ln(\frac{N}{N_{\max}}) \qquad (\textbf{Gompertz})$$

$$P_H(N) = \frac{\beta}{\beta + N^n}(1 - \frac{N}{N_{\max}}) \qquad (\textbf{Hill})$$

$$P_L(N) = \beta.(1 - \frac{N}{N_{\max}}) \qquad (\textbf{Logistic})$$

Box 1 shows the ability of each of these four functions to capture nature cell dynamic proliferation in the dish during short and long-term control experiments, identifying P_H as the closest to reality similarly to (Mackey, 1978).[1]

1.2 Phenotypic switch model for one drug (PSM1D)

As a reminder, our model is similar to Nam et al's (Nam et al., 2024). The phenotypic switch model for one drug (PSM1D) is a two-state system with a drug-tolerant phenotype T and a sensitive phenotype S_m, m being the death modality triggered by the drug under study (Apoptosis (A) for TRAIL and Necroptosis (N) for TBQ), with associated switching rates α_{S_mT} and α_{TS_m}. For each compartment, proliferation is only accounted by cell renewals (Stiehl et al., 2014). Therefore the number of new sensitive cells is proportional to the number of sensitive mother cells leading to the following phenotypic switch model (PSM) initial model:

[1]Note that we chose a specific form of the Hill function to avoid very small values for β

$$
\begin{cases}
\dot{S}_m &= \underbrace{\frac{S_m}{(T+S_m)}}.P_H(\beta,n,T+S_m).(T+S_m) - \alpha_{S_mT}.S_m + \alpha_{TS_m}.T \\
\dot{T} &= \underbrace{\frac{T}{(T+S_m)}}_{\text{cell renewal}}.\underbrace{P_H(\beta,n,T+S_m).(T+S_m)}_{\text{global proliferation}} + \underbrace{\alpha_{S_mT}.S_m - \alpha_{TS_m}.T}_{\text{phenotypic switch}}
\end{cases} \quad (\textbf{PSM1D})
$$

with

$$P_H(\beta,n,T+S_m) = \frac{\beta}{\beta+(T+S_m)^n}.(1-\frac{T+S_m}{N_{\max}}) \quad (\textbf{Proliferation function})$$

Theorem 1.1. *Model **PSM1D** admits two equilibrium points:*

$$N^0 = (S_m^0, T^0) = (0,0) \quad (1)$$

$$N^* = (S_m^*, T^*) = \left(N_{\max}\frac{\alpha_{TS_m}}{\alpha_{TS_m}+\alpha_{S_mT}}, N_{\max}\frac{\alpha_{S_mT}}{\alpha_{TS_m}+\alpha_{S_mT}}\right) \quad (\textbf{PSM1D equilibria})$$

with N^* is marginally stable and N^0 is not stable. Solutions are non-negative and bounded from above by $N_{\max}$.

In dynamical systems, a marginally stable system exhibits a bounded output, but it does not necessarily decay to the equilibrium (Murray, 2007). Being marginally stable allows cells to have more flexibility and adaptability in response to outside stimuli.

Proof. The jacobian of **PSM1D** is given by:

$$Jac(PSM1D)(N) = \begin{bmatrix} \frac{\partial P_H}{\partial S_m}S_m + P_H(\beta,n,T+S_m) - \alpha_{S_mT} & \frac{\partial P_H}{\partial T}S_m + \alpha_{TS_m} \\ \frac{\partial P_H}{\partial S_m}T + \alpha_{S_mT} & \frac{\partial P_H}{\partial T}T + P_H(\beta,n,T+S_m) - \alpha_{TS_m} \end{bmatrix} \quad (2)$$

with

$$\frac{\partial P_H}{\partial T} = \frac{\partial P_H}{\partial S_m} = \frac{-n\beta(T+S_m)^{n-1}}{(\beta+(T+S_m)^n)^2}.(1-\frac{T+S_m}{N_{\max}}) - \frac{1}{N_{\max}}.\frac{\beta}{\beta+(T+S_m)^n}. \quad (3)$$

Therefore, we have:

$$Jac(PSM1D)(N^0) = \begin{bmatrix} 1-\alpha_{S_mT} & +\alpha_{TS_m} \\ +\alpha_{S_mT} & 1-\alpha_{TS_m} \end{bmatrix} \quad (4)$$

and

$$Jac(PSM1D)(N^*) = \begin{bmatrix} \frac{-\beta}{\beta+N_{\max}^n}.\frac{\alpha_{TS_m}}{\alpha_{TS_m}+\alpha_{S_mT}} - \alpha_{S_mT} & \frac{-\beta}{\beta+N_{\max}^n}\frac{\alpha_{TS_m}}{\alpha_{TS_m}+\alpha_{S_mT}} + \alpha_{TS_m} \\ \frac{-\beta}{\beta+N_{\max}^n}.\frac{\alpha_{S_mT}}{\alpha_{TS_m}+\alpha_{S_mT}} + \alpha_{S_mT} & \frac{-\beta}{\beta+N_{\max}^n}\frac{\alpha_{S_mT}}{\alpha_{TS_m}+\alpha_{S_mT}} - \alpha_{TS_m} \end{bmatrix} \quad (5)$$

To identify the eigen values associated with N^0, we solve:

$$\det(Jac(PSM1D)(N^0) - \lambda I) = (1-\alpha_{S_mT}-\lambda)(1-\alpha_{TS_m}-\lambda) - \alpha_{TS_m}\alpha_{S_mT} = 0, \quad (6)$$

which gives the following equation:

$$\lambda^2 - (2-\alpha_{S_mT}-\alpha_{TS_m})\lambda + (1-\alpha_{S_mT}-\alpha_{TS_m}+\alpha_{S_mT}\alpha_{TS_m}) = 0. \quad (7)$$

The discriminant is given by:

$$\Delta = (2-\alpha_{S_mT}-\alpha_{TS_m})^2 - 4(1-\alpha_{S_mT}-\alpha_{TS_m}+\alpha_{S_mT}\alpha_{TS_m}) = (\alpha_{S_mT}-\alpha_{TS_m})^2 \quad (8)$$

we obtain the following eigen values:

$$\lambda_1^0 = 1, \quad \lambda_2^0 = 1 - \alpha_{S_mT} - \alpha_{TS_m}. \tag{9}$$

As $\lambda_1^0 = 1$, the system is never asymptotically stable. The stability type depends on λ_2^0. If $1 - \alpha_{S_mT} - \alpha_{TS_m} < 0$, the system has a saddle point, if $1 - \alpha_{S_mT} - \alpha_{TS_m} > 0$, the system is an unstable source. For N^*, solving the characteristic equation $\det(Jac(PSM1D)(N^*) - \lambda I) = 0$, we obtain the following eigen values:

$$\lambda^*_{-,+} = \frac{\left[\frac{-\beta}{\beta + N_{\max}^n}\left(\frac{\alpha_{TS_m}}{\alpha_{TS_m}+\alpha_{S_mT}} + \frac{\alpha_{S_mT}}{\alpha_{TS_m}+\alpha_{S_mT}}\right) - (\alpha_{S_mT} + \alpha_{TS_m})\right] \pm \sqrt{\Delta^*}}{2} \tag{10}$$

where:

$$\Delta^* = \left(\frac{-\beta}{\beta + N_{\max}^n}\left(\frac{\alpha_{TS_m}}{\alpha_{TS_m}+\alpha_{S_mT}} + \frac{\alpha_{S_mT}}{\alpha_{TS_m}+\alpha_{S_mT}}\right) - (\alpha_{S_mT} + \alpha_{TS_m})\right)^2. \tag{11}$$

Therefore, $N_{\max}$ is marginally stable. Calculating the derivative values for N^0 and N^* and the regularity of our function defining **PSM1D**, it is straightforward to show that our solutions are bounded by 0 and $N_{\max}$. □

1.3 Cytotoxics and cytostatics effects of one drug

Population scale is a level at which models can help measure drug cytostatic effects. Differentiating these effects from long-term cytotoxicity is challenging though. In most cancer population models, cell death is represented either as a decrease in proliferation (Pisco et al., 2013), a continuous process accounted for by a degradation term (Howard et al., 2022; Kumar et al., 2019; Gunnarsson et al., 2020) that could be drug-enhanced (Lorenzi et al., 2016) or a death probability (Chisholm et al., 2015). Here, we take advantage of the different time scales of the effect of TRAIL. Experimental data from single-cell studies using TRAIL and TBQ show that drug-induced cell death occurs between 1 and 6 hours after drug administration, while our model operates on a timescale consistent with cell proliferation and the acquisition of experimental data points, progressing over days. We therefore model cytotoxic effects as an impulsive process for both drugs, resetting the sensitive variable S to zero right after each drug administration time point t_d, since sensitive cells can only be identified retrospectively, i.e., at the moment of death. In summary, only sensitive cells can undergo cell death, which is exclusively linked to cytotoxic effects in the models (other death mechanisms are neglected) and leading to the following equality during model simulation:

$$S(t_d) = 0 \qquad \textbf{(Cytotoxic effects)}$$

Similarly, the dynamic of the cytotoxic drug D also integrates impulsive drug inputs, whether it is TRAIL or TBQ.

For a time point t, given a set of drug input times $t_d \in \{t_1, t_2, \ldots, t_n\}$, the concentration $D(t)$ of the cytotoxic drug at time t is defined by:

$$\mathcal{D}(t) = \max\left(D_{\text{input}}(t_d) + k_d \times (t - t_{\text{last\_input}}) + D_{\text{previous}}, 0\right) \qquad \textbf{(Drug dynamics)}$$

where:

- $D_{\text{input}}(t_d)$: Initial concentration after each drug input set to experimental drug dose (20 ng/mL for TRAIL and 40 ng/mL for TBQ).
- k_d: Constant rate at which the drug is degradated over time after each input.
- $t_{\text{last\_input}}$: Most recent drug input time before t.

- D_{previous}: Cumulative drug concentration from previous input intervals.

To test whether cytotoxic drugs also have cytostatic effects, we provide two configurations of our model where the proliferation rate β is rather inhibited (-) by the drug or kept constant (=), see Eq. **Drug-induced change**.

1.4 Modeling plasticity effects and how cell populations adapt to drug

In addition to proliferation inhibition, we hypothesize that TRAIL impacts not only cell death, but also the ability of cells to switch between sensitivity states, as suggested by (Flusberg et al., 2013) and our group. Fig. 1B and 1D indicate that multiple drug administrations within a brief period significantly increase the proportion of tolerant cells for both TRAIL and TBQ, leading to sustained resistance to future drug exposure. However, incorporating a resting period between two drug treatments restores the original drug sensitivity, and the initial proportion of sensitive cells reappears, resulting in increased cytotoxicity compared to multiple successive drug inputs. This adaptive behavior may be driven by cellular plasticity (Bell and Gilan, 2020), allowing cells to evade treatment through drug-induced activation of compensatory pathways. We therefore hypothesize that prolonged exposure to TRAIL either inhibits the transition from tolerant to sensitive state and/or enhances the transition from sensitive to tolerant. To test this hypothesis, we developed eight configurations of our model:

- +/-/- (1): Drug activates α_{S_mT}, inhibits α_{TS_m} and β
- +/=/- (2): Drug increases α_{S_mT} and inhibits only β
- =/-/- (3): Drug only decreases α_{TS_m} and β
- =/=/- (4): Drug does not impact switching rates but still inhibits β
- ./.= (5,6,7,8): Same hypothesis tested for switching rates but drug does not impact the proliferation rate

summarized in Appendix Table S1:

Topology	1	2	3	4	5	6	7	8
β	-				=			
α_{S_mT}	+		=		+		=	
α_{TS}	-	=	-	=	-	=	-	=

Appendix Table S1: Phenotypic Switch Model (PSM) configurations with parameter settings. Symbols: '+' denotes the activation of the parameter, '-' the inhibition, '=' indicates no change.

Activation (ϕ^+) and inhibition functions (ϕ^-) for each parameter θ (θ could be β, α_{S_mT} or α_{TS_m}) are given by :

$$\begin{aligned} \phi^+(\theta, \mu_\theta, \Sigma_\theta) &= 100 - (100 - \theta_i) \cdot e^{-\mu_\theta \cdot \Sigma_\theta} \\ \phi^-(\theta, \mu_\theta, \Sigma_\theta) &= \frac{\theta}{1 + \mu_\theta . \Sigma_\theta} \end{aligned} \qquad \text{(\textbf{Drug-induced change})}$$

Σ_θ represents the stress induced by the drug on the parameter θ and μ_θ shows how sensitive this parameters is to the stress. When all the drug is degradated, the rates return to their original value.

1.5 Drug memory and integro differential equations

Inspired by Nam et al.(Nam et al., 2024), which represents the memory effect of cisplatin exposure on sensitive and tolerant states by taking the area under the curve (AUC) of the cisplatin concentration over time, we chose to encode a drug-memory in our model. Taking the area under the curve is a method to avoid more complicated integro-differential equations. Integro-differential equations combine differential and integral components, with a rate of change depending on both its current and past states, incorporated via an integral term. These equations are especially useful to encode memory effects as they can model how tumor growth and treatment resistance evolve over time (Chisholm et al., 2016), accounting for the cumulative effects of past treatments on current response. Other methods can be used to integrate memory effects such as delay differential equations (Barbarossa et al., 2012) or fractional derivatives (Amilo et al., 2023).

Here, to simplify the model, we chose a specific form of an integro-differential equation presented by (Beretta et al., 1990). In their study, they showed that we could replace the integral part of the equation by adding one extra degree of freedom between drug dynamic in the dish and its impact on proliferation and switching rates. This extra degree of freedom is represented with an extra variable M_θ, one for each parameter impacted. This memory variable is also present in (Denis and François, 2024) study that test different functions for this memory. μ_θ represents the drug sensitivity of the parameter and λ_θ its reset speed, the speed at which the parameter "forgets" the drug impact. Each switching and proliferation rate has its independent sensitivity rate and reset speed along with their corresponding memory. Like Nam et al., we also include a saturation term in the memory equation to account for behaviors at saturating dose represented by the AUC at the saturation dose (set to 80ng/mL for TRAIL and 20ng/mL for TBQ (see Figure Appendix 1)):

$$\dot{M}_\theta = \mathcal{D}^{\text{drug}}\left(1 - \frac{M_\theta}{AUC_{sat}^{\text{drug}}}\right) - \lambda_\theta \cdot M_\theta \qquad \textbf{(Memory equation } \mathbf{M}^\theta\textbf{)}$$

with AUC_{sat}^{drug} is the integral over time of D^{drug} with only one drug input at drug saturating dose $D_{\text{sat.}}^{\text{drug}}$ which gives these equations for the PSM with topology 1 for TRAIL:

$$\begin{cases} \dot{S}_A = & \frac{S_A}{(T+S_A)}.(\phi^-(\beta,\mu_\beta,M_\beta),n,T+S_A).(T+S_A) \\ & -\phi^+(\alpha_{S_AT},\mu_{S_AT},M_{S_AT}).S_A + \phi^-(\alpha_{TS_A},\mu_{TS_A},M_{TS_A}).T \\ \dot{T} = & \frac{T}{(T+S_A)}.P(\phi^-(\beta,\mu_\beta,M_\beta),n,T+S_A).(T+S_A) \\ & +\phi^+(\alpha_{S_AT},\mu_{S_AT},M_{S_AT}).S_A - \phi^-(\alpha_{TS_A},\mu_{TS_A},M_{TS_A}).T \\ \dot{M}_{S_AT} = & \mathcal{D}^{\text{TRAIL}}\left(1-\frac{M_{S_AT}}{AUC_{sat}^{\text{TRAIL}}}\right) - \lambda_{S_AT}.M_{S_AT} \\ \dot{MTS}_A = & \mathcal{D}^{\text{TRAIL}}\left(1-\frac{M_{TS_A}}{AUC_{sat}^{\text{TRAIL}}}\right) - \lambda_{TS_A}.M_{TS_A} \\ \dot{M}\beta = & \mathcal{D}^{\text{TRAIL}}\left(1-\frac{M_\beta}{AUC_{sat}^{\text{TRAIL}}}\right) - \lambda_\beta.M_\beta \end{cases}$$

(PSM1D for TRAIL - Topology 1)

There is no need to recalculate the equilibrium points or study their stability. Indeed, without drug addition, D tends to zero on the long-term and M_θ too, therefore the value of model's parameters are identical at the equilibrium and at the initial time by design.

1.6 Drugs synergy - PSM2D

To create our phenotypic switch model with 2 drugs (PSM2D), we split our tolerant compartment into two new ones: tolerant T to both drugs and S_N that stands for Sensitive to Necroptosis, the death modality induced by TBQ. From S_A perspective, T and S_N are seen as tolerant. We set

$\alpha_{S_A S_N} = \alpha_{S_A T}.\frac{S_N}{S_N+T}$ while $\alpha_{S_A T} = \alpha_{S_A T}.\frac{T}{S_N+T}$ and $\alpha_{S_N S_A} = \alpha_{TS_A}$, meaning that we conserve the same switching rates from and to S_A than PSM1D but the percentage of sensitive cells to apoptosis that become tolerant or sensitive to Necroptosis, is proportional to there size. $\alpha_{S_A T}$, $\alpha_{S_N T}$, α_{TS_A} and α_{TS_N} conserves their value from the calibration of PSM1D. How each drug impacts their corresponding switching rates is identical to the PSM1D for each drug along with the respective values of μ_θ and λ_θ. For instance, if we use the topology 3 for TBQ, $\alpha_{S_N T}$ will be activated by TBQ.

Fig. 5B suggests that a tolerance to TRAIL increases the sensitivity to TBQ. We model this ability by enforcing the activation of the switching rate α_{TS_N} by TRAIL (in red the following example given by **PSM2D - Topology 3 for A., 2 for N.**). The corresponding μ_{TS_N} and λ_{TS_N} are set to the value of the initial guess used for sensitive rate and reset speed during calibration of PSM1D. Finally, we made two assumptions. First, the effects of the two drugs on proliferation is simply additive whereas their effects on the switching rate from S_N to T is multiplicative/composed.

The following equations describe PSM2D where the topology 3 (=/-/-) is used for TRAIL and topology 2 for TBQ (+/=/-), topology with the smallest RMSE (Fig. S5):

$$\begin{cases} \dot{S}_A = & P_H\left(\phi^-(\beta,\mu_\beta,M_\beta),n,T+S_A+S_N\right).S_A \\ & -\alpha_{S_A T}.S_A + \phi^-(\alpha_{TS_A},\mu_{TS_A},M_{TS_A}).(S_N+T) \\ \dot{S}_N = & P_H(\phi^-(\beta,\mu_\beta,M_\beta),n,T+S_A+S_N).S_N. \\ & +\alpha_{S_A T}.S_A.\frac{S_N}{T+S_N} - \phi^-(\alpha_{TS_A},\mu_{TS_A},M_{TS_A}).S_N \\ & -\phi^+(\alpha_{S_N T},\mu_{S_N T},M_{S_N T}).S_N + {\color{red}\phi^+(\alpha_{TS_N},\mu_{TS_N},M_{TS_N})}.T \\ \dot{T} = & P_H(\phi^-(\beta,\mu_\beta,M_\beta),n,T+S_A+S_N).T \\ & +\alpha_{S_A T}.S_A.\frac{T}{T+S_N} - \phi^-(\alpha_{TS_A},\mu_{TS_A},M_{TS_A}).T \\ & +\phi^+(\alpha_{S_N T},\mu_{S_N T},M_{S_N T}).S_N - {\color{red}\phi^+(\alpha_{TS_N},\mu_{TS_N},M_{TS_N})}.T \\ \dot{M}S_N T = & \mathcal{D}^{\text{TBQ}}\left(1-\frac{M_{S_N T}}{AUC_{sat}^{\text{TBQ}}}\right) - \lambda_{S_N T}.M_{S_N T} \\ \dot{M}TS_A = & \mathcal{D}^{\text{TRAIL}}\left(1-\frac{M_{TS_A}}{AUC_{sat}^{\text{TRAIL}}}\right) - \lambda_{TS_A}.M_{TS_A} \\ \dot{M}TS_N = & \mathcal{D}^{\text{TRAIL}}\left(1-\frac{M_{TS_N}}{AUC_{sat}^{\text{TRAIL}}}\right) - \lambda_{TS_N}.M_{TS_N} \\ \dot{M}\beta = & (\mathcal{D}^{\text{TRAIL}}+\mathcal{D}^{\text{TBQ}})\left(1-\frac{M_\beta}{AUC_{sat}^{\text{TRAIL}+\text{TBQ}}}\right) - \lambda_\beta.M_\beta \end{cases}$$

(**PSM2D - Topology 3 for A., 2 for N.**)

Similarly to model **PSM1D**, in the absence of drugs, the model PSM2D admits two equilibrium points $N_2^0 = (S_A^0, S_N^0, T^0) = 0_{\mathbb{R}^3}$ and $N_2^* = (S_A^*, S_N^*, T^*)$ with

$$S_A^* = \frac{\alpha_{TS_A}}{\alpha_{TS_A}+\alpha_{S_A T}}.N_{\max} \quad (12)$$

$$S_N^* = \frac{\alpha_{S_A T}.\alpha_{TS_N}}{(\alpha_{TS_N}+\alpha_{S_N T}).(\alpha_{TS_A}+\alpha_{S_A T})}.N_{\max} \quad (13)$$

$$T^* = \frac{\alpha_{S_A T}.\alpha_{S_N T}}{(\alpha_{TS_N}+\alpha_{S_N T}).(\alpha_{TS_A}+\alpha_{S_A T})}.N_{\max}. \quad (14)$$

(15)

Let us rewrite the system to study its equilibria by posing:

$$N = S_A + S_N + T \quad (16)$$

$$V = S_N + T \quad (17)$$

which leads to the following equivalent system[2]:

$$\begin{cases} \dot{N} = & P_H\left(\beta, n, N\right).N \\ \dot{V} = & P_H\left(\beta, n, N\right).V + \alpha_{S_AT}.S_A - \alpha_{TS_A}V \\ \dot{S}_A = & P_H\left(\beta, n, N\right).S_A - \alpha_{S_AT}.S_A + \alpha_{TS_A}V \end{cases} \qquad \textbf{(PSM2D}^* \textbf{ (without drugs))}$$

Note: Let us remark here that this change of variable shows that α_{S_NT} and α_{TS_N} are not identifiable in PSM2D and have a weak impact on N and S_A, explaining the heatmap results obtained in Fig. EV6B.

PSM2D - Topology 3 for A., 2 for N. admits once two equilibria N^0 and N^* and the associated jacobian is given by:

$$Jac(PSM2D^*) = \begin{bmatrix} P_H(N) + \frac{\partial P_H}{\partial N}.N & 0 & 0 \\ \frac{\partial P_H}{\partial N}V & P_H(N) - \alpha_{TS_A} & \alpha_{S_AT} \\ \frac{\partial P_H}{\partial N}S_A & \alpha_{TS_A} & P_H(N) - \alpha_{S_AT} \end{bmatrix} \tag{18}$$

For N_0, the jacobian becomes:

$$Jac(PSM2D^*)(N^0) = \begin{bmatrix} 1 & 0 & 0 \\ 0 & 1 - \alpha_{TS_A} & \alpha_{S_AT} \\ 0 & \alpha_{TS_A} & 1 - \alpha_{S_AT} \end{bmatrix} \tag{19}$$

Solving the characteristic equation :

$$\det(Jac(PSM2D^*)(N^0) - \lambda I) = 0, \tag{20}$$

we obtain the following equation

$$1 - (\alpha_{TS_A} + \alpha_{S_AT}) - 2\lambda + \alpha_{TS_A}\alpha_{S_AT} + \lambda(\alpha_{TS_A} + \alpha_{S_AT}) + \lambda^2 - \alpha_{TS_A}\alpha_{S_AT} = 0 \tag{21}$$

and after factorization:

$$\lambda^2 - (\alpha_{TS_A} + \alpha_{S_AT})\lambda + (1 - \alpha_{TS_A})(1 - \alpha_{S_AT}) = 0. \tag{22}$$

Thus, the eigenvalues of are:

$$\lambda_1^0 = 1, \quad \lambda_{2,3}^0 = \frac{(\alpha_{TS_A} + \alpha_{S_AT}) \pm \sqrt{(\alpha_{TS_A} + \alpha_{S_AT})^2 - 4(1 - \alpha_{TS_A})(1 - \alpha_{S_AT})}}{2} \tag{23}$$

Therefore N^0 is unstable.

For the second equilibrium N^*, we notice that for $N \in (0, N_{\max}), dN/dt > 0$, therefore we have :

$$\lim_{t \to +\infty} N = N_{\max} \implies \lim_{t \to +\infty} P_H(N) \to 0 \tag{24}$$

and

$$\begin{cases} \dot{V} & = \alpha_{S_AT}S_A - \alpha_{TS_A}V \\ \dot{S}_A & = -\alpha_{S_AT}S_A + \alpha_{TS_A}V. \end{cases} \tag{25}$$

Summing the two equalities, we obtain:

$$\dot{V} + \dot{S}_A = 0 \implies V + S_A = V(t_0) + S_A(t_0). \tag{26}$$

Therefore, S_A satisfies:

$$\dot{S}_A = -\alpha_{S_AT}S_A + \alpha_{TS_A}(V(t_0) + S_A(t_0) - S_A). \tag{27}$$

The unique eigen value associated to S_A equals $-(\alpha_{S_AT} + \alpha_{TS_A})$ which is negative, demonstrating that S_A converges toward this equilibrium.

From the study of the system's equilibria, it is clear that their is no sign of bistability on the system.

[2]Note that with this form, it is clear that we could have not calibrated α_{TS_N} and α_{S_NT} using directly PSM2D.

2 Calibration

All calibrations were performed using the *minimize* function from *scipy* library with the Nelder-Mead algorithm. Boundaries values and initial guess were defined from a first manual fit. Simulations were performed over 7 days (or specified otherwise), first drug input happening 1h after simulation started. At each drug input, the S_m population is set to 0. Therefore during model calibration, we compare the total cell density total at time t_d with the measurement taken 24h after real drug input.

2.1 Proliferation

Proliferation parameters β (and n if necessary) were only calibrated on control experiments, never on experimental data with drugs. We hypothesized that cells adapt their proliferation rate but also their proliferating dynamics according to their original seeding density. To do so, we calibrated two times each of our four models for cell proliferation: a first time where the model's parameters were identical for all initial cell densities after seeding (No Parameter adaptation), and a second time where the parameters were estimated for each initial seeding condition (Parameter adaptation - Bx 1).

2.1.1 Parameter adaptation to initial cell density

To estimate each parameters of the four proliferation functions for each initial cell density, we used a weighted mean square error:

$$\mathbb{C}_{P,E} = \sum_{t_m \in \mathbb{T}_E} \omega_{.,E,m} \left(C(t_m) - N(t_m)\right)^2 \tag{28}$$

Each proliferation function P and each control experiment E had its own set of weights $\omega_{.,E,m}$, $\mathbb{T}_E$ being the set of measurement times, one every 24 hours with more measurements for long-term experiments with initial cell density much smaller than short-term experiments. All weights used could be found in Appendix Code (see function *define_params_guess_for_proliferation_calibration_unshared_parameters* in results/proliferation_study.py).

2.1.2 No parameter adaptation to initial cell density

When parameters were identical for all initial cell seeding densities, we simply took the weighted sum of the previous cost $\mathbb{C}_{P,E}$ divided by the number of measurements taken for each experiment $\dim(\mathbb{T}_E)$.

$$\mathbb{C}_P = \sum_E \frac{W_E}{\dim(\mathbb{T}_E)} \left(\sum_{t_m \in \mathbb{T}_E} \omega_{.,E,m} \left(C(t_m) - N(t_m)\right)^2 \right) \tag{29}$$

with $W_E = 1e5$ for short-term experiments that are control experiments to be compared with corresponding treated cells and therefore more important for the modeling process. $W_E \in [1e-8, 1e-7, 1e1, 1e1, 1e-7, 1e-7]$ for long-term experiments which were only designed and performed to capture naive cell proliferation dynamics and select the best proliferation function.

2.2 PSM1D fitting

To calibrate PSM1D, we used a penalized and weighted mean square error cost $\mathbb{C}_{\text{PSM1D}}$ comparing model's simulation with data from Fig. 1B-D given by:

$$\mathbb{C}_{\text{PSM1D}} = \sum_{r \in \text{drug regimen}} \left(\sum_{t_m \in [0,24,48,72]} \omega_{r,m} \left(C(t_m) - N(t_m)\right)^2 \right) + \underbrace{10 \times \left(\left(\frac{\alpha_{S_m T}}{\alpha_{S_m T} + \alpha_{T S_m}} - T_0^{prop}\right)^2 + \left(\frac{\alpha_{T S_m}}{\alpha_{S_m T} + \alpha_{T S_m}} - S_{m,0}^{prop}\right)^2 \right)}_{\text{penalization}} \tag{30}$$

(**Fitting cost PSM1D**)

with T_0^{prop} the proportion of tolerant cells in the population right after first drug input and $\omega_{r,m}$ the weight corresponding to the drug regimen r and measurement time t_m. $C(t_m)$ is the cell density measured at time t_m and $N(t_m)$ the total cell population at time t_m in the model. Respecting a period of 72h retrieves the original proportion of sensitive cells in the population, we therefore assumed that the natural equilibrium of the population responding to a specific dose of TRAIL was given by the proportion of sensitive cells (and tolerant) after first drug input. As the equilibrium point of the system that we were interested in is given by $N^{100} = (S^{100}, T^{100}) = \left(100 \frac{\alpha_{TS}}{\alpha_{TS} + \alpha_{ST}}, 100 \frac{\alpha_{ST}}{\alpha_{TS} + \alpha_{ST}}\right)$, we used this information to penalyze our cost and infer better switching rates.

We used different weights $\omega_{r,m}$ for each data point defined in the following Appendix Table S2 :

Drug	Regimen	Measurement time after first drug input		
		24h	48h	72h
TRAIL	Repeated	1000	10	100
	w Resting	100	10	100
	Sustained	1000	10	1000
TBQ	Repeated	1000	100	100
	w Resting	100	1000	100
	Sustained	1000	10	100

Appendix Table S2: Weights used during calibration of PSM1D according to drug regimen.

We only estimated switching and sensitivity rates along with reset speed and drug degradation rate. Appendix Table S3 gives the boundaries and initial guess used for each estimated parameters while Appendix Table S4 defines the values of the model fixed parameters such as the initial conditions and drug doses.

Name	Parameter	Initial guess	Lower bound	Upper bound	Death Modality
Drug degradation rate	k_d	-1	-10	-0.01	Apoptotic
		-0.9	-10	-0.1	Necroptotic
Switching rate from T to S	α_{TS}	100 x S_0	1e-3	100-1e-3	Apop./Necroptotic
Switching rate from S to T	α_{ST}	100 x T_0	1e-3	100-1e-3	Apop./Necroptotic
Reset speed of β, α_{TS} and α_{ST}	λ_θ	1e-2	1e-6	0.5	Apop./Necroptotic
Sensitivity rate of β	μ_β	1e-2	1e-3	10	Apop./Necroptotic
Sensitivity rate of α_{TS}	μ_{ts}	1e-2	1e-3	10	Apop./Necroptotic
Sensitivity rate of α_{ST}	μ_{ts}	1e-2	1e-6	10	Apop./Necroptotic

Appendix Table S3: Initial guess and parameters boundaries used during calibration after a first manual fit.

Name	Parameter	Value	Death Modality
Drug concentration (ng/mL)	D_{input}	20	Apoptotic
		40	Necroptotic
Initial condition for S (% of area in the dish)	S_0	49.116	Apoptotic
		18.276	Necroptotic
Initial condition for T (% of area in the dish)	T_0	2.485	Apoptotic
		33.325	Necroptotic
Initial Cell Seeding (% of area in the dish)	N_0	51.601	Apop./Necroptotic
TRAIL saturating dose (ng/mL)	$D_{\text{sat.}}^{\text{TRAIL}}$	80	Apoptotic
TBQ saturating dose (ng/mL)	$D_{\text{sat.}}^{\text{TBQ}}$	20	Necroptotic

Appendix Table S4: Values of fixed parameters for PSM1D with S_0 = Original Cell Density - Cell density after 1st drug input. $D_{\text{sat.}}^{\text{TBQ}}$ from Appendix Figure S1.

2.3 PSM2D fitting

In PSM2D simulations, the initial proportion of S_A in PSM2D was identical to the initial proportion of S_A in PSM1D because the same dose of TRAIL was used while the initial cell density before drug input was different (32.842 for PSM2D and 51.601 for PSM1D). The initial proportion of S_N was equal to the difference between Cell density after first input of TRAIL and Cell density after first input of TBQ during alternating regimen divided by the original cell density before any treatment. Proliferation parameters were estimated for control experiments with the same initial cell seeding while μ_β and λ_β are set to their value for PSM1D with TRAIL.

We performed a manual fit to estimate μ_{TS_N} and λ_{TS_N}. A major difference in the order of magnitude of λ_{TS_N} the rate of reset of α_{TS_N} activated by TRAIL and the rate of reset corresponding to other switching and proliferation rates must be noted, with λ_{TS_N} a hundred times larger than the other reset rates. This major difference has one important consequence: there is no memory of TRAIL for the necroptotic switching rate. TRAIL directly impacts α_{TS_N}, which mimics the drug dynamics and exhibits the reverse of the equilibrium of the necroptotic system with α_{TS_N} becoming much larger than $\alpha_{S_N T}$.

3 Appendix - Python Code

This zip file contains two folders "phenotypic_switch_model" and "Data".

Data is a folder with two subdirectories:

- **Experimental Data** that have all the .xlsx files containing experimental data to generate Fig.1:
  - one_drug_cell_density_measurements.xlsx (Fig. 1B,D)
  - one_drug_cell_density_evolution.xlsx (Fig. 1C,E)
  - two_drugs_cell_density_measurements.xlsx (Fig. 5B)
  - two_drugs_cell_density_evolution.xlsx (Fig. 5C)

  but also data from control experiments to measure naive proliferation:
  - proliferation_data (Box 1).

  Parameters for model simulations (drug input times, death times and measurements times) are available in:

    - one_drug_cell_density_measurements.xlsx
    - two_drugs_cell_density_evolution.xlsx

  In addition, two_drugs_cell_density_evolution_LT.xlsx contains the simulation parameters for long-term simulations (Fig. S5H).

- **Calibration Parameters** contains the parameters of the proliferation models for the Hill model for each initial cell seeding (beta_0_parameters_for_Hill_proliferation.xlsx) and a folder PSM1D_Article_parameters in which you can find .xlsx containing all parameters (estimated and fixed) to simulate the PSM1D model parameter, one file for each topology and death modality.

phenotypic_switch_model contains the Python code associated to this article. It consists in three major components and a main called main_phenotypic_switch.py:

- **Data Utilities** that creates all the necessary subfolders (**saving_data.py**), load experimental data and model's parameters (**loading_data.py**), and visualize them by creating Fig. 1 and 5 of the article (**data_visualization.py**).
- **Results** contains three different .py, one for each results presenting in the paper:
    - **proliferation_study** to identify the best cell proliferation function from control data and create part of the subplots of Box 1.
    - **topology_comparison_one_drug_phenotypic_switch** that simulates the 8 different model's topologies using data from Fig. 1B (for TRAIL) and 1D (TBQ), graphs the solutions, saves the corresponding figures in Supplementary_Information and creates Fig. 2 and Fig. S2 to compare the quality of fit but also growth rate dynamics between the 8 topologies for each drug.
    - **two_drugs_phenotypic_switch** combines each PSM1D topology for TRAIL with each PSM1D topology for TBQ, generating 64 new topologies for PSM2D to simulate in silico an alternated drug regimen between TRAIL and TBQ. The solutions of the model topology with the smallest RMSE in comparison with data from Fig. 5 are then used to generate Fig. 5F-G. We also provide two additional boards, exact replicate of Fig. 5F-G but also providing models solutions in the absence of drug treatment. The second board provides data a replicate of the experiment presented in Fig. 5 A-B, also alternating TRAIL and TBQ.
- **Simulations** folder gathers all functions used to run our model's simulation and compare it with experimental data:
    - **Models** is the set of model's equations along with the different proliferations & activation/inhibition functions and the drug dynamic.
    - **Plotting** regroups all the graphing functions used.
    - **Solvers** is the function used to simulate our model with the cytotoxic effects of drug input.

Each function is commented and has a corresponding dosctring.

To run the code, simply :

```
pip install -r requirements.txt
python3 Code/main_phenotypic_switch.py

```

with Python 3.10. This code creates a folder "Figures" in the same directory than Data and Code containing a subfolder for each Figure in the article using experimental data or representing simulation results. It also generates two Appendix subfolders in **Figures** with all the simulations boards for each PSM1D (Supplementary_Information_PSM2D_Simulations) and PSM2D (Supplementary_Information_PSM2D_Simulations) topologies and death modalities. It also saves the simulated solutions in Data_Solutions_Output.

4 Appendix - Simulations

The simulations results are available online as Source Data for each model topology (one PDF file per topology). For both PSM1D and PSM2D models, solutions obtained with the estimated parameters plotted for each drug regimen were graphed. Each PDF file is named according to the drug modality (A or N) and the topology used. For instance, PSM1_A_1.pdf is the simulation plots for the PSM1D using topology 1 and TRAIL, while PSM1_N_5.pdf is the simulation board for the topology 5 using TBQ. For PSM2D, PSM2_A_1_N_3.pdf is the simulation board for PSM2D combining the topology 1 for TRAIL and the topology 3 for TBQ.

In each Simulations Results PDF file, a column represents a drug regimen while each row is a variable or parameter evolution over time in the model:

- First row is the comparison between data points and extracted points in black from the total population N variable of the model
- Second row gives T, S_m and N dynamics.
- Third row is the drug dynamic D in ng/mL.
- Fourth row represents the switching rate evolution when drug impacts them.
- Fifth row represents the proliferation rate β evolution when drug inhibits it.
- Sixth row is the memory variables solutions.
- Seventh row is the contribution of proliferation in net growth rate.
- Eighth row is the contribution of phenotypic switch in net growth rate.

5 Appendix - Figures

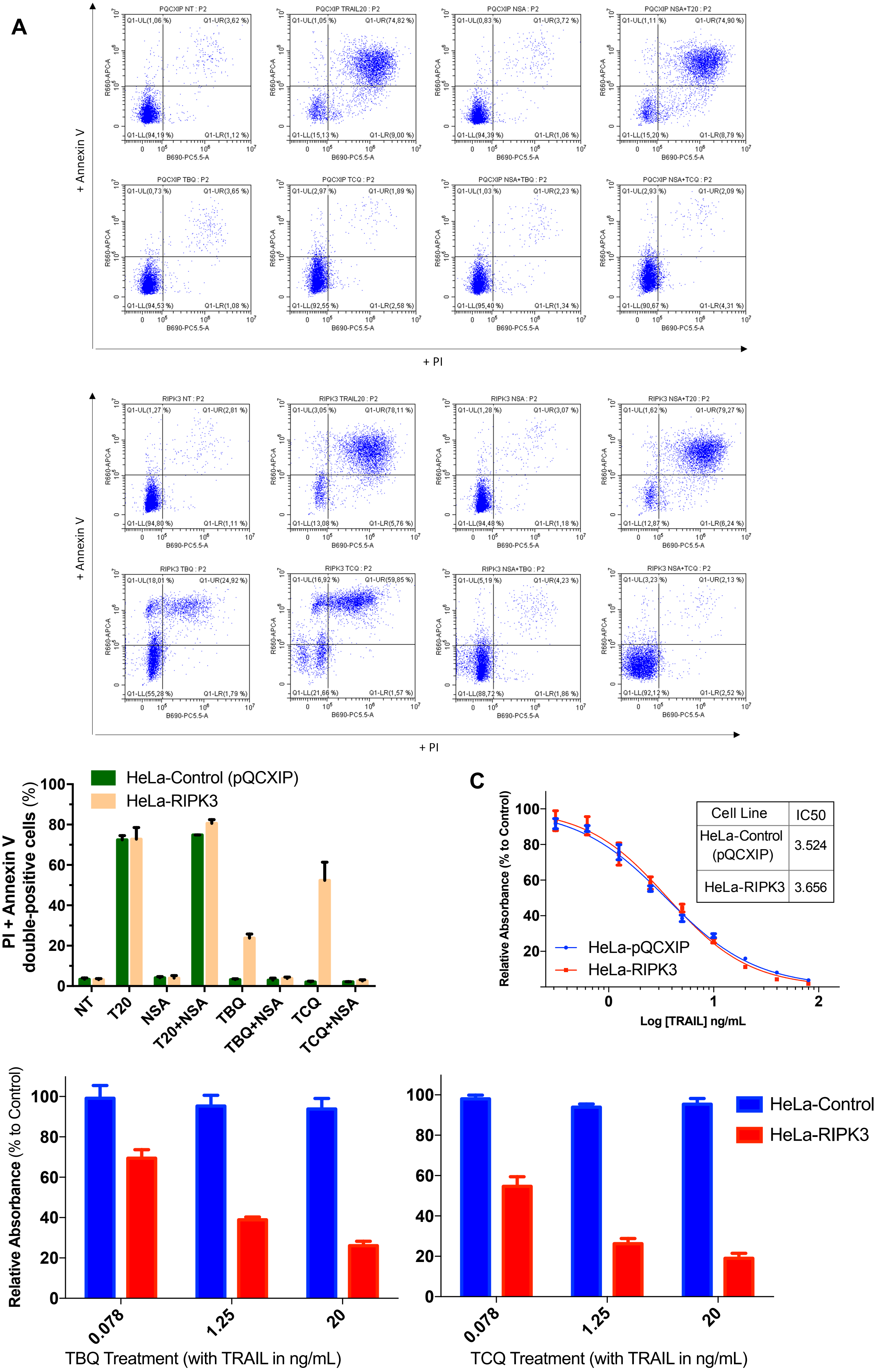
A
PQCXIP NT : P2
PQCXIP TRAIL20 : P2
PQCXIP NSA : P2
PQCXIP NSA+T20 : P2
PQCXIP TBQ : P2
PQCXIP TCQ : P2
PQCXIP NSA+TBQ : P2
PQCXIP NSA+TCQ : P2
RIPK3 NT : P2
RIPK3 TRAIL20 : P2
RIPK3 NSA : P2
RIPK3 NSA+T20 : P2
RIPK3 TBQ : P2
RIPK3 TCQ : P2
RIPK3 NSA+TBQ : P2
RIPK3 NSA+TCQ : P2
R660-APCA
B690-PC5.5-A
+ Annexin V
+ PI
B
HeLa-Control (pQCXIP)
HeLa-RIPK3
PI + Annexin V double-positive cells (%)
NT
T20
NSA
T20+NSA
TBQ
TBQ+NSA
TCQ
TCQ+NSA
C
Cell Line
IC50
HeLa-Control (pQCXIP)
3.524
HeLa-RIPK3
3.656
HeLa-pQCXIP
HeLa-RIPK3
Relative Absorbance (% to Control)
Log [TRAIL] ng/mL
D
Relative Absorbance (% to Control)
HeLa-Control
HeLa-RIPK3
0.078
1.25
20
TBQ Treatment (with TRAIL in ng/mL)
TCQ Treatment (with TRAIL in ng/mL)

Appendix Figure S1: A HeLa-RIPK3-Cherry cell line to monitor necroptosis induction in live-cell experiments. **(A)** Flow cytometry, TRAIL treatment (20ng/mL) and TBQ/TCQ (with 10ng/ml TRAIL) with or without MLKL inhibitor NSA (1µM) for 24h in HeLa-control (pQCXIP) and HeLa-RIPK3 cells. **(B)** Quantification of PI and annexin V double positive population in HeLa-Control (pQCXIP) and HeLa-RIPK3 cells. Graph bars represent the mean of three independent experiments ±SD. **(C)** Cell viability at 24h of HeLa-Control (pQCXIP) versus HeLa-RIPK3 cells with Cell-Titer-Glo assay after a TRAIL dose response. Data points represent the mean of three independent experiments ±SD. **(D)** TBQ and TCQ pro-necroptotic treatments using three concentrations of TRAIL. Effect of this dose response on HeLa-control (pQCXIP) versus HeLa-RIPK3 using Cell-Titer-Glo assay. Bar graphs show the mean of two independent experiments ±SD.

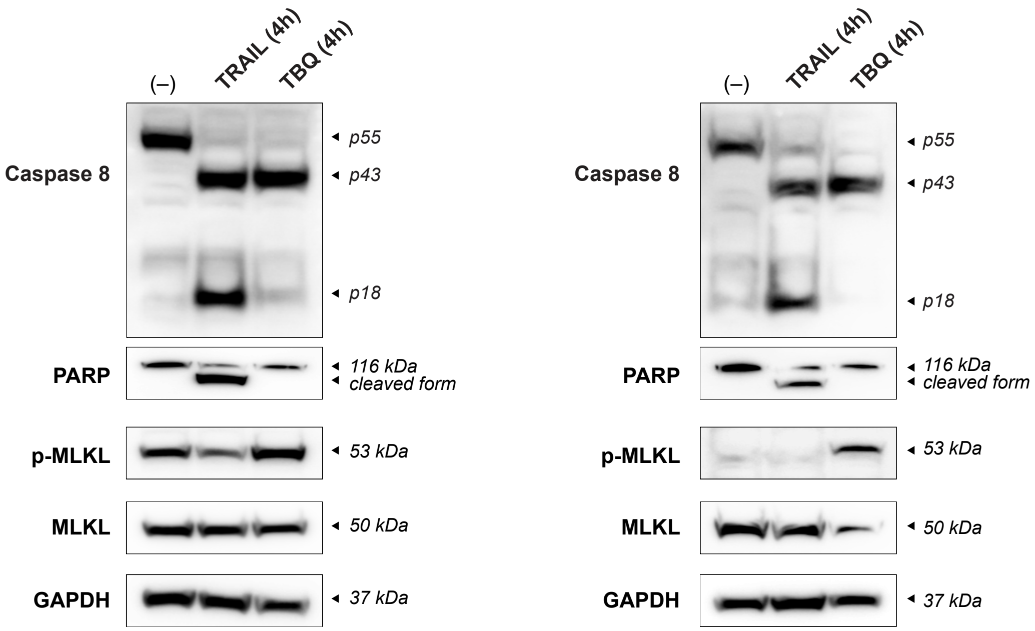

Appendix Figure S2: Protein markers of apoptosis and necroptosis signaling pathways activation in HeLa-RIPK3 and HT-29 cells lines. Protein expressions of Caspase 8, PARP, cleaved PARP, phospho-MLKL, MLKL and GAPDH in control condition (-), and after TRAIL treatment (25ng/mL, 4h), and after TBQ treatment (with TRAIL 10ng/ml, 4h) measured by Western blot analyses in HeLa-RIPK3 **(A)** and in HT-29 cells **(B)**. Two representative Western blot experiments are shown.

References

Amilo, D., Kaymakamzade, B., and Hincal, E. (2023). A fractional-order mathematical model for lung cancer incorporating integrated therapeutic approaches. *Scientific Reports*, 13(1):12426. Publisher: Nature Publishing Group.

Barbarossa, M. V., Kuttler, C., Zinsl, J., Barbarossa, M. V., Kuttler, C., and Zinsl, J. (2012). Delay equations modeling the effects of phase-specific drugs and immunotherapy on proliferating tumor cells. *Mathematical Biosciences and Engineering*, 9(2):241–257. Cc_license_type: cc_by Number: 1551-0018_2012_2_241 Primary_atype: Mathematical Biosciences and Engineering.

Bell, C. C. and Gilan, O. (2020). Principles and mechanisms of non-genetic resistance in cancer. *British Journal of Cancer*, 122(4):465–472. Publisher: Nature Publishing Group.

Beretta, E., Bischi, G. I., and Solimano, F. (1990). Stability in chemostat equations with delayed nutrient recycling. *Journal of Mathematical Biology*, 28(1):99–111.

Chisholm, R. H., Lorenzi, T., Desvillettes, L., and Hughes, B. D. (2016). Evolutionary dynamics of phenotype-structured populations: from individual-level mechanisms to population-level consequences. *Zeitschrift für angewandte Mathematik und Physik*, 67(4):100.

Chisholm, R. H., Lorenzi, T., Lorz, A., Larsen, A. K., Almeida, L. N. d., Escargueil, A., and Clairambault, J. (2015). Emergence of Drug Tolerance in Cancer Cell Populations: An Evolutionary Outcome of Selection, Nongenetic Instability, and Stress-Induced Adaptation. *Cancer Research*, 75(6):930–939.

Cotner, M., Meng, S., Jost, T., Gardner, A., De Santiago, C., and Brock, A. (2023). Integration of quantitative methods and mathematical approaches for the modeling of cancer cell proliferation dynamics. *American Journal of Physiology-Cell Physiology*, 324(2):C247–C262.

Denis, C. M. and François, P. (2024). Unclocklike oscillators with frequency memory for the entrainment of biological clocks. arXiv:2405.05180.

Flusberg, D. A., Roux, J., Spencer, S. L., and Sorger, P. K. (2013). Cells surviving fractional killing by TRAIL exhibit transient but sustainable resistance and inflammatory phenotypes. *Molecular Biology of the Cell*, 24(14):2186–2200.

Gunnarsson, E. B., De, S., Leder, K., and Foo, J. (2020). Understanding the role of phenotypic switching in cancer drug resistance. *Journal of Theoretical Biology*, 490:110162.

Howard, G. R., Jost, T. A., Yankeelov, T. E., and Brock, A. (2022). Quantification of long-term doxorubicin response dynamics in breast cancer cell lines to direct treatment schedules. *PLOS Computational Biology*, 18(3):e1009104.

Kumar, N., Cramer, G. M., Dahaj, S. A. Z., Sundaram, B., Celli, J. P., and Kulkarni, R. V. (2019). Stochastic modeling of phenotypic switching and chemoresistance in cancer cell populations. *Scientific Reports*, 9(1):10845. Publisher: Nature Publishing Group.

Lorenzi, T., Chisholm, R. H., and Clairambault, J. (2016). Tracking the evolution of cancer cell populations through the mathematical lens of phenotype-structured equations. *Biology Direct*, 11(1):43.

Mackey, M. C. (1978). Unified hypothesis for the origin of aplastic anemia and periodic hematopoiesis. *Blood*, 51(5):941–956.

Murray, J. D. (2007). *Mathematical biology: I. An introduction*, volume 17. Springer Science & Business Media.

Nam, A., Mohanty, A., Bhattacharya, S., Kotnala, S., Achuthan, S., Hari, K., Srivastava, S., Guo, L., Nathan, A., Chatterjee, R., and al. (2024). Dynamic phenotypic switching and group behavior help non-small cell lung cancer cells evade chemotherapy.

Pisco, A. O., Brock, A., Zhou, J., Moor, A., Mojtahedi, M., Jackson, D., and Huang, S. (2013). Non-Darwinian dynamics in therapy-induced cancer drug resistance. *Nature Communications*, 4(1):2467. Publisher: Nature Publishing Group.

Stiehl, T., Baran, N., Ho, A. D., and Marciniak-Czochra, A. (2014). Clonal selection and therapy resistance in acute leukaemias: mathematical modelling explains different proliferation patterns at diagnosis and relapse. *Journal of The Royal Society Interface*, 11(94):20140079. Publisher: Royal Society.
