# Supplementary figures and images for: Transition between cell states of sensitivity reveals molecular vulnerability of drug-tolerant cells
Source: Mol Syst Biol. 2025 Oct 1;21(12):4. doi: 10.1038/s44320-025-00150-0 (PMC12673137; doi:10.1038/s44320-025-00150-0)

## Slide 1
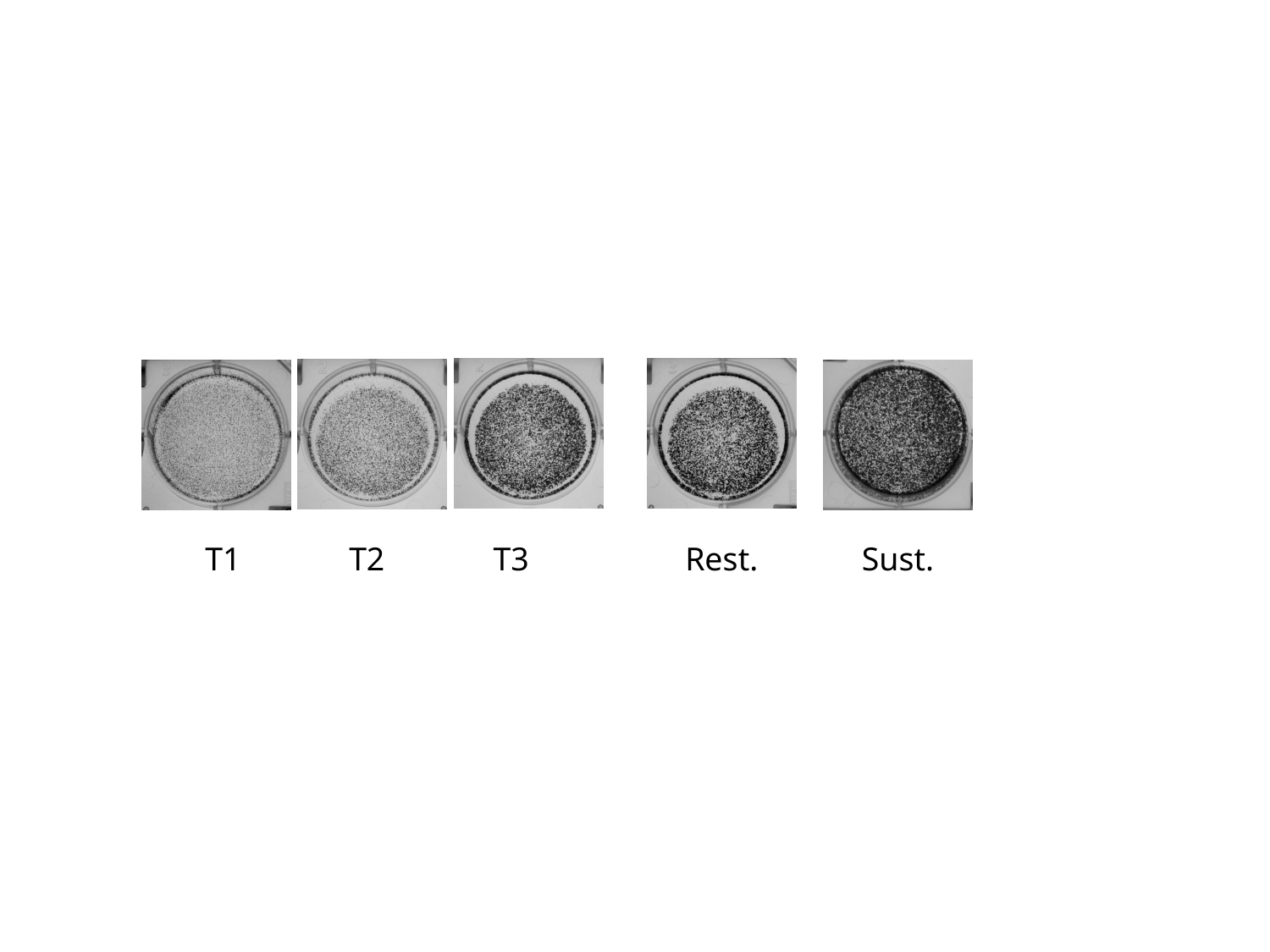

T1
T2
T3
Rest.
Sust.

Supplement: Supplementary file 3 — Source data Fig. 1 [file 44320_2025_150_MOESM3_ESM.zip › SD_Figure_1/Fig_1D_cell_density_images.pptx]

## Slide 1
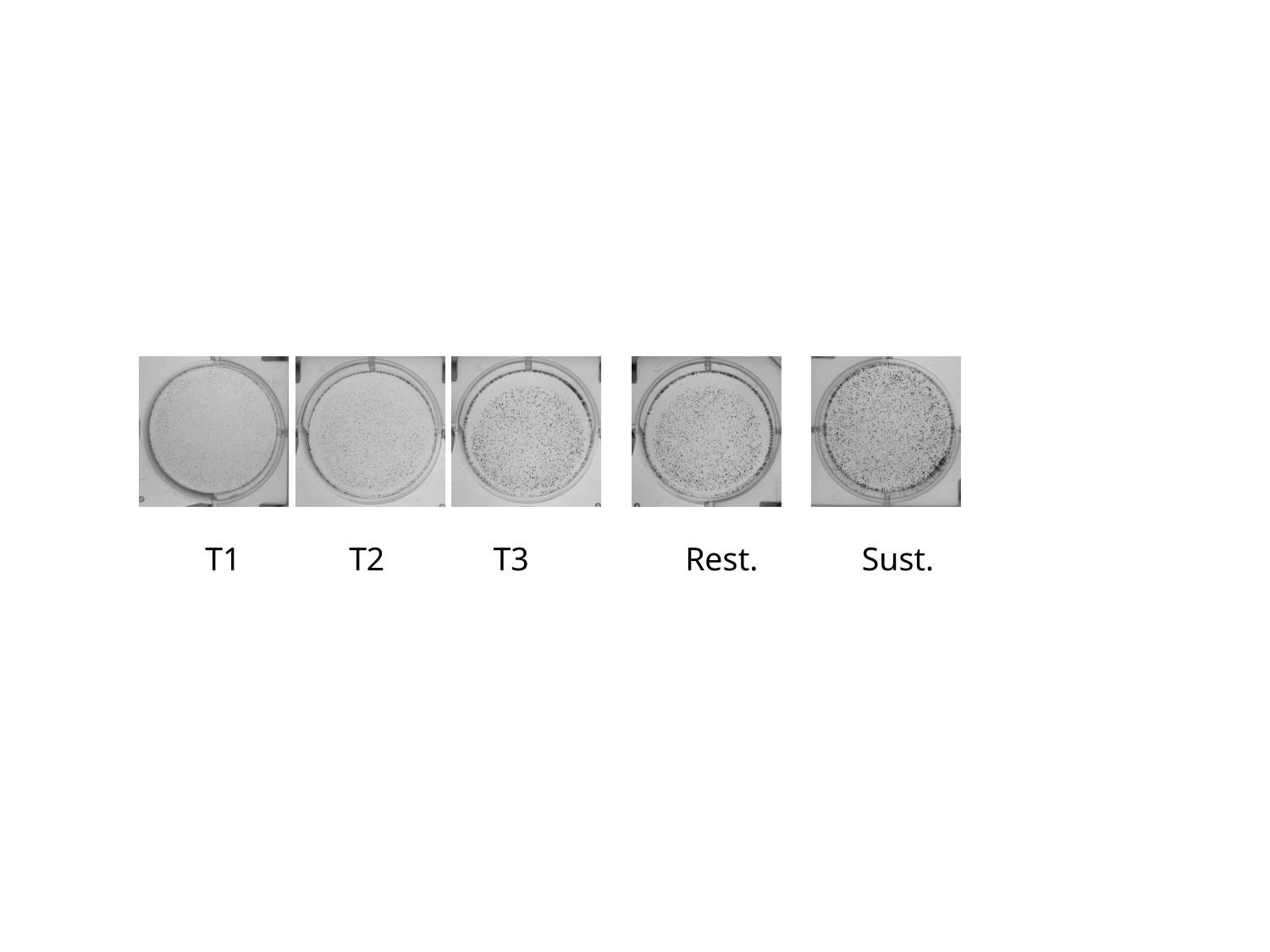

T1
T2
T3
Rest.
Sust.

Supplement: Supplementary file 3 — Source data Fig. 1 [file 44320_2025_150_MOESM3_ESM.zip › SD_Figure_1/Fig_1B_cell_density_images.pptx]

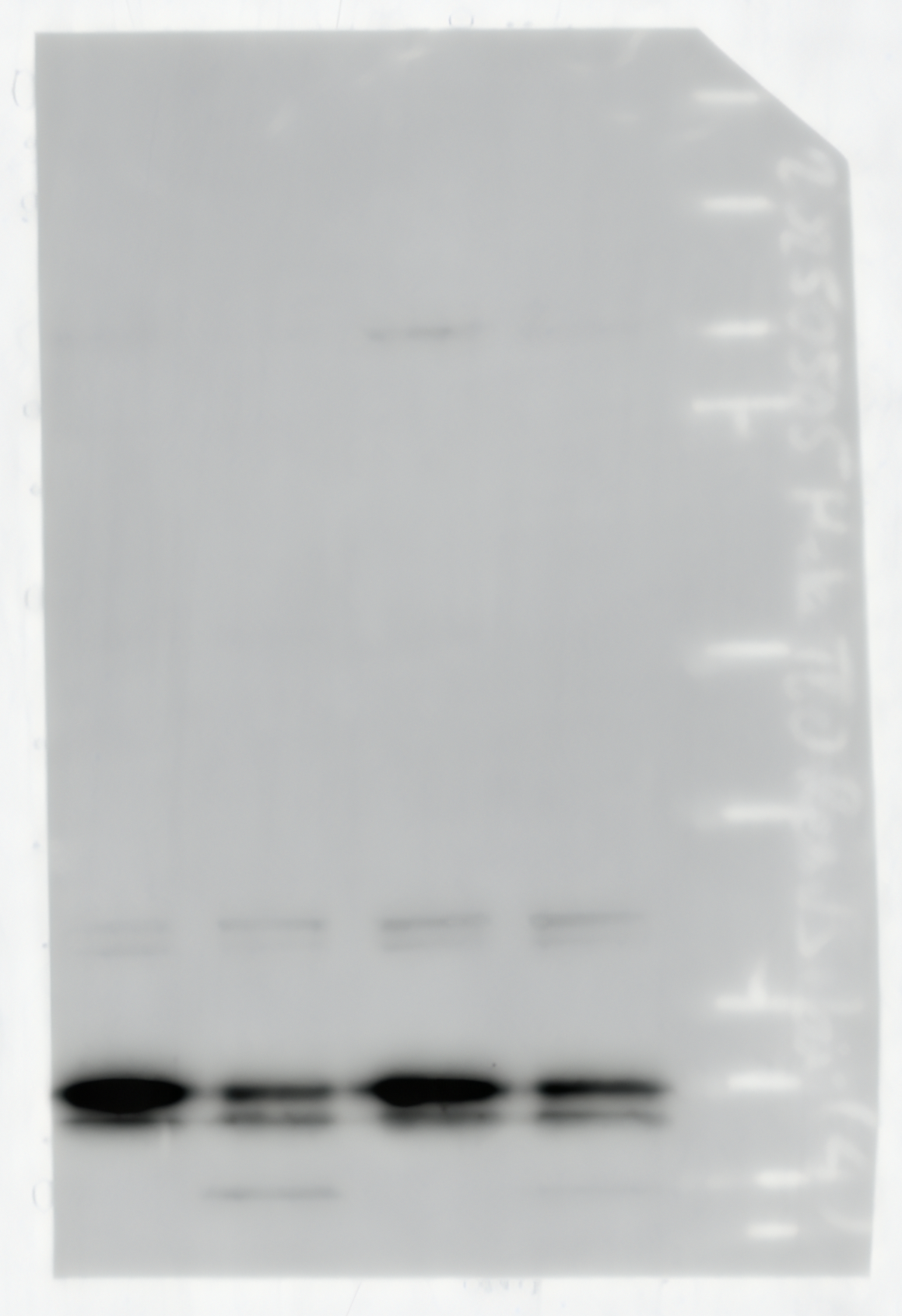

Supplement: Supplementary file 5 — Source data Fig. 4 [file 44320_2025_150_MOESM5_ESM.zip › SD_Figure_4/Fig_4A_Westen_blots/Bid.tif]

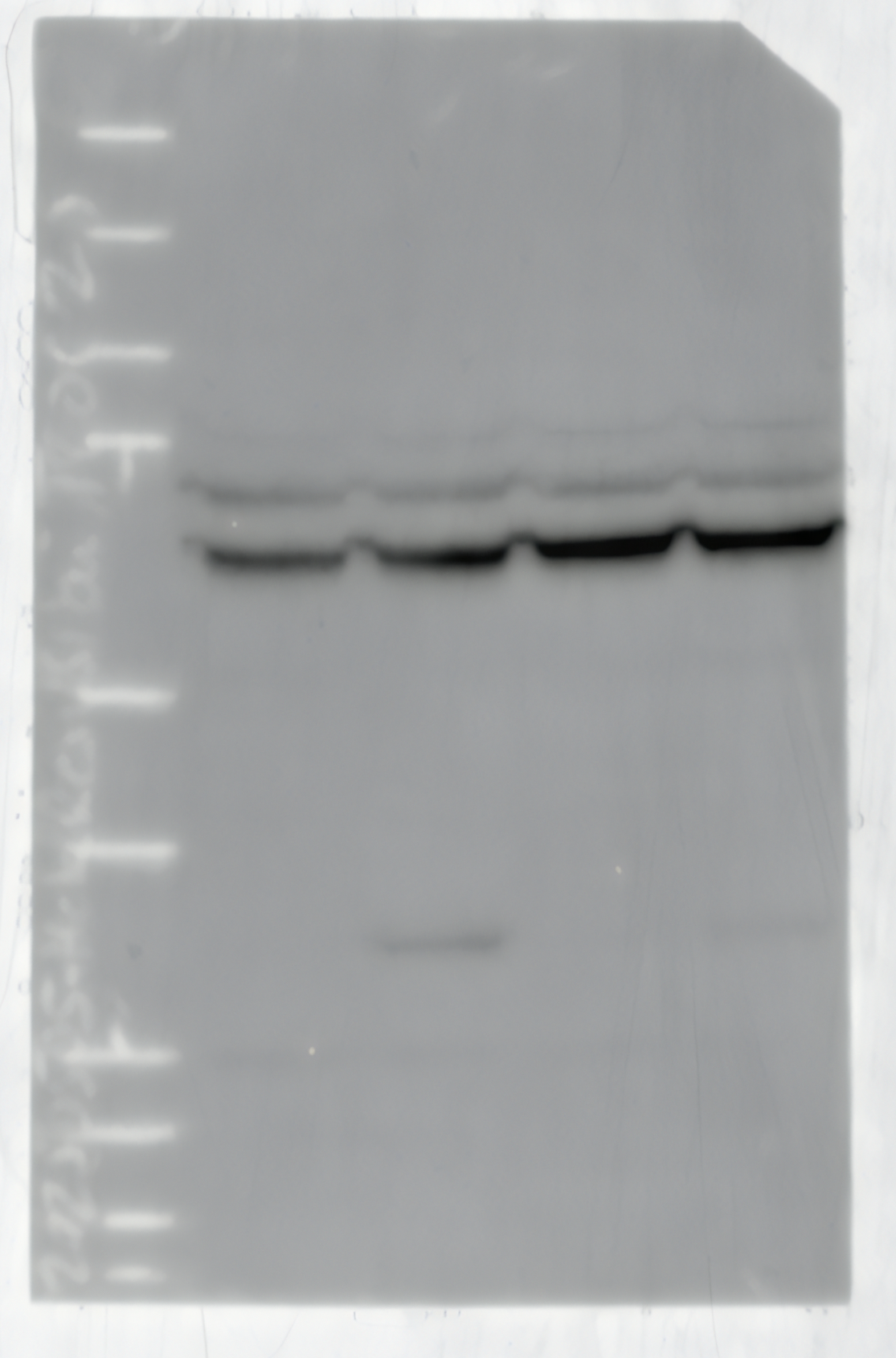

Supplement: Supplementary file 5 — Source data Fig. 4 [file 44320_2025_150_MOESM5_ESM.zip › SD_Figure_4/Fig_4A_Westen_blots/RIPK3.tif]

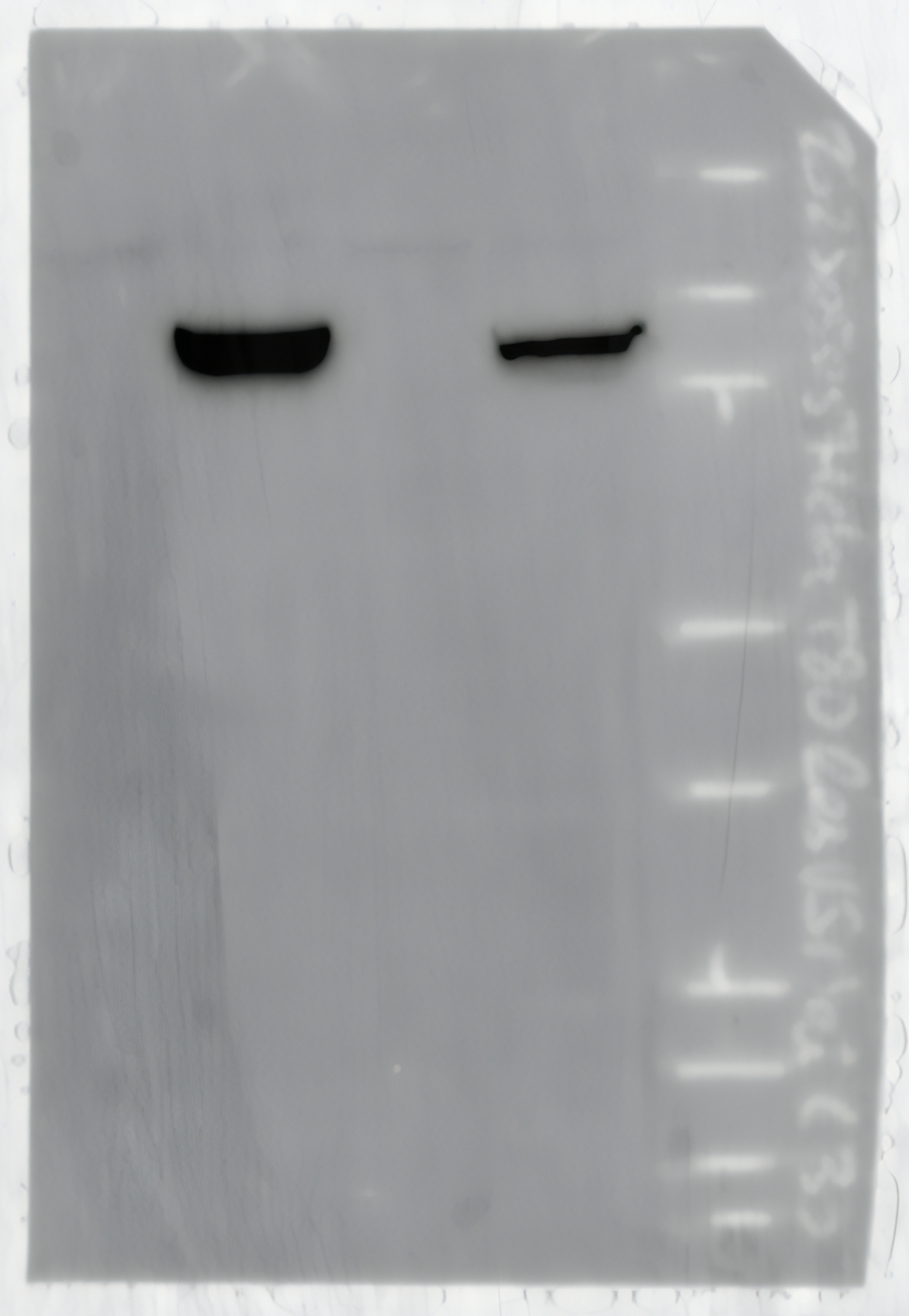

Supplement: Supplementary file 5 — Source data Fig. 4 [file 44320_2025_150_MOESM5_ESM.zip › SD_Figure_4/Fig_4A_Westen_blots/PARP.tif]

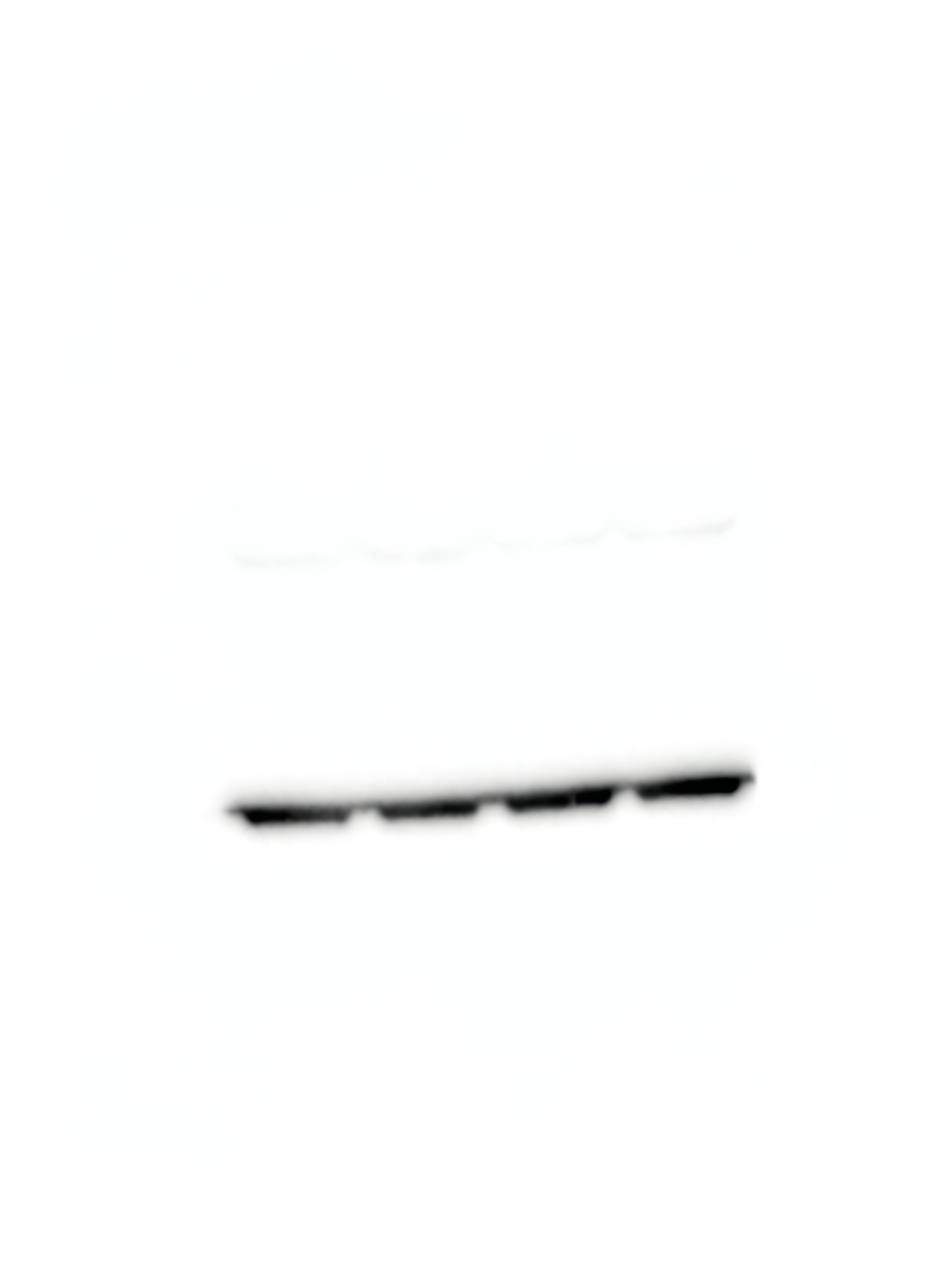

Supplement: Supplementary file 5 — Source data Fig. 4 [file 44320_2025_150_MOESM5_ESM.zip › SD_Figure_4/Fig_4A_Westen_blots/GAPDH.tif]

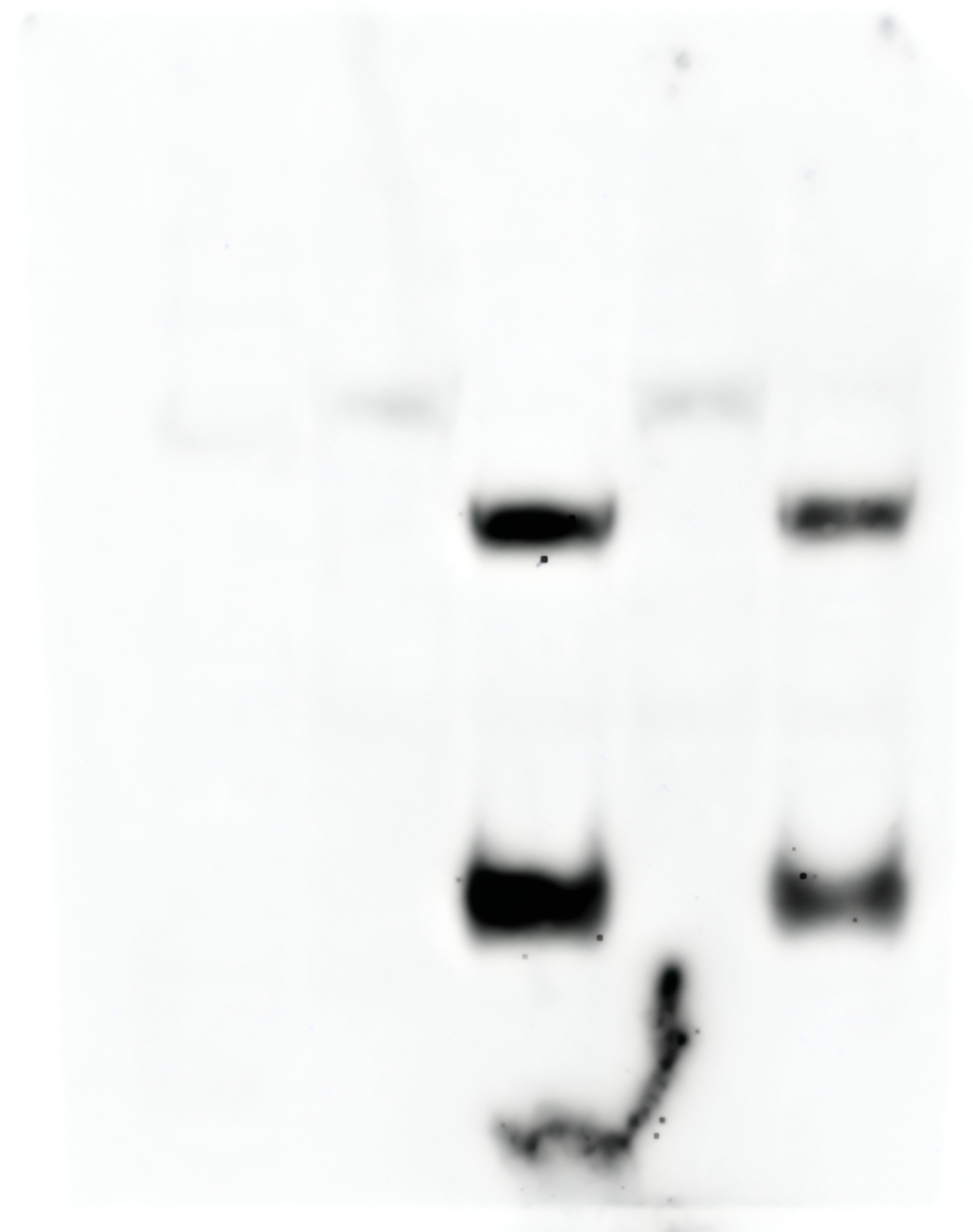

Supplement: Supplementary file 5 — Source data Fig. 4 [file 44320_2025_150_MOESM5_ESM.zip › SD_Figure_4/Fig_4A_Westen_blots/CASPASE8.tif]

## Slide 1
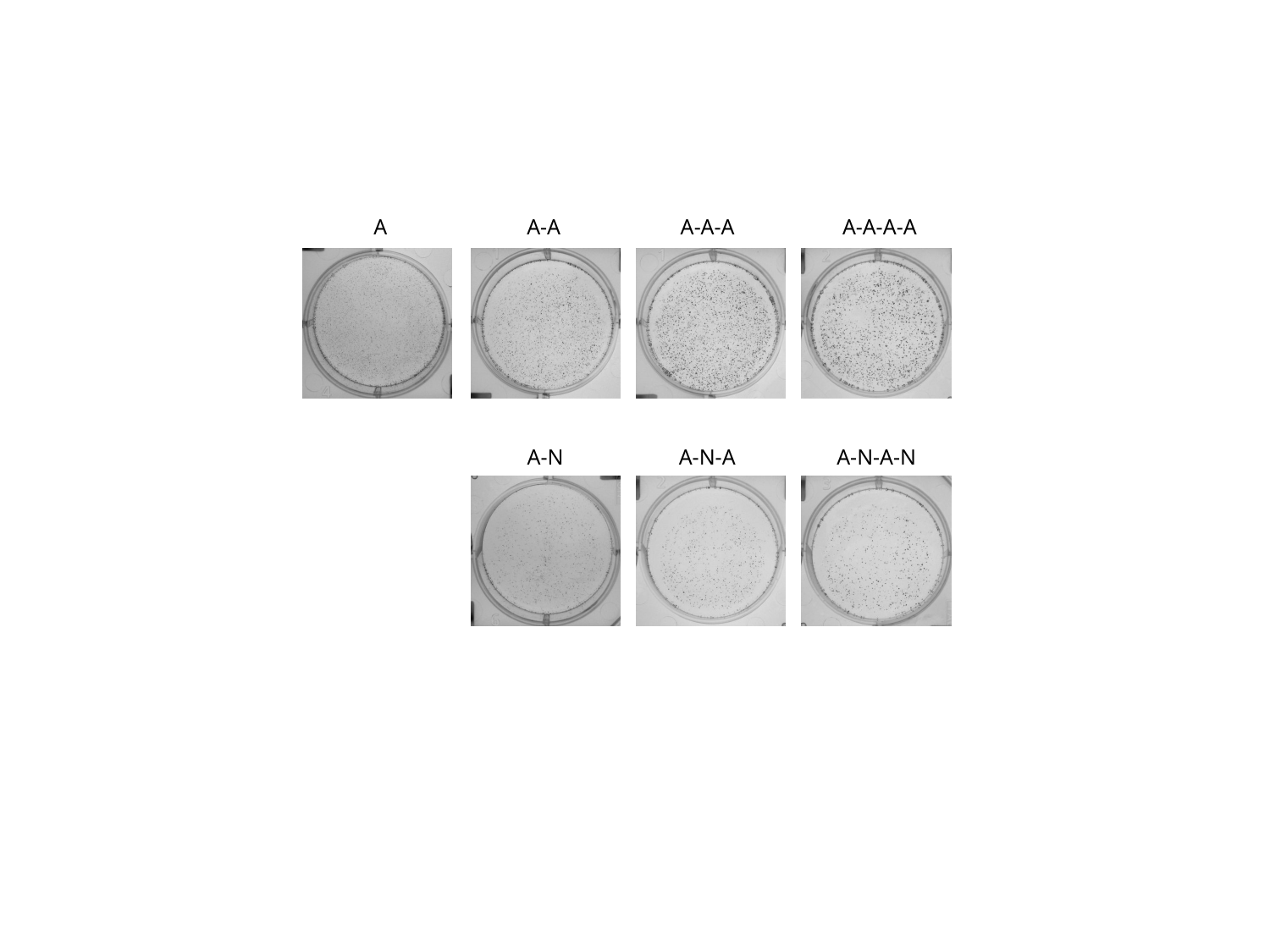

A
A-A
A-A-A
A-A-A-A
A-N
A-N-A
A-N-A-N

Supplement: Supplementary file 6 — Source data Fig. 5 [file 44320_2025_150_MOESM6_ESM.zip › SD_Figure_5/Fig_5B_cell_density_images.pptx]

TRAIL/TBQ phenotypic switch Model A 8, Model N 7  
RMSE AAAA = 3.8738, RMSE ANAN = 1.4018

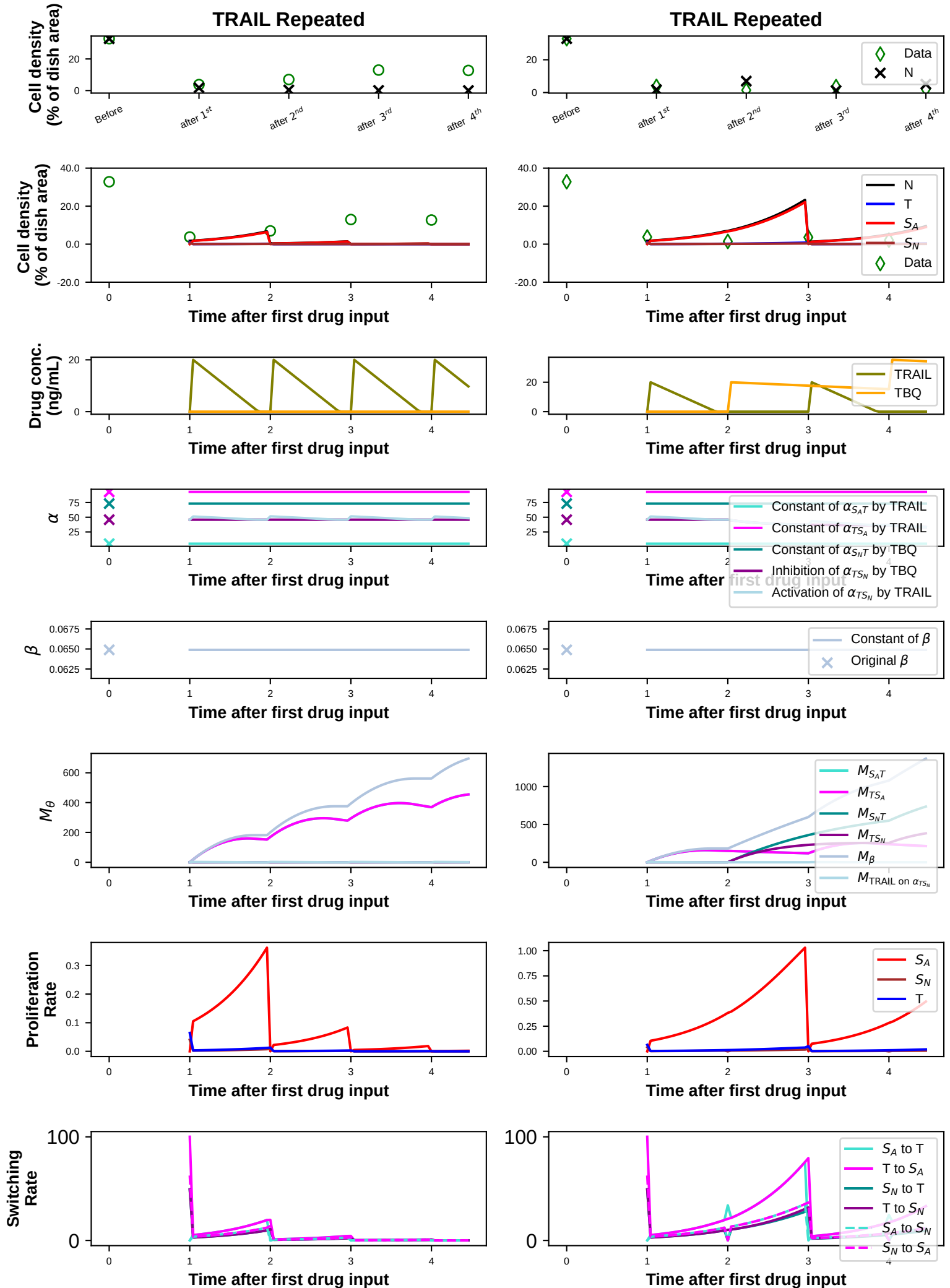

Supplement: Supplementary file 7 — Appendix Simulations Results [file 44320_2025_150_MOESM7_ESM.zip › Appendix_Simulations_Results/PSM2D_Simulations/PSM2_A_8_N_7.pdf]

TRAIL/TBQ phenotypic switch Model A 1, Model N 4  
RMSE AAAA = 1.4527, RMSE ANAN = 0.9567

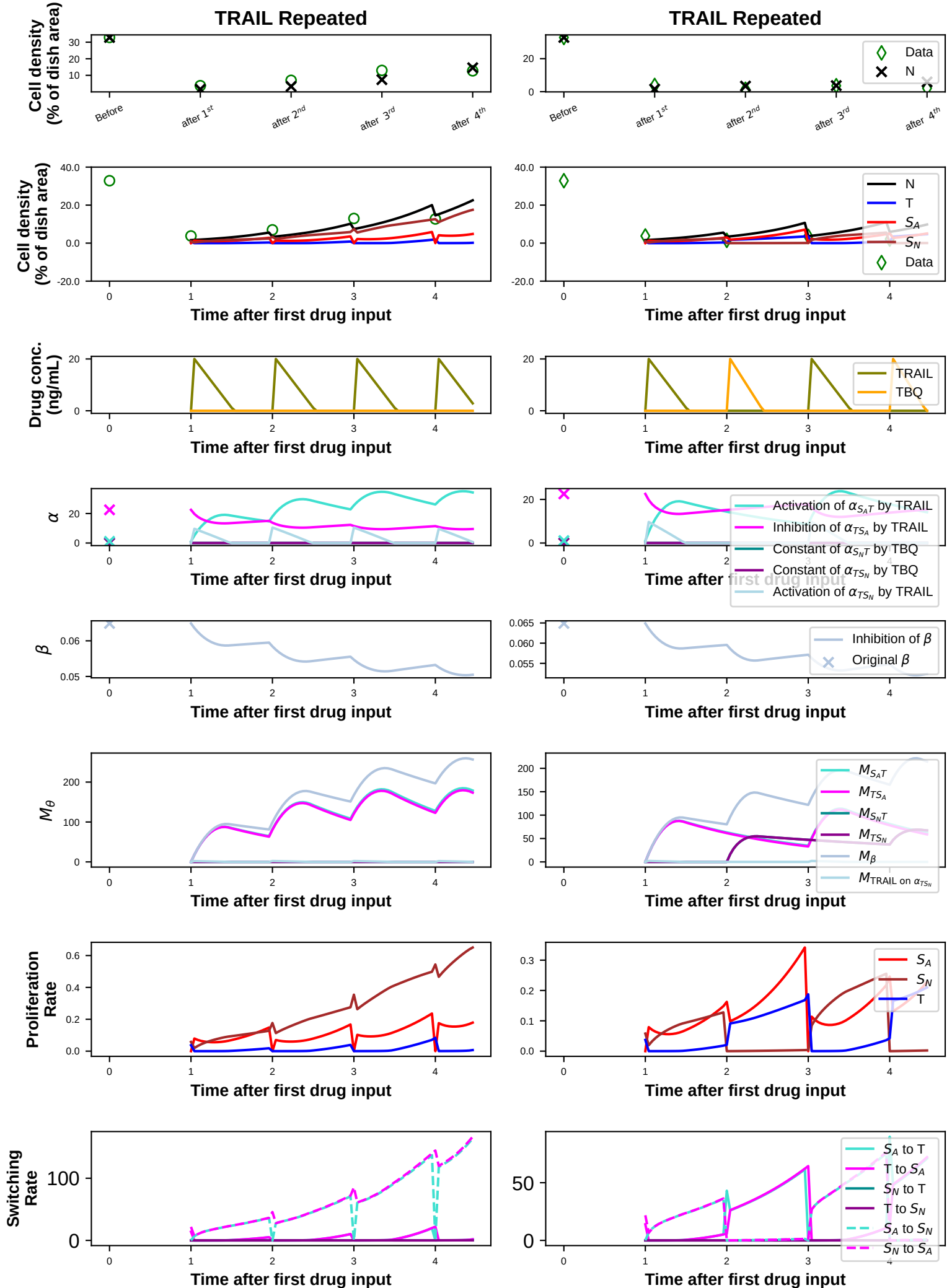

Supplement: Supplementary file 7 — Appendix Simulations Results [file 44320_2025_150_MOESM7_ESM.zip › Appendix_Simulations_Results/PSM2D_Simulations/PSM2_A_1_N_4.pdf]

# TRAIL/TBQ phenotypic switch Model A 1, Model N 5

RMSE AAAA = 1.4511, RMSE ANAN = 0.8012

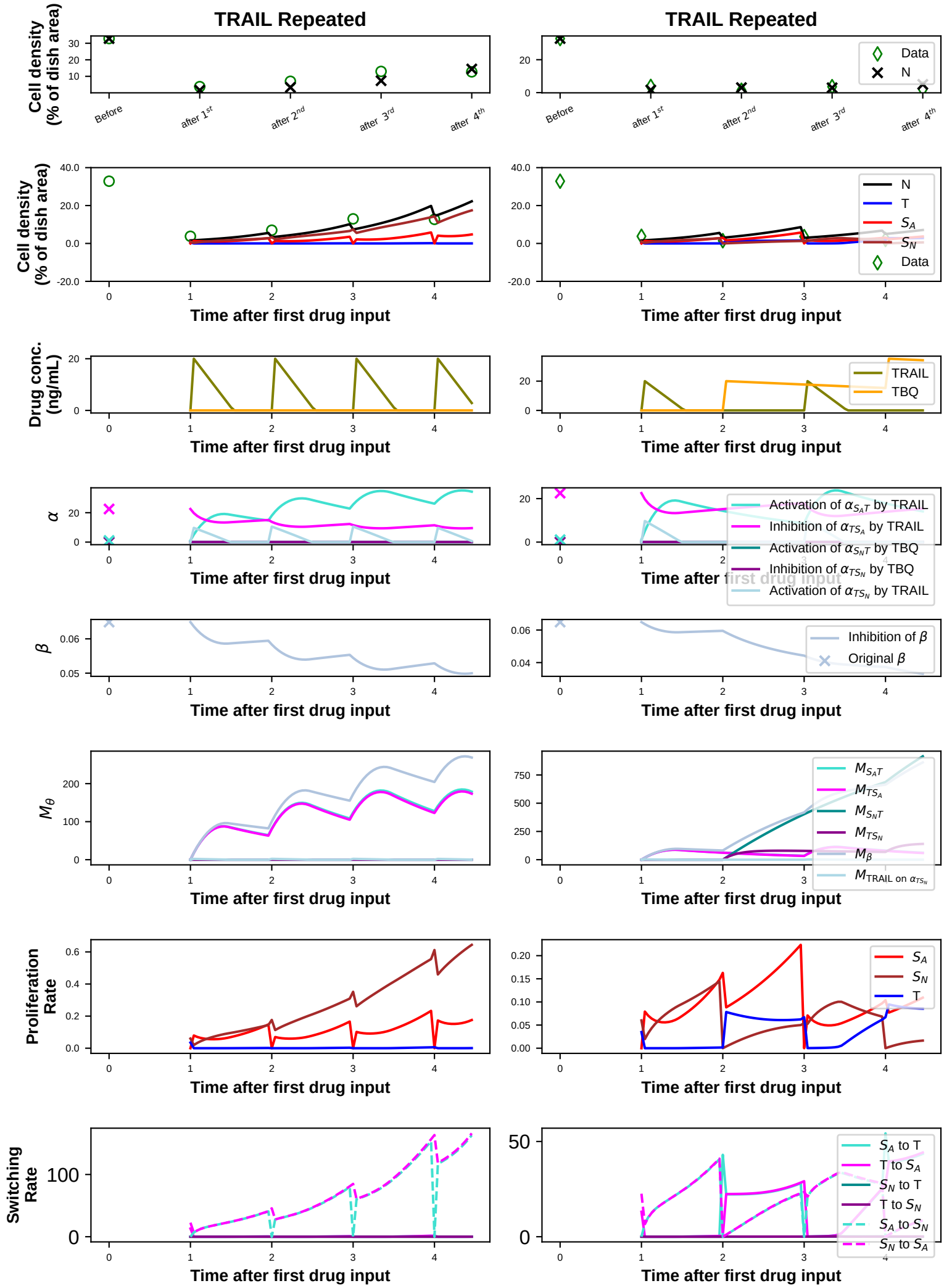

Supplement: Supplementary file 7 — Appendix Simulations Results [file 44320_2025_150_MOESM7_ESM.zip › Appendix_Simulations_Results/PSM2D_Simulations/PSM2_A_1_N_5.pdf]

TRAIL/TBQ phenotypic switch Model A 8, Model N 6  
RMSE AAAA = 3.8738, RMSE ANAN = 1.3493

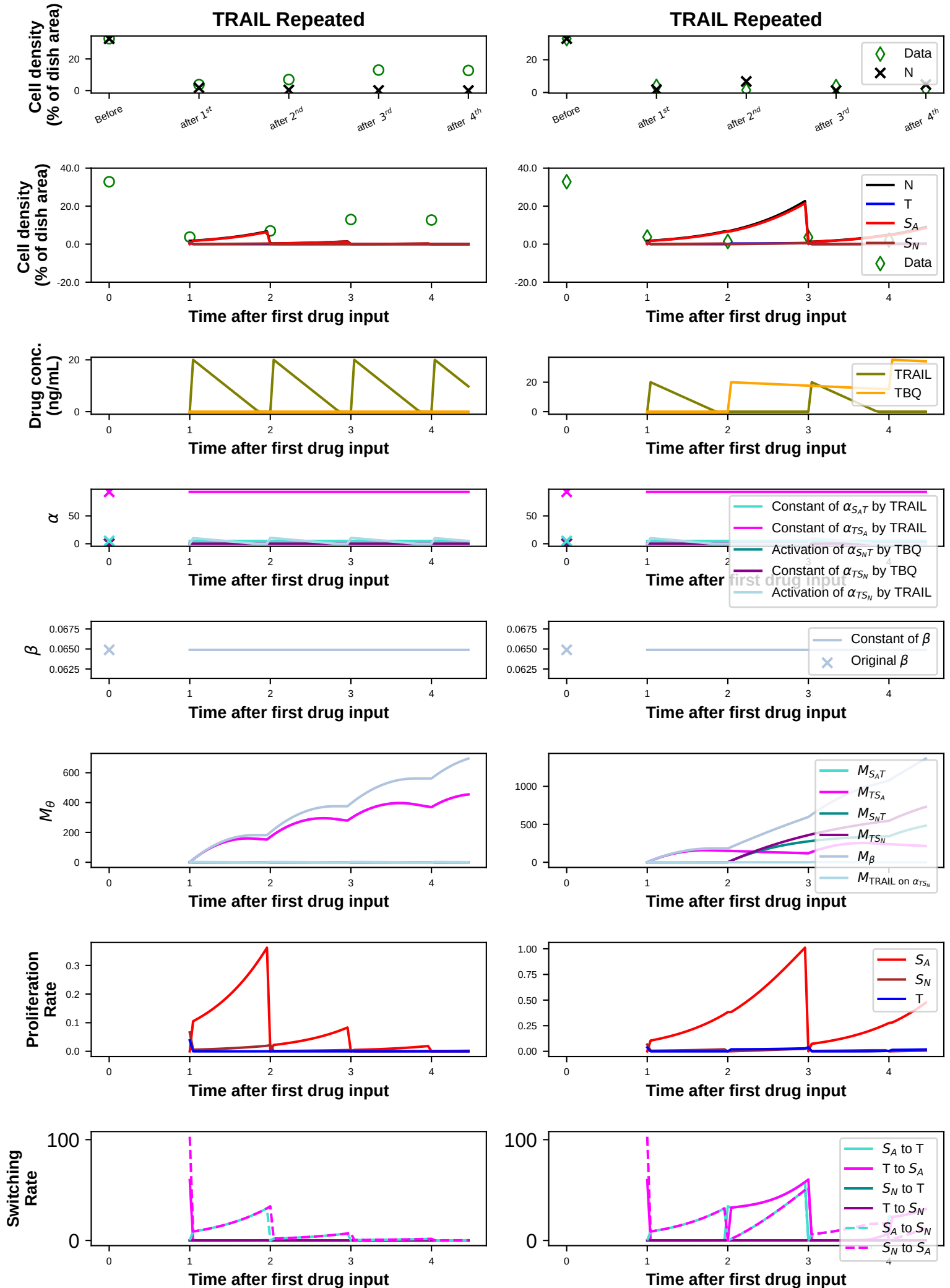

Supplement: Supplementary file 7 — Appendix Simulations Results [file 44320_2025_150_MOESM7_ESM.zip › Appendix_Simulations_Results/PSM2D_Simulations/PSM2_A_8_N_6.pdf]

TRAIL/TBQ phenotypic switch Model A 4, Model N 8  
RMSE AAAA = 1.6096, RMSE ANAN = 4.7242

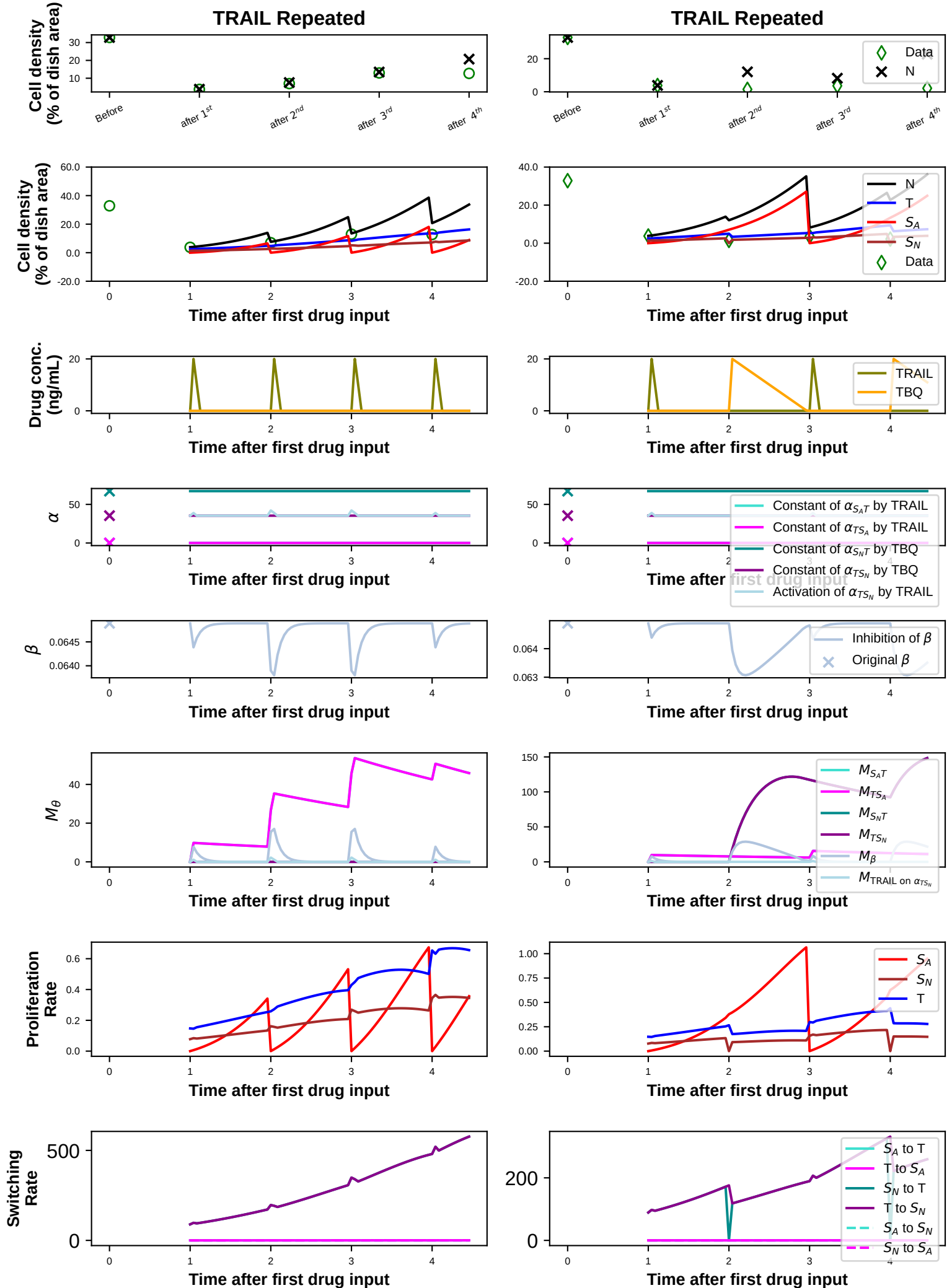

Supplement: Supplementary file 7 — Appendix Simulations Results [file 44320_2025_150_MOESM7_ESM.zip › Appendix_Simulations_Results/PSM2D_Simulations/PSM2_A_4_N_8.pdf]

TRAIL/TBQ phenotypic switch Model A 8, Model N 4  
RMSE AAAA = 3.8738, RMSE ANAN = 1.3491

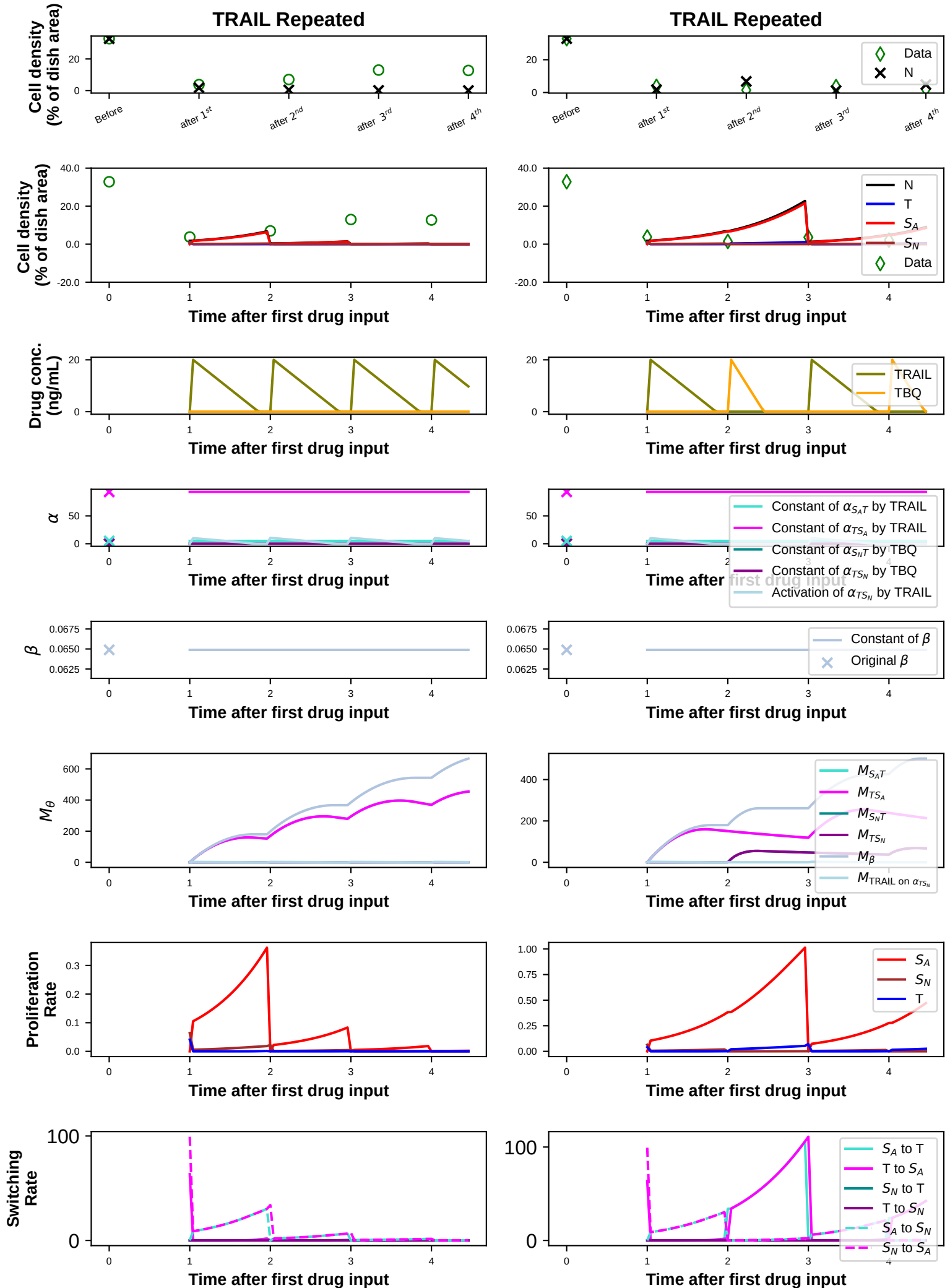

Supplement: Supplementary file 7 — Appendix Simulations Results [file 44320_2025_150_MOESM7_ESM.zip › Appendix_Simulations_Results/PSM2D_Simulations/PSM2_A_8_N_4.pdf]

TRAIL/TBQ phenotypic switch Model A 1, Model N 7  
RMSE AAAA = 1.4511, RMSE ANAN = 1.547

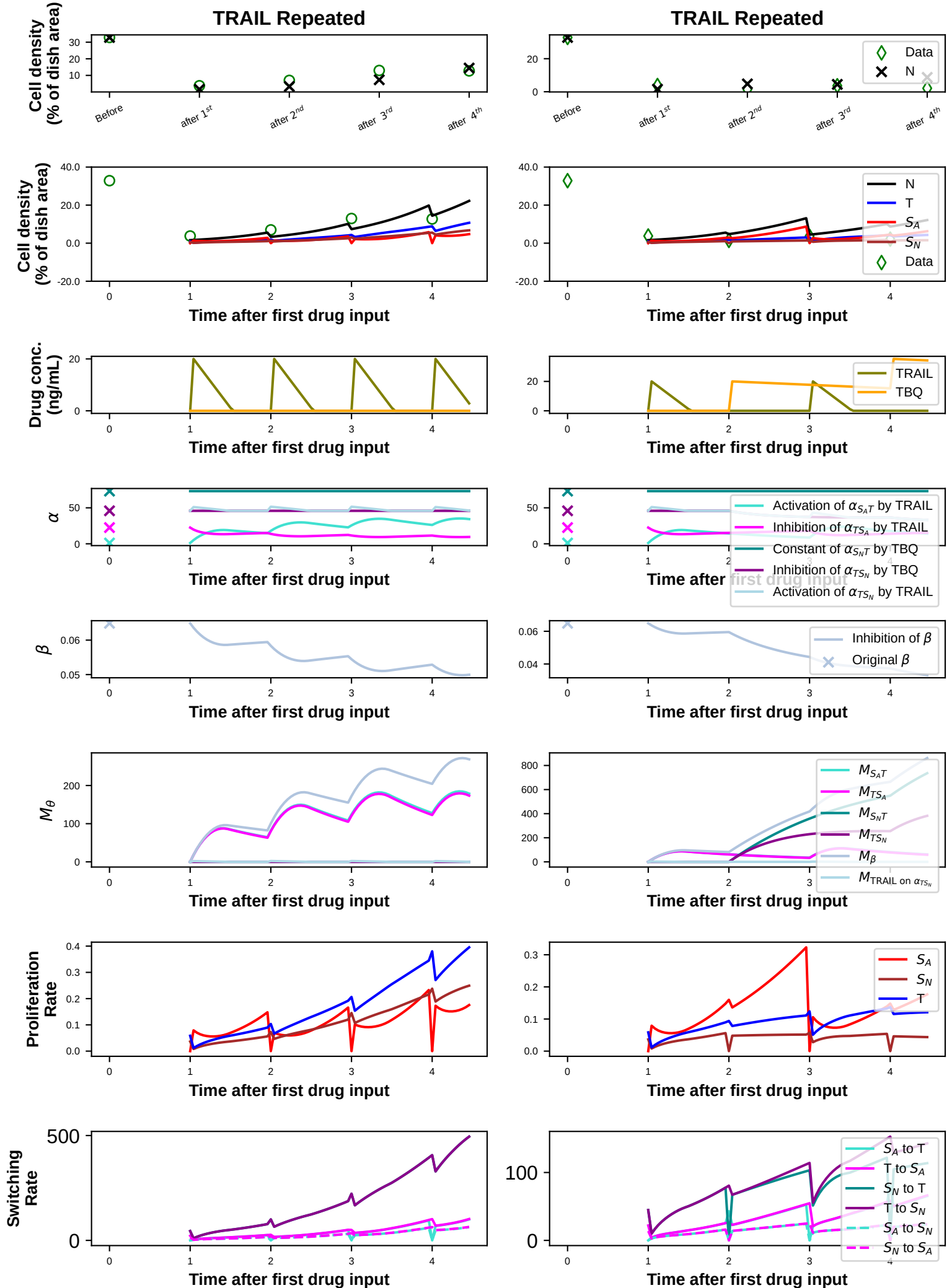

Supplement: Supplementary file 7 — Appendix Simulations Results [file 44320_2025_150_MOESM7_ESM.zip › Appendix_Simulations_Results/PSM2D_Simulations/PSM2_A_1_N_7.pdf]

# TRAIL/TBQ phenotypic switch Model A 1, Model N 6

RMSE AAAA = 1.4511, RMSE ANAN = 0.7621

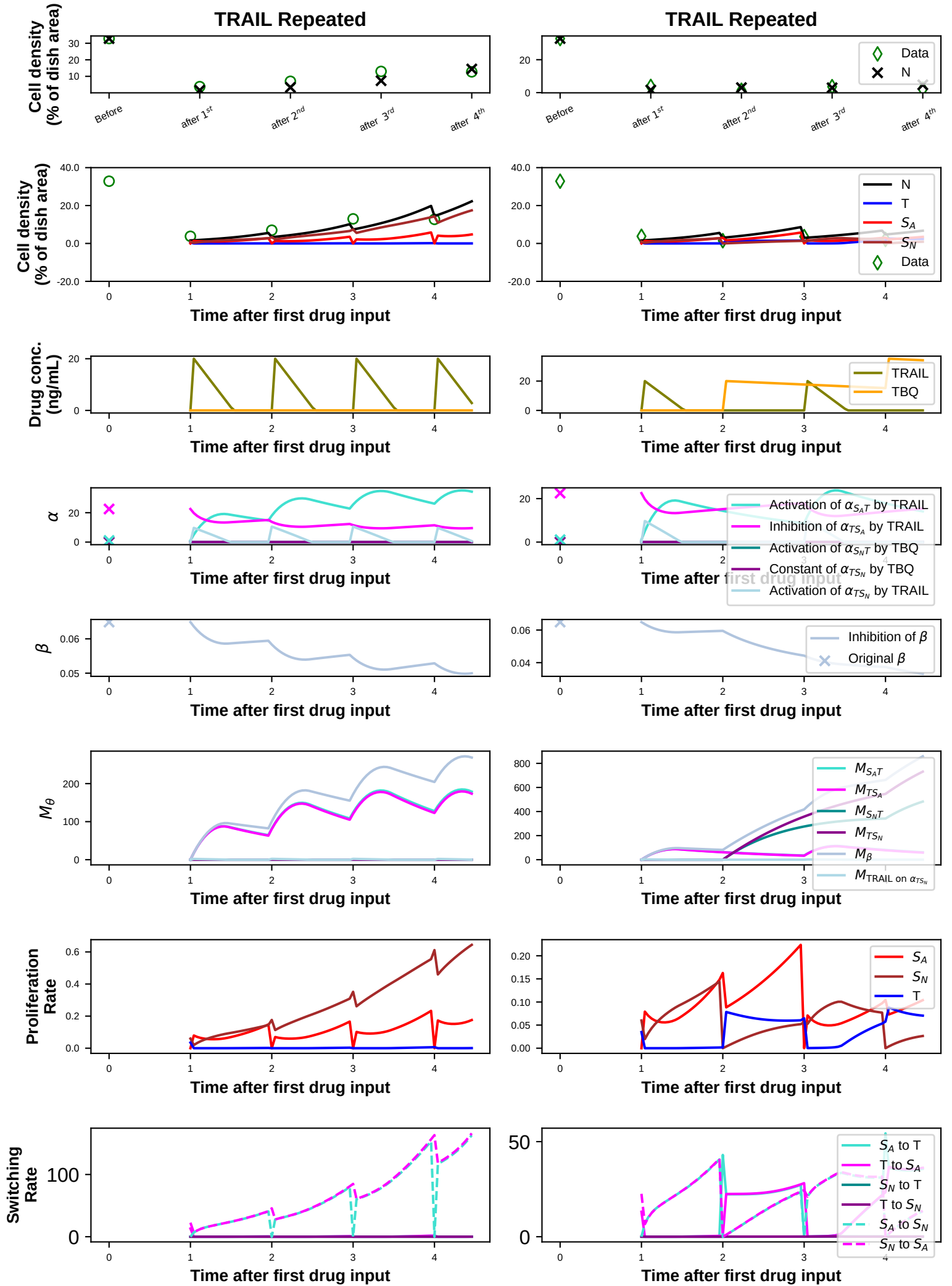

Supplement: Supplementary file 7 — Appendix Simulations Results [file 44320_2025_150_MOESM7_ESM.zip › Appendix_Simulations_Results/PSM2D_Simulations/PSM2_A_1_N_6.pdf]

# TRAIL/TBQ phenotypic switch Model A 8, Model N 5

RMSE AAAA = 3.8738, RMSE ANAN = 1.3502

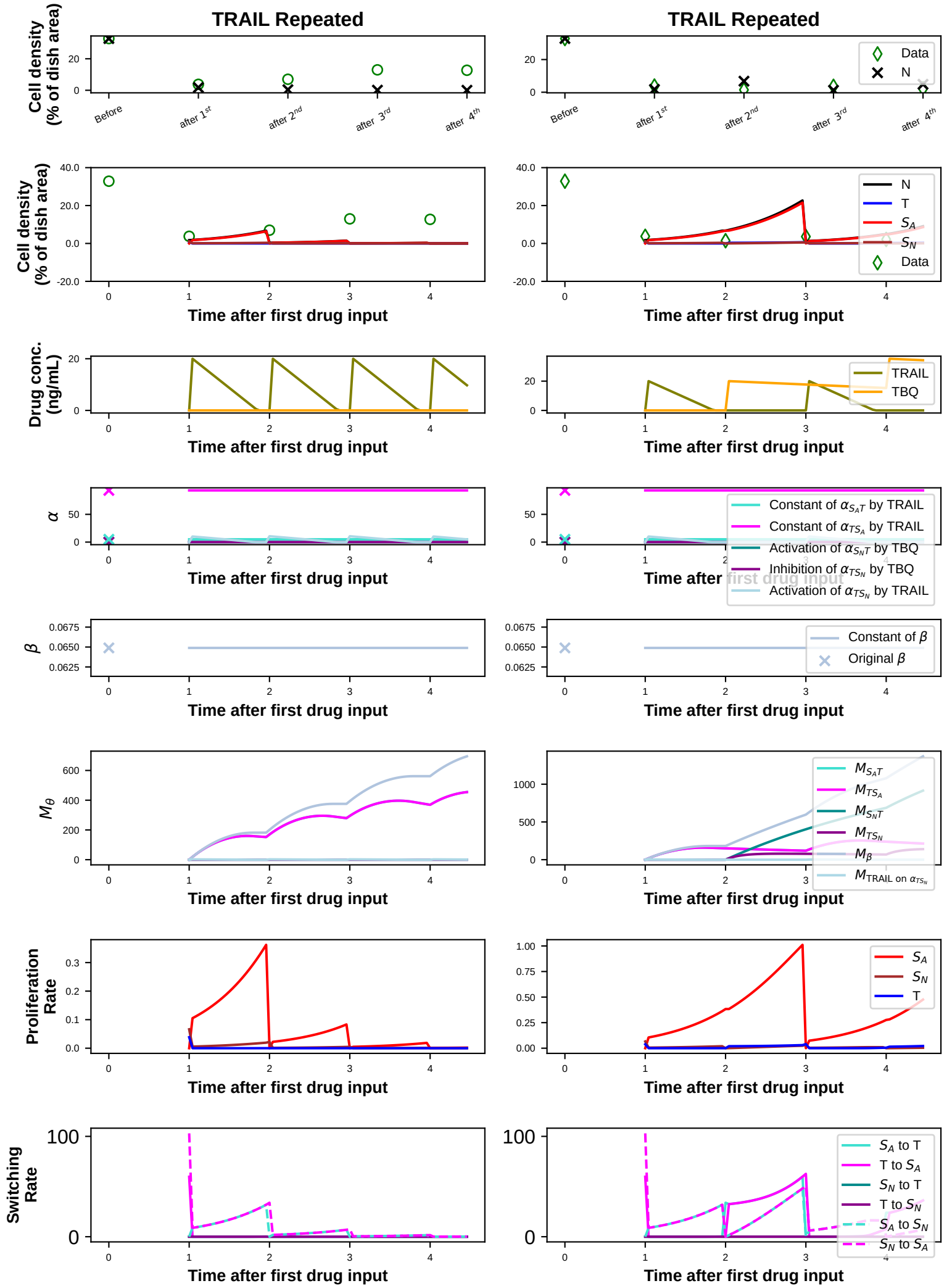

Supplement: Supplementary file 7 — Appendix Simulations Results [file 44320_2025_150_MOESM7_ESM.zip › Appendix_Simulations_Results/PSM2D_Simulations/PSM2_A_8_N_5.pdf]

# TRAIL/TBQ phenotypic switch Model A 8, Model N 1

RMSE AAAA = 3.8738, RMSE ANAN = 1.3833

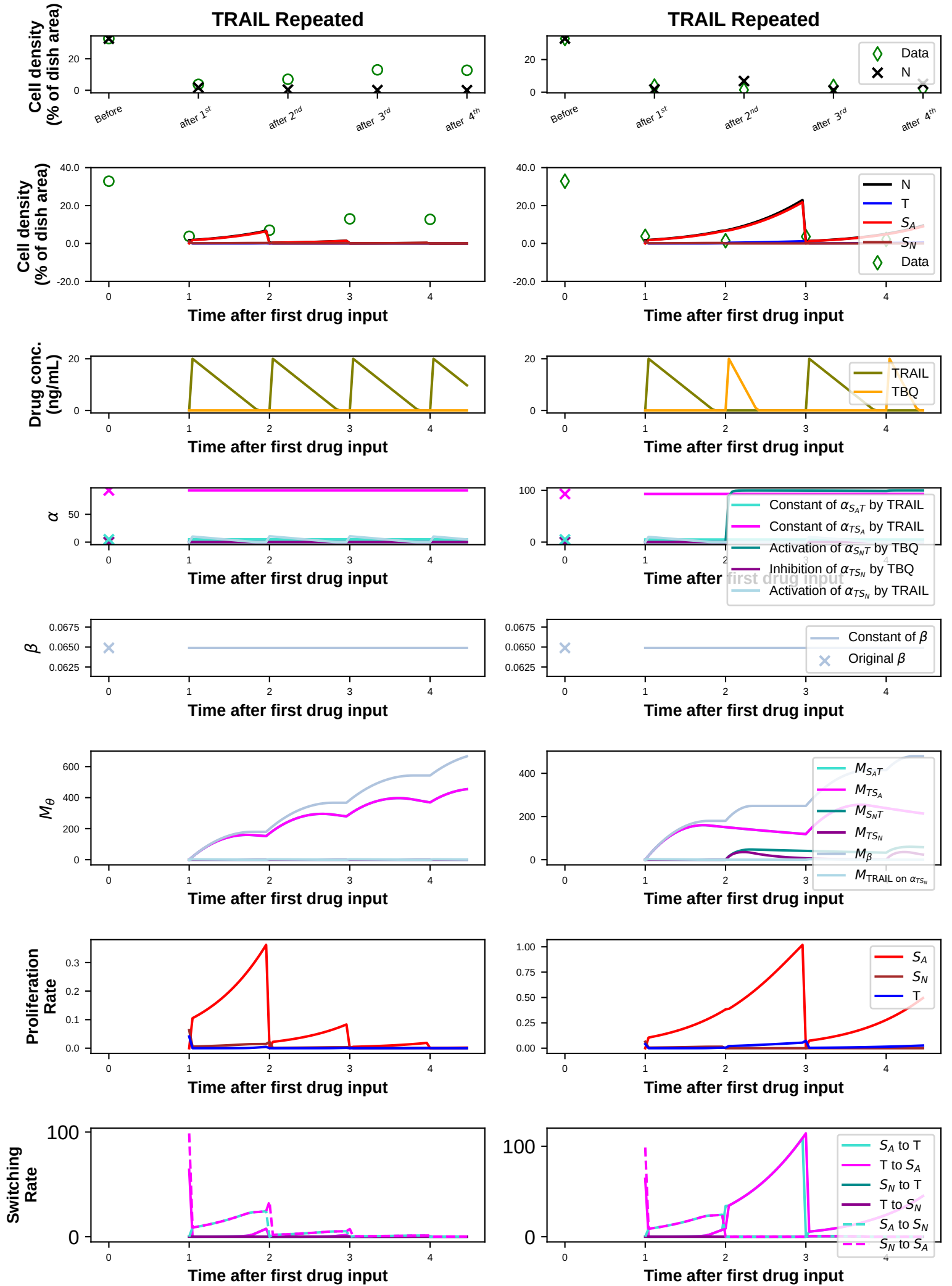

Supplement: Supplementary file 7 — Appendix Simulations Results [file 44320_2025_150_MOESM7_ESM.zip › Appendix_Simulations_Results/PSM2D_Simulations/PSM2_A_8_N_1.pdf]

# TRAIL/TBQ phenotypic switch Model A 1, Model N 2

RMSE AAAA = 1.4526, RMSE ANAN = 1.739

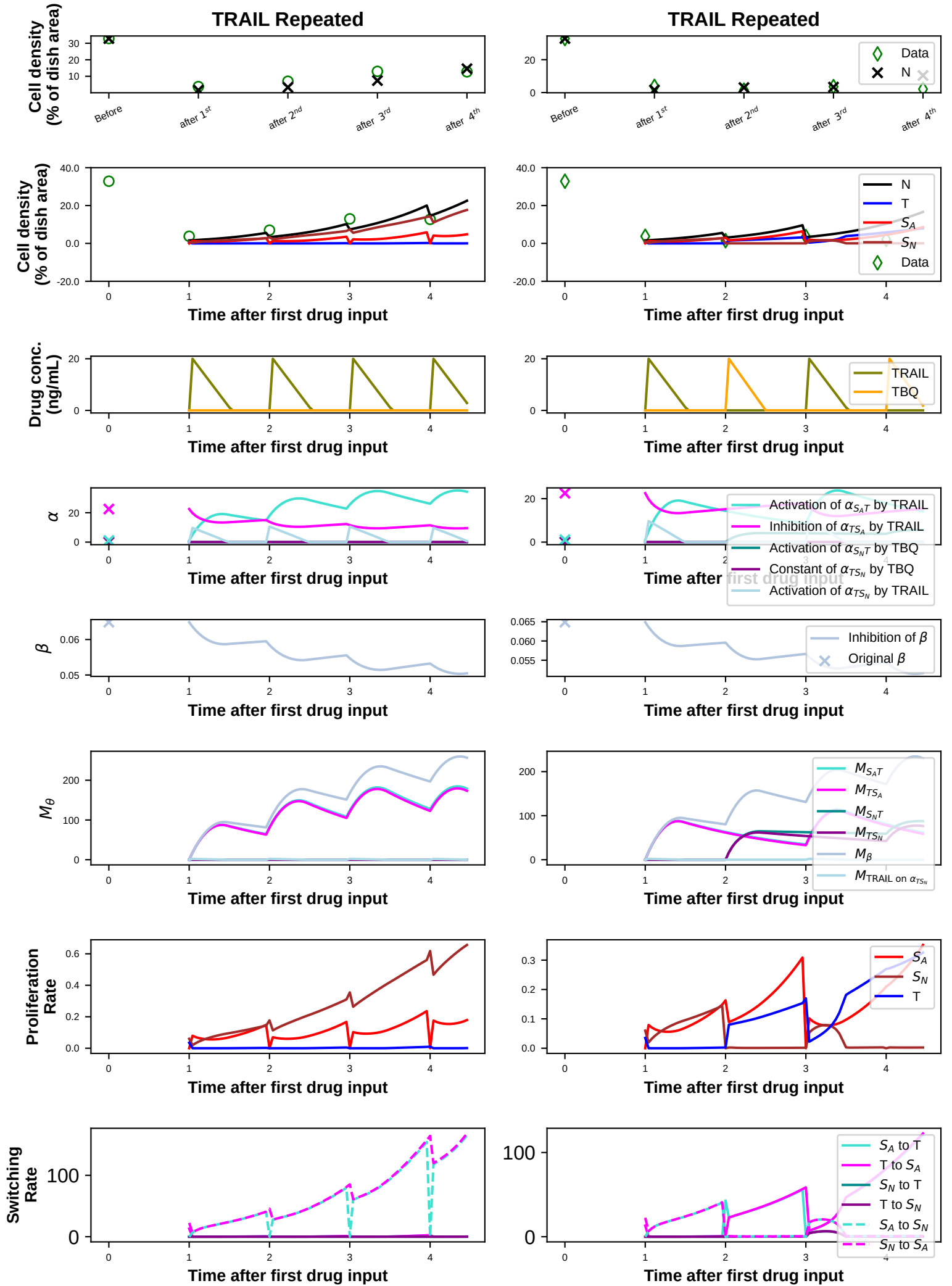

Supplement: Supplementary file 7 — Appendix Simulations Results [file 44320_2025_150_MOESM7_ESM.zip › Appendix_Simulations_Results/PSM2D_Simulations/PSM2_A_1_N_2.pdf]

**RMSE AAAA = 1.4527, RMSE ANAN = 2.8392**

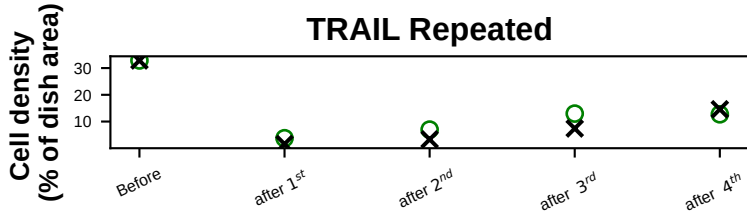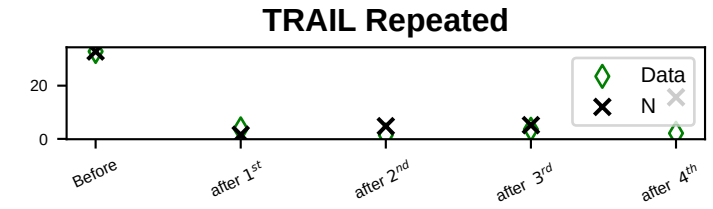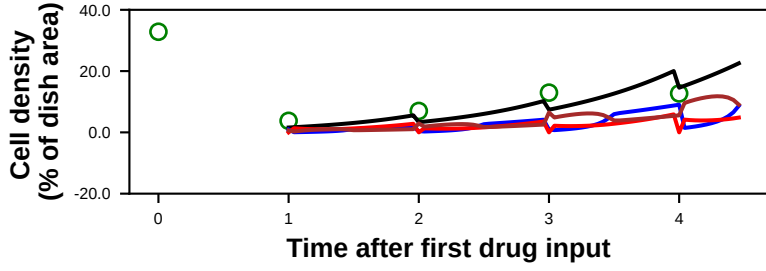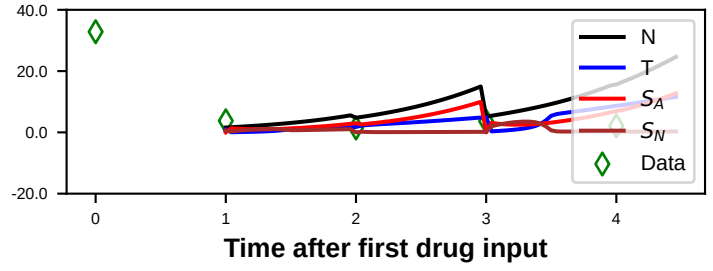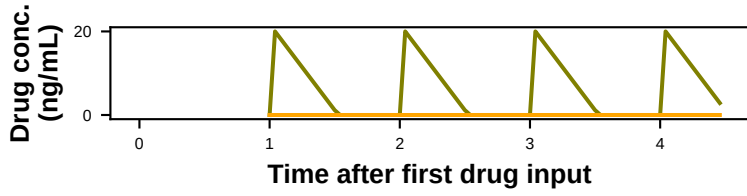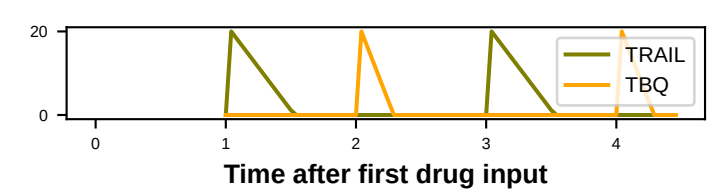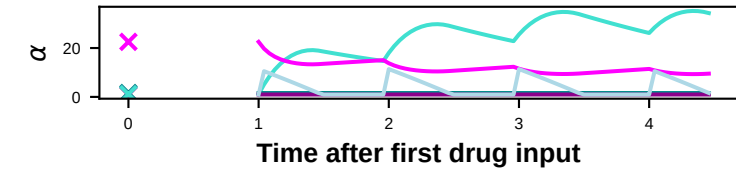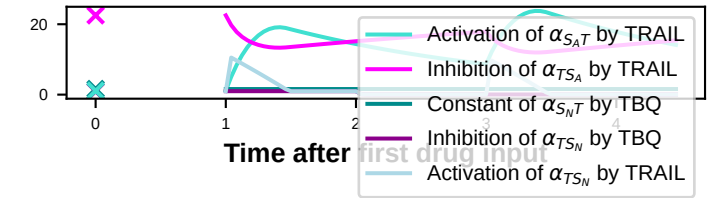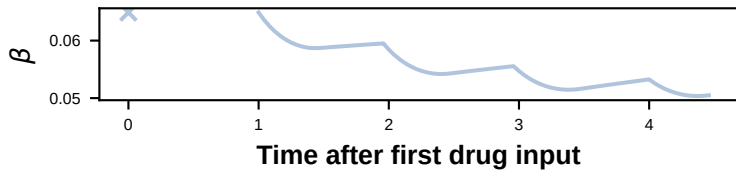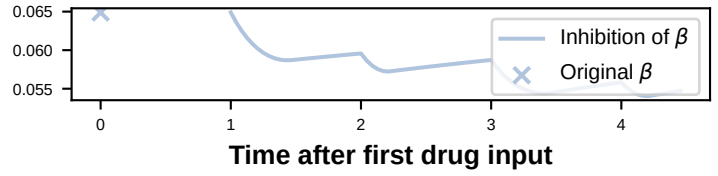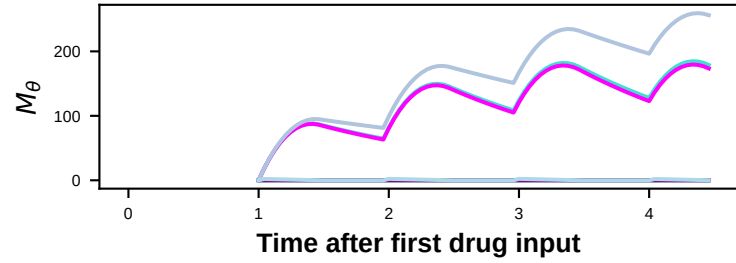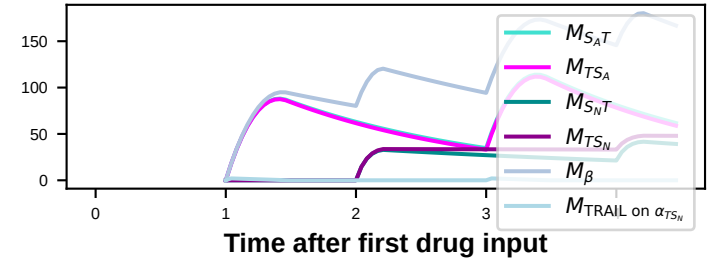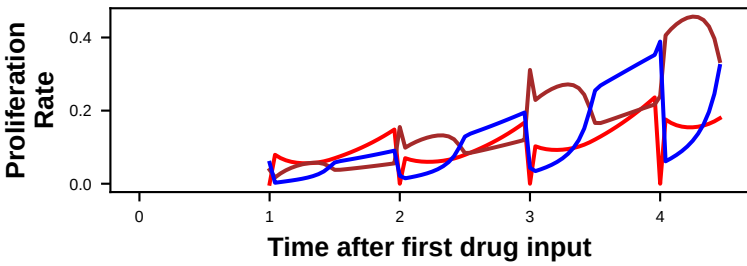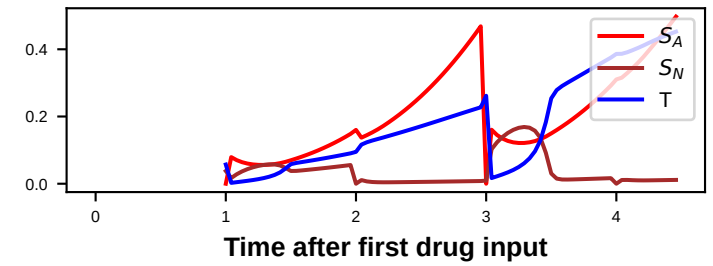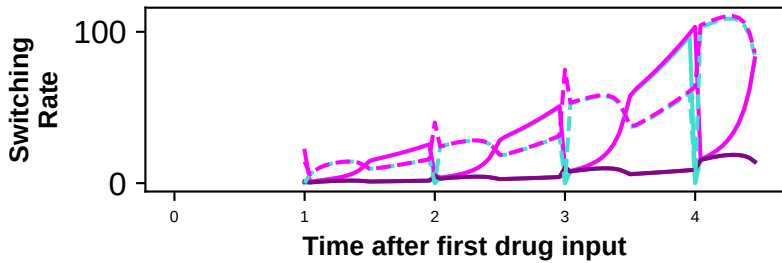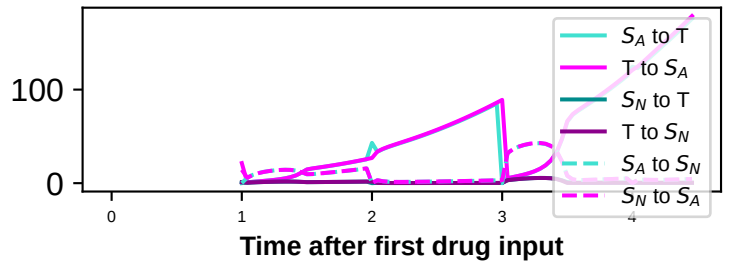

Supplement: Supplementary file 7 — Appendix Simulations Results [file 44320_2025_150_MOESM7_ESM.zip › Appendix_Simulations_Results/PSM2D_Simulations/PSM2_A_1_N_3.pdf]

TRAIL/TBQ phenotypic switch Model A 8, Model N 2  
RMSE AAAA = 3.8738, RMSE ANAN = 1.3648

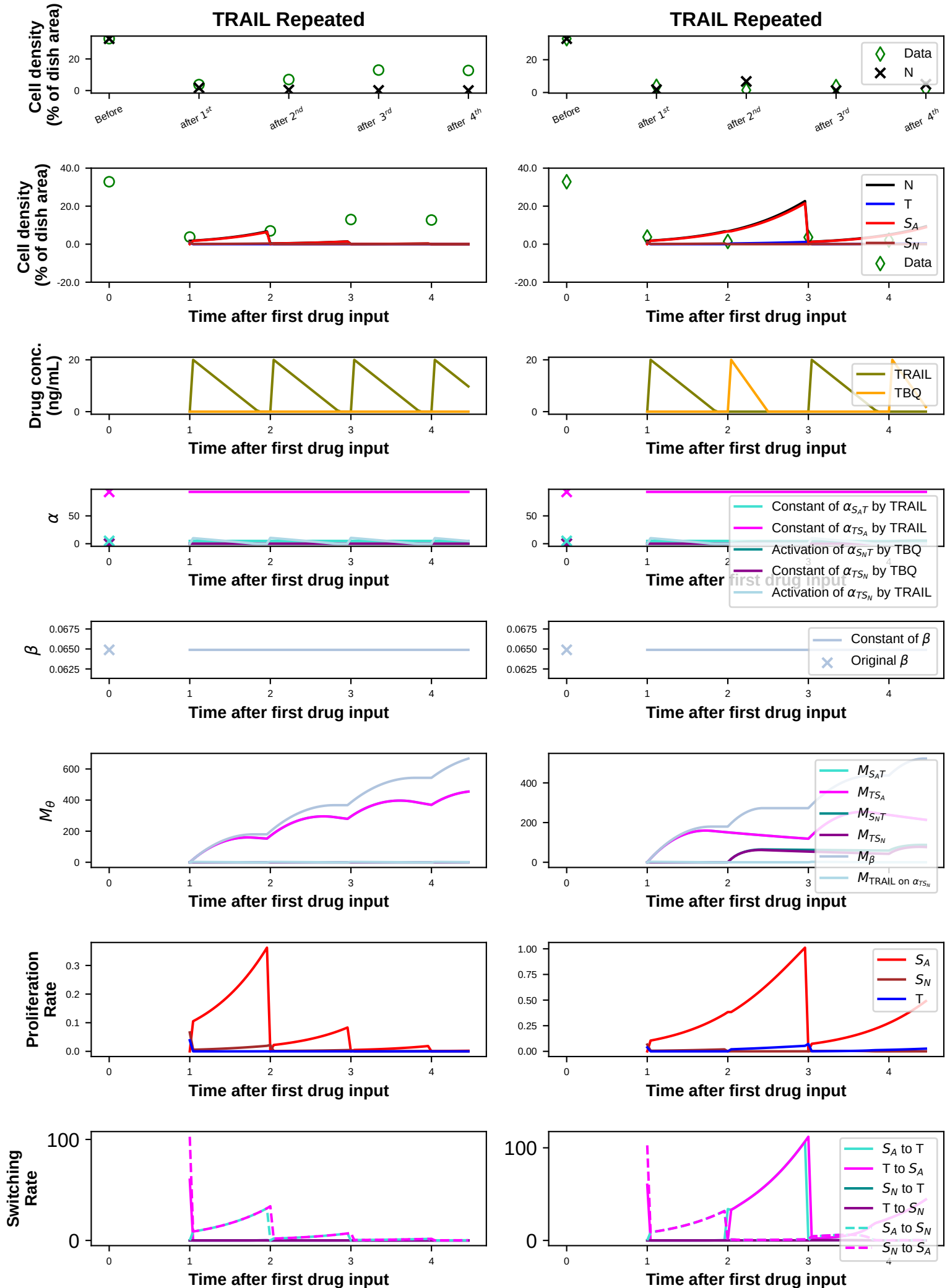

Supplement: Supplementary file 7 — Appendix Simulations Results [file 44320_2025_150_MOESM7_ESM.zip › Appendix_Simulations_Results/PSM2D_Simulations/PSM2_A_8_N_2.pdf]

# TRAIL/TBQ phenotypic switch Model A 1, Model N 1

RMSE AAAA = 1.4527, RMSE ANAN = 2.4448

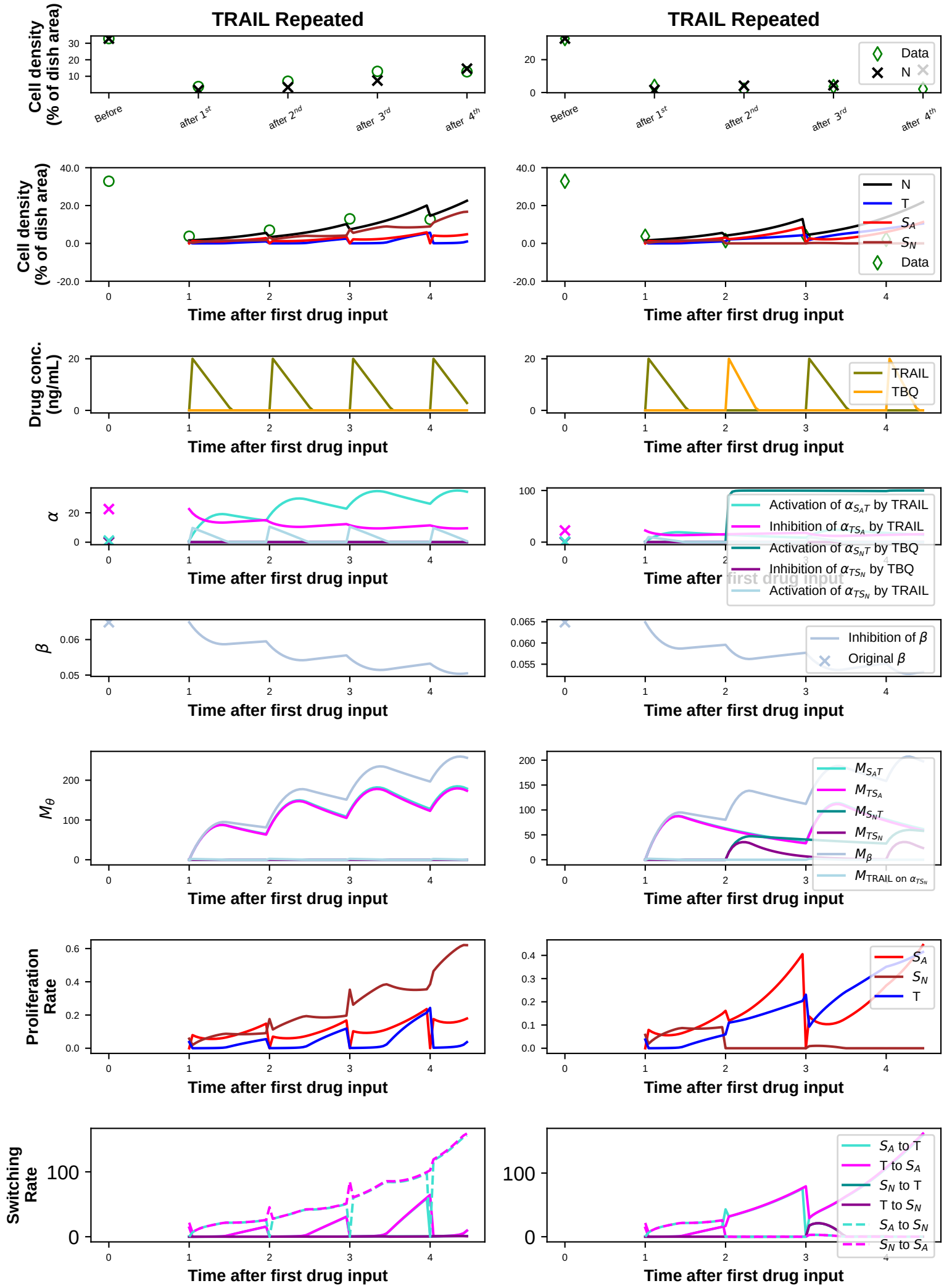

Supplement: Supplementary file 7 — Appendix Simulations Results [file 44320_2025_150_MOESM7_ESM.zip › Appendix_Simulations_Results/PSM2D_Simulations/PSM2_A_1_N_1.pdf]

TRAIL/TBQ phenotypic switch Model A 5, Model N 8  
RMSE AAAA = 2.0923, RMSE ANAN = 3.7089

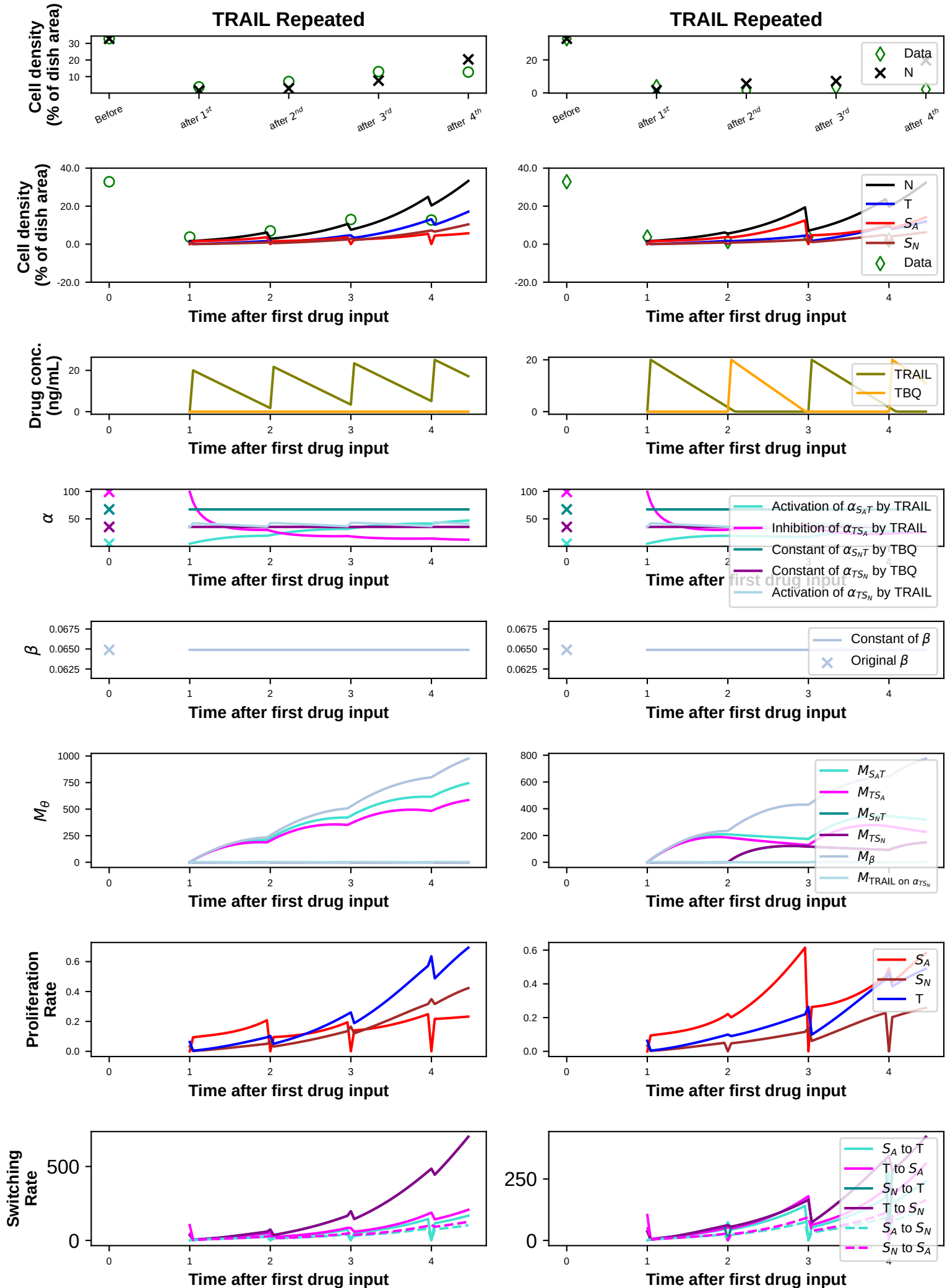

Supplement: Supplementary file 7 — Appendix Simulations Results [file 44320_2025_150_MOESM7_ESM.zip › Appendix_Simulations_Results/PSM2D_Simulations/PSM2_A_5_N_8.pdf]

TRAIL/TBQ phenotypic switch Model A 8, Model N 3  
RMSE AAAA = 3.8738, RMSE ANAN = 1.4076

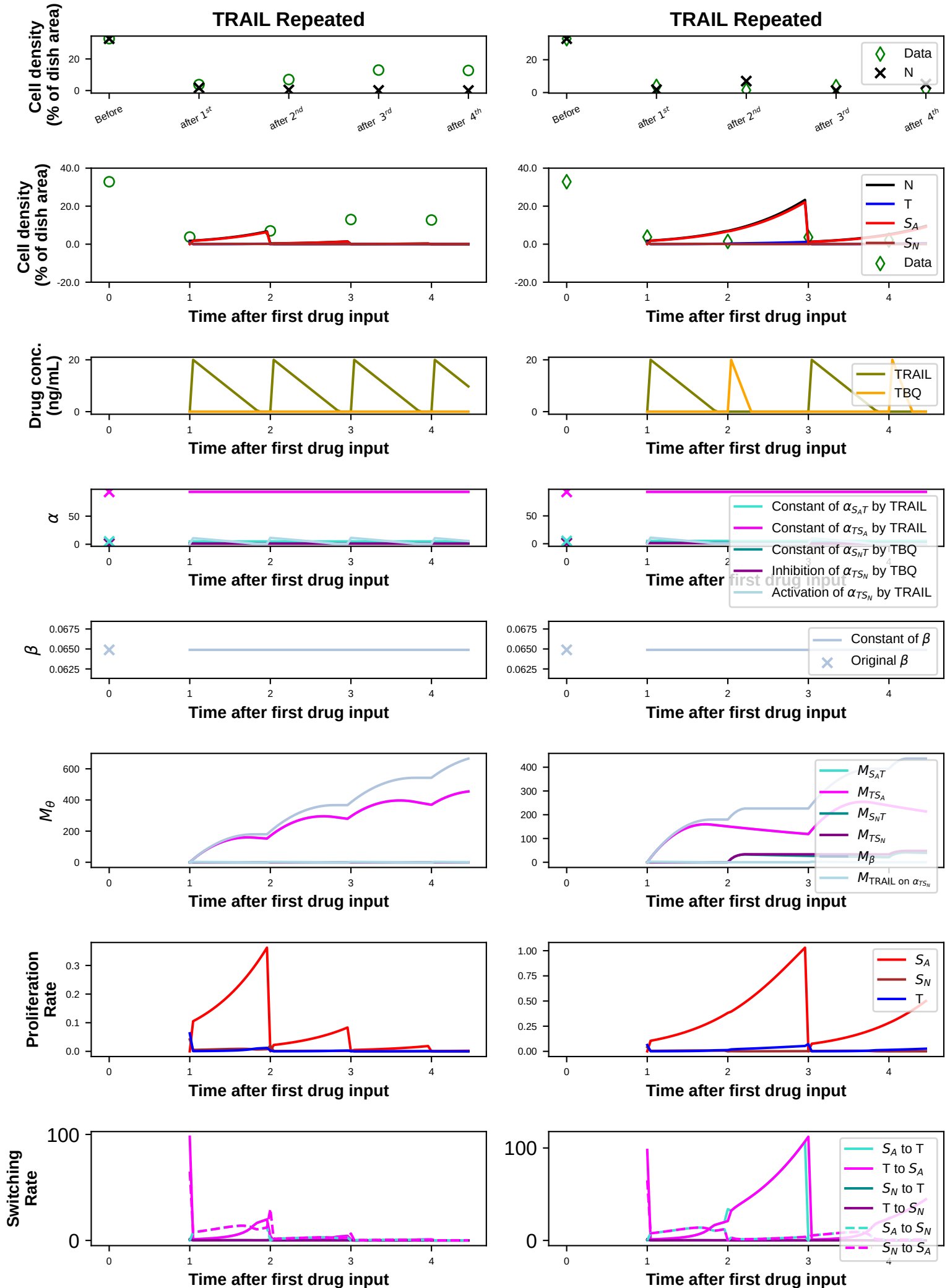

Supplement: Supplementary file 7 — Appendix Simulations Results [file 44320_2025_150_MOESM7_ESM.zip › Appendix_Simulations_Results/PSM2D_Simulations/PSM2_A_8_N_3.pdf]

TRAIL/TBQ phenotypic switch Model A 2, Model N 7  
RMSE AAAA = 1.5191, RMSE ANAN = 2.8581

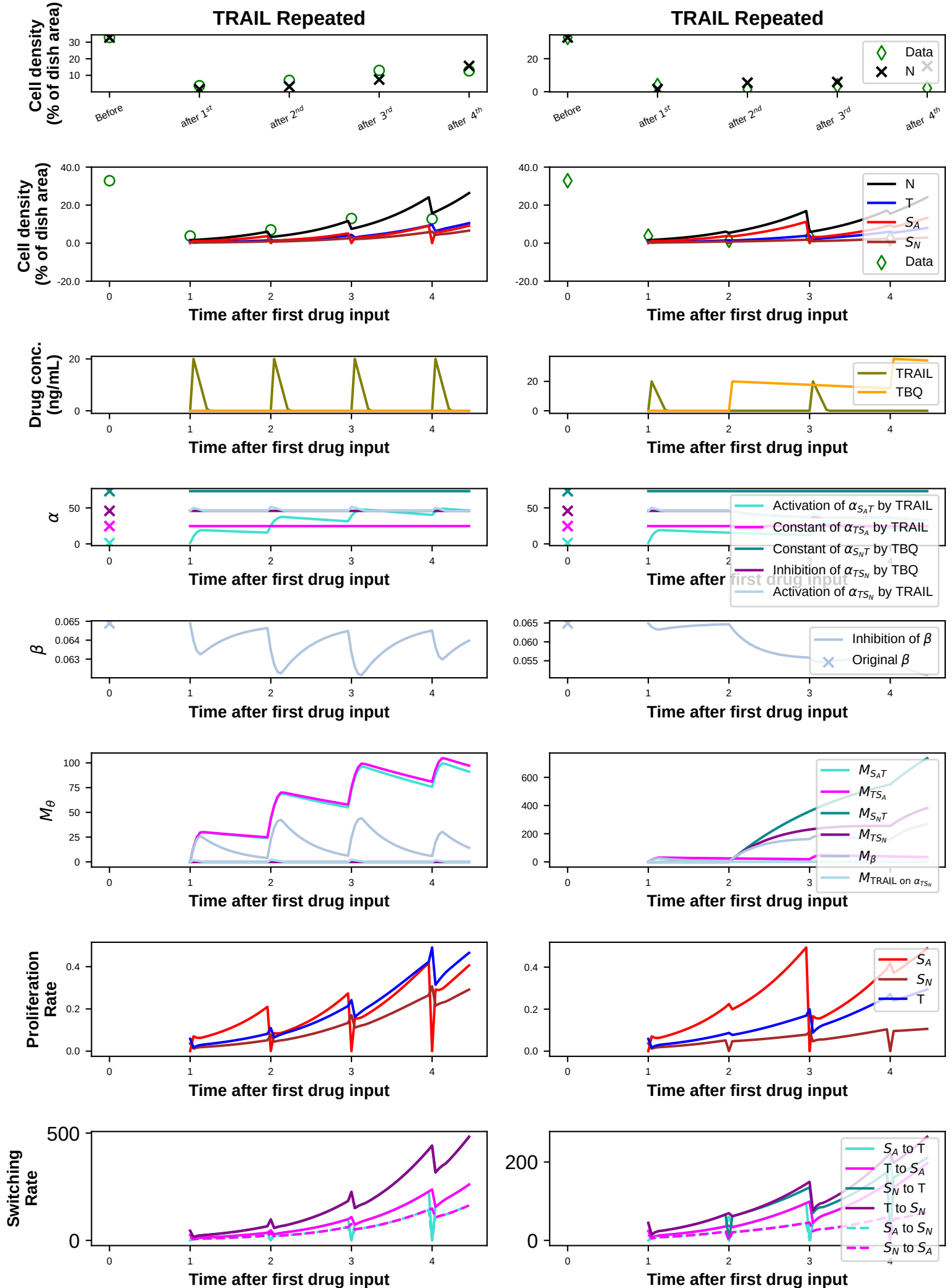

Supplement: Supplementary file 7 — Appendix Simulations Results [file 44320_2025_150_MOESM7_ESM.zip › Appendix_Simulations_Results/PSM2D_Simulations/PSM2_A_2_N_7.pdf]

# TRAIL/TBQ phenotypic switch Model A 3, Model N 2

RMSE AAAA = 1.2828, RMSE ANAN = 0.4919

## TRAIL Repeated

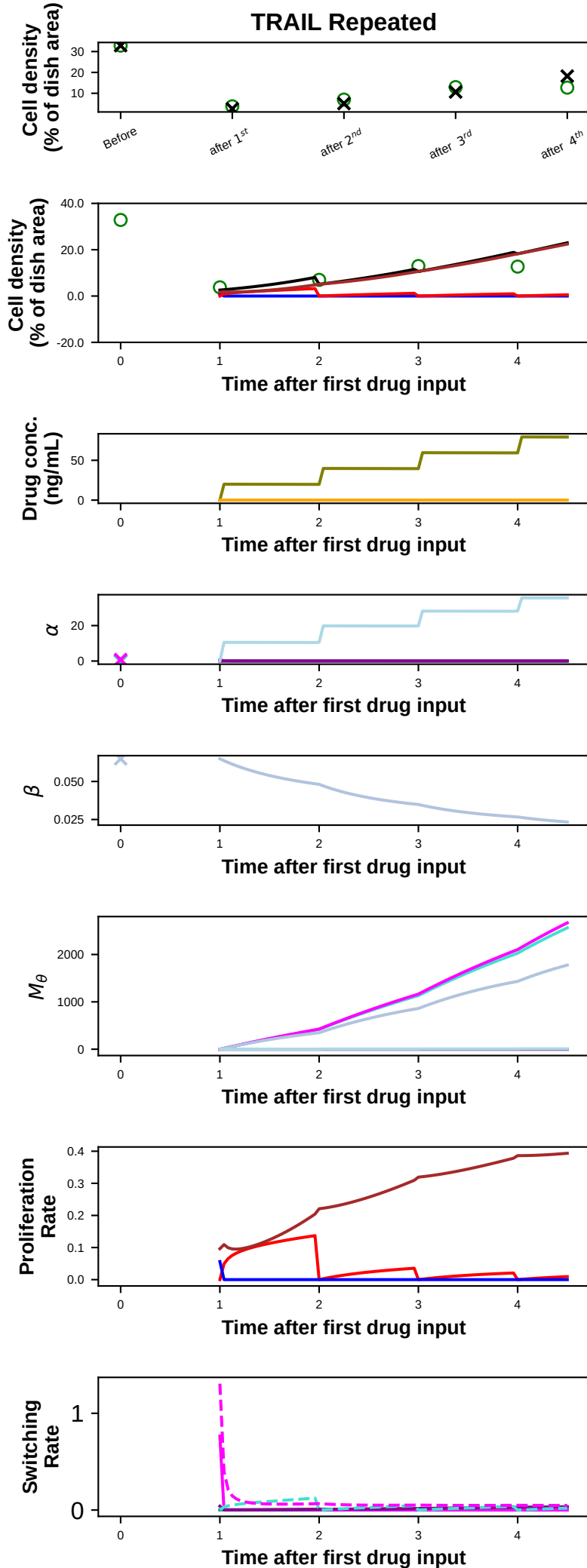

## TRAIL Repeated

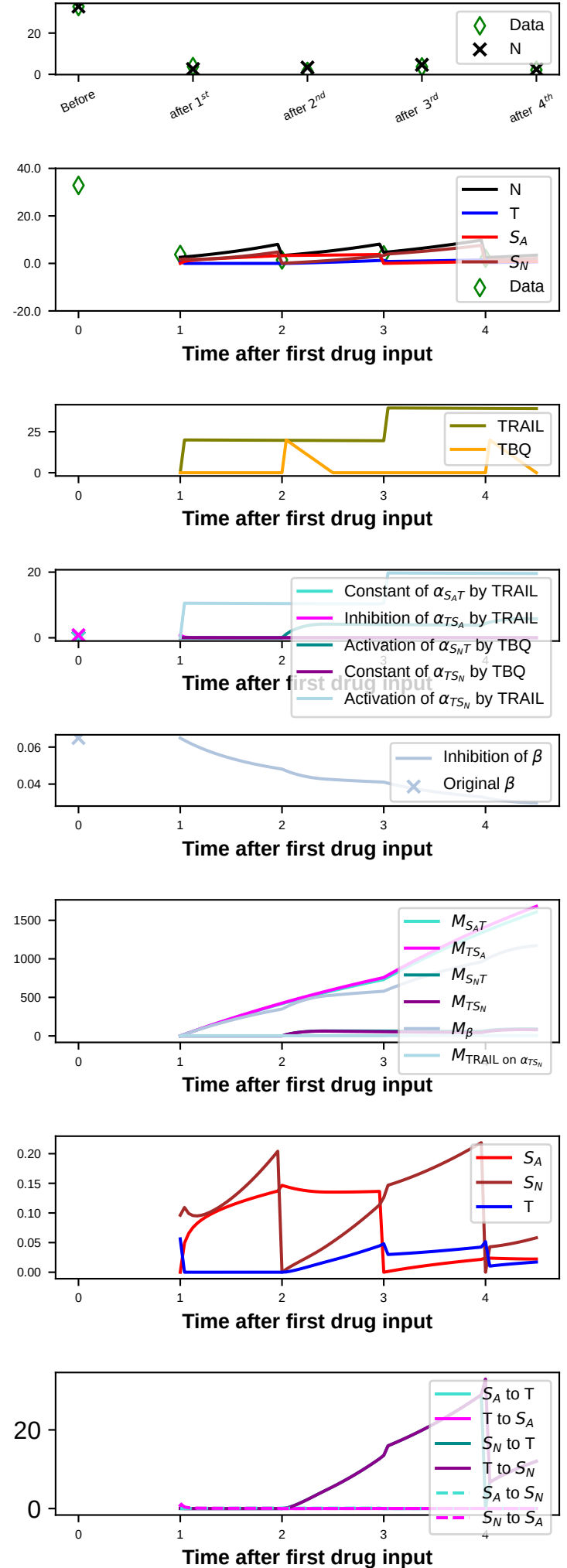

Supplement: Supplementary file 7 — Appendix Simulations Results [file 44320_2025_150_MOESM7_ESM.zip › Appendix_Simulations_Results/PSM2D_Simulations/PSM2_A_3_N_2.pdf]

TRAIL/TBQ phenotypic switch Model A 3, Model N 3  
RMSE AAAA = 1.2831, RMSE ANAN = 0.7376

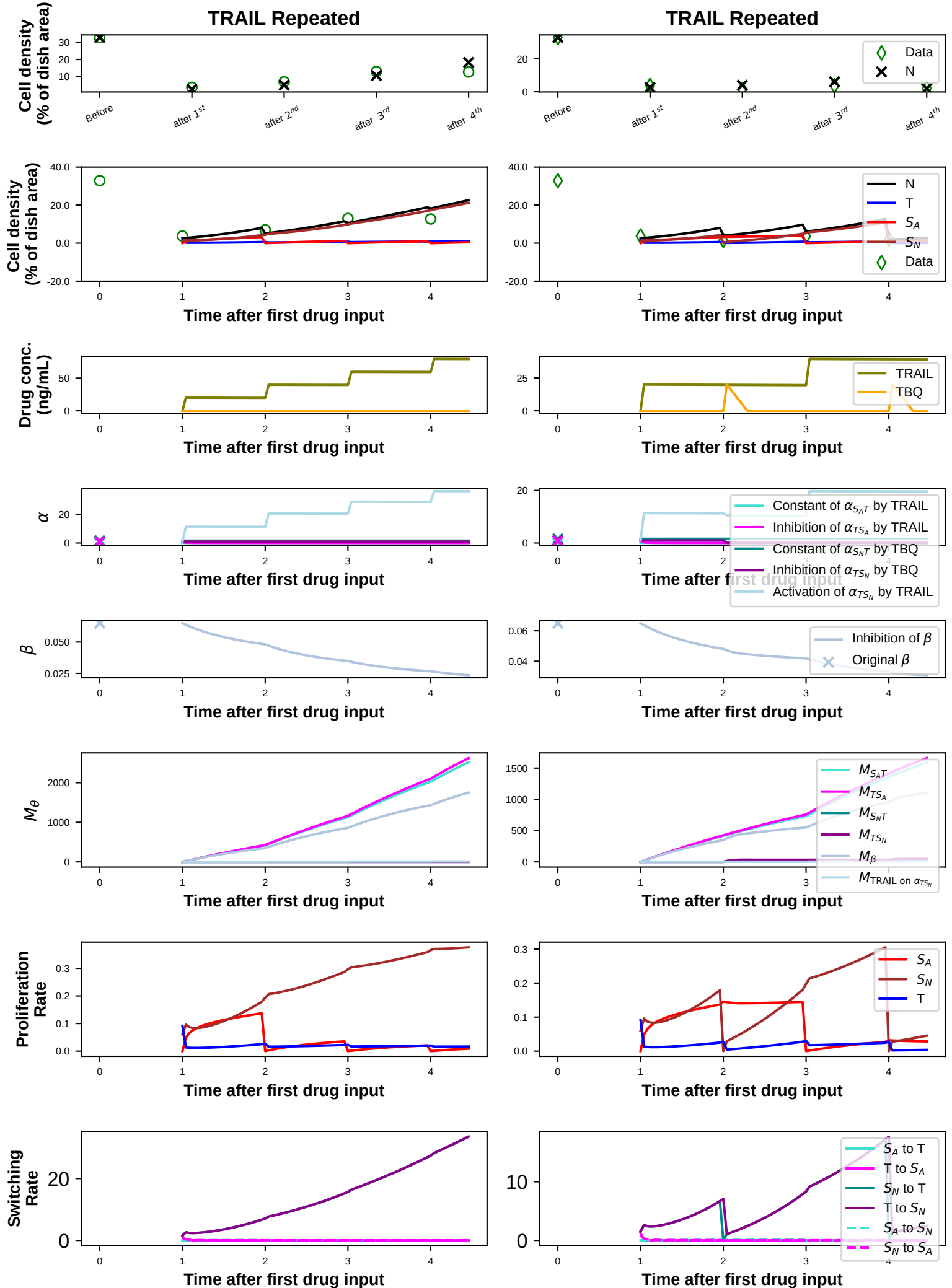

Supplement: Supplementary file 7 — Appendix Simulations Results [file 44320_2025_150_MOESM7_ESM.zip › Appendix_Simulations_Results/PSM2D_Simulations/PSM2_A_3_N_3.pdf]

TRAIL/TBQ phenotypic switch Model A 2, Model N 6  
RMSE AAAA = 1.5191, RMSE ANAN = 1.8247

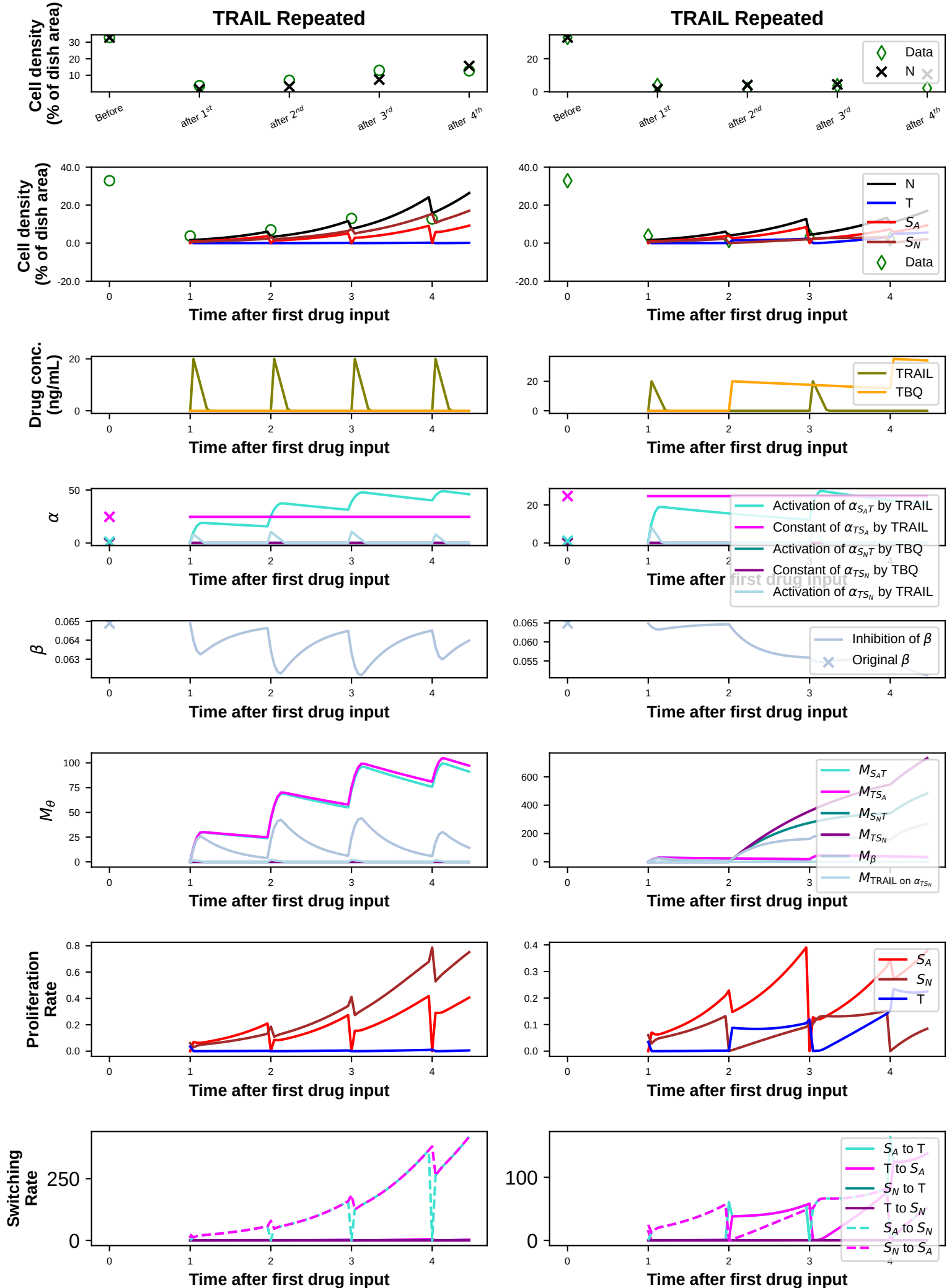

Supplement: Supplementary file 7 — Appendix Simulations Results [file 44320_2025_150_MOESM7_ESM.zip › Appendix_Simulations_Results/PSM2D_Simulations/PSM2_A_2_N_6.pdf]

TRAIL/TBQ phenotypic switch Model A 2, Model N 4  
RMSE AAAA = 1.5196, RMSE ANAN = 2.0807

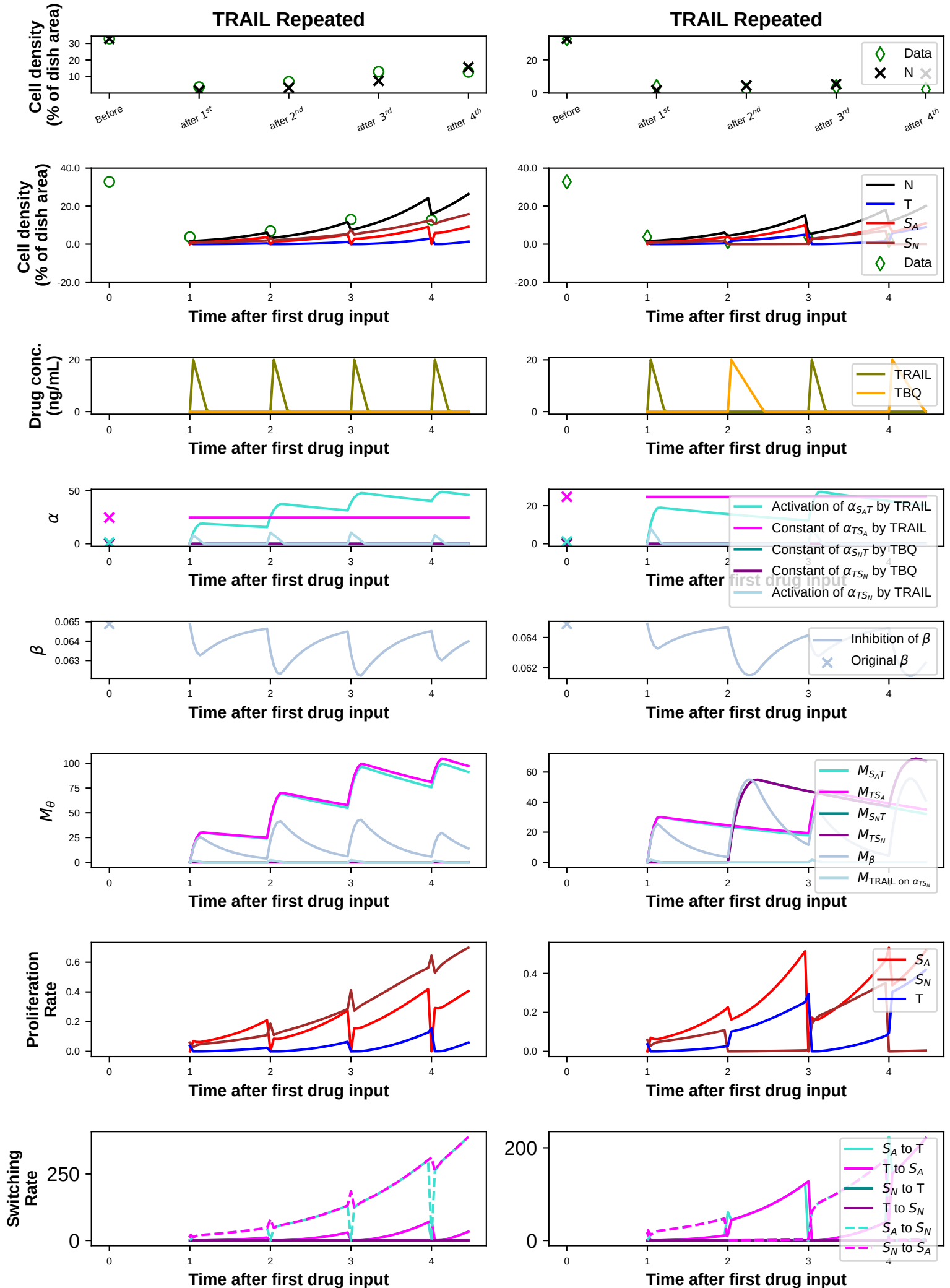

Supplement: Supplementary file 7 — Appendix Simulations Results [file 44320_2025_150_MOESM7_ESM.zip › Appendix_Simulations_Results/PSM2D_Simulations/PSM2_A_2_N_4.pdf]

TRAIL/TBQ phenotypic switch Model A 3, Model N 1  
RMSE AAAA = 1.283, RMSE ANAN = 1.4218

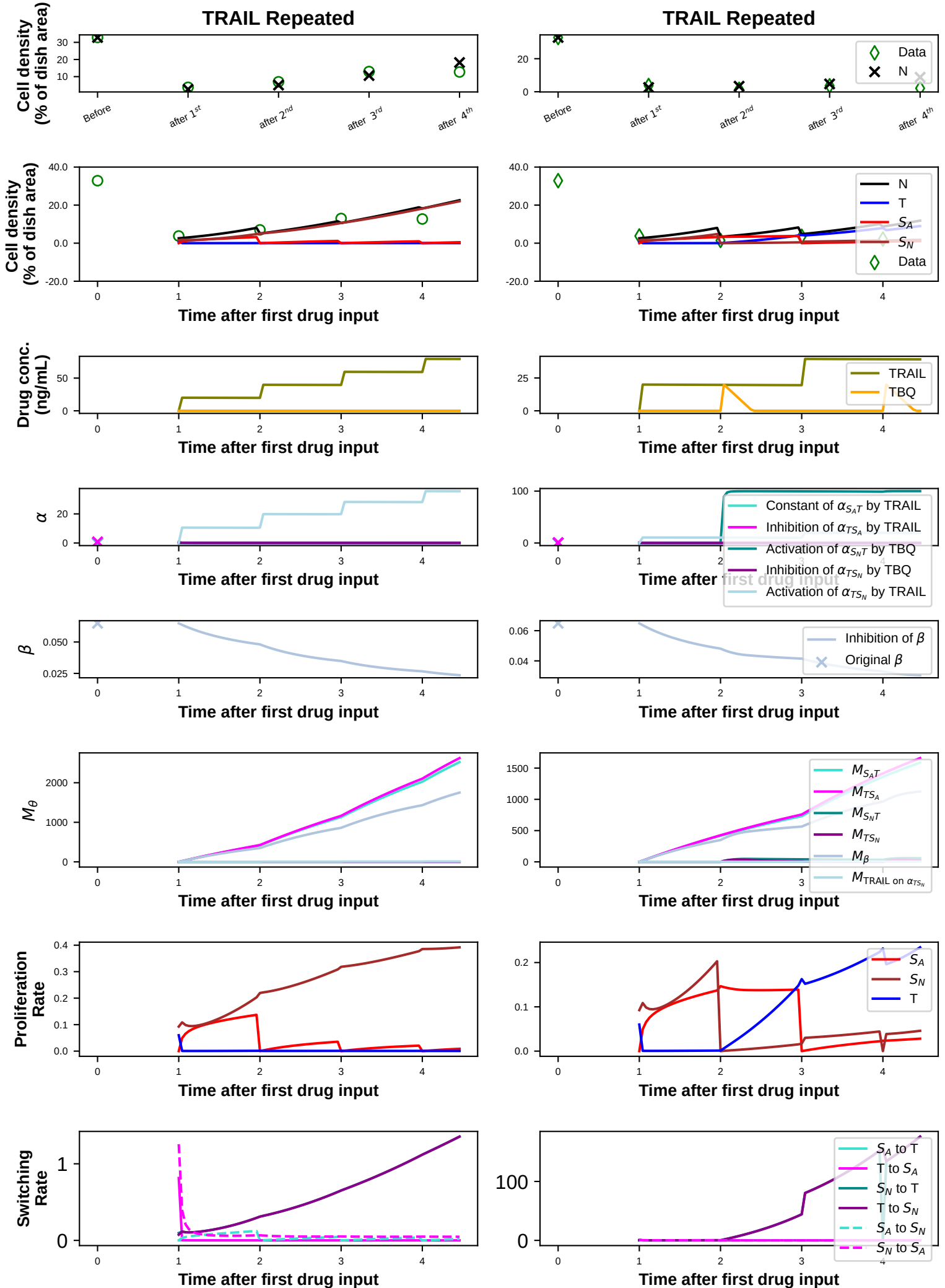

Supplement: Supplementary file 7 — Appendix Simulations Results [file 44320_2025_150_MOESM7_ESM.zip › Appendix_Simulations_Results/PSM2D_Simulations/PSM2_A_3_N_1.pdf]

TRAIL/TBQ phenotypic switch Model A 7, Model N 8  
RMSE AAAA = 2.7205, RMSE ANAN = 4.8206

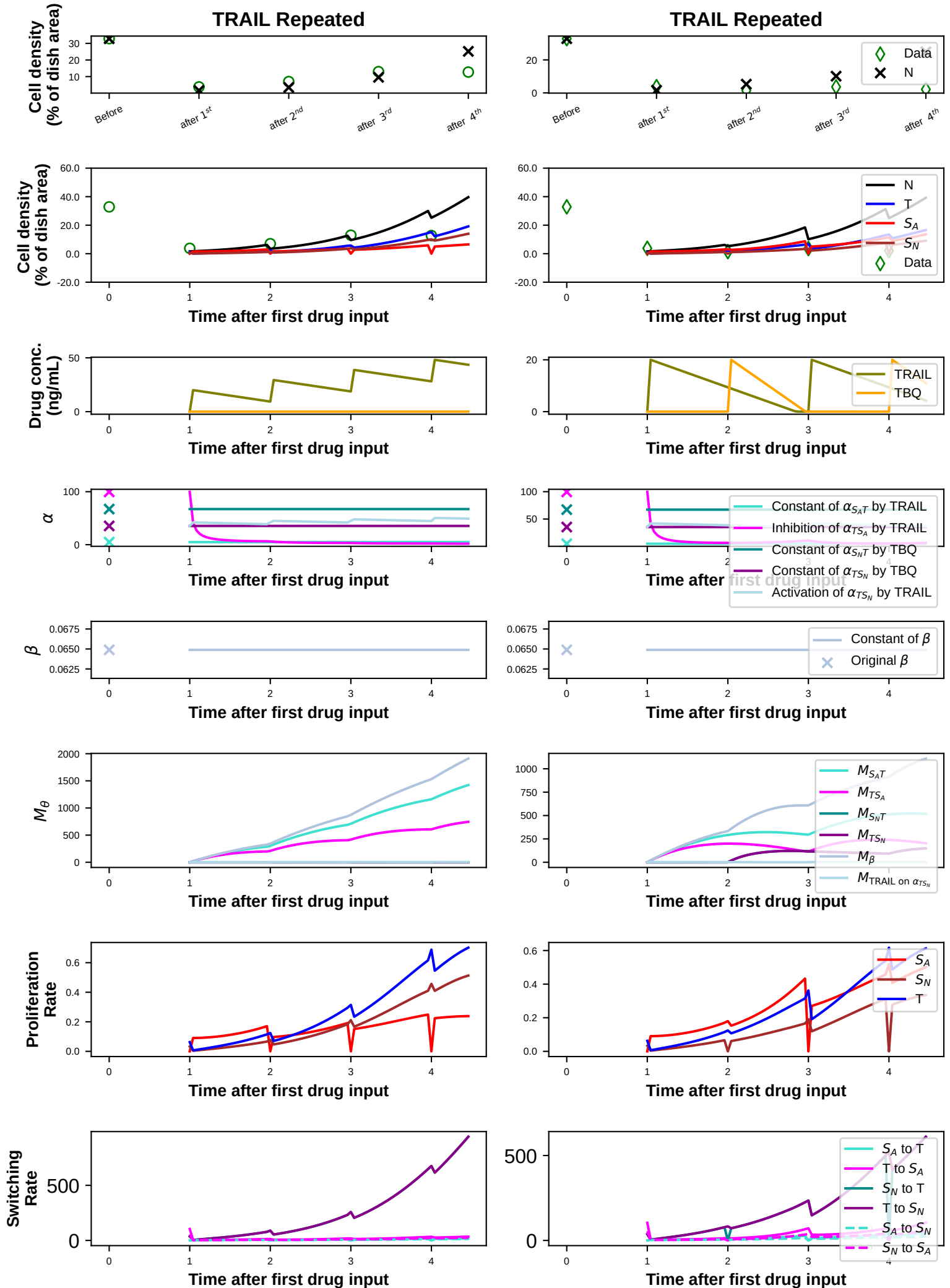

Supplement: Supplementary file 7 — Appendix Simulations Results [file 44320_2025_150_MOESM7_ESM.zip › Appendix_Simulations_Results/PSM2D_Simulations/PSM2_A_7_N_8.pdf]

TRAIL/TBQ phenotypic switch Model A 2, Model N 5  
RMSE AAAA = 1.5191, RMSE ANAN = 1.9253

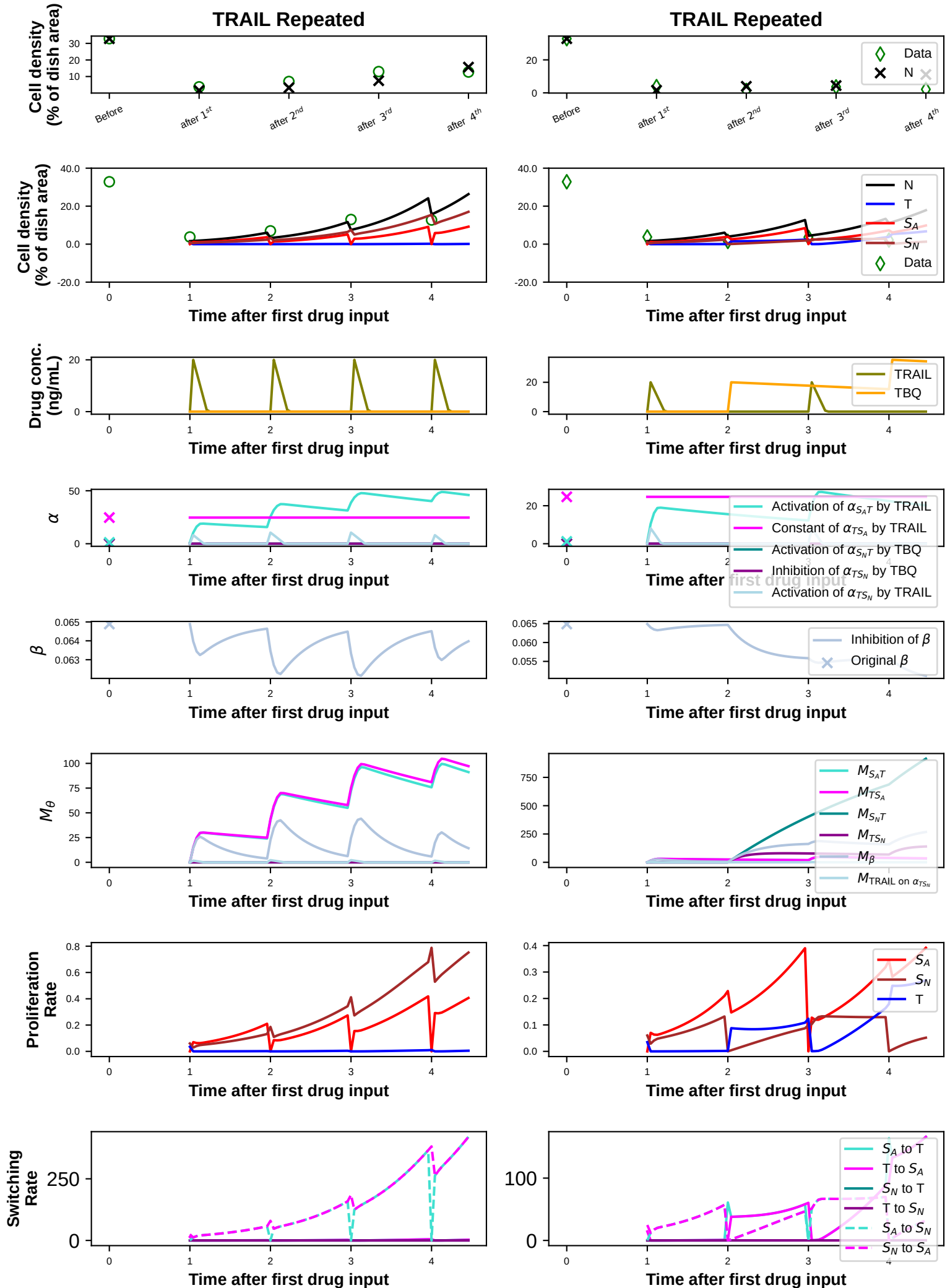

Supplement: Supplementary file 7 — Appendix Simulations Results [file 44320_2025_150_MOESM7_ESM.zip › Appendix_Simulations_Results/PSM2D_Simulations/PSM2_A_2_N_5.pdf]

# TRAIL/TBQ phenotypic switch Model A 2, Model N 1

RMSE AAAA = 1.5196, RMSE ANAN = 3.8874

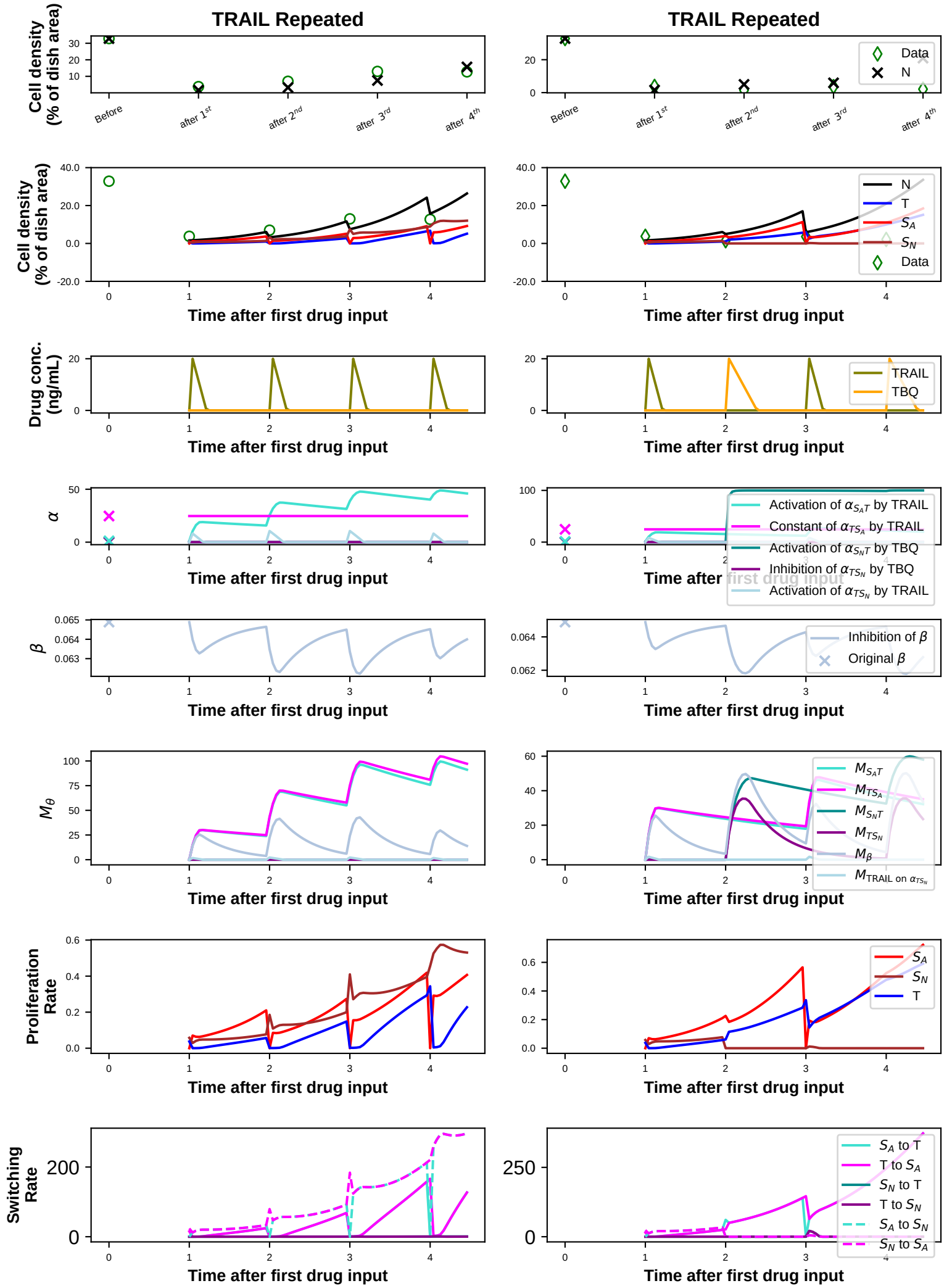

Supplement: Supplementary file 7 — Appendix Simulations Results [file 44320_2025_150_MOESM7_ESM.zip › Appendix_Simulations_Results/PSM2D_Simulations/PSM2_A_2_N_1.pdf]

TRAIL/TBQ phenotypic switch Model A 3, Model N 4  
RMSE AAAA = 1.2829, RMSE ANAN = 0.5565

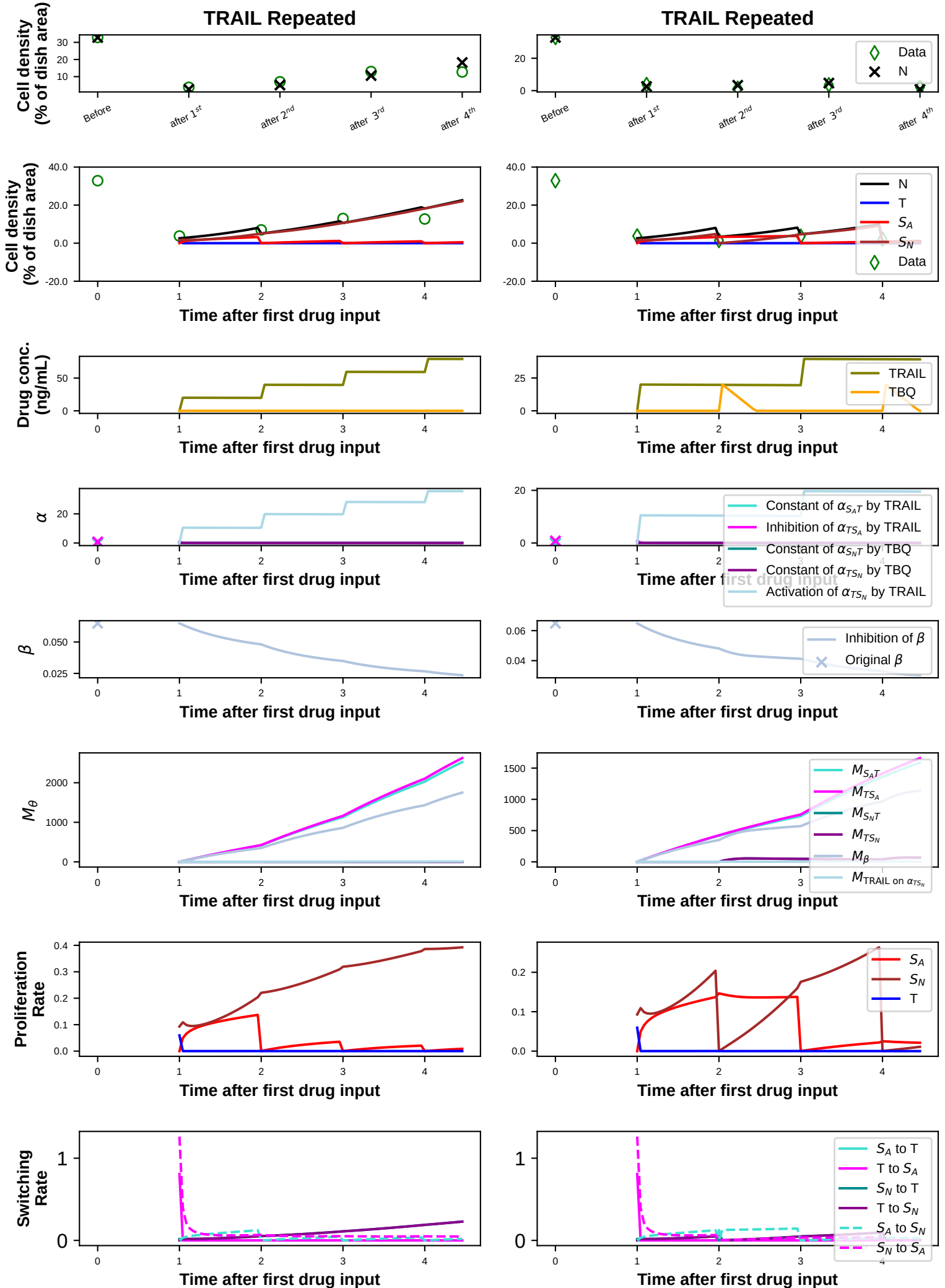

Supplement: Supplementary file 7 — Appendix Simulations Results [file 44320_2025_150_MOESM7_ESM.zip › Appendix_Simulations_Results/PSM2D_Simulations/PSM2_A_3_N_4.pdf]

TRAIL/TBQ phenotypic switch Model A 6, Model N 8  
RMSE AAAA = 2.8189, RMSE ANAN = 6.3592

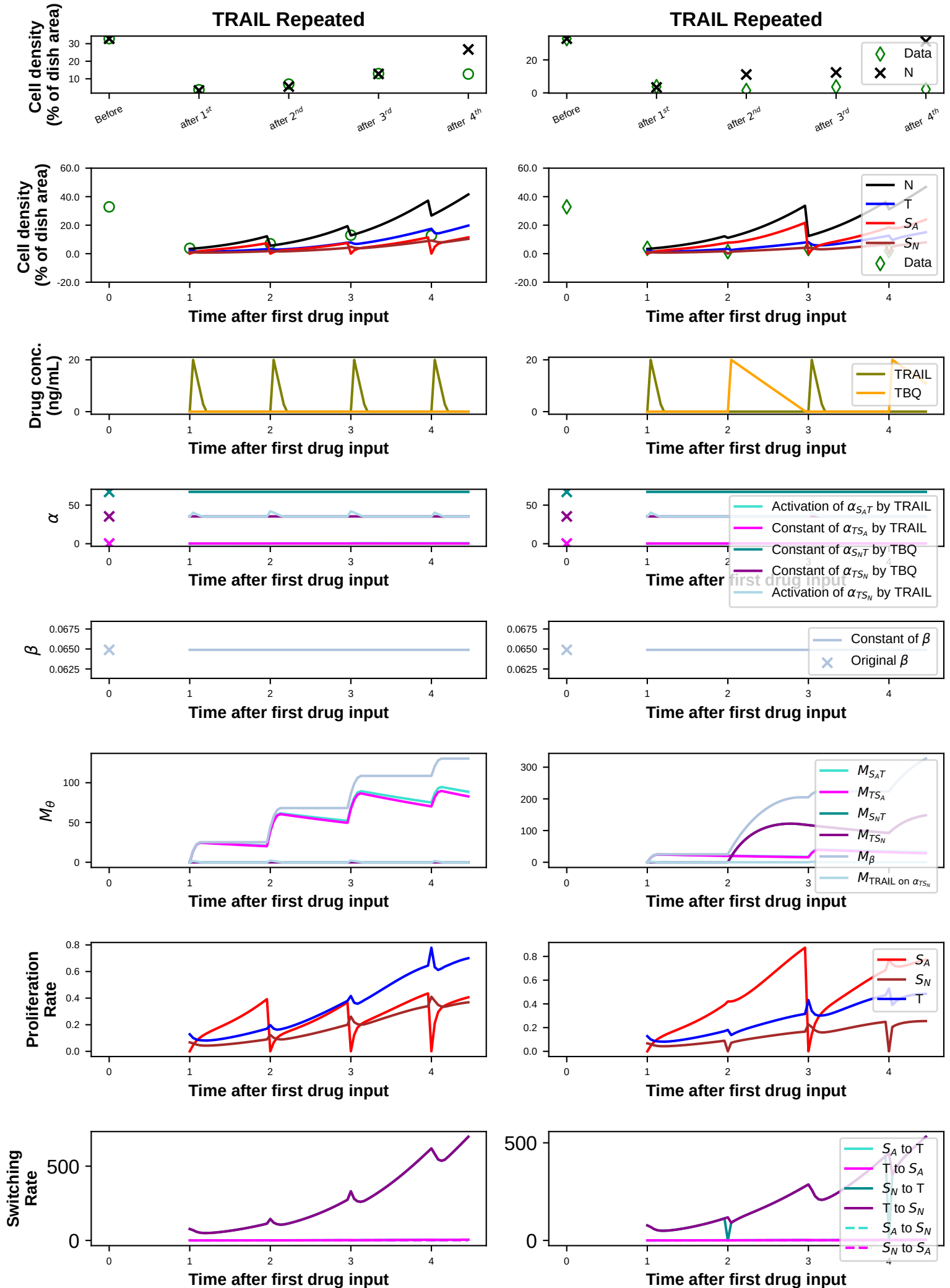

Supplement: Supplementary file 7 — Appendix Simulations Results [file 44320_2025_150_MOESM7_ESM.zip › Appendix_Simulations_Results/PSM2D_Simulations/PSM2_A_6_N_8.pdf]

TRAIL/TBQ phenotypic switch Model A 3, Model N 5  
RMSE AAAA = 1.276, RMSE ANAN = 0.5476

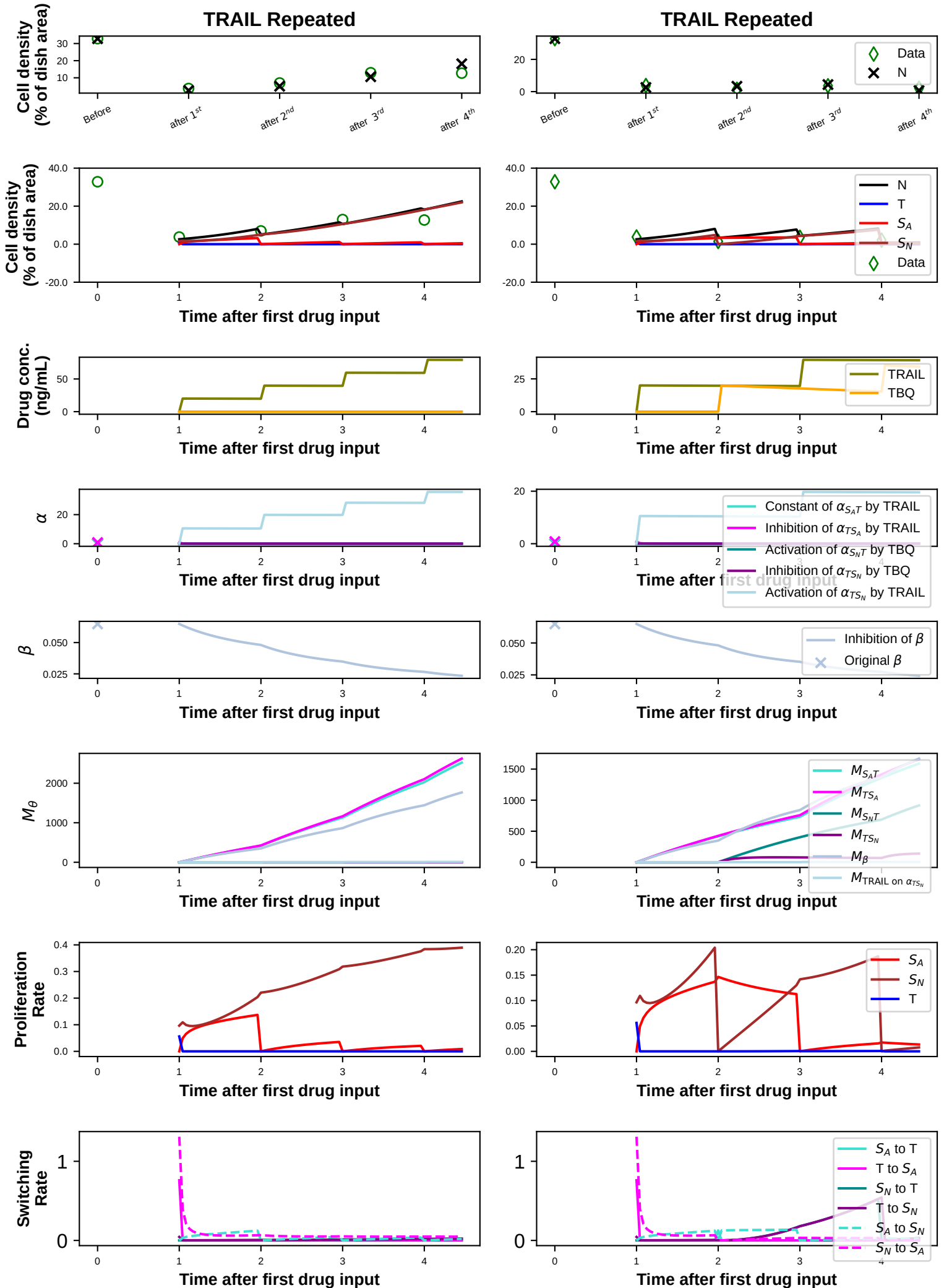

Supplement: Supplementary file 7 — Appendix Simulations Results [file 44320_2025_150_MOESM7_ESM.zip › Appendix_Simulations_Results/PSM2D_Simulations/PSM2_A_3_N_5.pdf]

# TRAIL/TBQ phenotypic switch Model A 2, Model N 2

RMSE AAAA = 1.5196, RMSE ANAN = 3.145

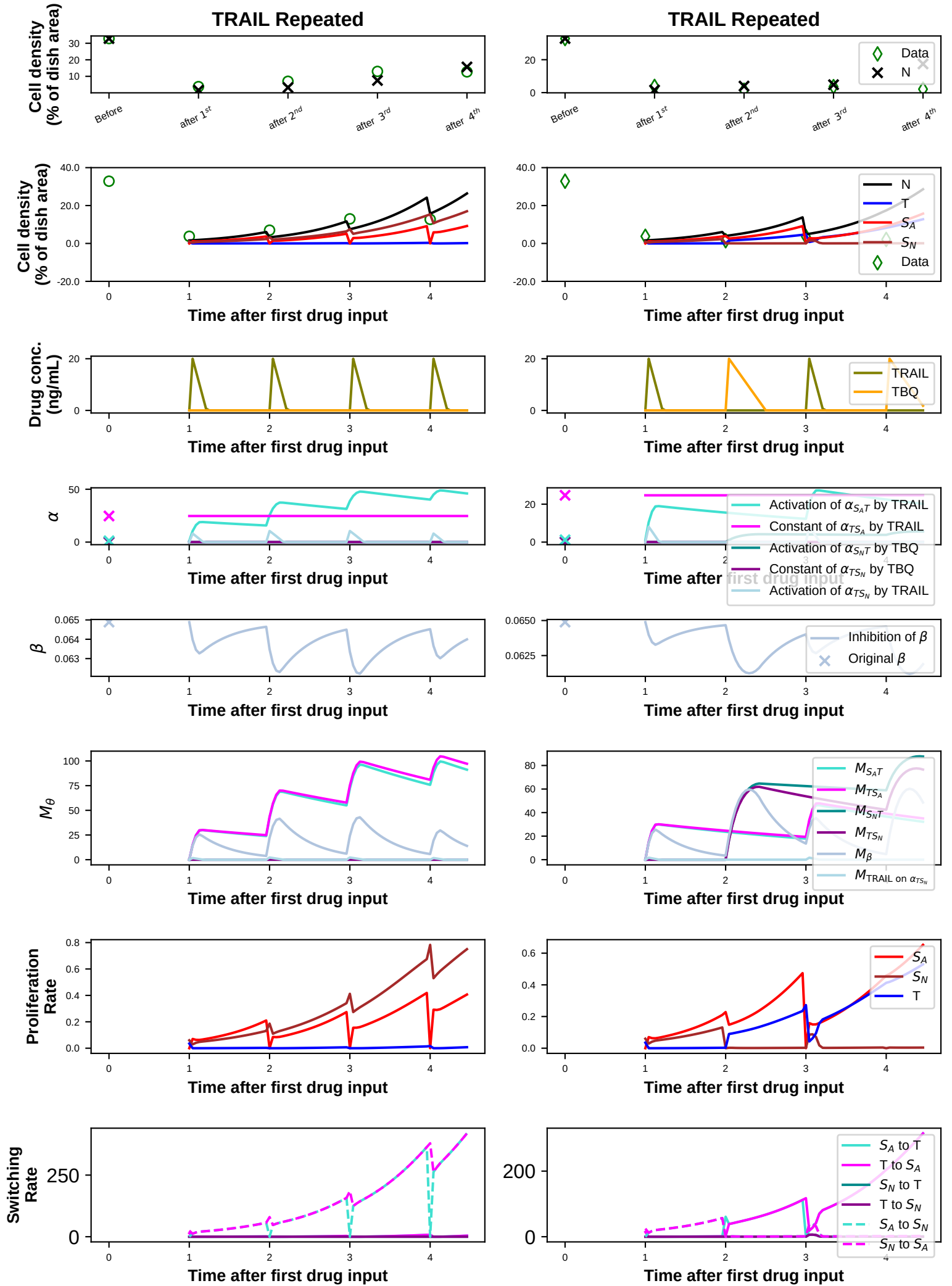

Supplement: Supplementary file 7 — Appendix Simulations Results [file 44320_2025_150_MOESM7_ESM.zip › Appendix_Simulations_Results/PSM2D_Simulations/PSM2_A_2_N_2.pdf]

TRAIL/TBQ phenotypic switch Model A 3, Model N 7  
RMSE AAAA = 1.276, RMSE ANAN = 2.6014

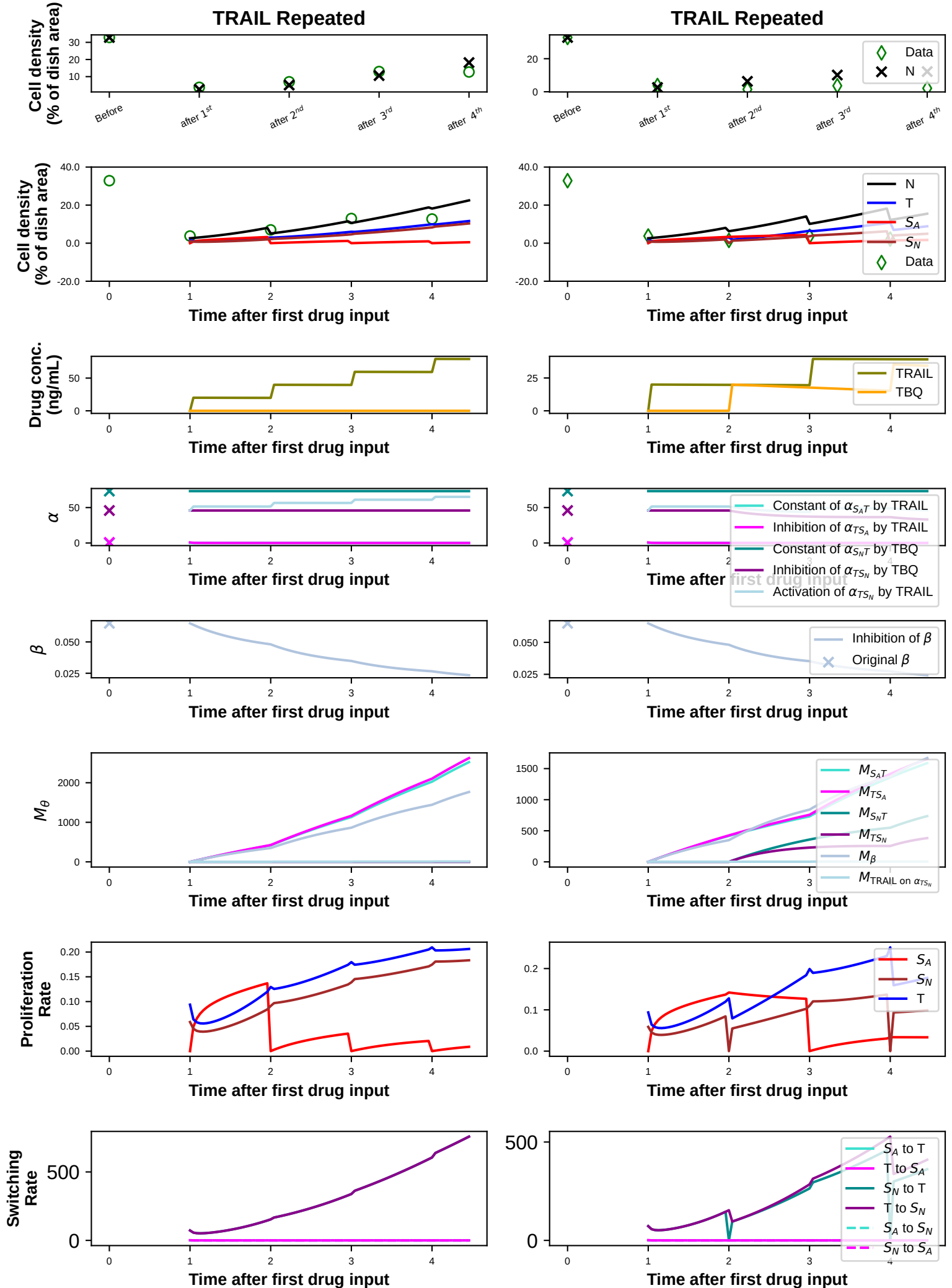

Supplement: Supplementary file 7 — Appendix Simulations Results [file 44320_2025_150_MOESM7_ESM.zip › Appendix_Simulations_Results/PSM2D_Simulations/PSM2_A_3_N_7.pdf]

TRAIL/TBQ phenotypic switch Model A 3, Model N 6  
RMSE AAAA = 1.2761, RMSE ANAN = 0.5481

TRAIL Repeated

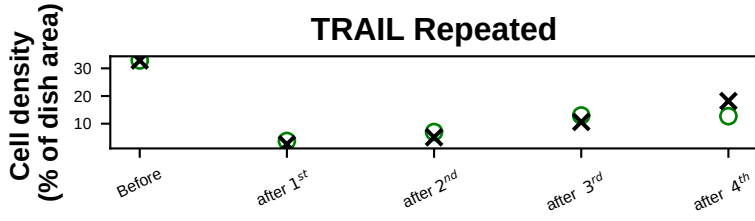

TRAIL Repeated

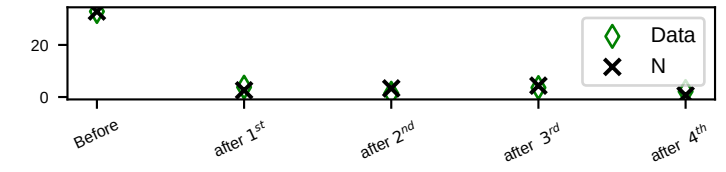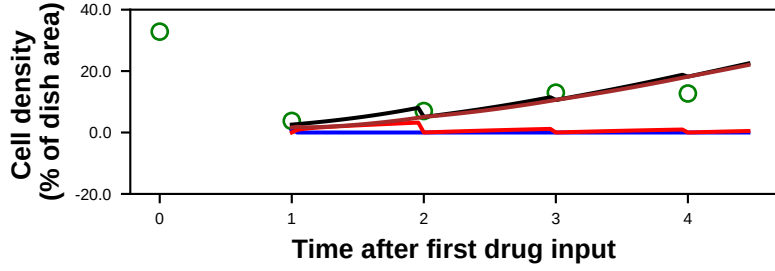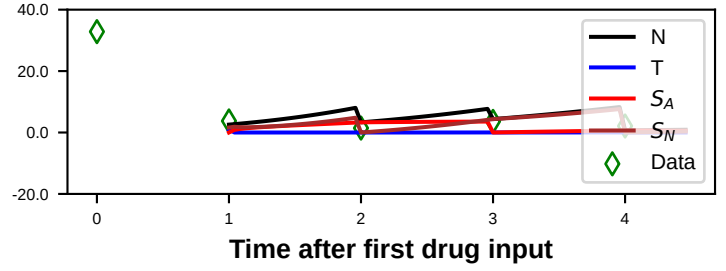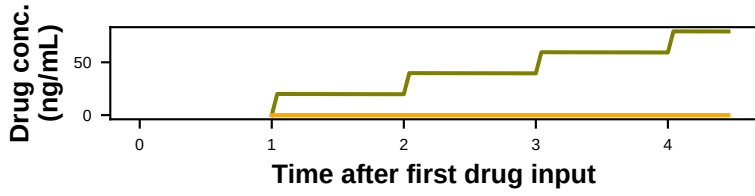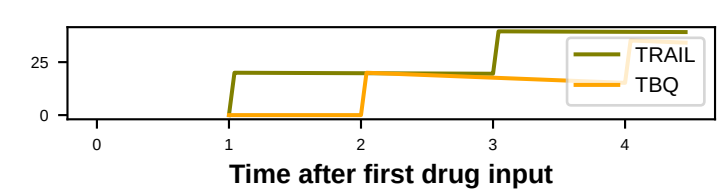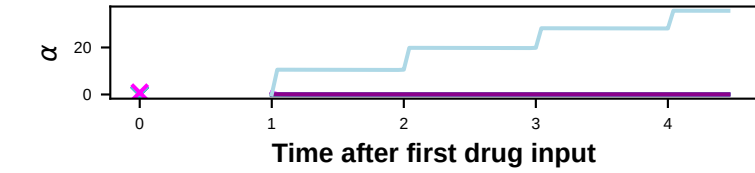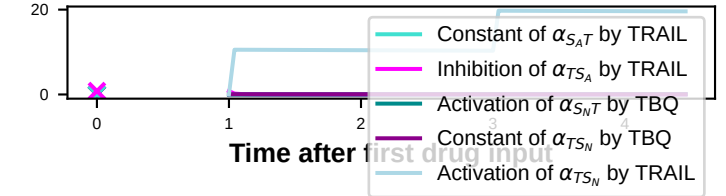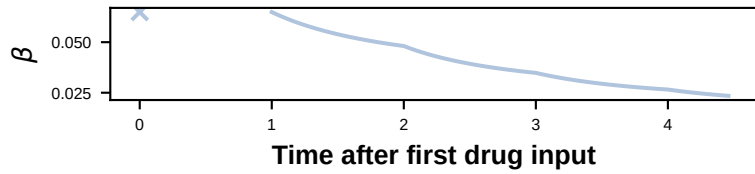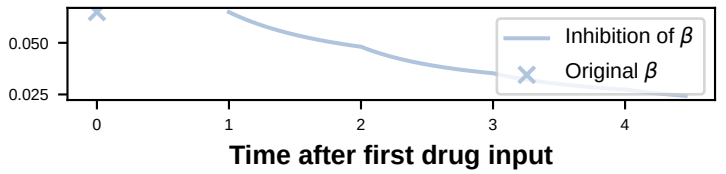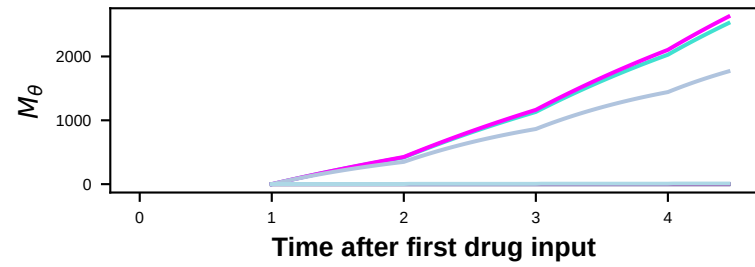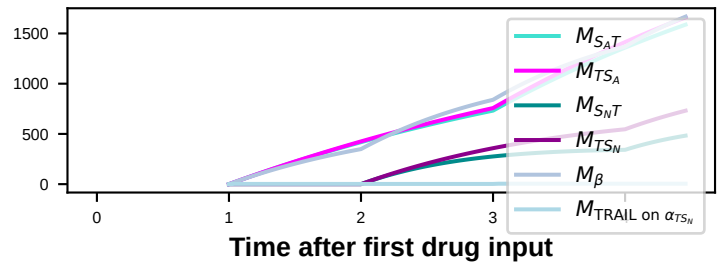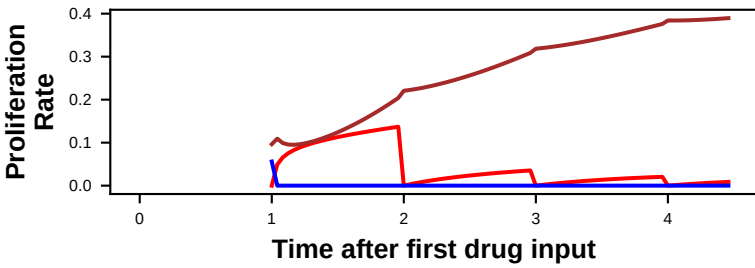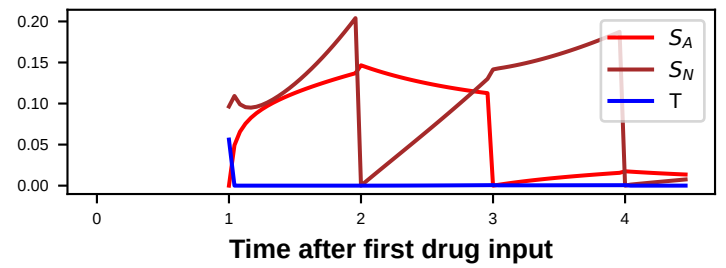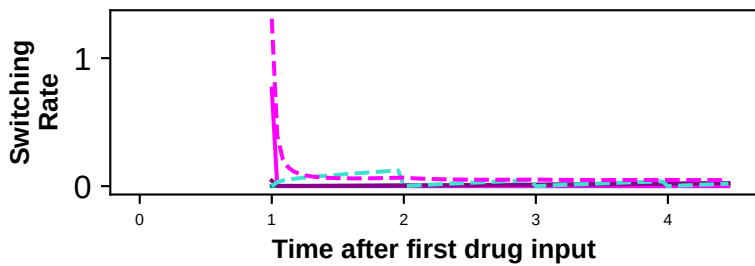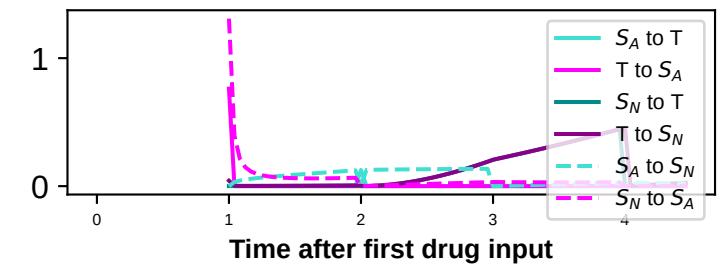

Supplement: Supplementary file 7 — Appendix Simulations Results [file 44320_2025_150_MOESM7_ESM.zip › Appendix_Simulations_Results/PSM2D_Simulations/PSM2_A_3_N_6.pdf]

# TRAIL/TBQ phenotypic switch Model A 2, Model N 3

RMSE AAAA = 1.5197, RMSE ANAN = 4.1368

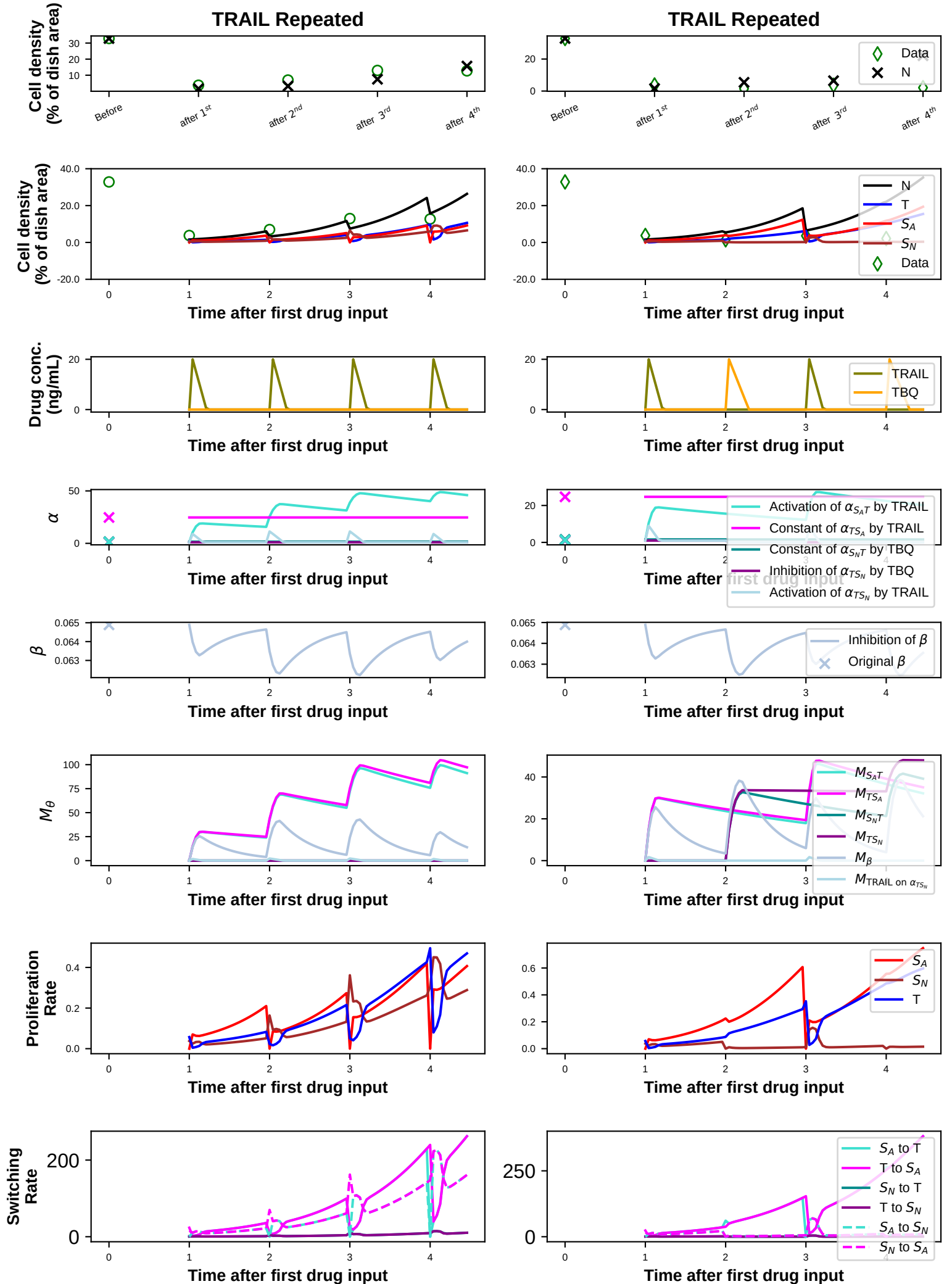

Supplement: Supplementary file 7 — Appendix Simulations Results [file 44320_2025_150_MOESM7_ESM.zip › Appendix_Simulations_Results/PSM2D_Simulations/PSM2_A_2_N_3.pdf]

# TRAIL/TBQ phenotypic switch Model A 6, Model N 6

RMSE AAAA = 2.8189, RMSE ANAN = 4.5236

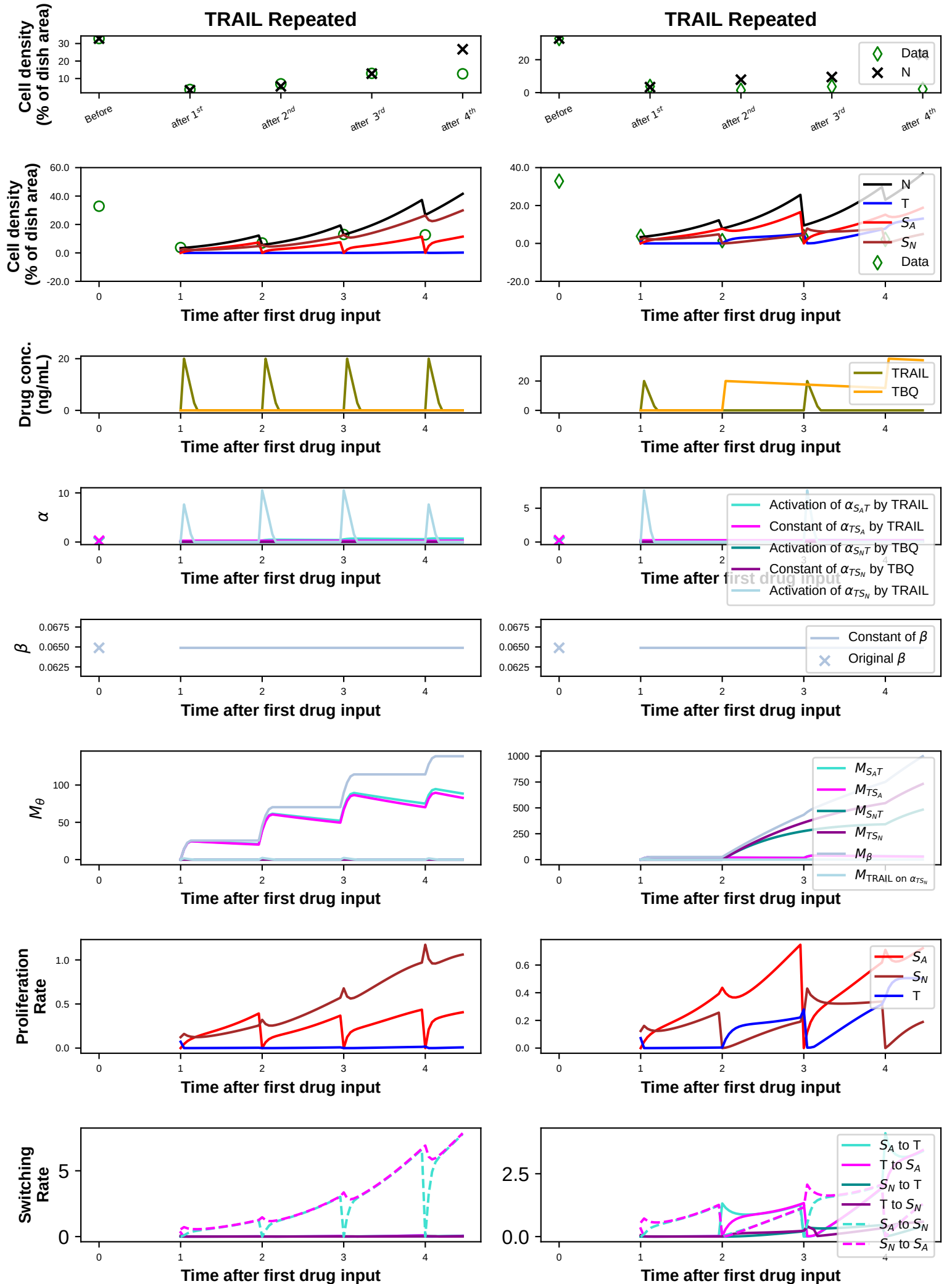

Supplement: Supplementary file 7 — Appendix Simulations Results [file 44320_2025_150_MOESM7_ESM.zip › Appendix_Simulations_Results/PSM2D_Simulations/PSM2_A_6_N_6.pdf]

TRAIL/TBQ phenotypic switch Model A 7, Model N 3  
RMSE AAAA = 2.7205, RMSE ANAN = 2.4924

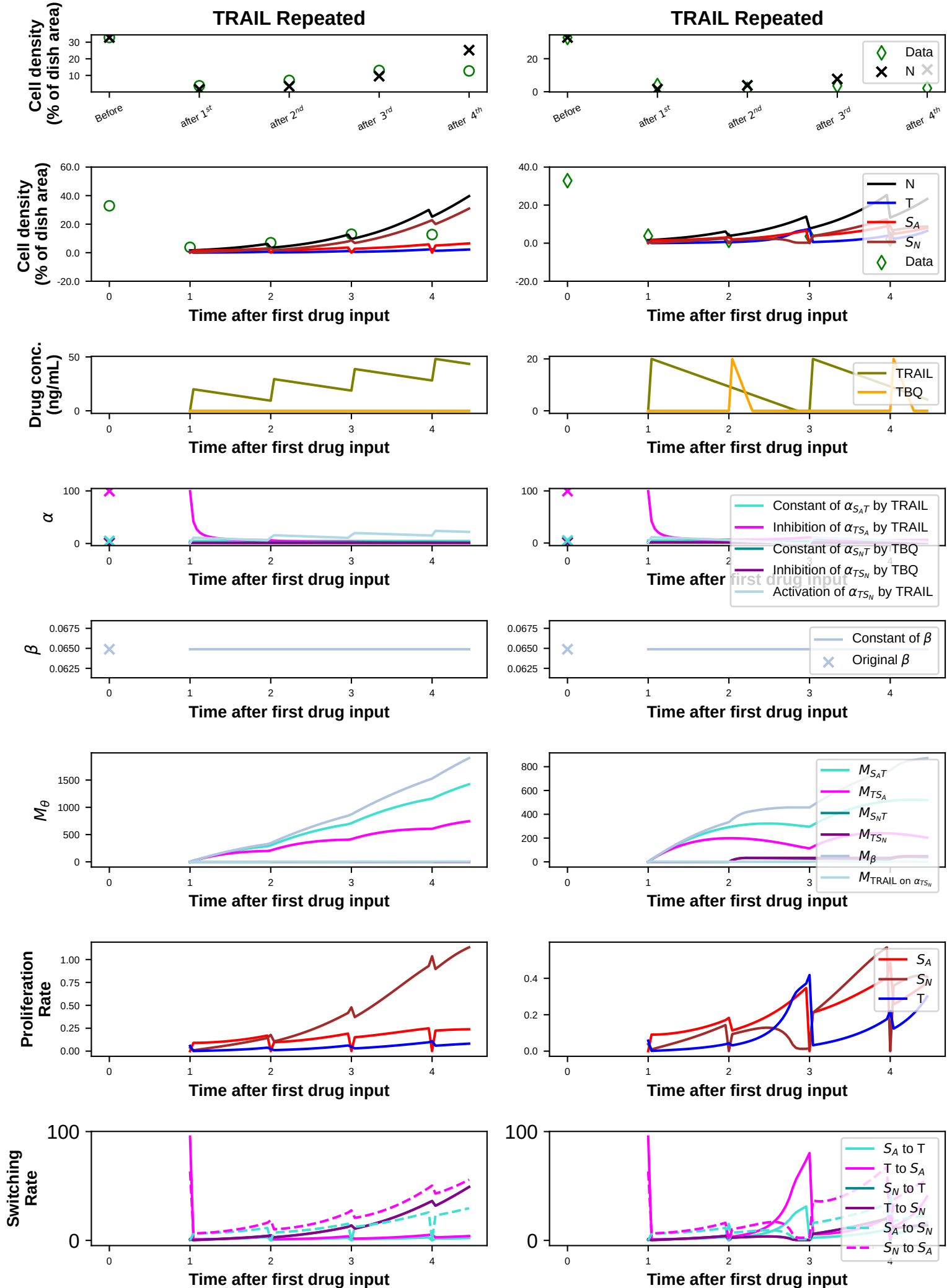

Supplement: Supplementary file 7 — Appendix Simulations Results [file 44320_2025_150_MOESM7_ESM.zip › Appendix_Simulations_Results/PSM2D_Simulations/PSM2_A_7_N_3.pdf]

TRAIL/TBQ phenotypic switch Model A 7, Model N 2  
RMSE AAAA = 2.7205, RMSE ANAN = 2.5768

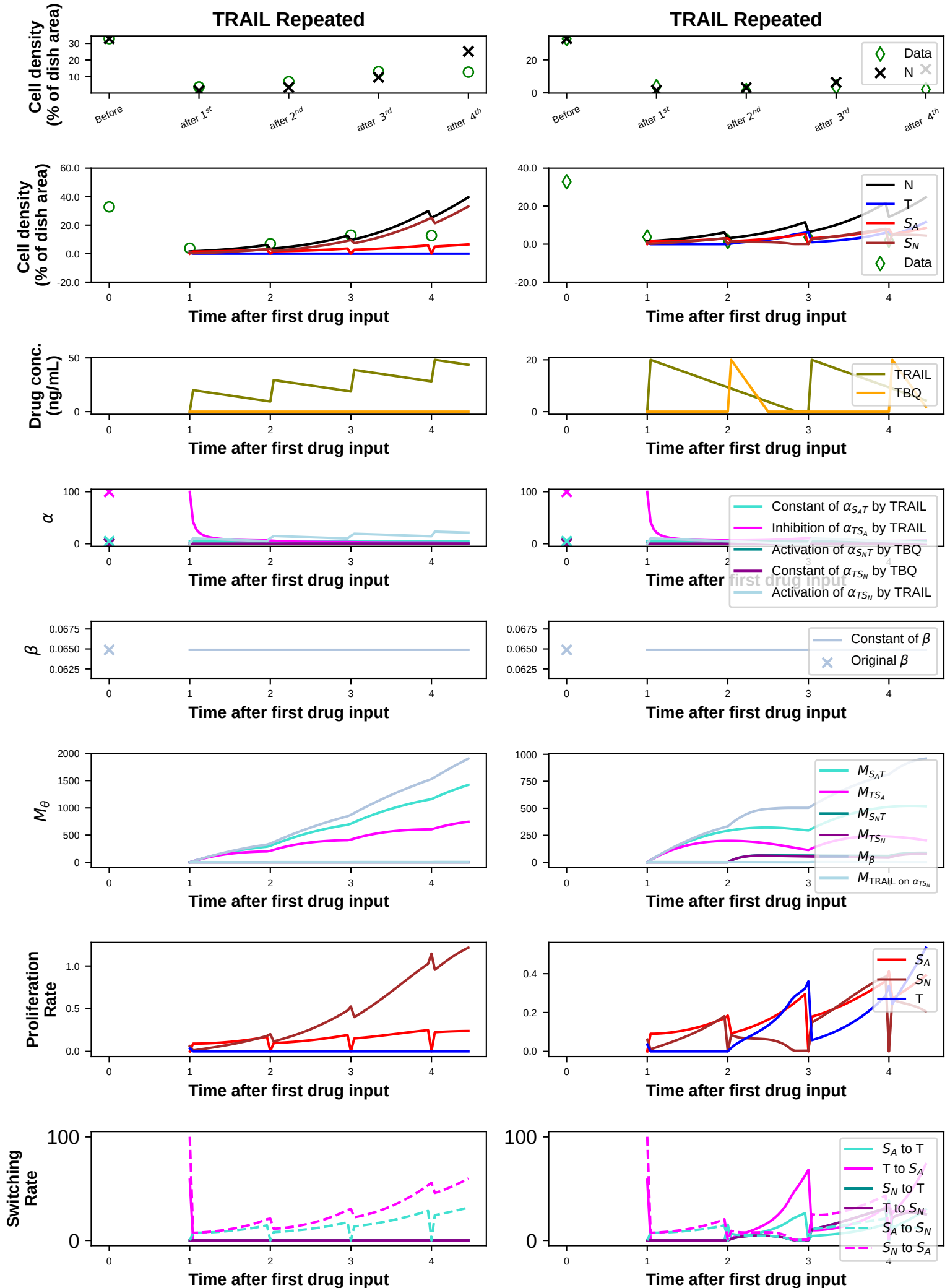

Supplement: Supplementary file 7 — Appendix Simulations Results [file 44320_2025_150_MOESM7_ESM.zip › Appendix_Simulations_Results/PSM2D_Simulations/PSM2_A_7_N_2.pdf]

TRAIL/TBQ phenotypic switch Model A 6, Model N 7  
RMSE AAAA = 2.8189, RMSE ANAN = 6.4832

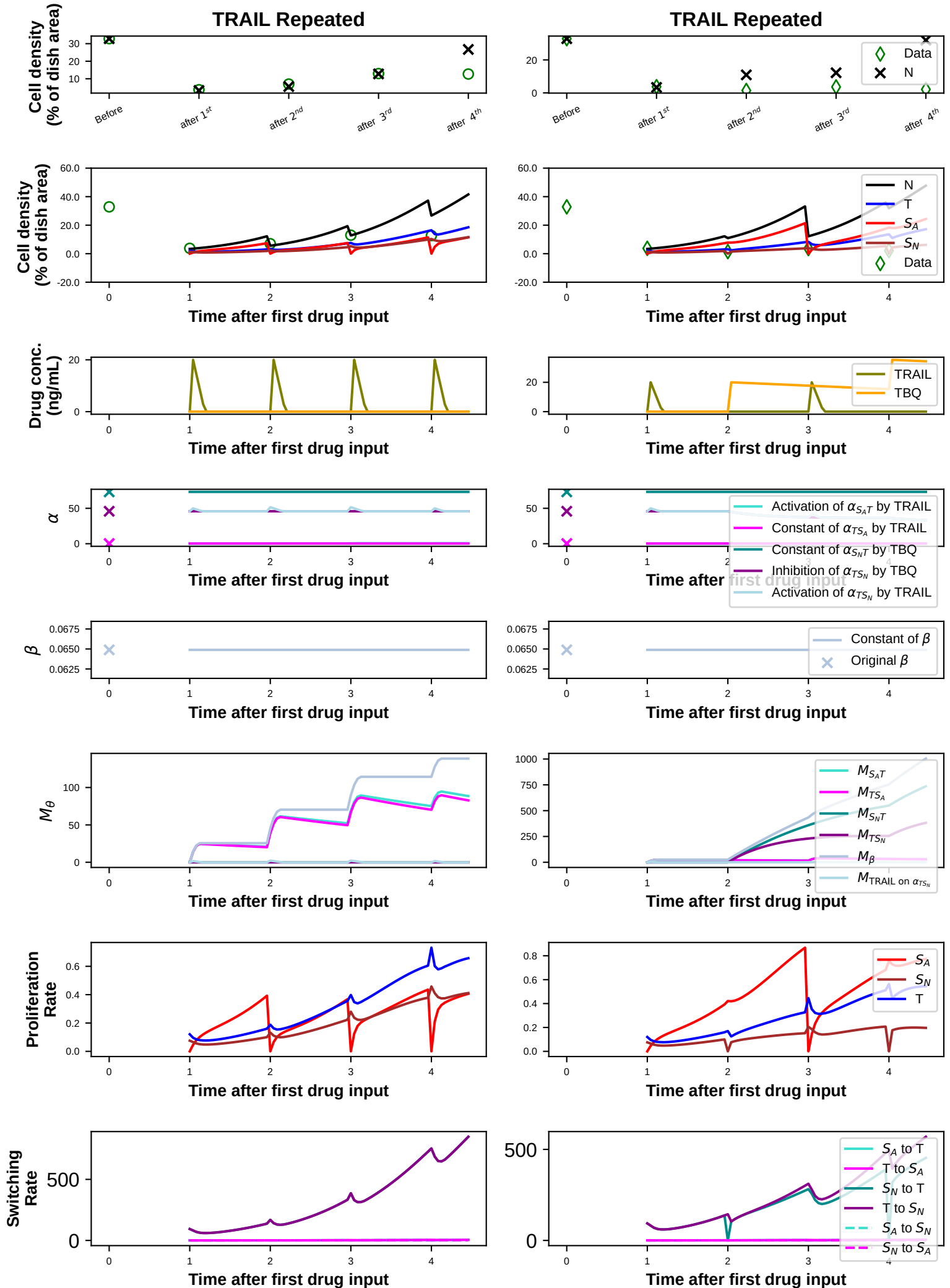

Supplement: Supplementary file 7 — Appendix Simulations Results [file 44320_2025_150_MOESM7_ESM.zip › Appendix_Simulations_Results/PSM2D_Simulations/PSM2_A_6_N_7.pdf]

TRAIL/TBQ phenotypic switch Model A 3, Model N 8  
RMSE AAAA = 1.2824, RMSE ANAN = 2.8033

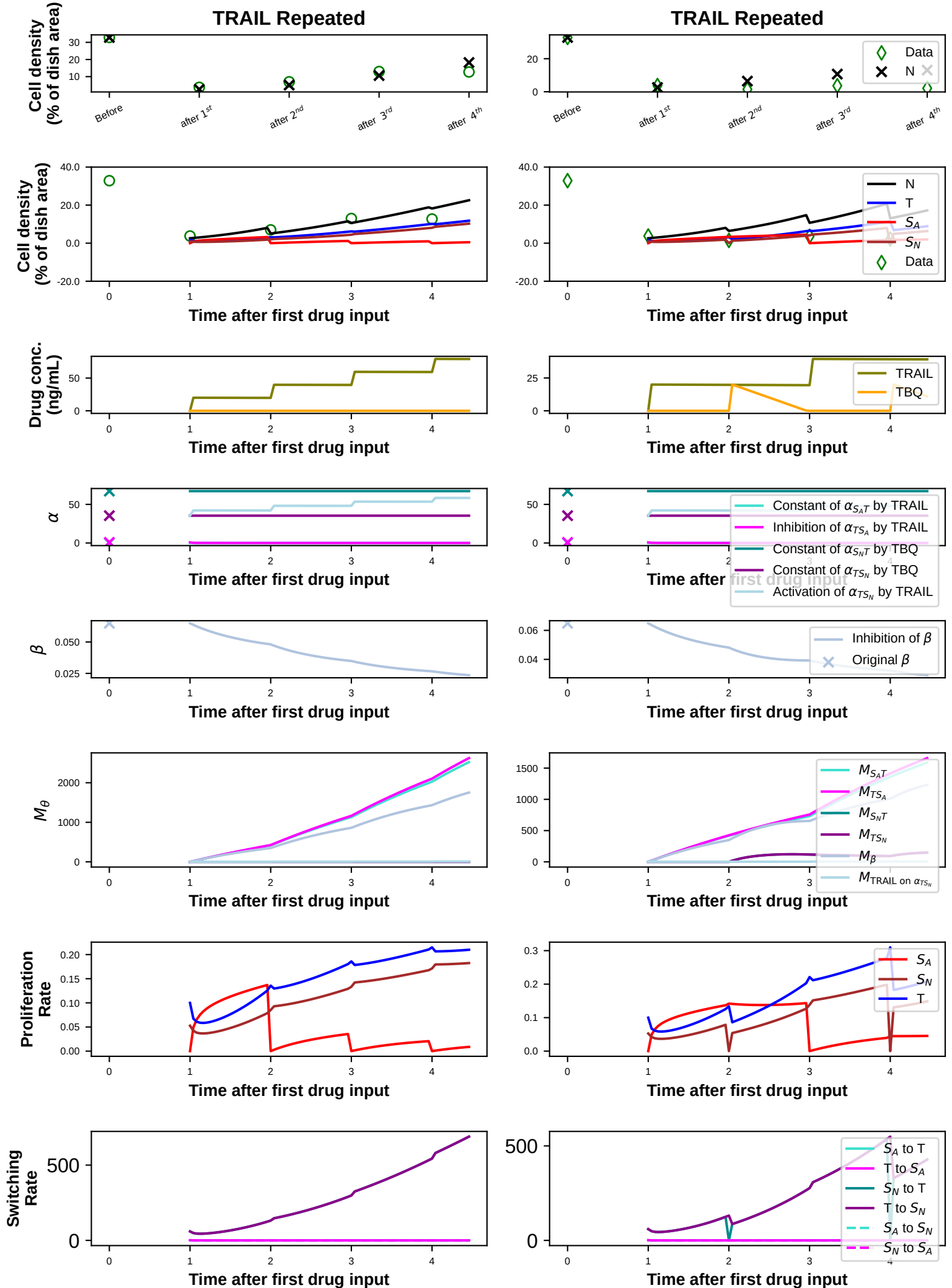

Supplement: Supplementary file 7 — Appendix Simulations Results [file 44320_2025_150_MOESM7_ESM.zip › Appendix_Simulations_Results/PSM2D_Simulations/PSM2_A_3_N_8.pdf]

TRAIL/TBQ phenotypic switch Model A 6, Model N 5  
RMSE AAAA = 2.8189, RMSE ANAN = 4.7672

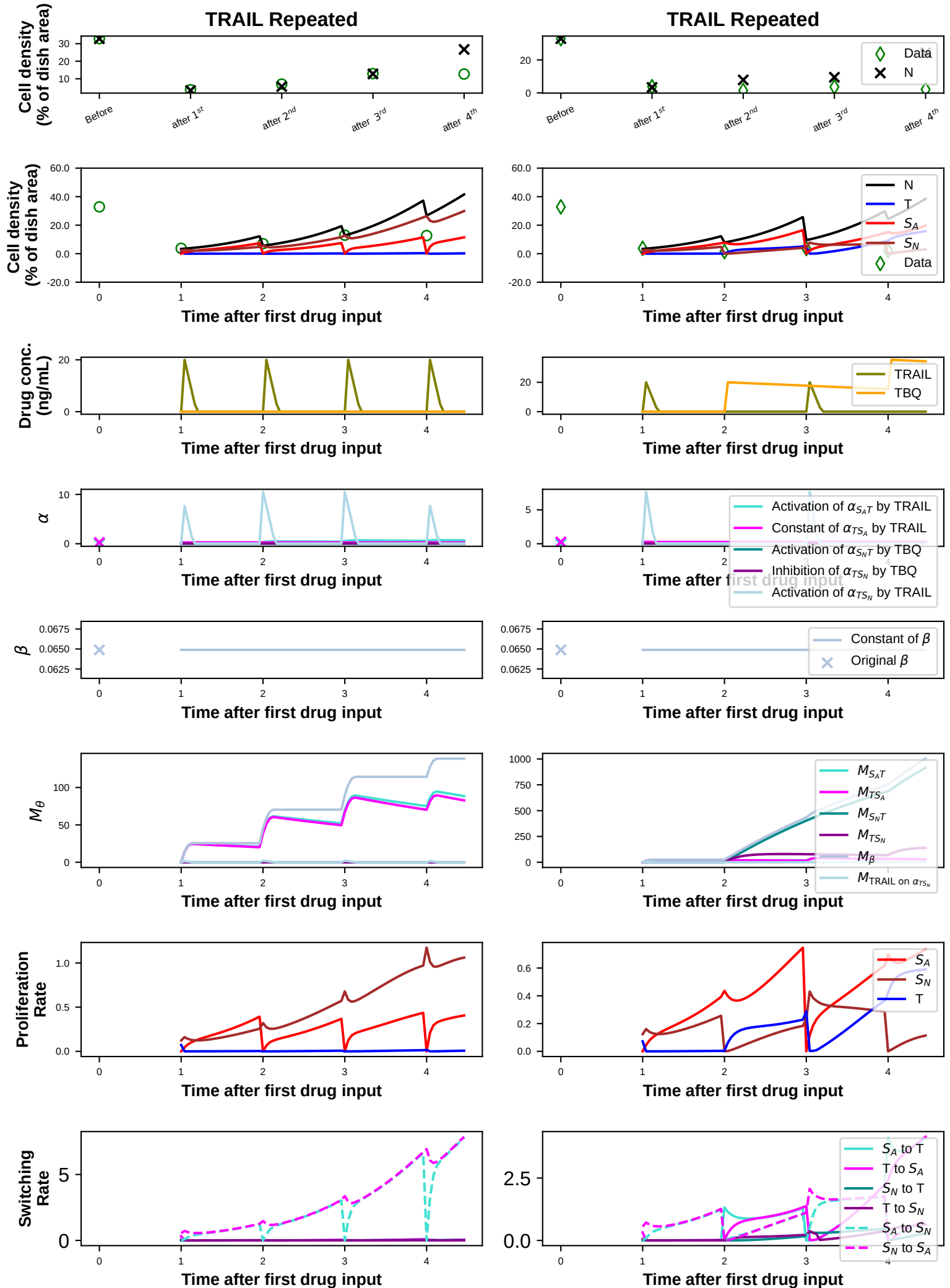

Supplement: Supplementary file 7 — Appendix Simulations Results [file 44320_2025_150_MOESM7_ESM.zip › Appendix_Simulations_Results/PSM2D_Simulations/PSM2_A_6_N_5.pdf]

# TRAIL/TBQ phenotypic switch Model A 7, Model N 1

RMSE AAAA = 2.7205, RMSE ANAN = 4.0997

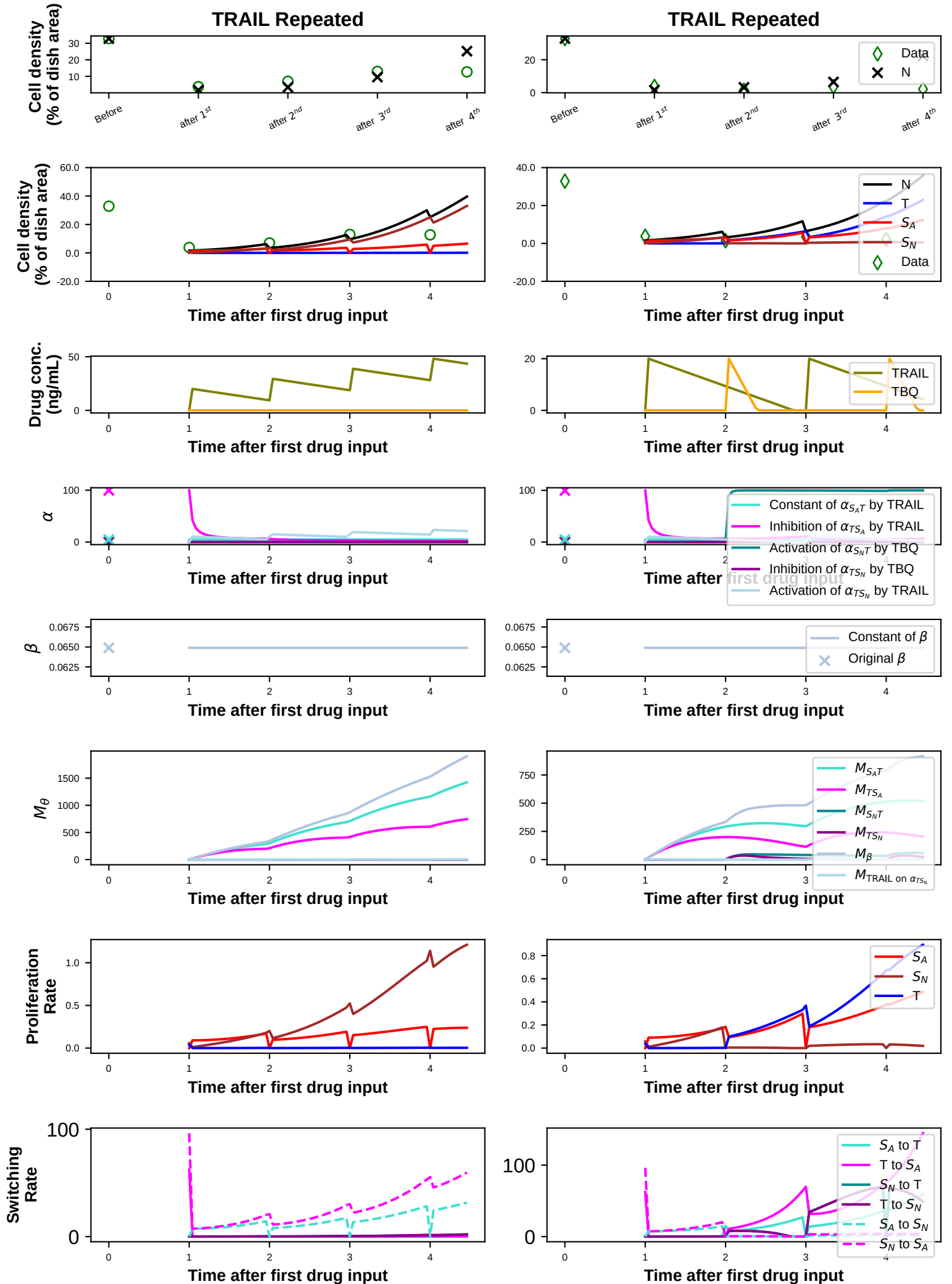

Supplement: Supplementary file 7 — Appendix Simulations Results [file 44320_2025_150_MOESM7_ESM.zip › Appendix_Simulations_Results/PSM2D_Simulations/PSM2_A_7_N_1.pdf]

TRAIL/TBQ phenotypic switch Model A 6, Model N 4  
RMSE AAAA = 2.8189, RMSE ANAN = 4.0185

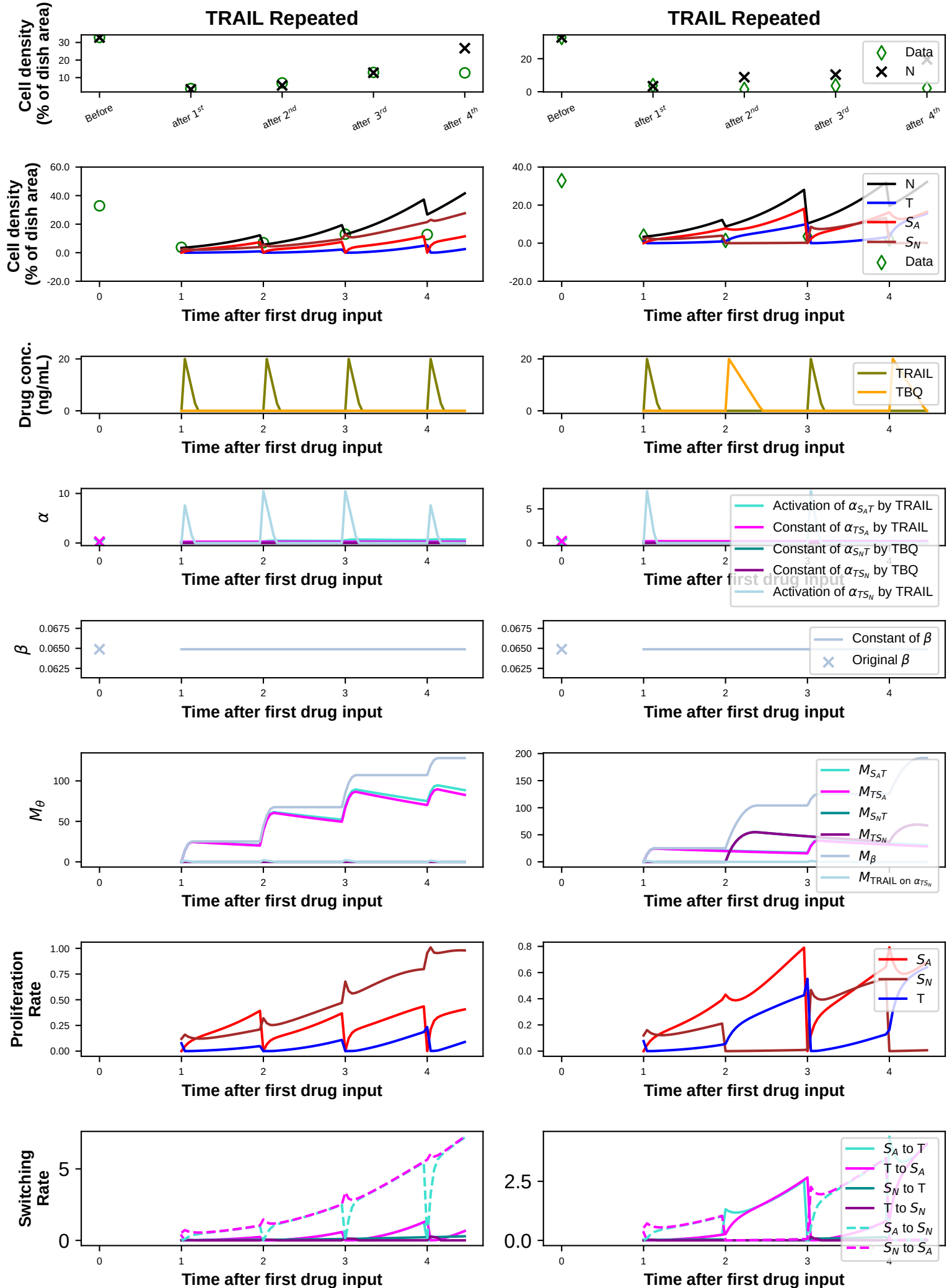

Supplement: Supplementary file 7 — Appendix Simulations Results [file 44320_2025_150_MOESM7_ESM.zip › Appendix_Simulations_Results/PSM2D_Simulations/PSM2_A_6_N_4.pdf]

# TRAIL/TBQ phenotypic switch Model A 2, Model N 8

RMSE AAAA = 1.5195, RMSE ANAN = 3.3584

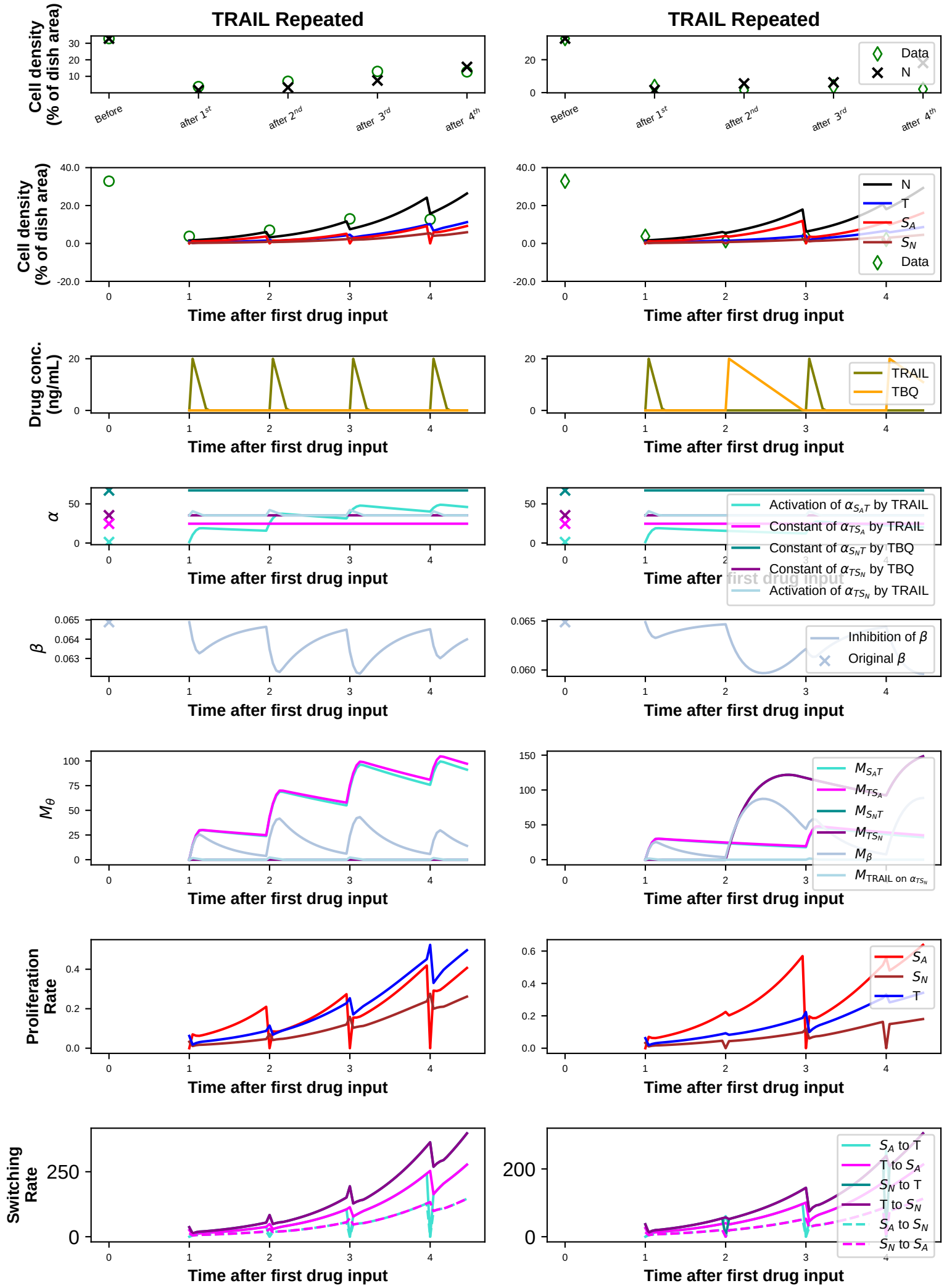

Supplement: Supplementary file 7 — Appendix Simulations Results [file 44320_2025_150_MOESM7_ESM.zip › Appendix_Simulations_Results/PSM2D_Simulations/PSM2_A_2_N_8.pdf]

TRAIL/TBQ phenotypic switch Model A 7, Model N 5  
RMSE AAAA = 2.7205, RMSE ANAN = 1.4318

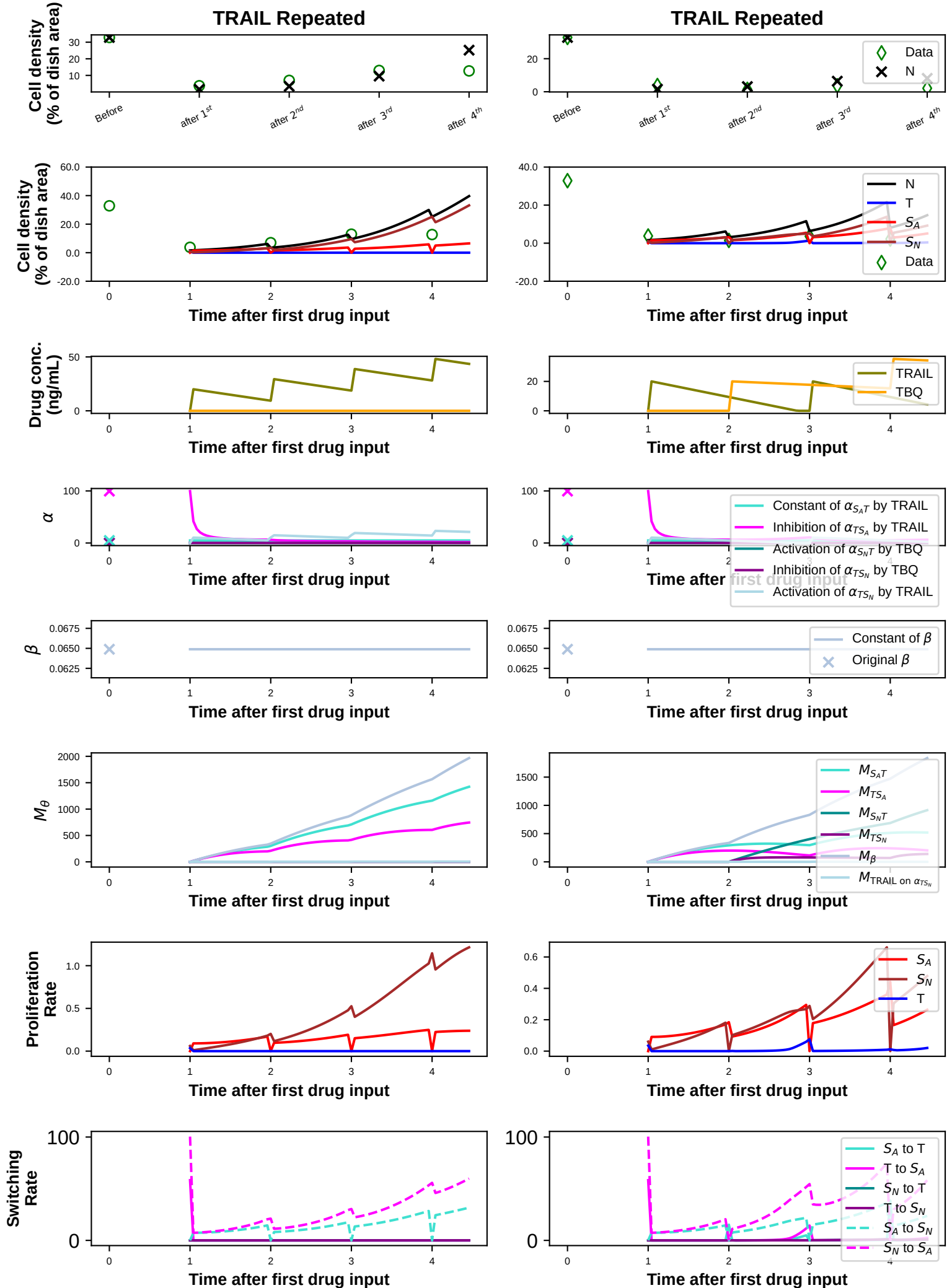

Supplement: Supplementary file 7 — Appendix Simulations Results [file 44320_2025_150_MOESM7_ESM.zip › Appendix_Simulations_Results/PSM2D_Simulations/PSM2_A_7_N_5.pdf]

TRAIL/TBQ phenotypic switch Model A 7, Model N 4  
RMSE AAAA = 2.7205, RMSE ANAN = 1.4057

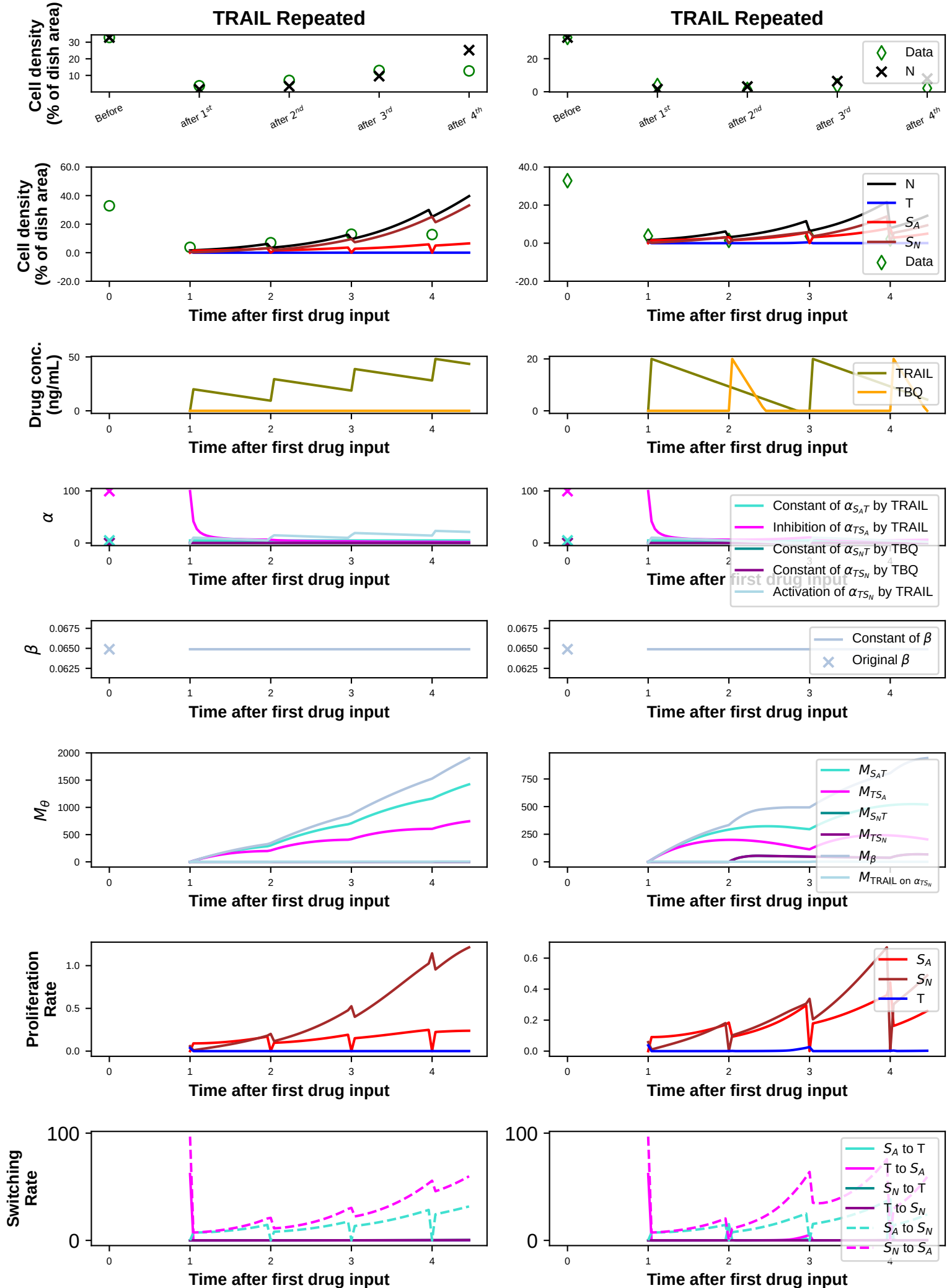

Supplement: Supplementary file 7 — Appendix Simulations Results [file 44320_2025_150_MOESM7_ESM.zip › Appendix_Simulations_Results/PSM2D_Simulations/PSM2_A_7_N_4.pdf]

# TRAIL/TBQ phenotypic switch Model A 6, Model N 1

RMSE AAAA = 2.8189, RMSE ANAN = 7.0952

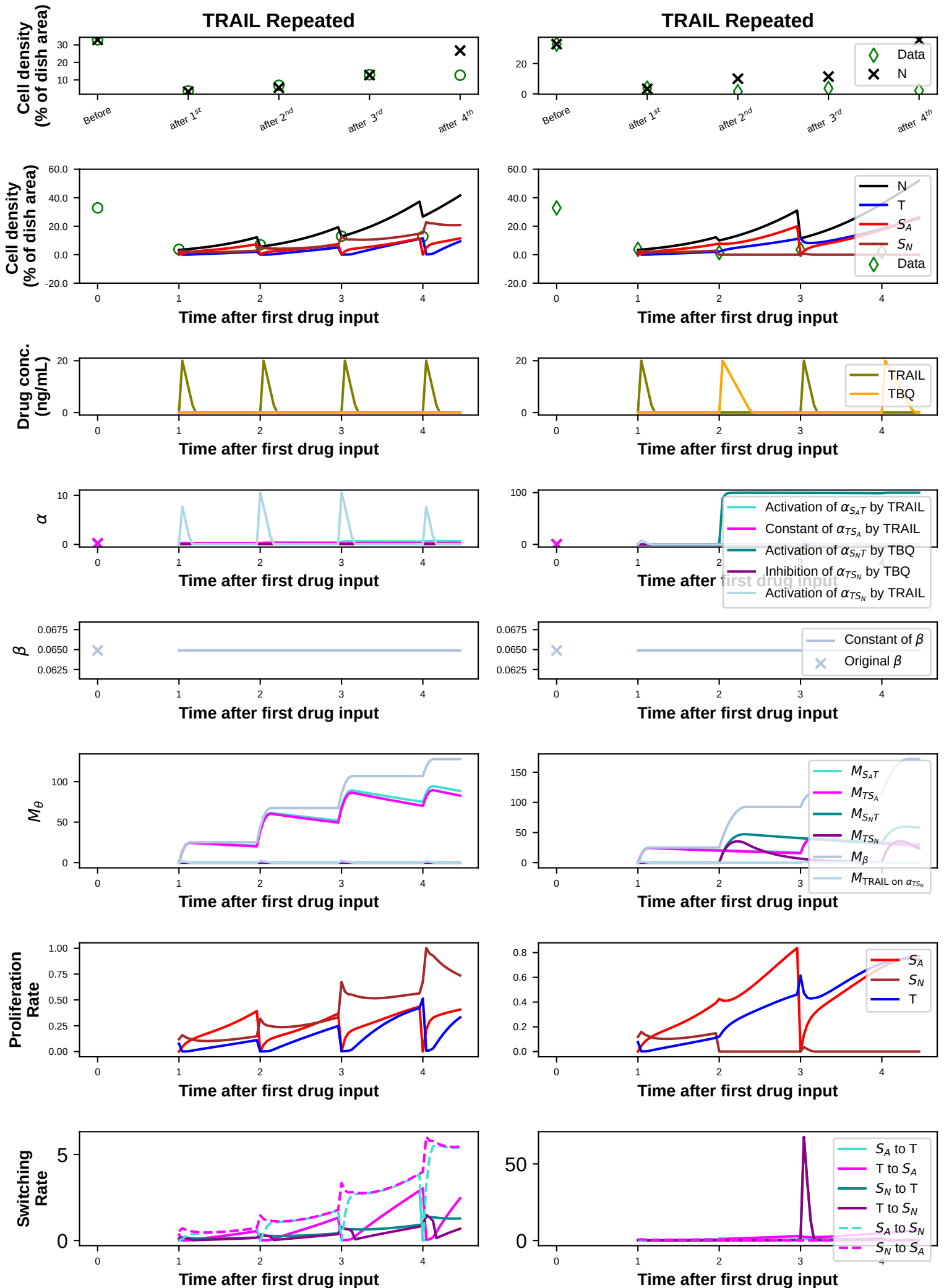

Supplement: Supplementary file 7 — Appendix Simulations Results [file 44320_2025_150_MOESM7_ESM.zip › Appendix_Simulations_Results/PSM2D_Simulations/PSM2_A_6_N_1.pdf]

# TRAIL/TBQ phenotypic switch Model A 6, Model N 3

RMSE AAAA = 2.8189, RMSE ANAN = 7.3629

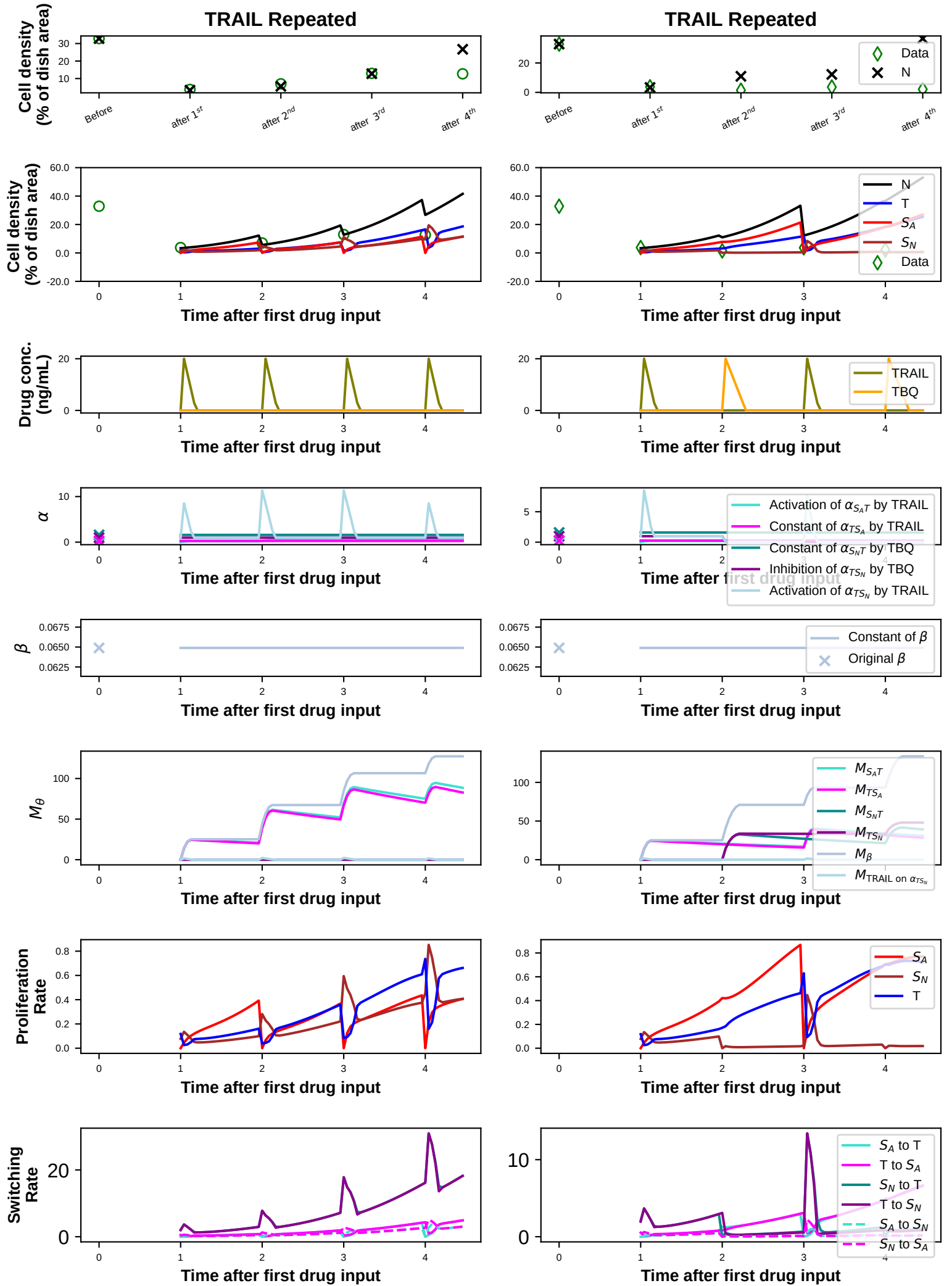

Supplement: Supplementary file 7 — Appendix Simulations Results [file 44320_2025_150_MOESM7_ESM.zip › Appendix_Simulations_Results/PSM2D_Simulations/PSM2_A_6_N_3.pdf]

# TRAIL/TBQ phenotypic switch Model A 7, Model N 6

RMSE AAAA = 2.7205, RMSE ANAN = 1.4263

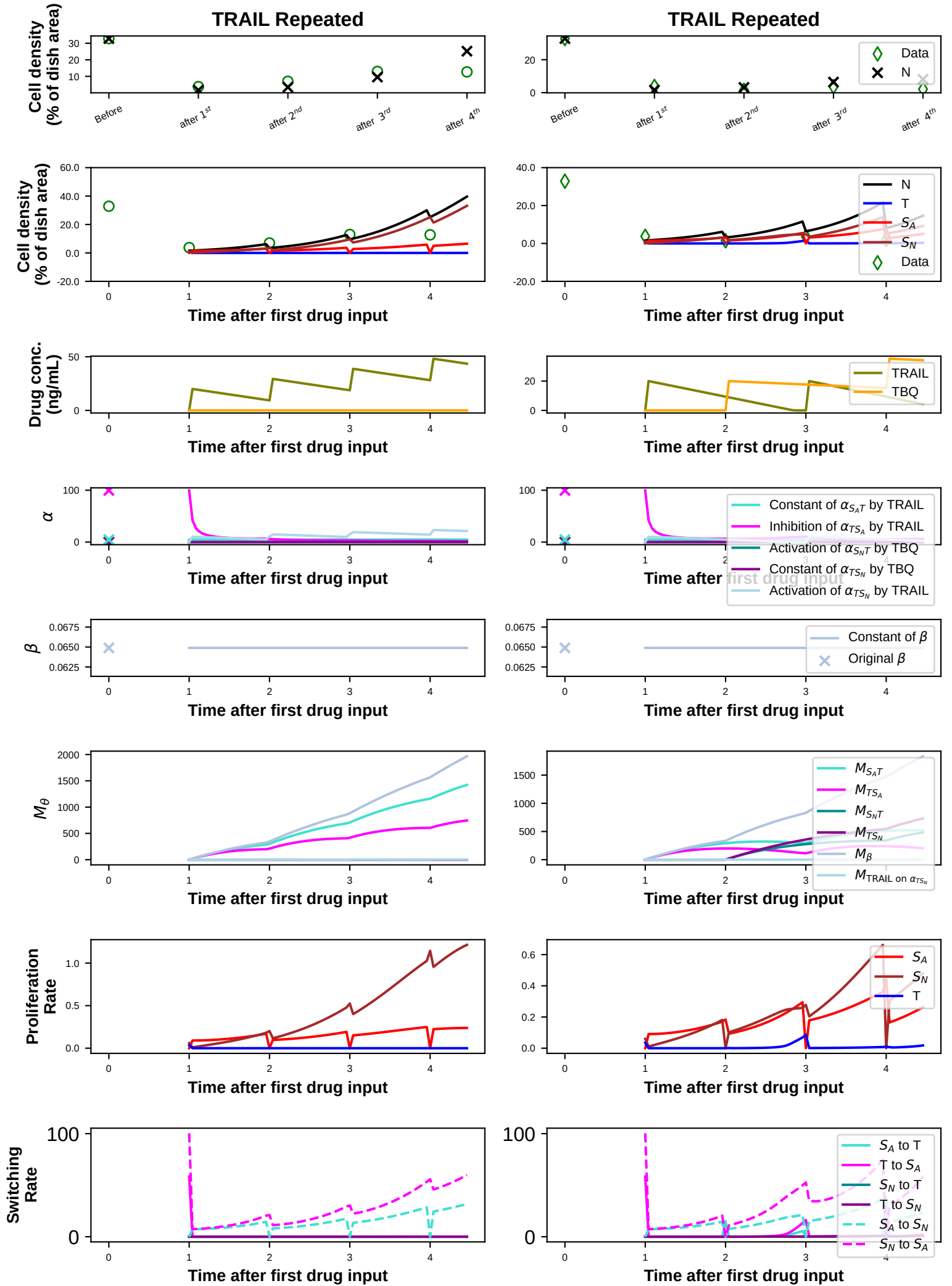

Supplement: Supplementary file 7 — Appendix Simulations Results [file 44320_2025_150_MOESM7_ESM.zip › Appendix_Simulations_Results/PSM2D_Simulations/PSM2_A_7_N_6.pdf]

TRAIL/TBQ phenotypic switch Model A 7, Model N 7  
RMSE AAAA = 2.7205, RMSE ANAN = 4.9546

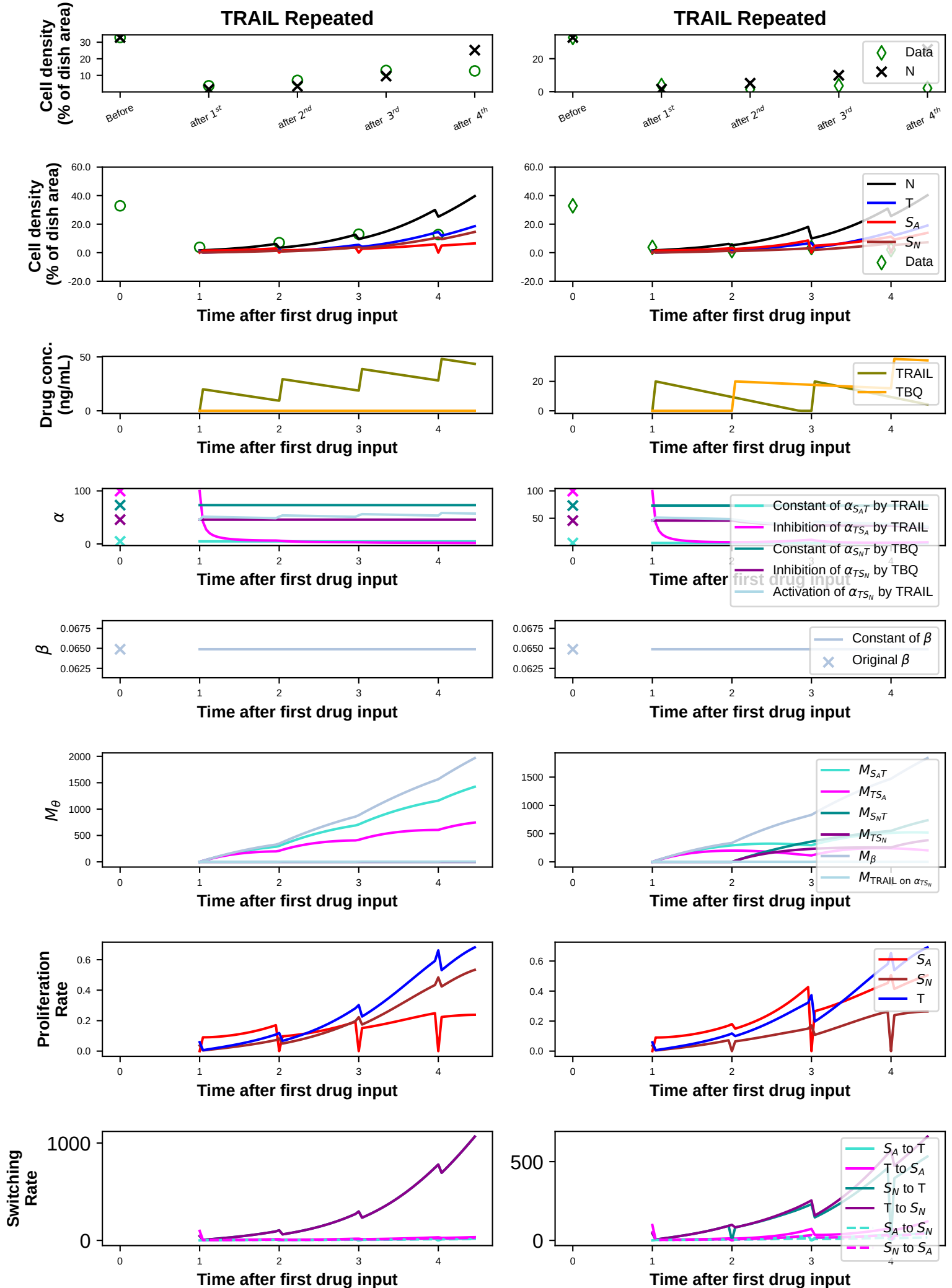

Supplement: Supplementary file 7 — Appendix Simulations Results [file 44320_2025_150_MOESM7_ESM.zip › Appendix_Simulations_Results/PSM2D_Simulations/PSM2_A_7_N_7.pdf]

TRAIL/TBQ phenotypic switch Model A 6, Model N 2  
RMSE AAAA = 2.8189, RMSE ANAN = 6.0281

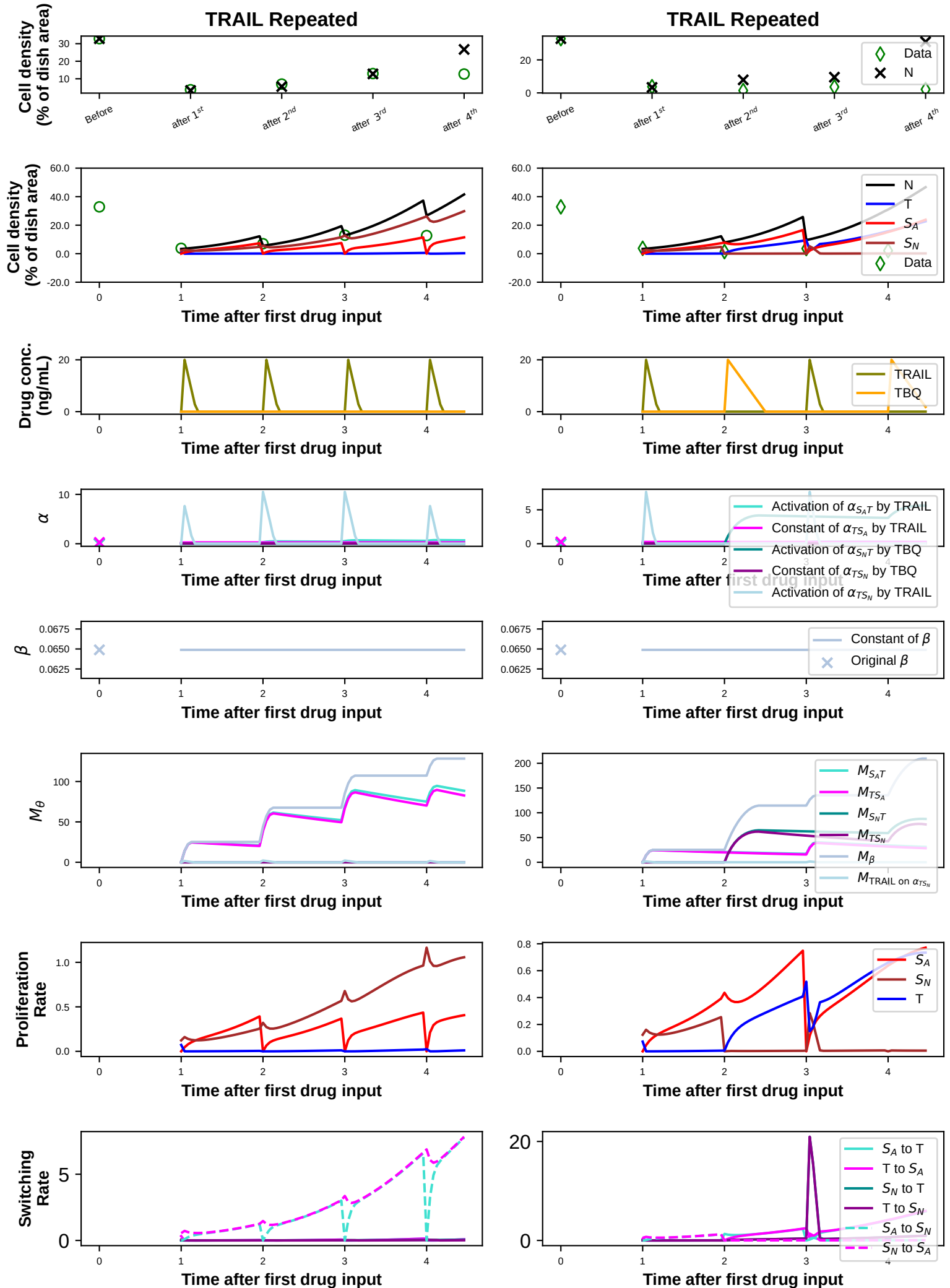

Supplement: Supplementary file 7 — Appendix Simulations Results [file 44320_2025_150_MOESM7_ESM.zip › Appendix_Simulations_Results/PSM2D_Simulations/PSM2_A_6_N_2.pdf]

TRAIL/TBQ phenotypic switch Model A 5, Model N 5  
RMSE AAAA = 2.0923, RMSE ANAN = 1.4941

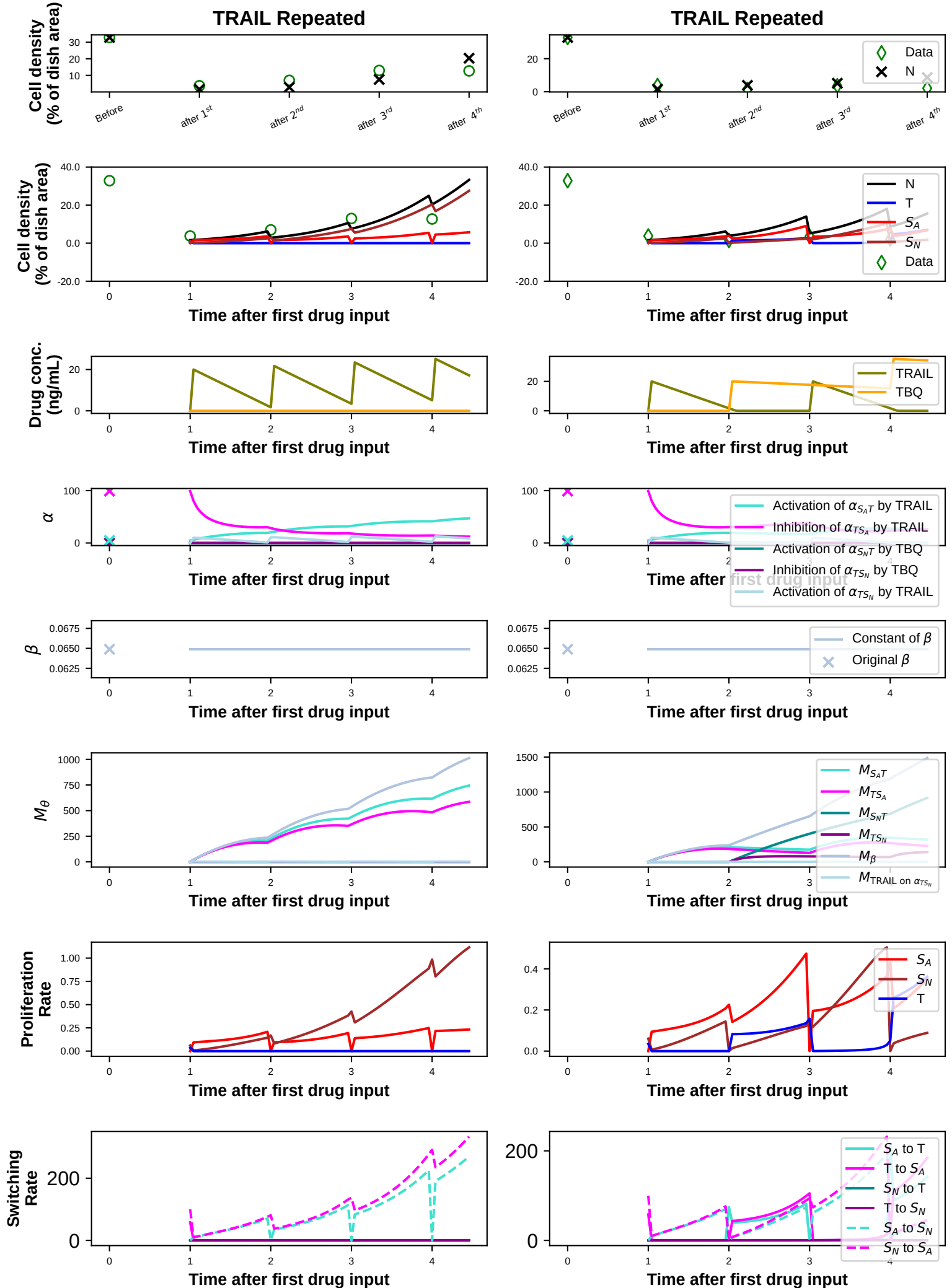

Supplement: Supplementary file 7 — Appendix Simulations Results [file 44320_2025_150_MOESM7_ESM.zip › Appendix_Simulations_Results/PSM2D_Simulations/PSM2_A_5_N_5.pdf]

TRAIL/TBQ phenotypic switch Model A 5, Model N 4  
RMSE AAAA = 2.0923, RMSE ANAN = 1.3742

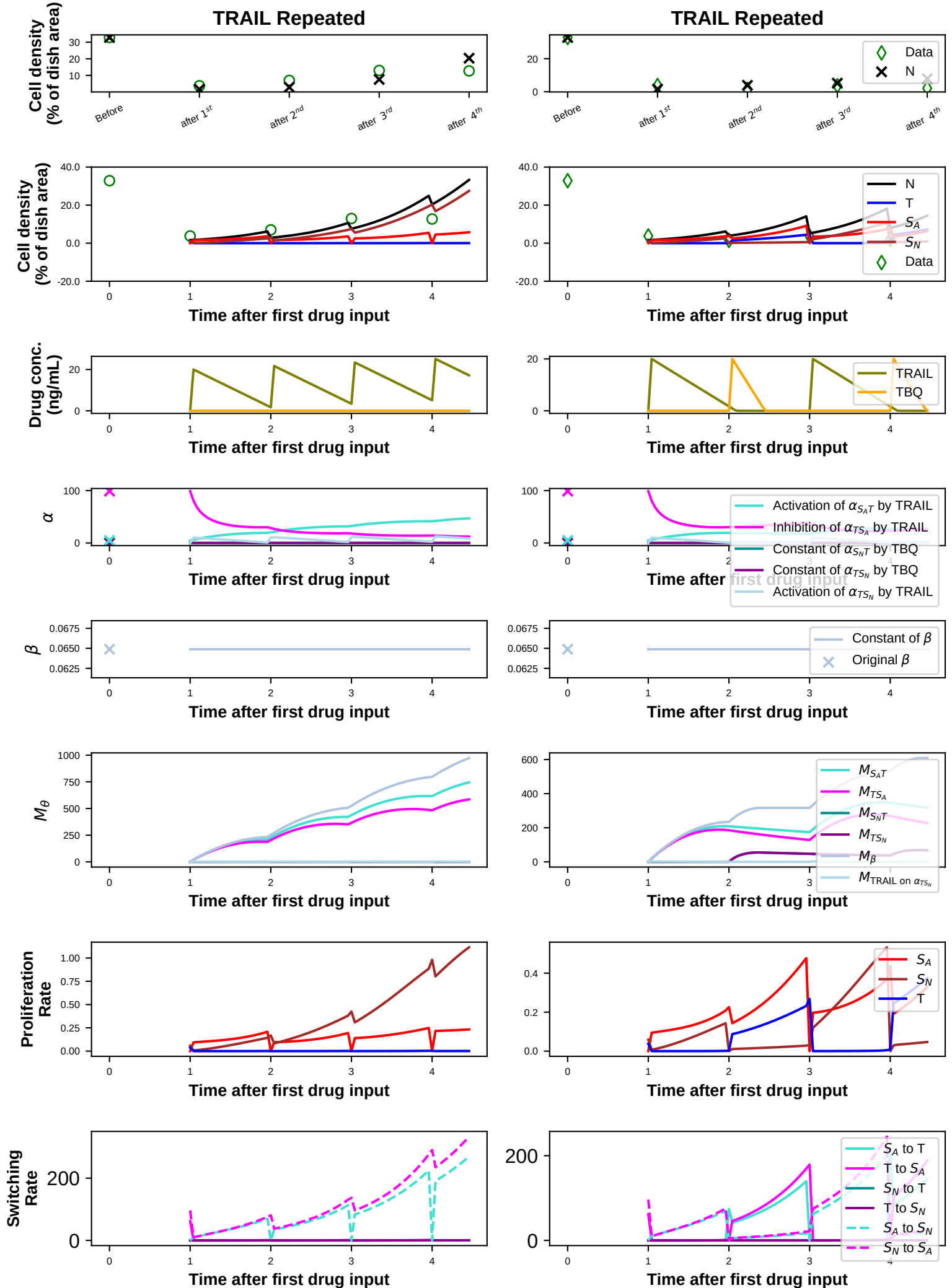

Supplement: Supplementary file 7 — Appendix Simulations Results [file 44320_2025_150_MOESM7_ESM.zip › Appendix_Simulations_Results/PSM2D_Simulations/PSM2_A_5_N_4.pdf]

**RMSE AAAA = 1.6097, RMSE ANAN = 4.219**

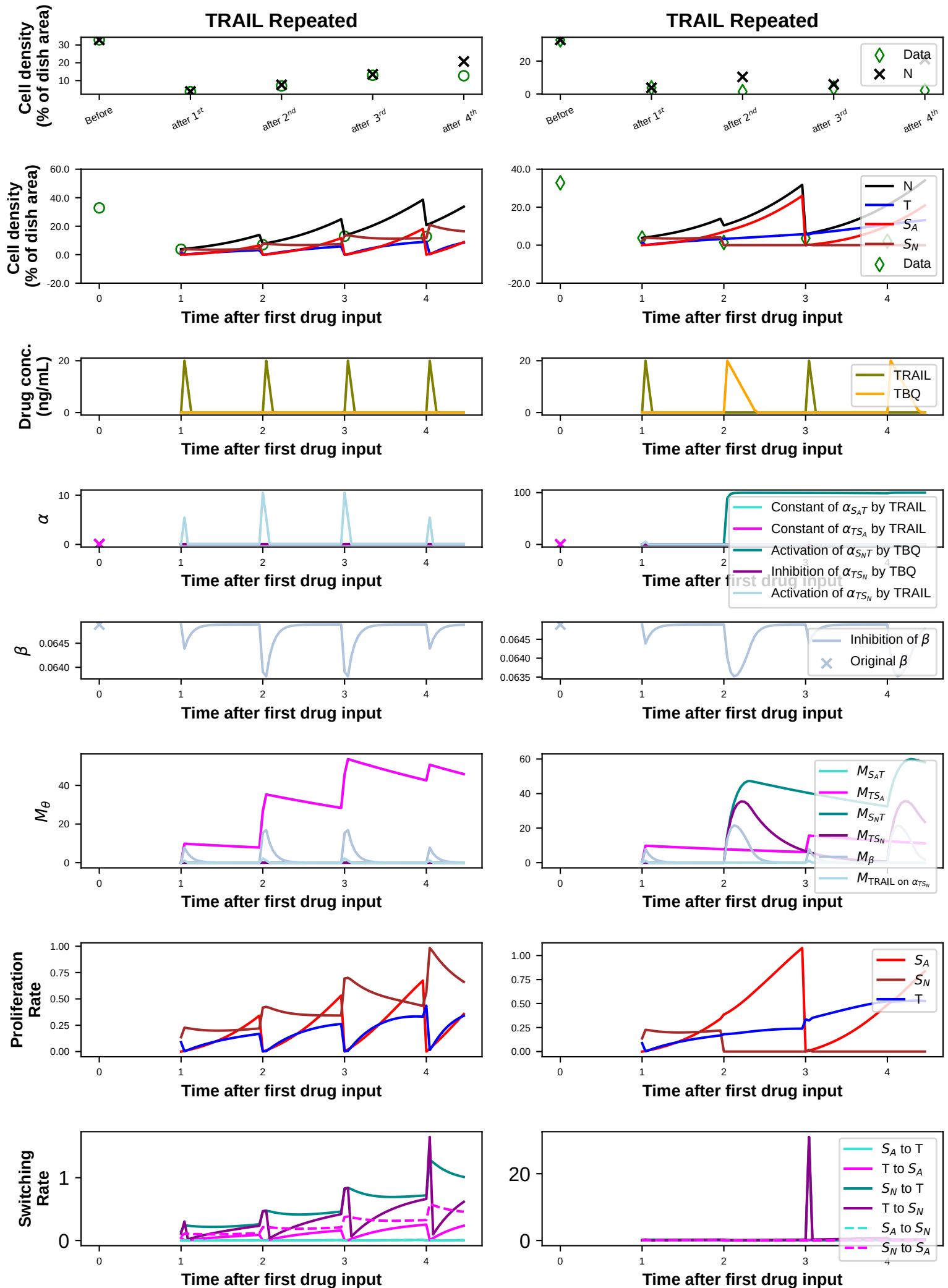

Supplement: Supplementary file 7 — Appendix Simulations Results [file 44320_2025_150_MOESM7_ESM.zip › Appendix_Simulations_Results/PSM2D_Simulations/PSM2_A_4_N_1.pdf]

TRAIL/TBQ phenotypic switch Model A 4, Model N 3  
RMSE AAAA = 1.6097, RMSE ANAN = 5.3262

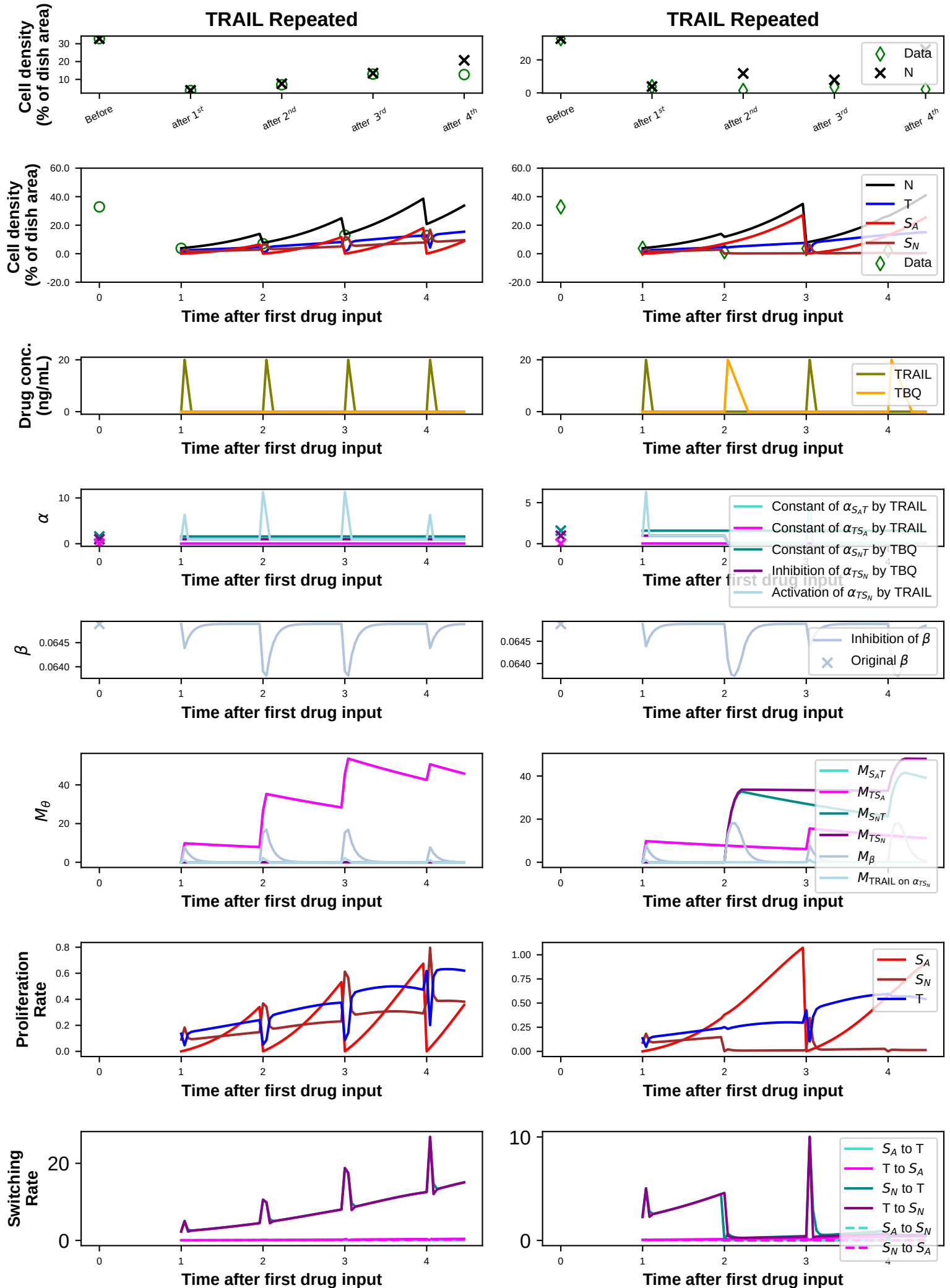

Supplement: Supplementary file 7 — Appendix Simulations Results [file 44320_2025_150_MOESM7_ESM.zip › Appendix_Simulations_Results/PSM2D_Simulations/PSM2_A_4_N_3.pdf]

TRAIL/TBQ phenotypic switch Model A 5, Model N 6  
RMSE AAAA = 2.0923, RMSE ANAN = 1.4691

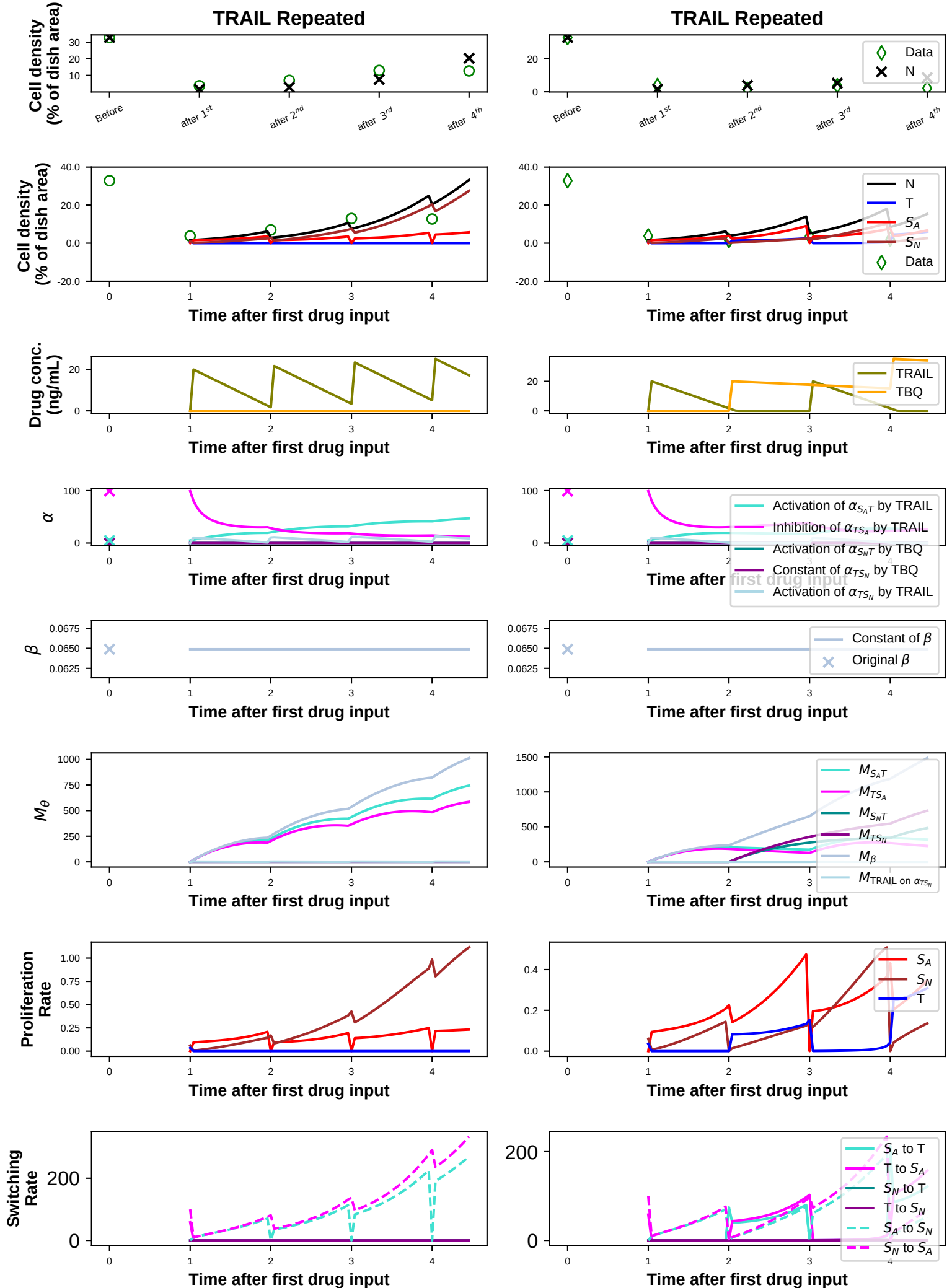

Supplement: Supplementary file 7 — Appendix Simulations Results [file 44320_2025_150_MOESM7_ESM.zip › Appendix_Simulations_Results/PSM2D_Simulations/PSM2_A_5_N_6.pdf]

TRAIL/TBQ phenotypic switch Model A 5, Model N 7  
RMSE AAAA = 2.0923, RMSE ANAN = 3.8086

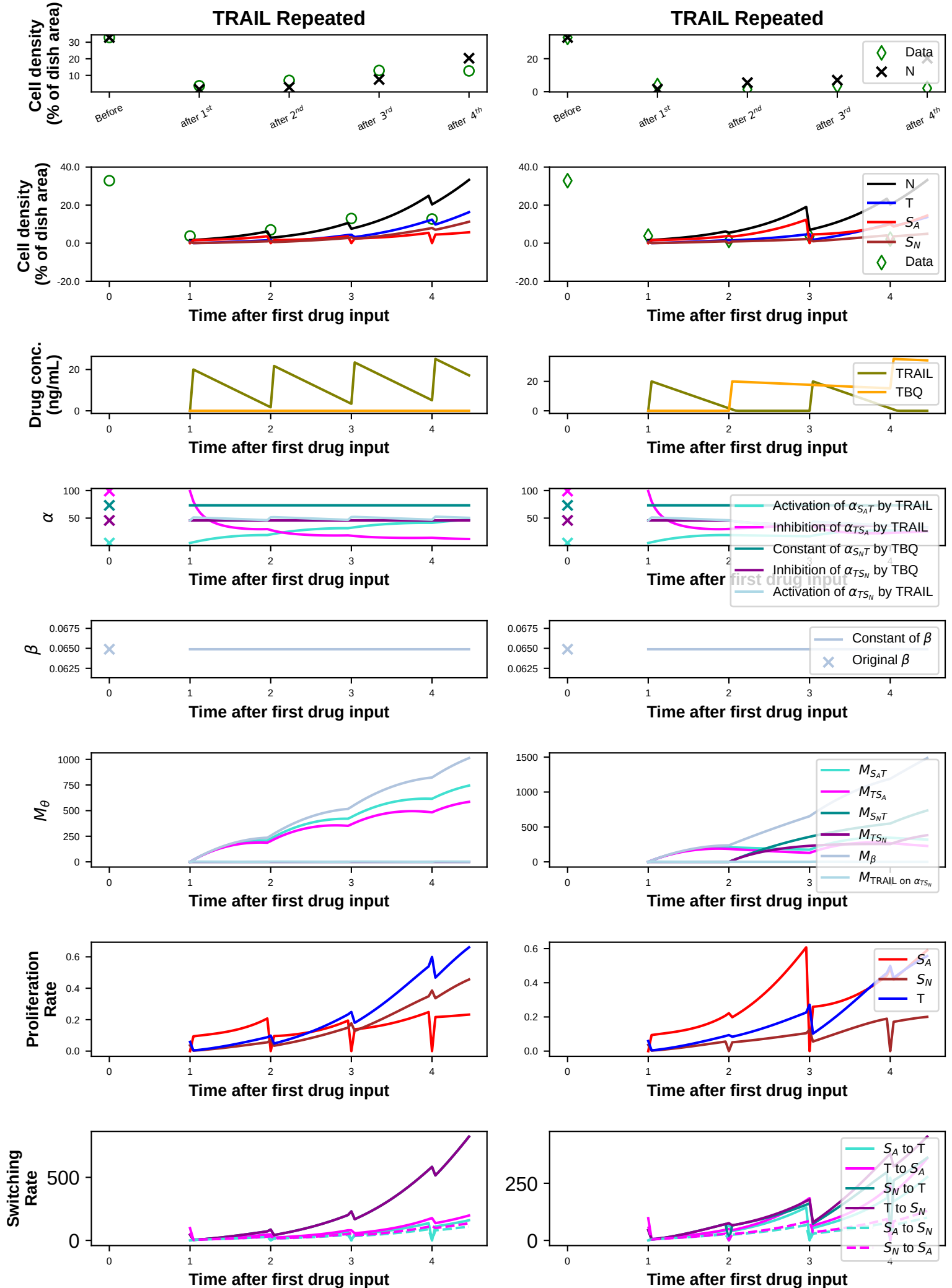

Supplement: Supplementary file 7 — Appendix Simulations Results [file 44320_2025_150_MOESM7_ESM.zip › Appendix_Simulations_Results/PSM2D_Simulations/PSM2_A_5_N_7.pdf]

# TRAIL/TBQ phenotypic switch Model A 4, Model N 2

RMSE AAAA = 1.6097, RMSE ANAN = 1.3091

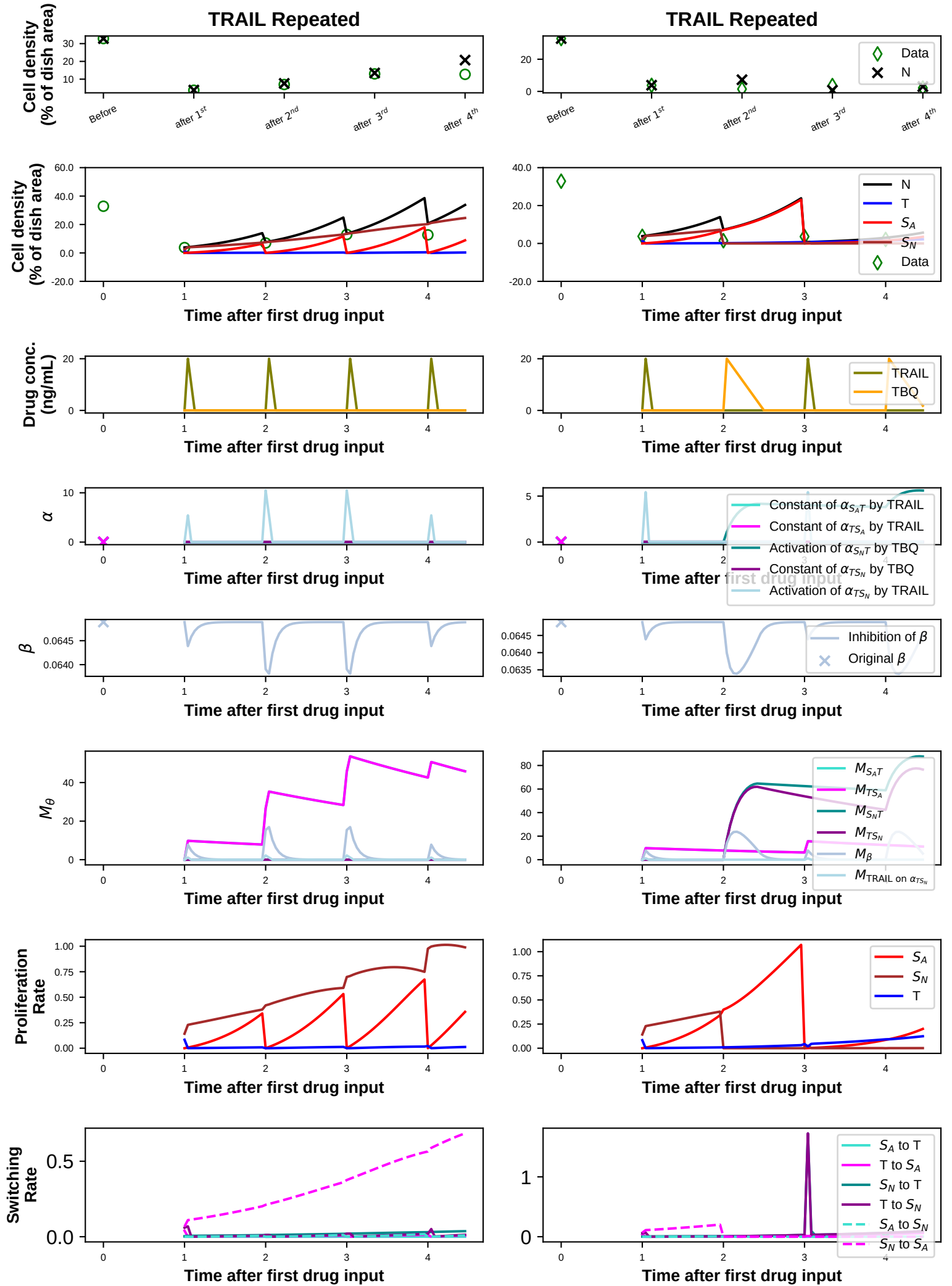

Supplement: Supplementary file 7 — Appendix Simulations Results [file 44320_2025_150_MOESM7_ESM.zip › Appendix_Simulations_Results/PSM2D_Simulations/PSM2_A_4_N_2.pdf]

TRAIL/TBQ phenotypic switch Model A 4, Model N 6  
RMSE AAAA = 1.6093, RMSE ANAN = 1.2979

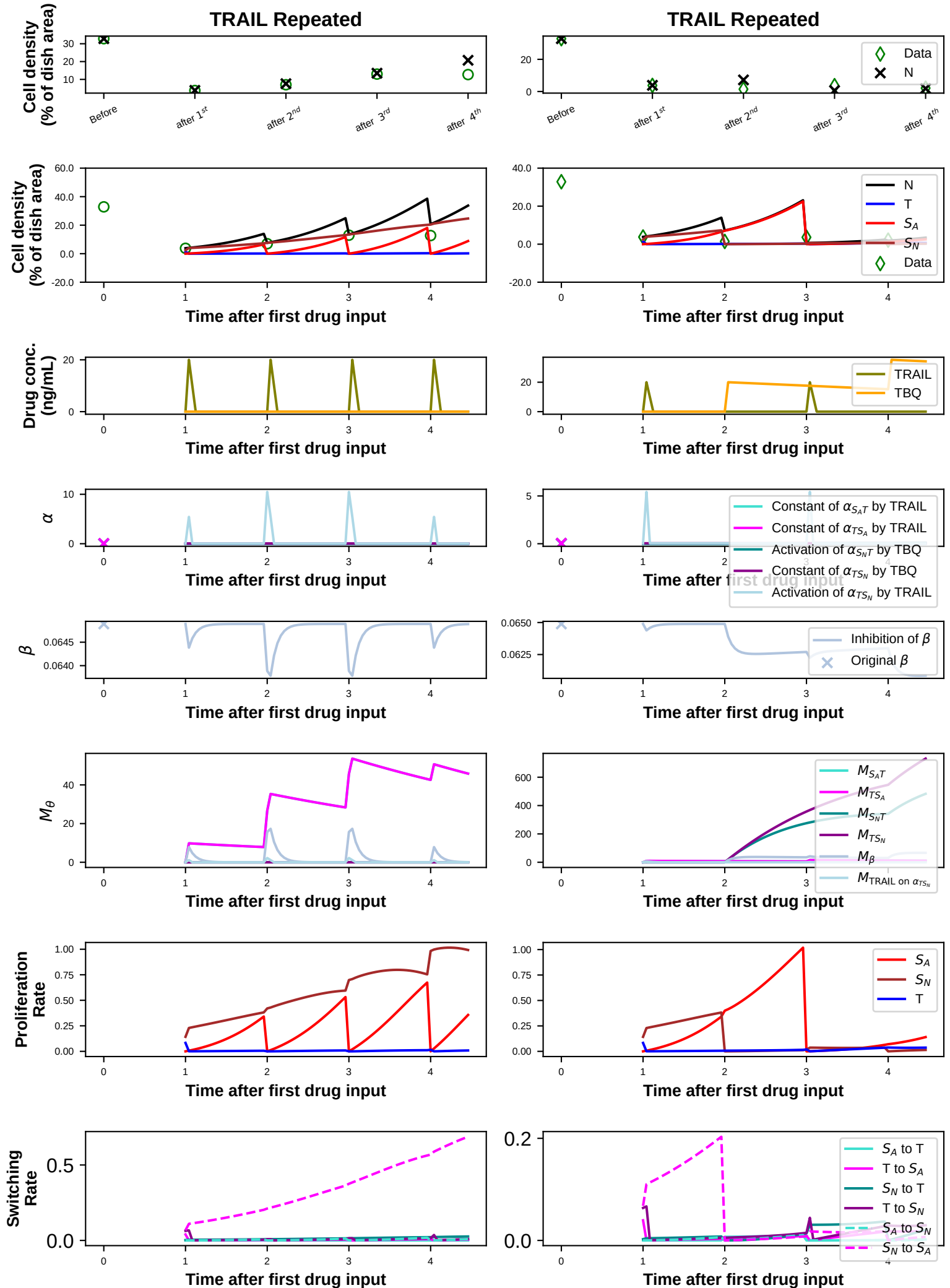

Supplement: Supplementary file 7 — Appendix Simulations Results [file 44320_2025_150_MOESM7_ESM.zip › Appendix_Simulations_Results/PSM2D_Simulations/PSM2_A_4_N_6.pdf]

TRAIL/TBQ phenotypic switch Model A 5, Model N 3  
RMSE AAAA = 2.0923, RMSE ANAN = 3.6685

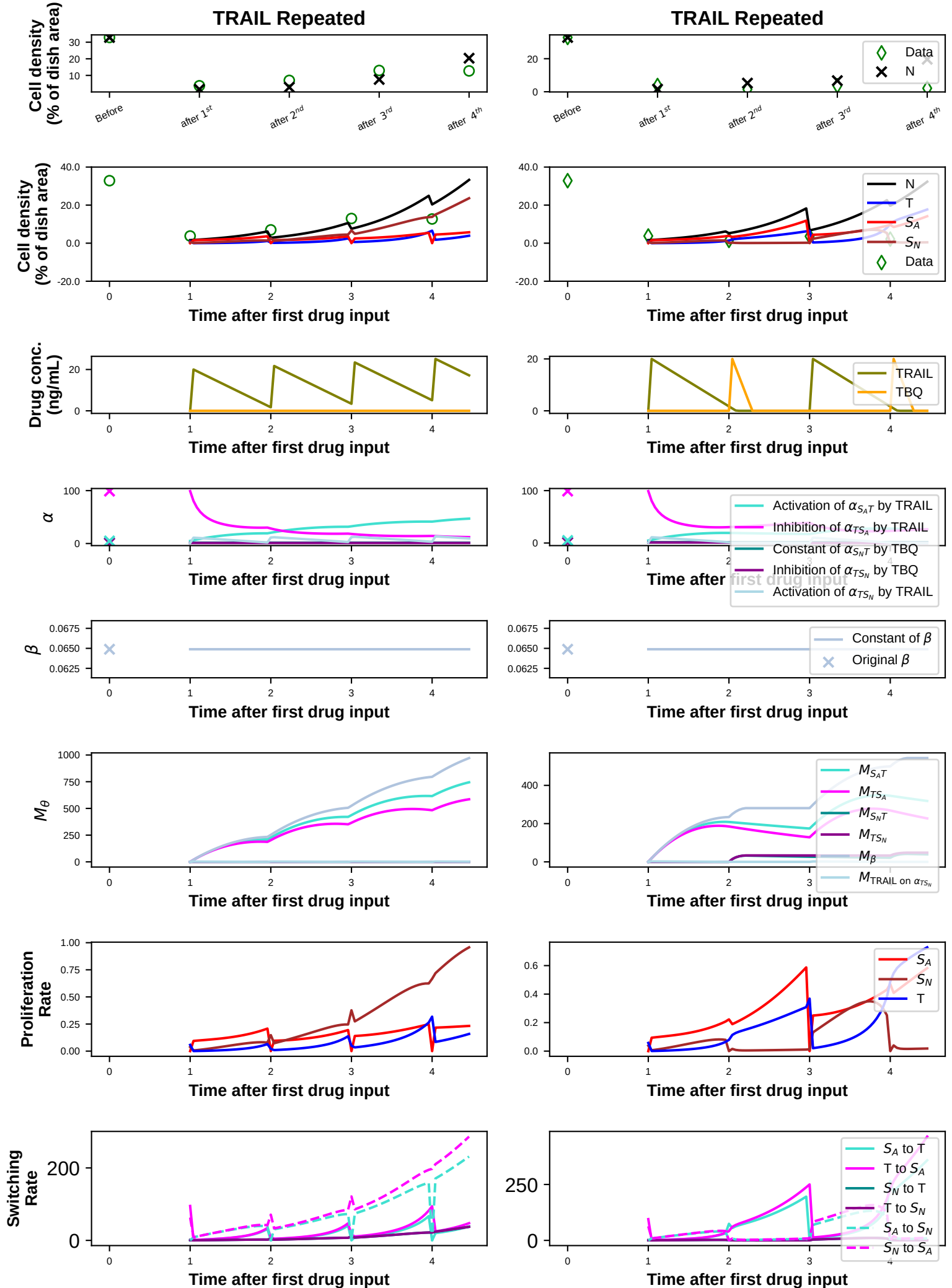

Supplement: Supplementary file 7 — Appendix Simulations Results [file 44320_2025_150_MOESM7_ESM.zip › Appendix_Simulations_Results/PSM2D_Simulations/PSM2_A_5_N_3.pdf]

TRAIL/TBQ phenotypic switch Model A 8, Model N 8  
RMSE AAAA = 3.8738, RMSE ANAN = 1.4034

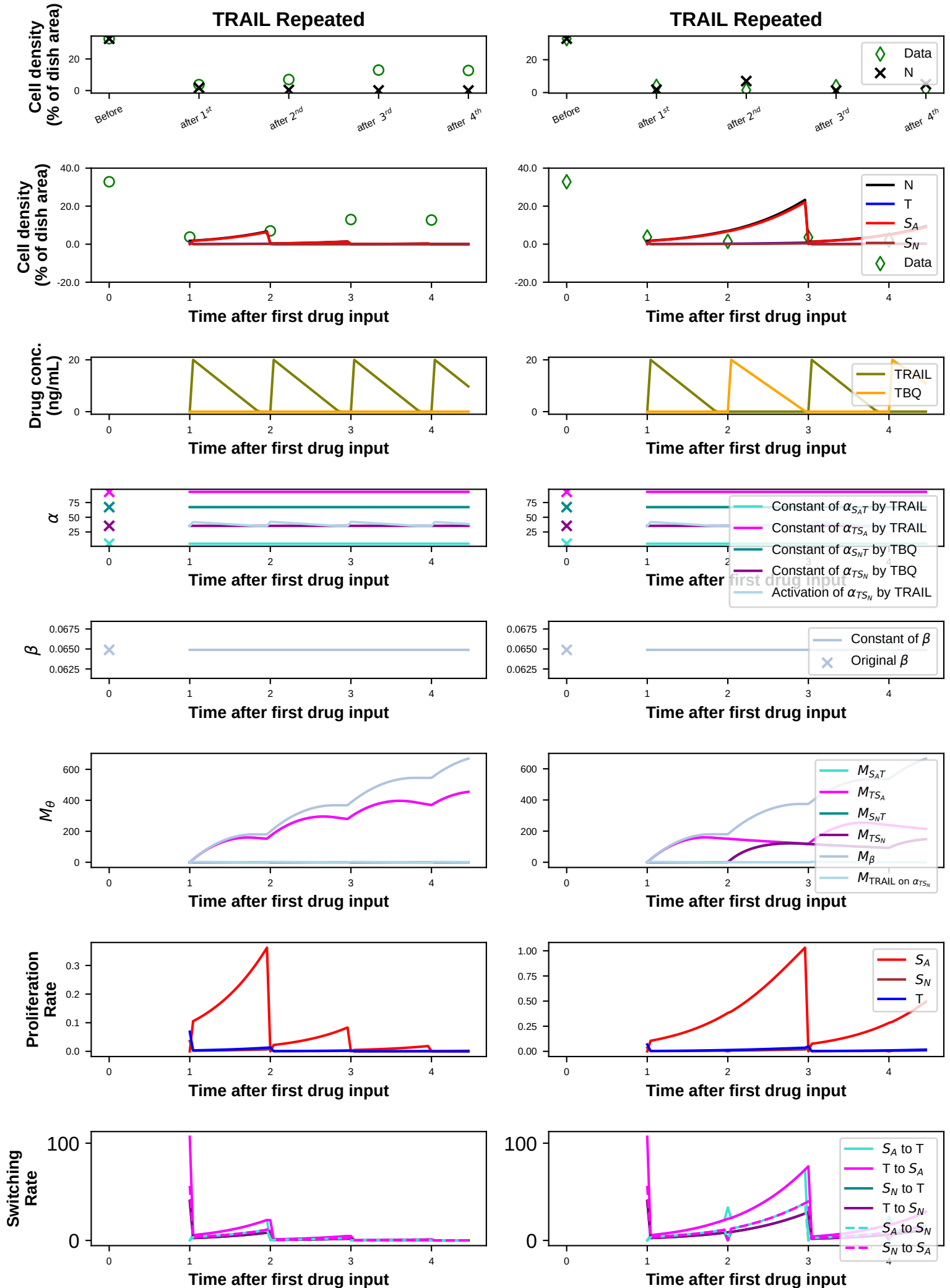

Supplement: Supplementary file 7 — Appendix Simulations Results [file 44320_2025_150_MOESM7_ESM.zip › Appendix_Simulations_Results/PSM2D_Simulations/PSM2_A_8_N_8.pdf]

TRAIL/TBQ phenotypic switch Model A 5, Model N 2  
RMSE AAAA = 2.0923, RMSE ANAN = 3.1729

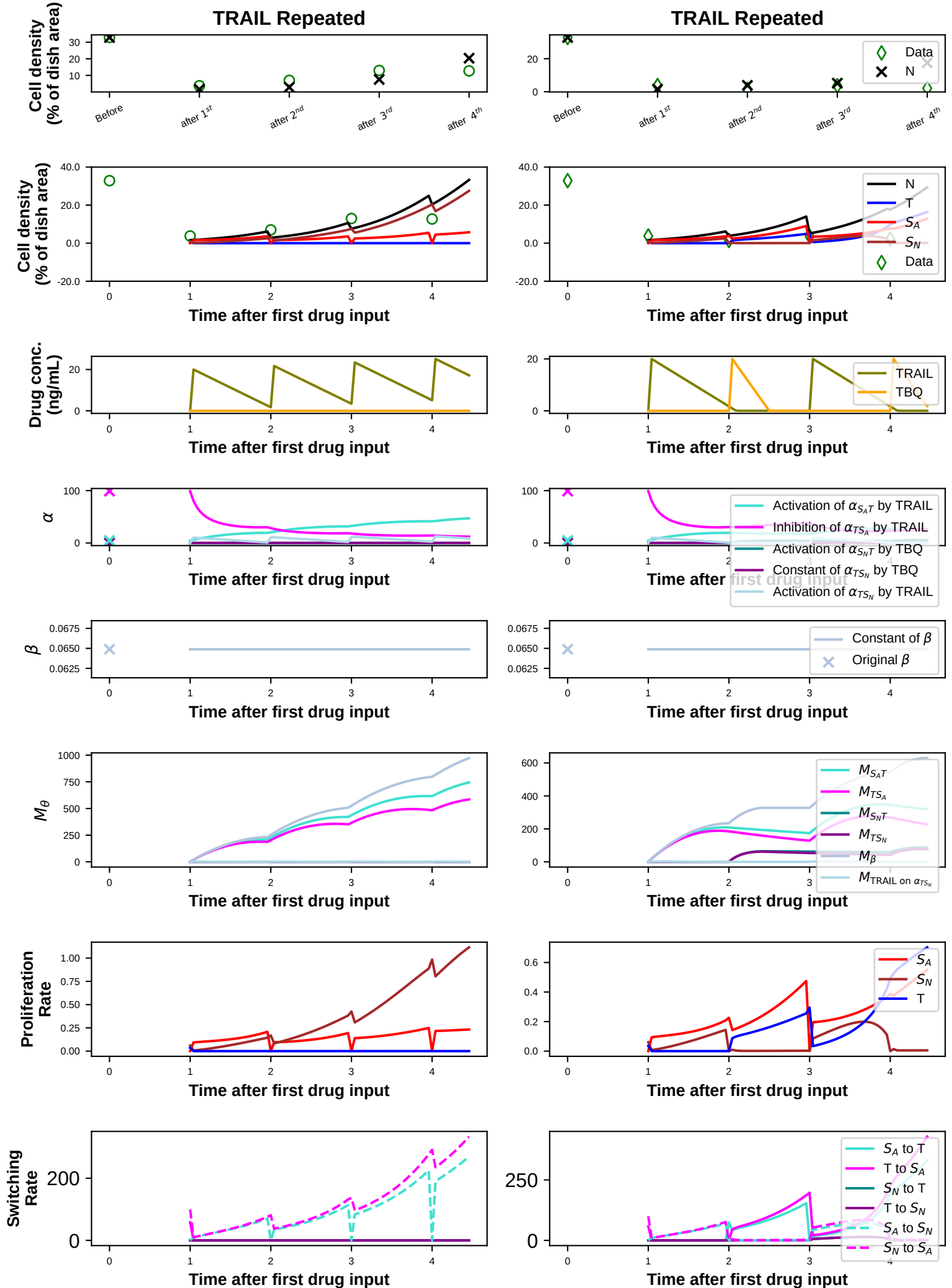

Supplement: Supplementary file 7 — Appendix Simulations Results [file 44320_2025_150_MOESM7_ESM.zip › Appendix_Simulations_Results/PSM2D_Simulations/PSM2_A_5_N_2.pdf]

TRAIL/TBQ phenotypic switch Model A 4, Model N 7  
RMSE AAAA = 1.6093, RMSE ANAN = 4.483

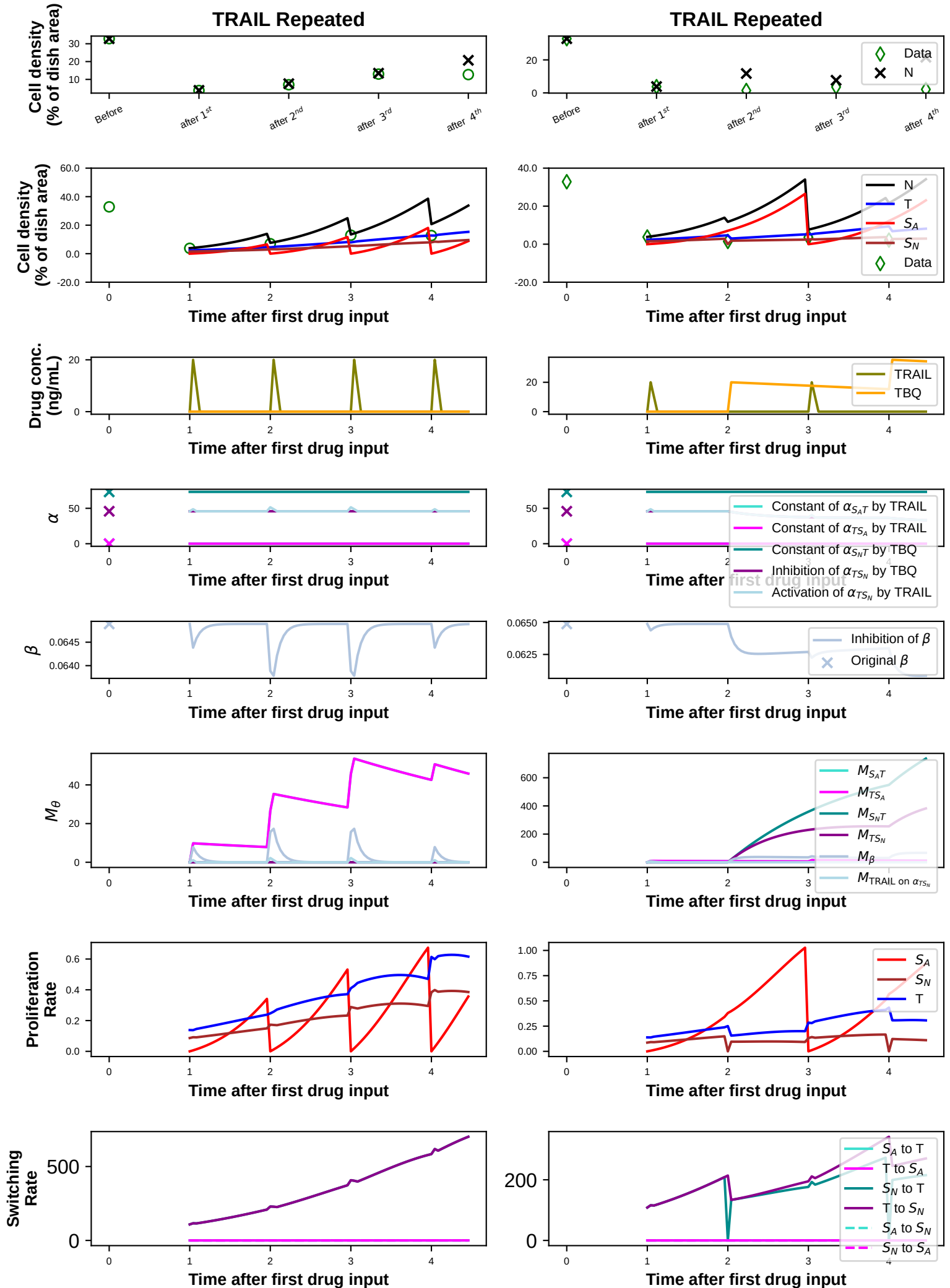

Supplement: Supplementary file 7 — Appendix Simulations Results [file 44320_2025_150_MOESM7_ESM.zip › Appendix_Simulations_Results/PSM2D_Simulations/PSM2_A_4_N_7.pdf]

TRAIL/TBQ phenotypic switch Model A 4, Model N 5  
RMSE AAAA = 1.6093, RMSE ANAN = 1.2973

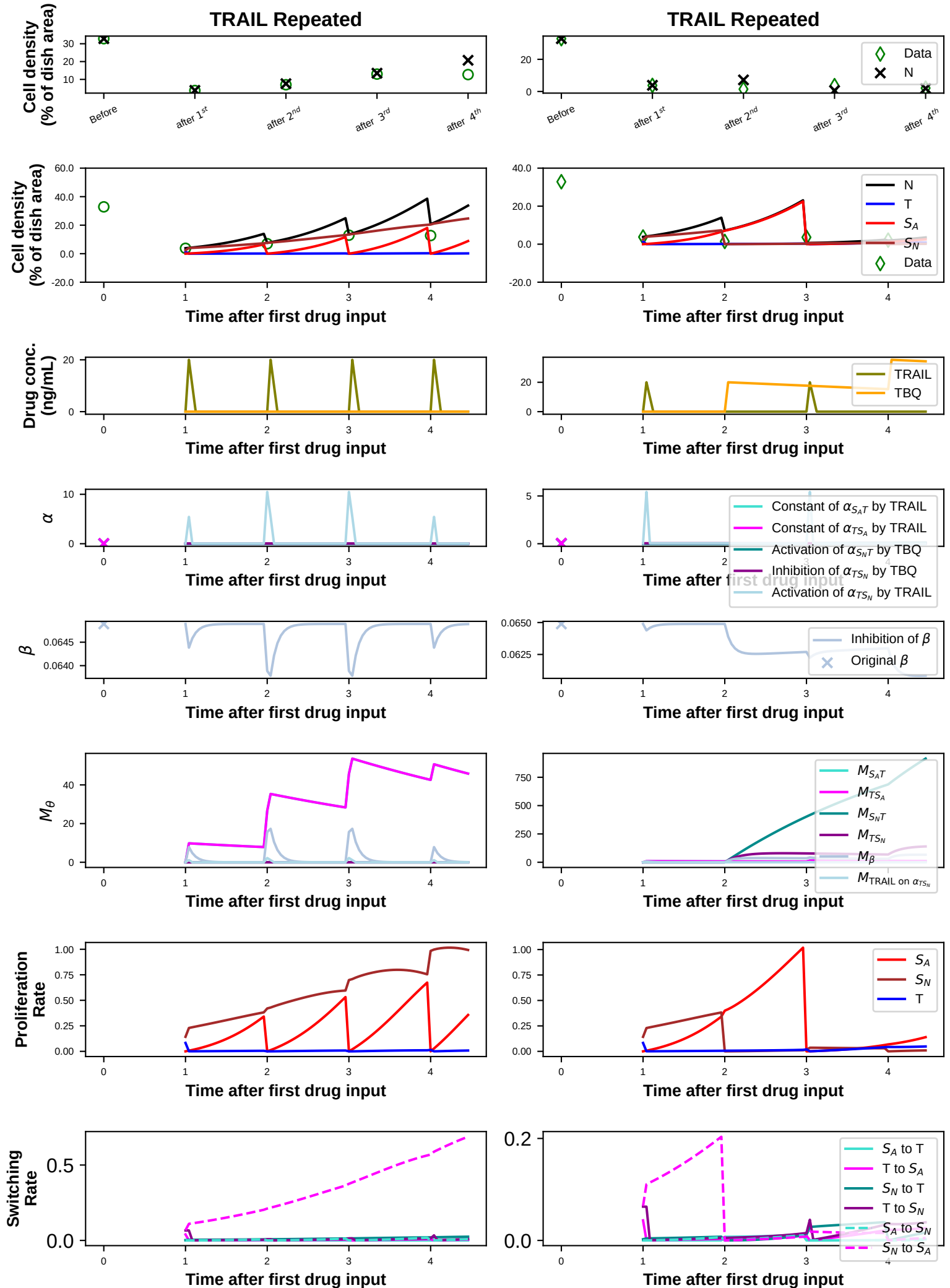

Supplement: Supplementary file 7 — Appendix Simulations Results [file 44320_2025_150_MOESM7_ESM.zip › Appendix_Simulations_Results/PSM2D_Simulations/PSM2_A_4_N_5.pdf]

TRAIL/TBQ phenotypic switch Model A 1, Model N 8  
RMSE AAAA = 1.4524, RMSE ANAN = 2.0015

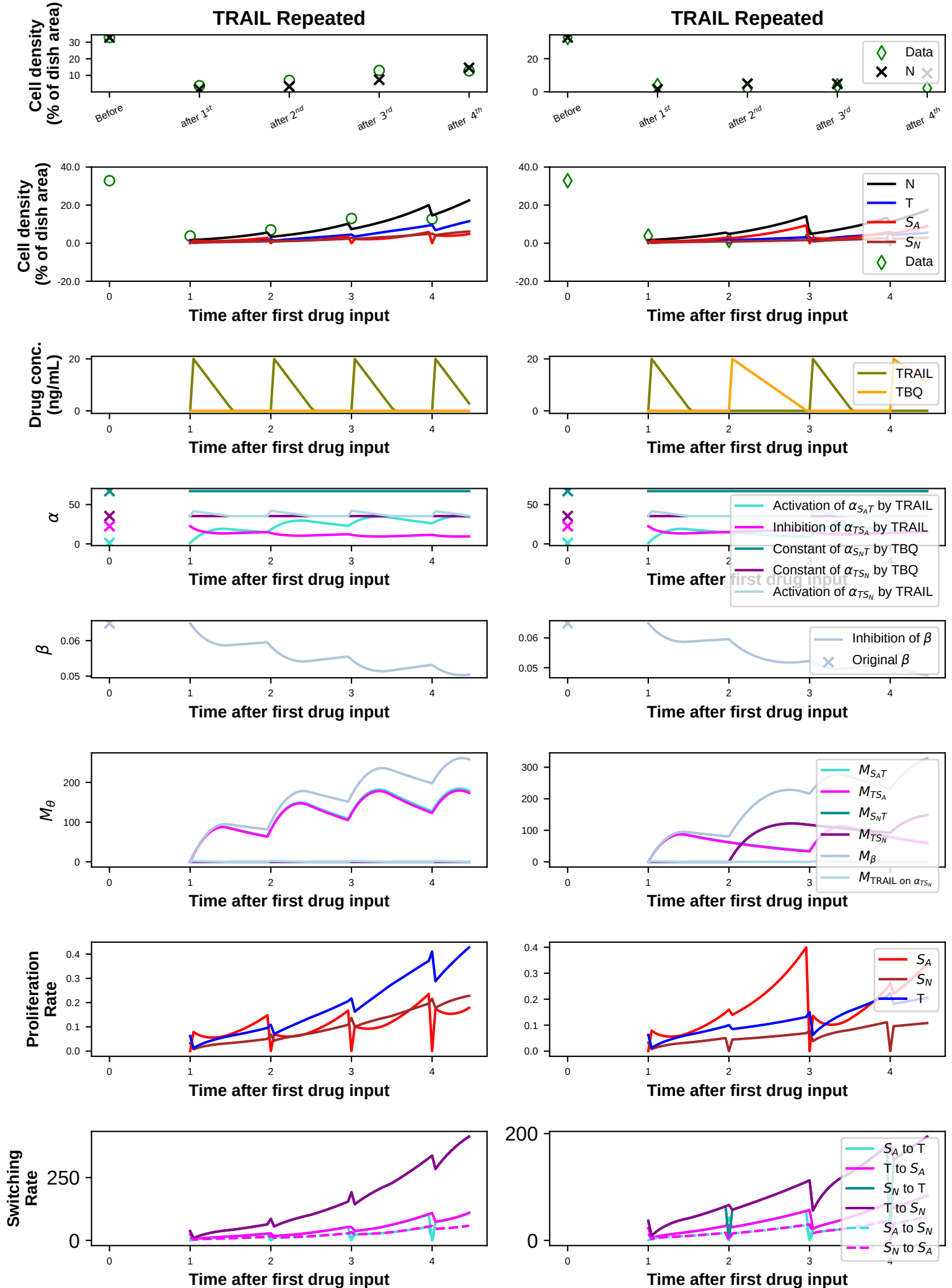

Supplement: Supplementary file 7 — Appendix Simulations Results [file 44320_2025_150_MOESM7_ESM.zip › Appendix_Simulations_Results/PSM2D_Simulations/PSM2_A_1_N_8.pdf]

# TRAIL/TBQ phenotypic switch Model A 5, Model N 1

RMSE AAAA = 2.0923, RMSE ANAN = 3.5814

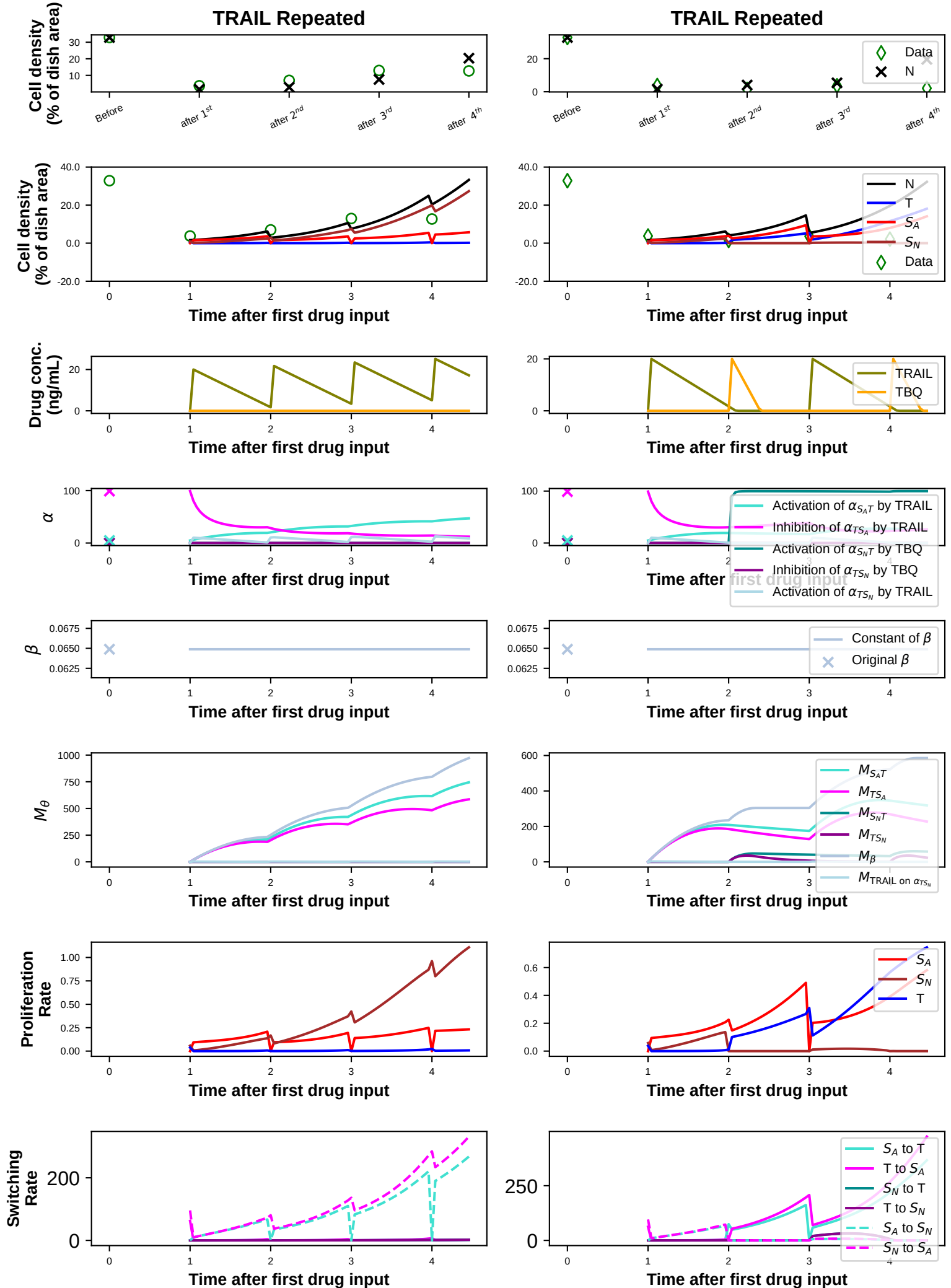

Supplement: Supplementary file 7 — Appendix Simulations Results [file 44320_2025_150_MOESM7_ESM.zip › Appendix_Simulations_Results/PSM2D_Simulations/PSM2_A_5_N_1.pdf]

TRAIL/TBQ phenotypic switch Model A 4, Model N 4  
RMSE AAAA = 1.6097, RMSE ANAN = 1.7826

TRAIL Repeated

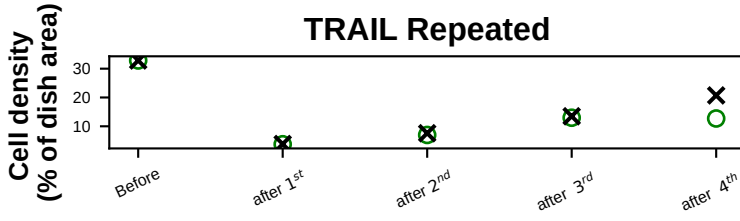

TRAIL Repeated

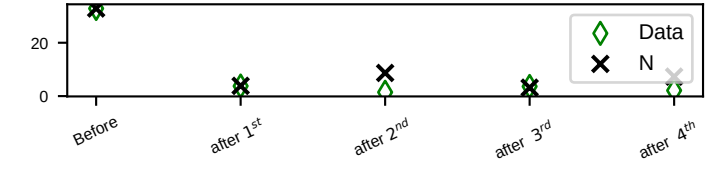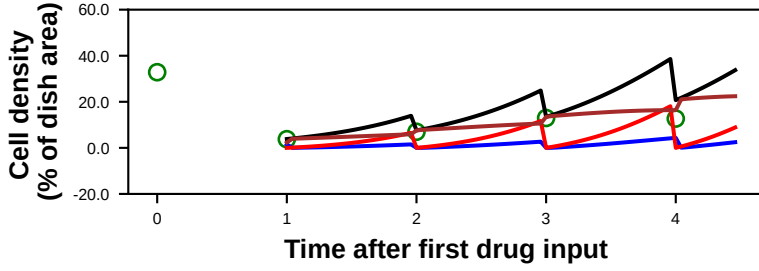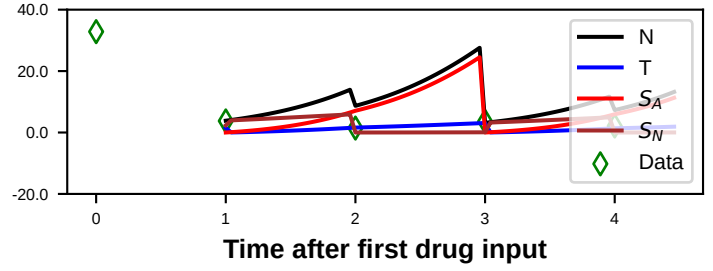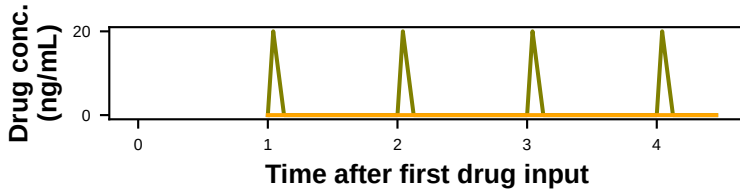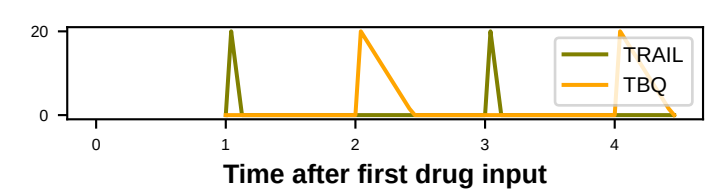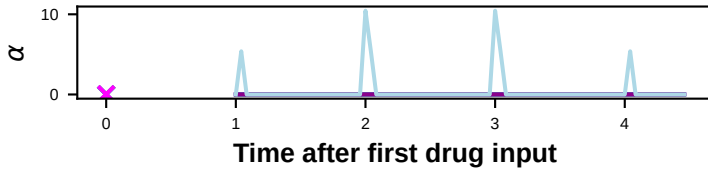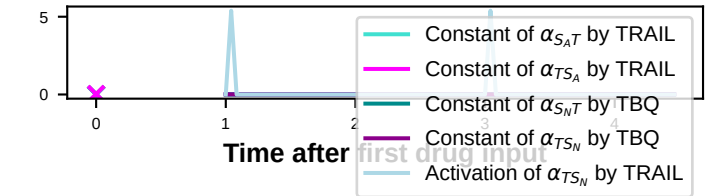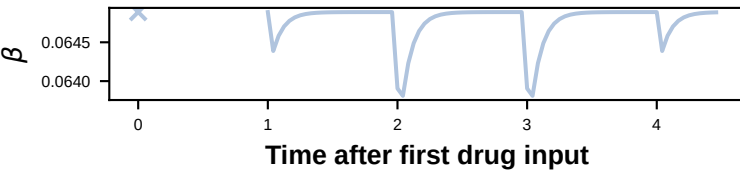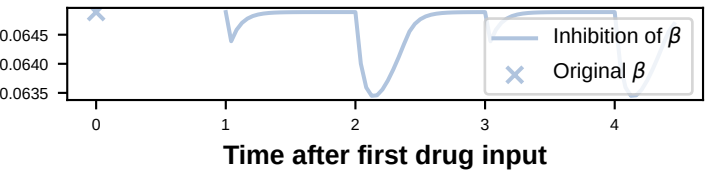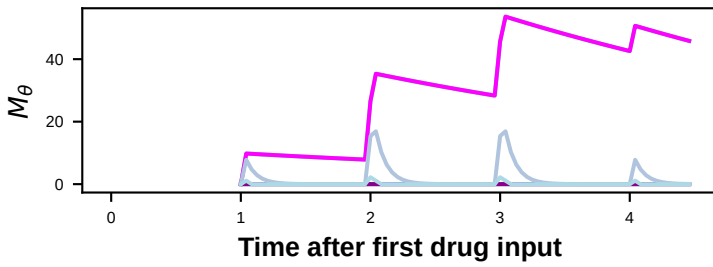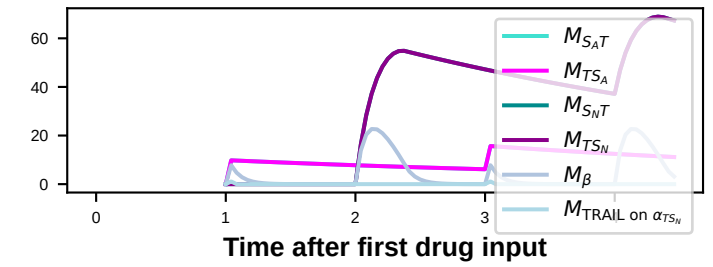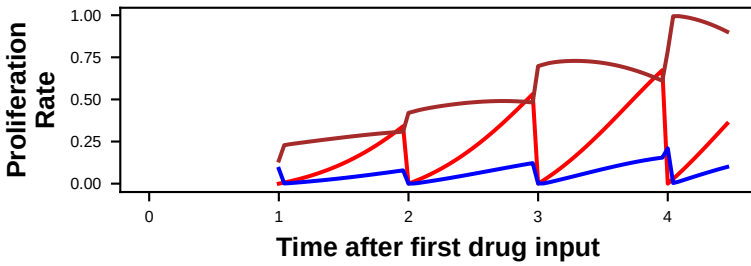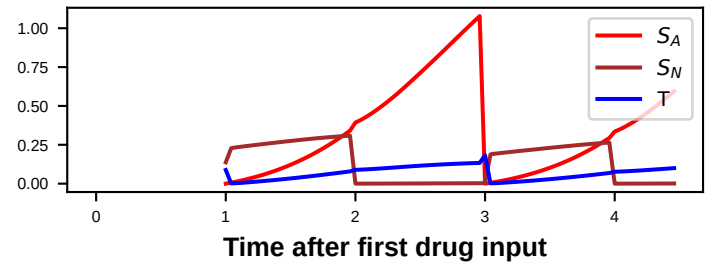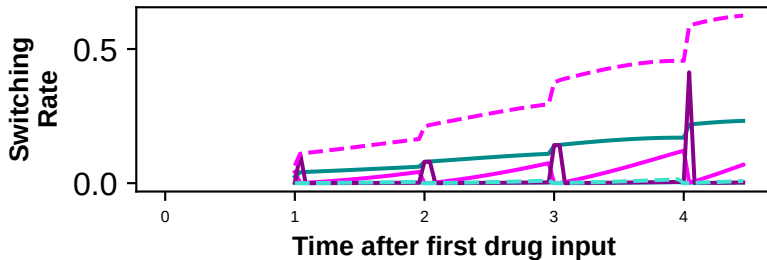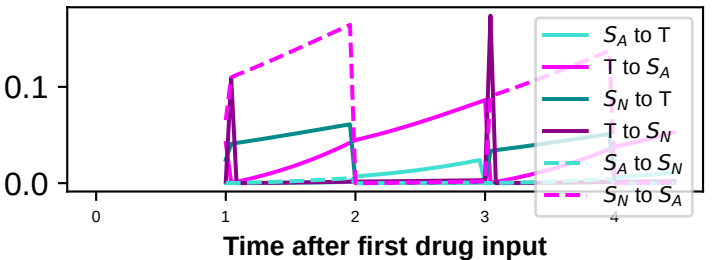

Supplement: Supplementary file 7 — Appendix Simulations Results [file 44320_2025_150_MOESM7_ESM.zip › Appendix_Simulations_Results/PSM2D_Simulations/PSM2_A_4_N_4.pdf]

## Model 8 calibrated for pro-necroptotic treatment

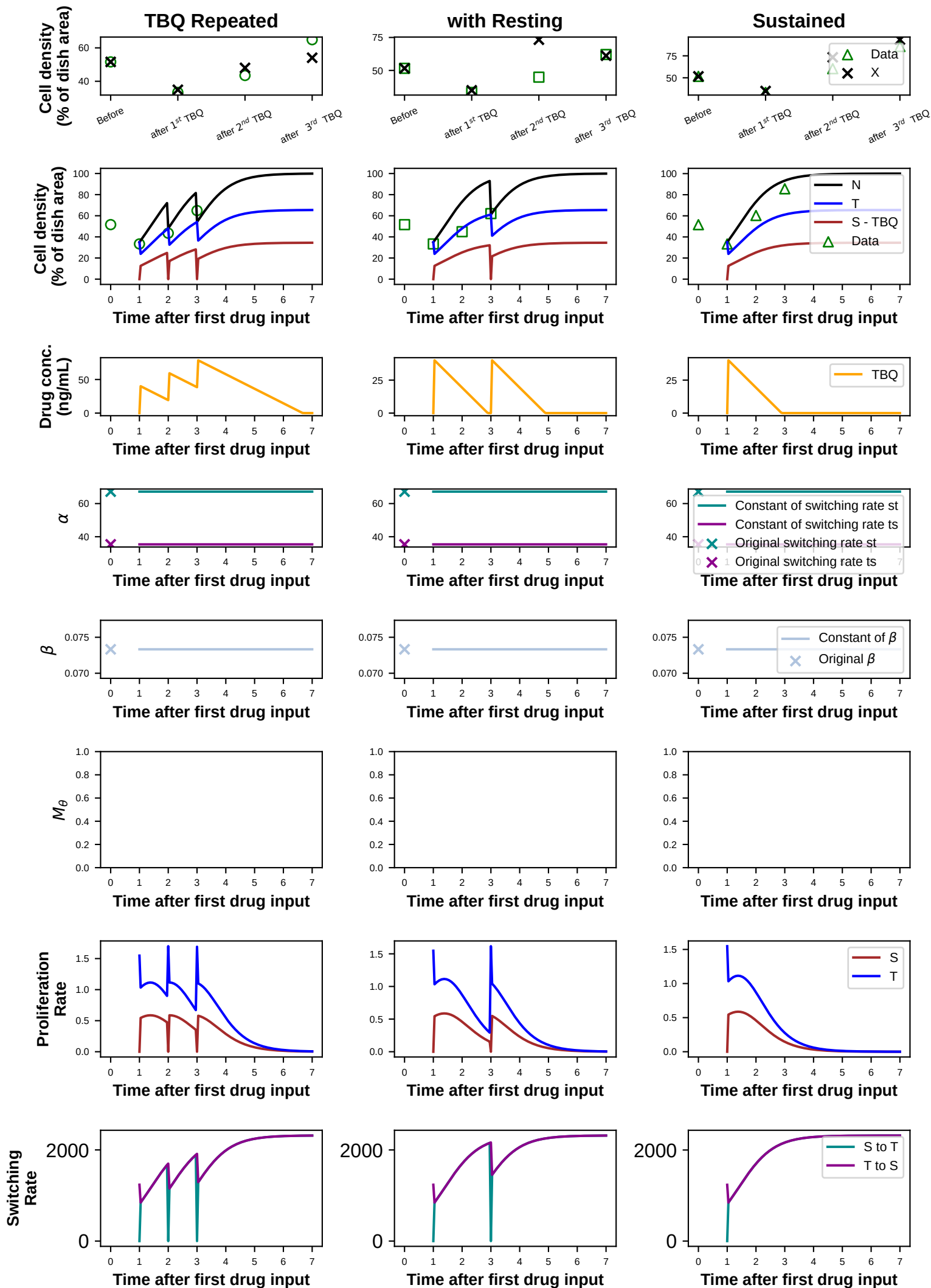

Supplement: Supplementary file 7 — Appendix Simulations Results [file 44320_2025_150_MOESM7_ESM.zip › Appendix_Simulations_Results/PSM1D_Simulations/PSM1_N_8.pdf]

## Model 7 calibrated for pro-apoptotic treatment

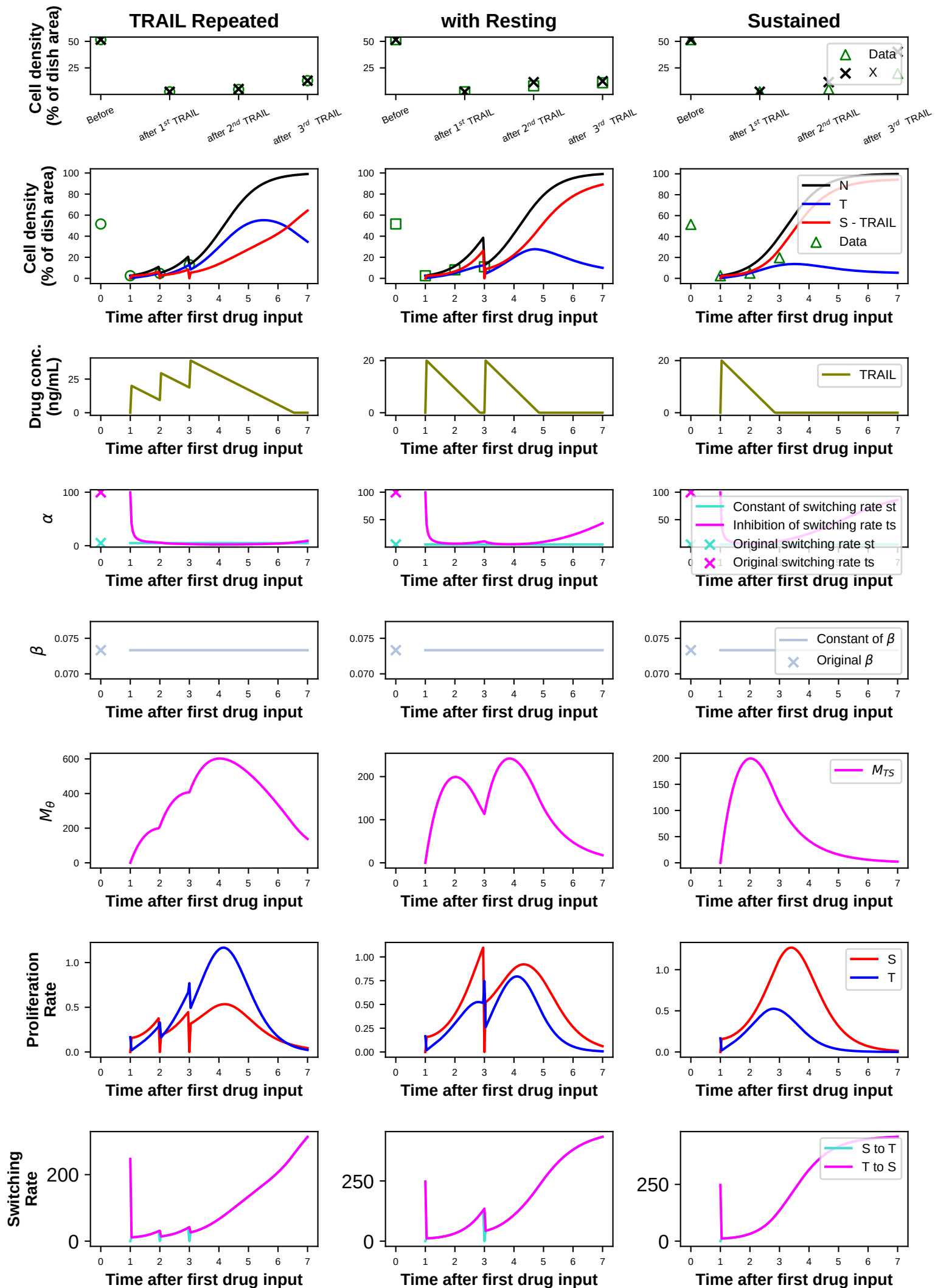

Supplement: Supplementary file 7 — Appendix Simulations Results [file 44320_2025_150_MOESM7_ESM.zip › Appendix_Simulations_Results/PSM1D_Simulations/PSM1_A_7.pdf]

## Model 6 calibrated for pro-apoptotic treatment

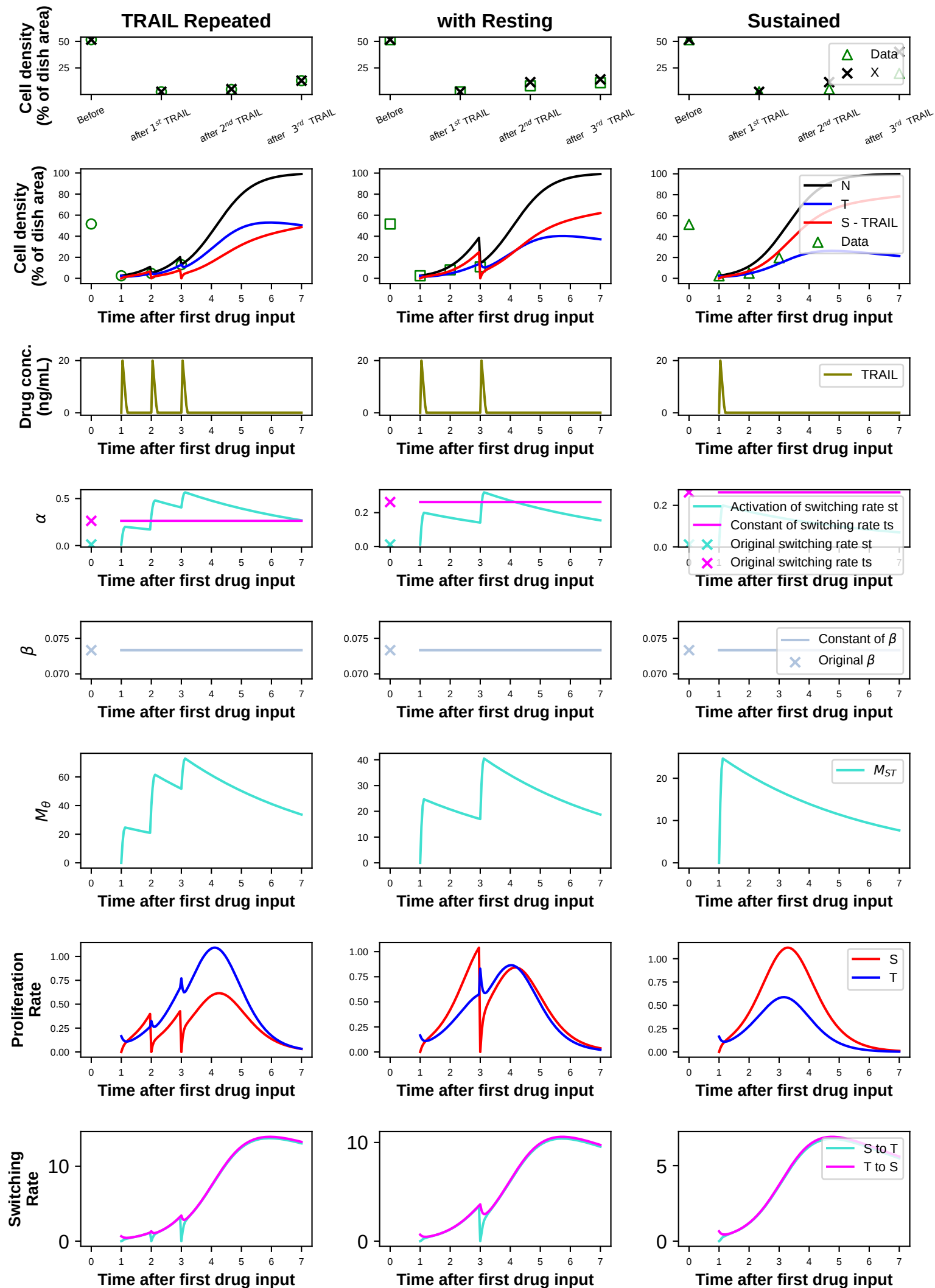

Supplement: Supplementary file 7 — Appendix Simulations Results [file 44320_2025_150_MOESM7_ESM.zip › Appendix_Simulations_Results/PSM1D_Simulations/PSM1_A_6.pdf]

## Model 4 calibrated for pro-apoptotic treatment

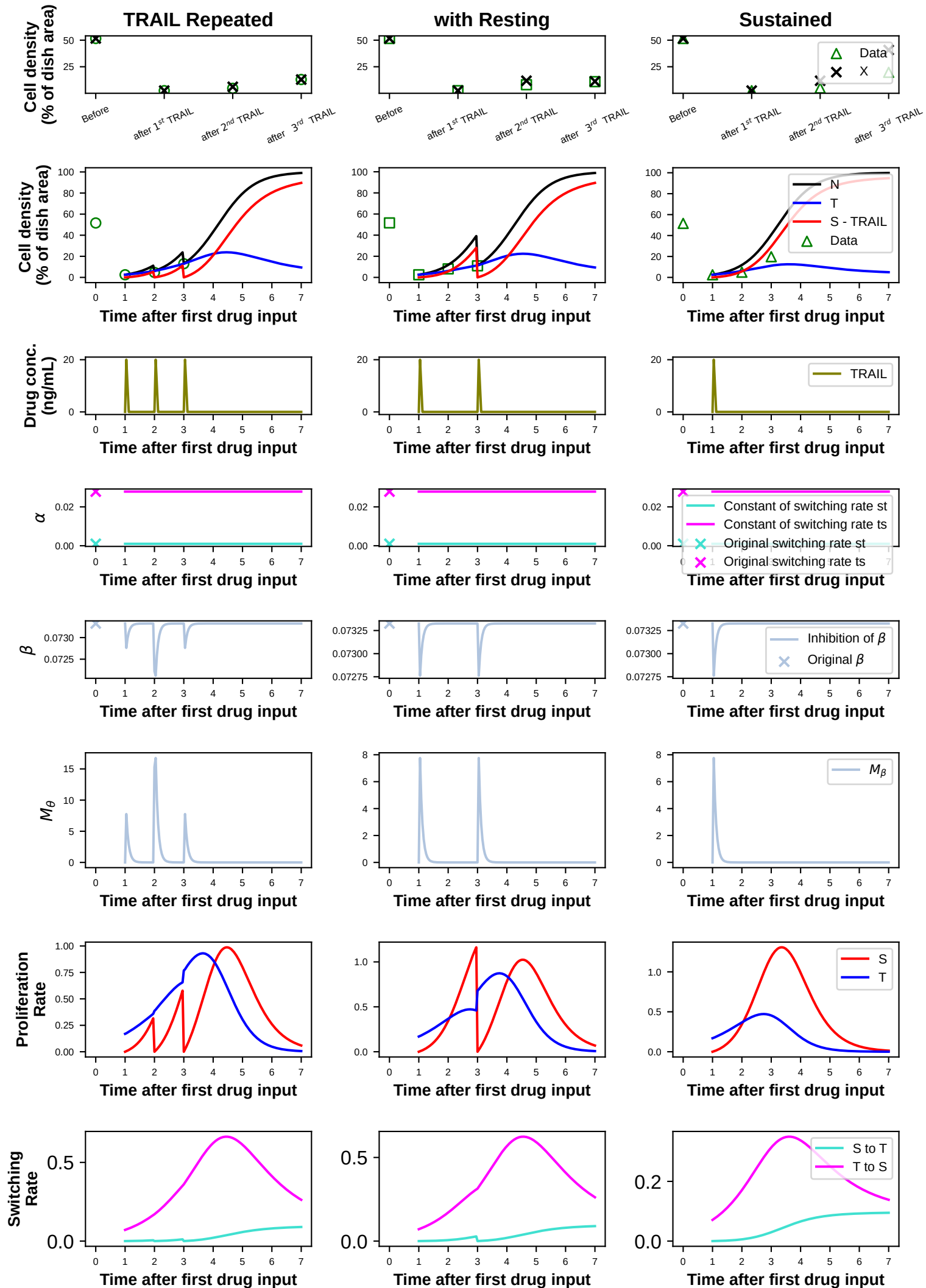

Supplement: Supplementary file 7 — Appendix Simulations Results [file 44320_2025_150_MOESM7_ESM.zip › Appendix_Simulations_Results/PSM1D_Simulations/PSM1_A_4.pdf]

## Model 5 calibrated for pro-apoptotic treatment

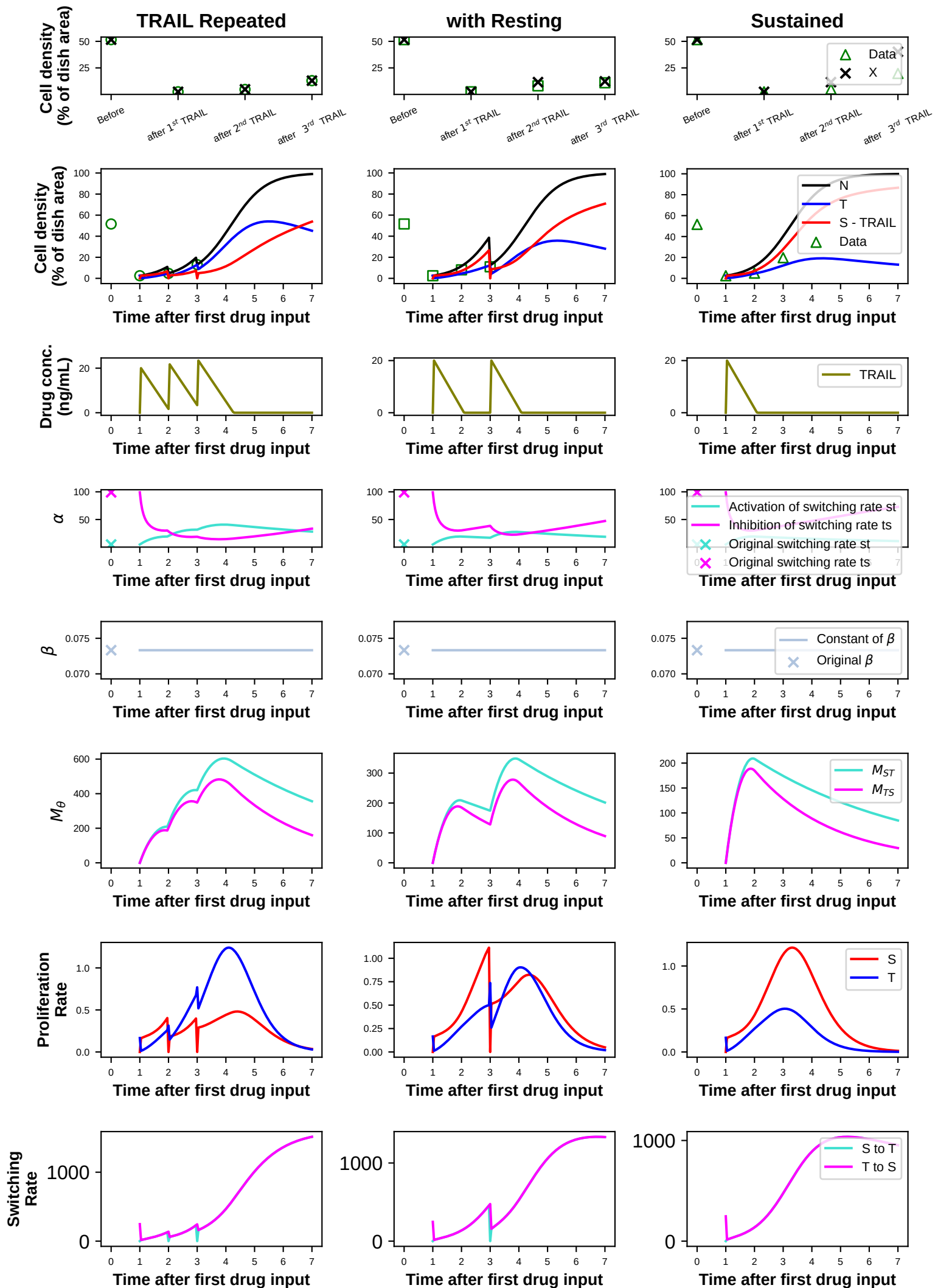

Supplement: Supplementary file 7 — Appendix Simulations Results [file 44320_2025_150_MOESM7_ESM.zip › Appendix_Simulations_Results/PSM1D_Simulations/PSM1_A_5.pdf]

## Model 1 calibrated for pro-apoptotic treatment

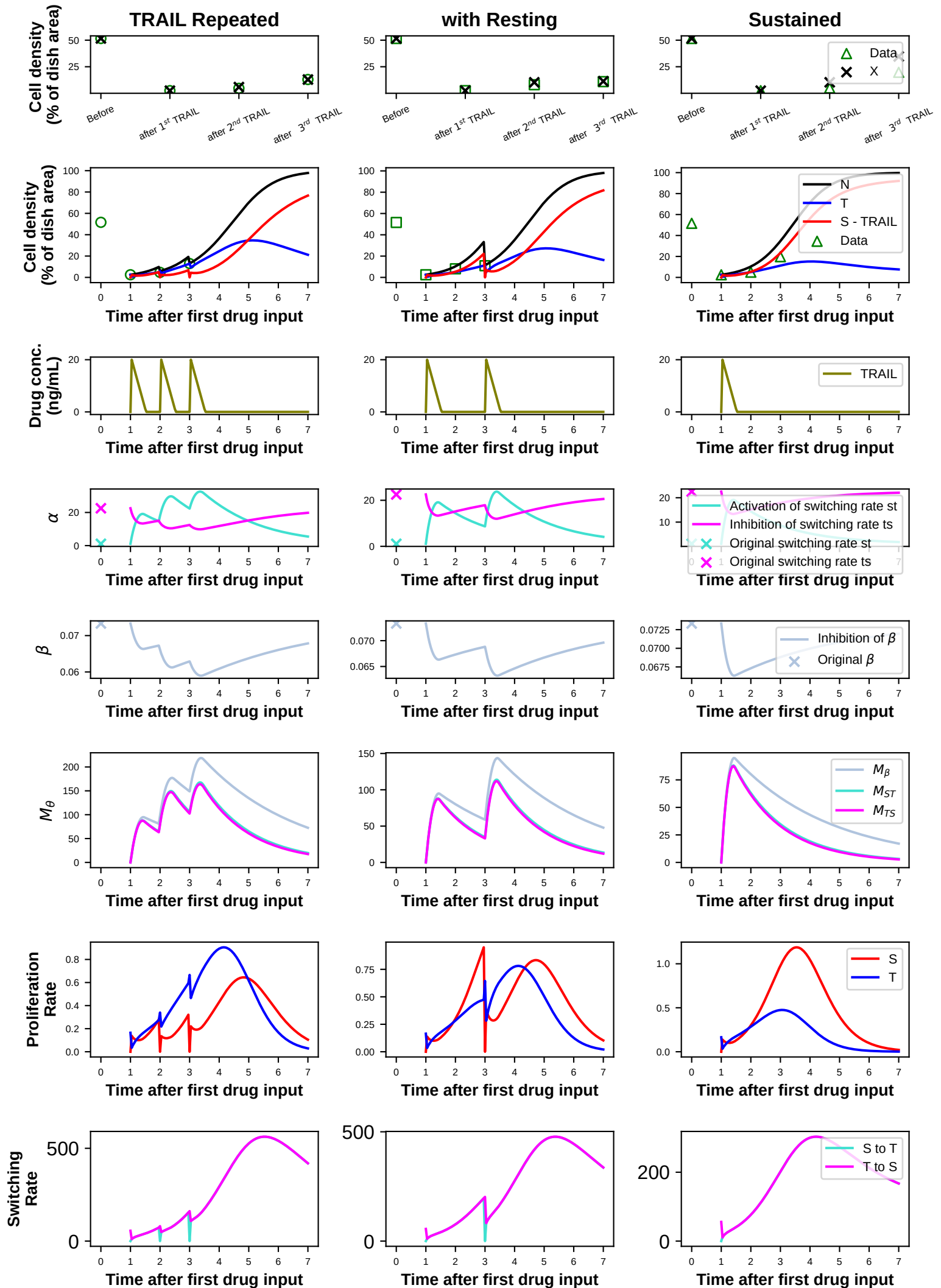

Supplement: Supplementary file 7 — Appendix Simulations Results [file 44320_2025_150_MOESM7_ESM.zip › Appendix_Simulations_Results/PSM1D_Simulations/PSM1_A_1.pdf]

## Model 2 calibrated for pro-apoptotic treatment

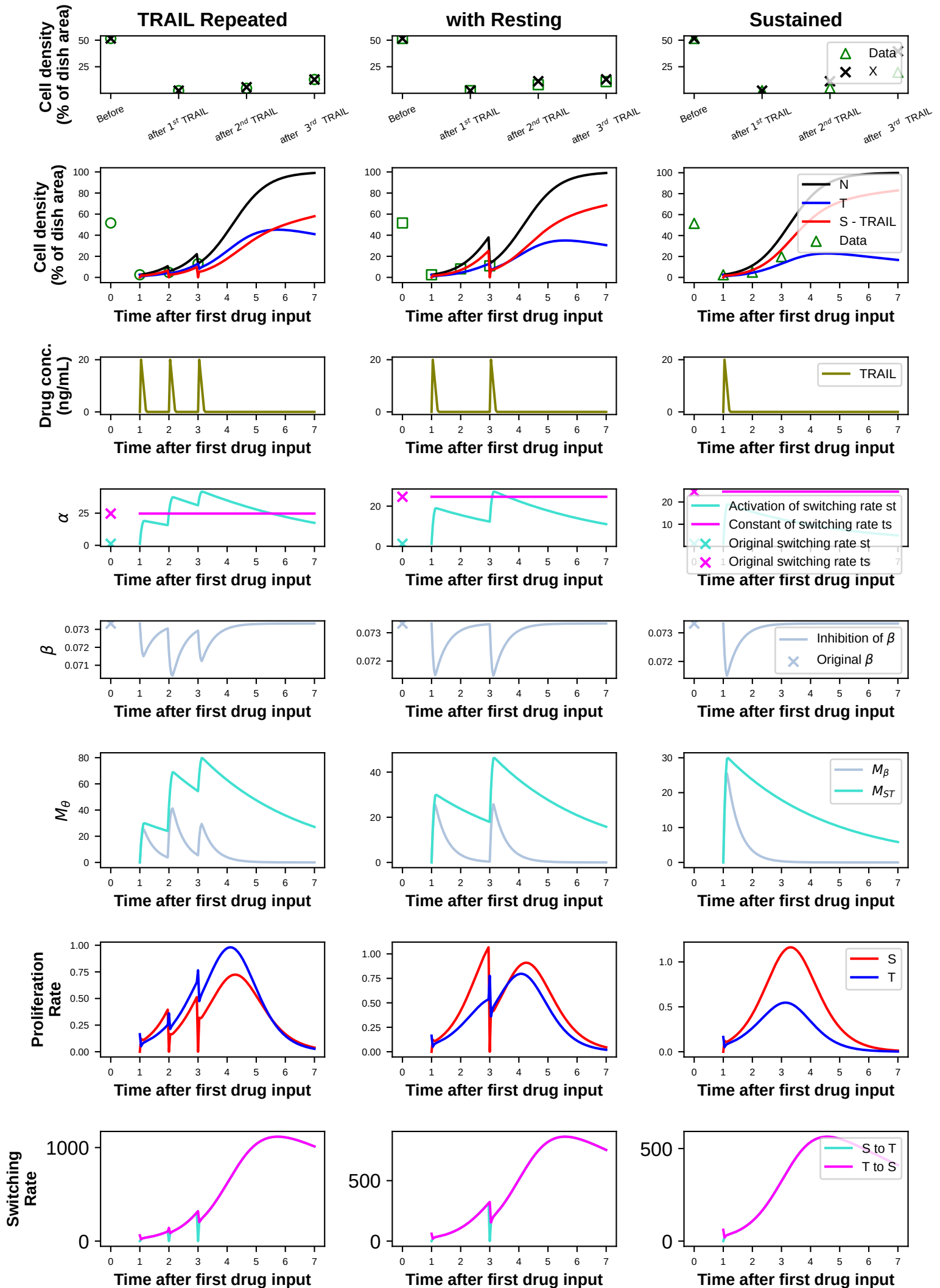

Supplement: Supplementary file 7 — Appendix Simulations Results [file 44320_2025_150_MOESM7_ESM.zip › Appendix_Simulations_Results/PSM1D_Simulations/PSM1_A_2.pdf]

## Model 3 calibrated for pro-apoptotic treatment

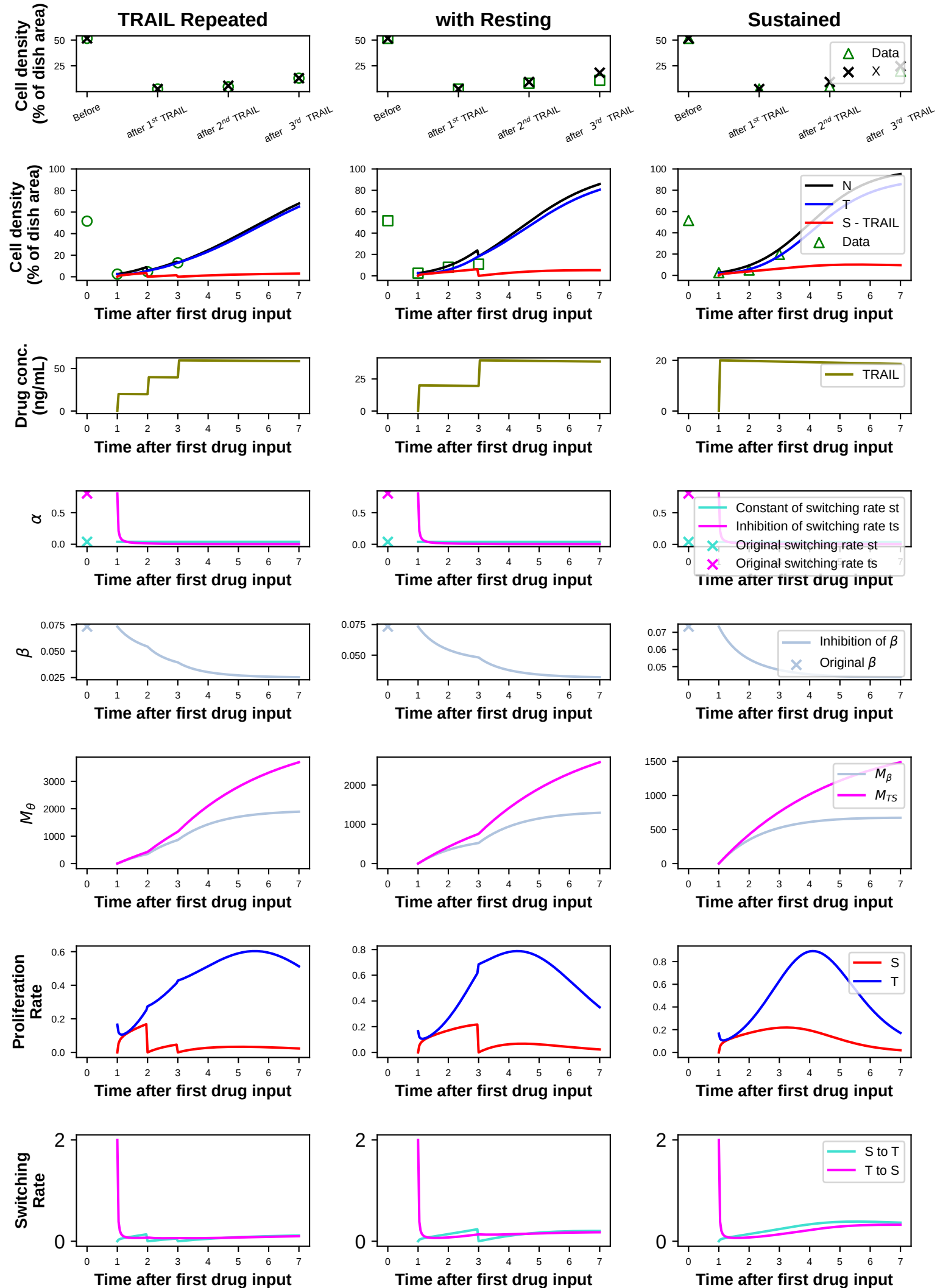

Supplement: Supplementary file 7 — Appendix Simulations Results [file 44320_2025_150_MOESM7_ESM.zip › Appendix_Simulations_Results/PSM1D_Simulations/PSM1_A_3.pdf]

# Model 8 calibrated for pro-apoptotic treatment

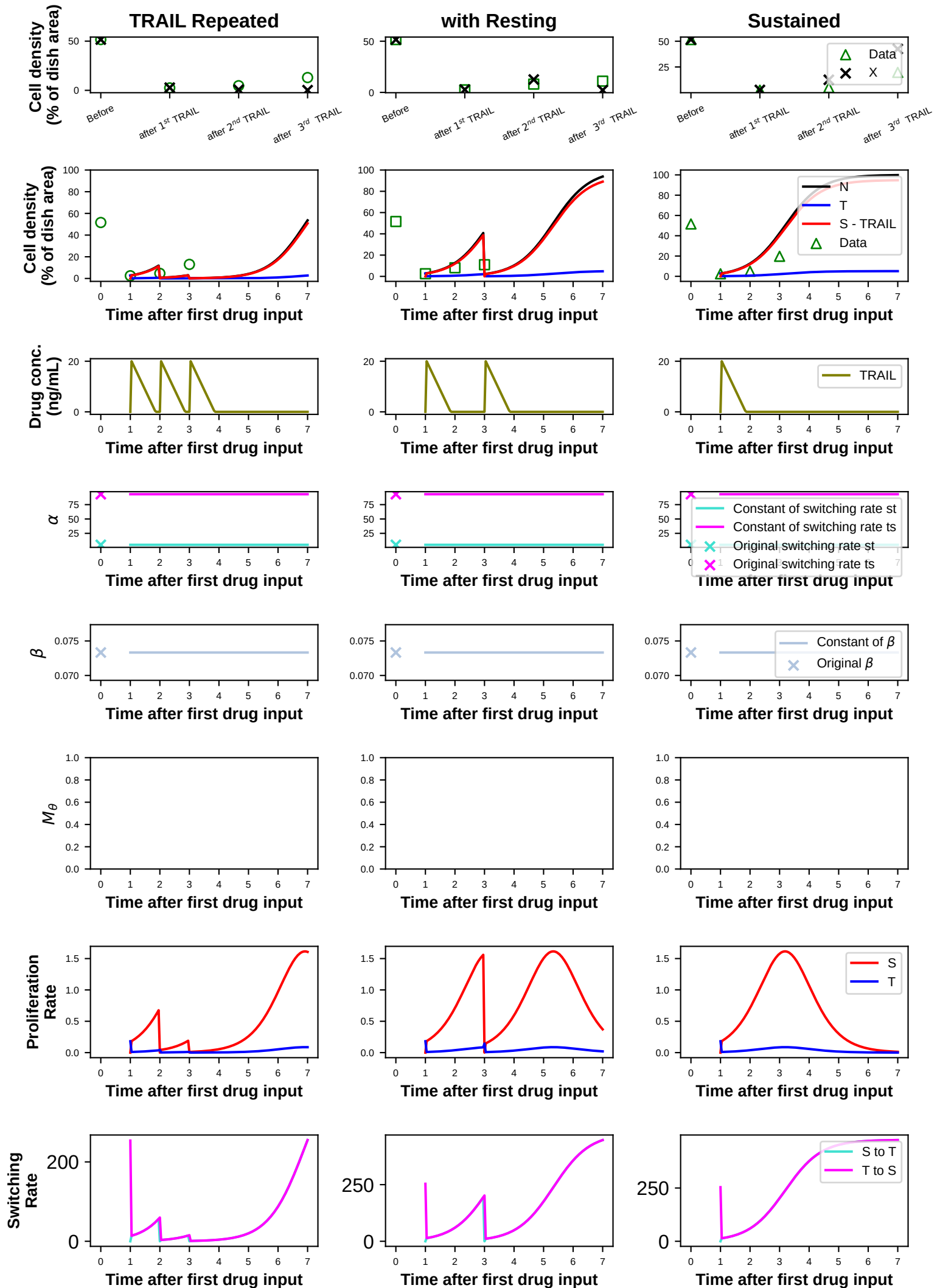

Supplement: Supplementary file 7 — Appendix Simulations Results [file 44320_2025_150_MOESM7_ESM.zip › Appendix_Simulations_Results/PSM1D_Simulations/PSM1_A_8.pdf]

## Model 2 calibrated for pro-necroptotic treatment

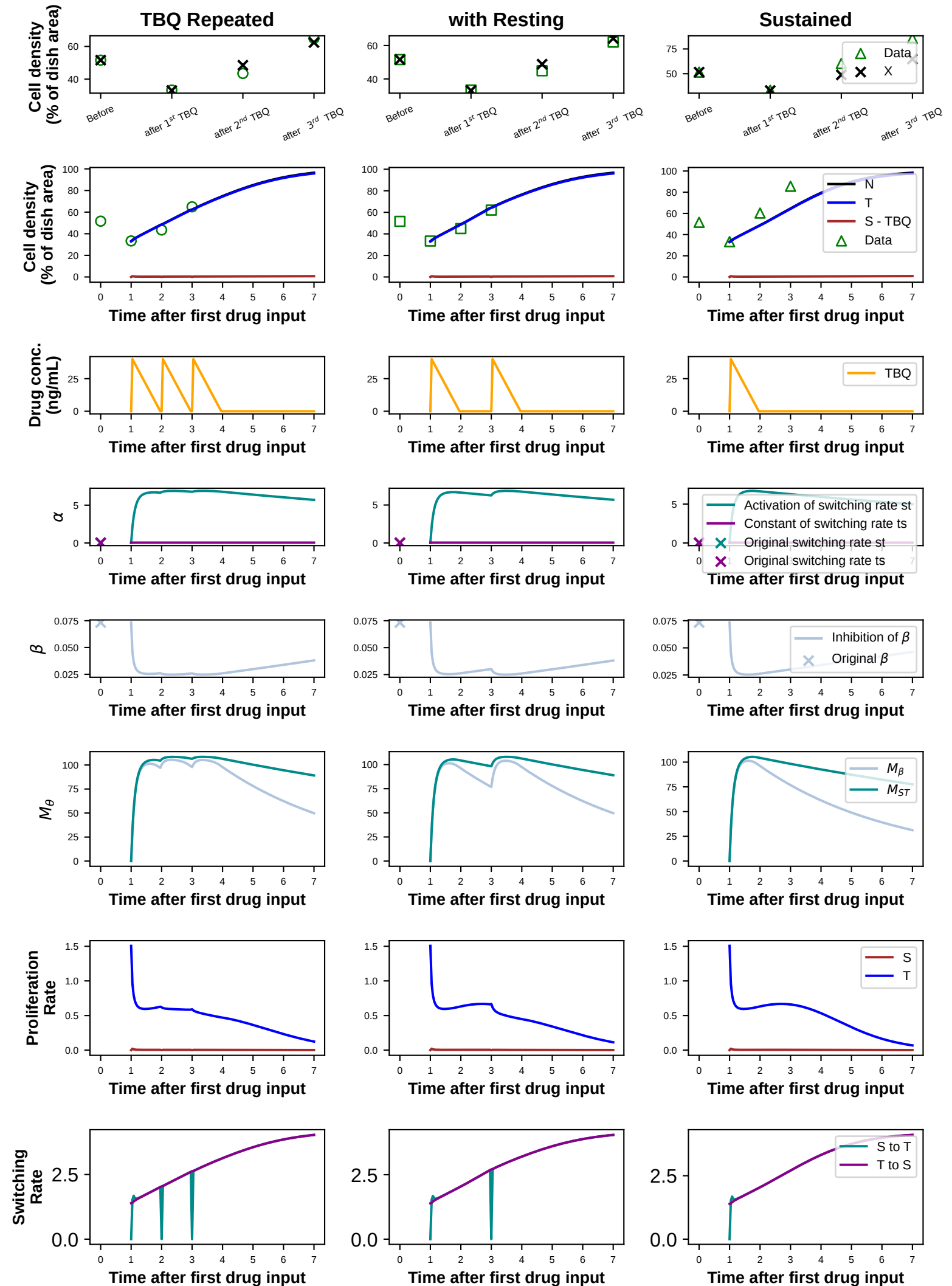

Supplement: Supplementary file 7 — Appendix Simulations Results [file 44320_2025_150_MOESM7_ESM.zip › Appendix_Simulations_Results/PSM1D_Simulations/PSM1_N_2.pdf]

# Model 3 calibrated for pro-necroptotic treatment

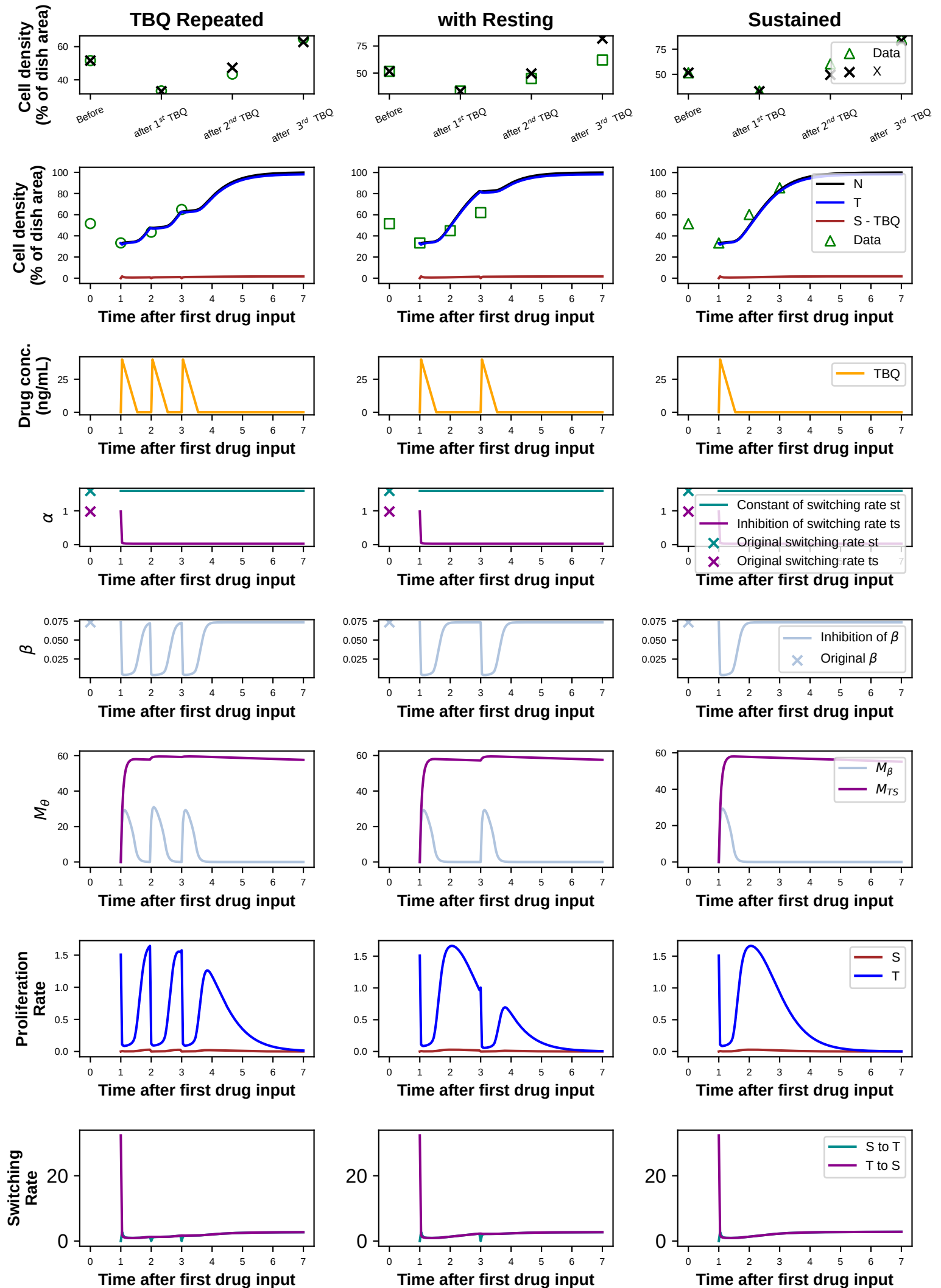

Supplement: Supplementary file 7 — Appendix Simulations Results [file 44320_2025_150_MOESM7_ESM.zip › Appendix_Simulations_Results/PSM1D_Simulations/PSM1_N_3.pdf]

# Model 1 calibrated for pro-necroptotic treatment

## TBQ Repeated

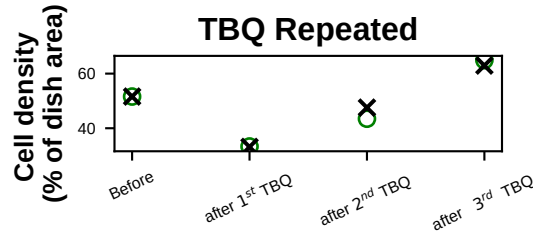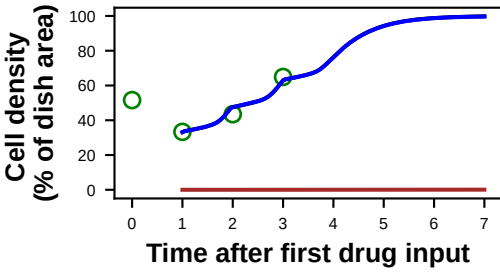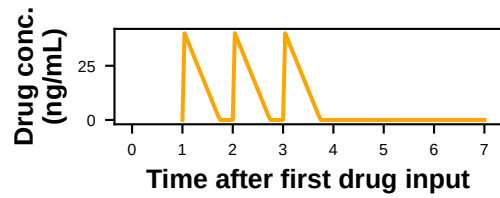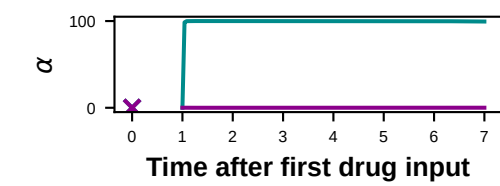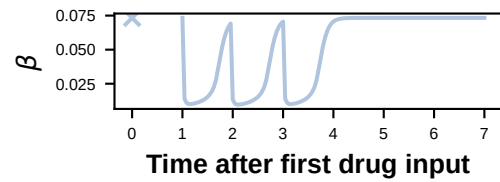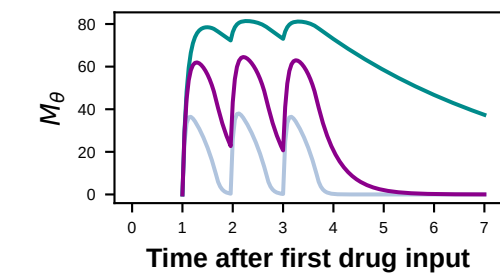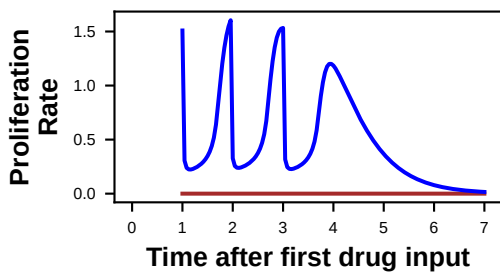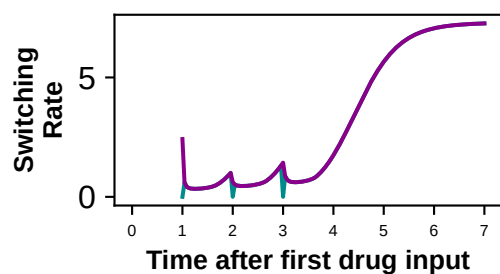

## with Resting

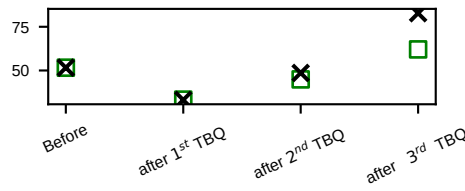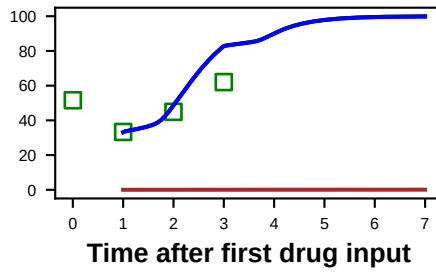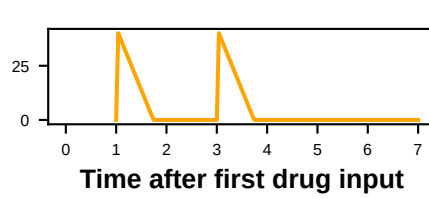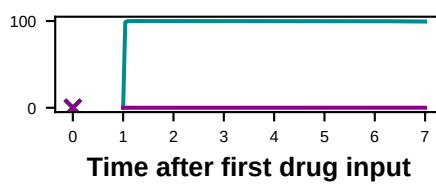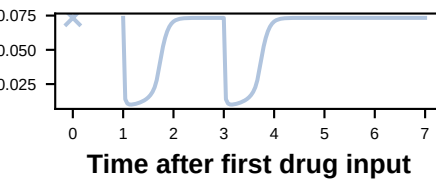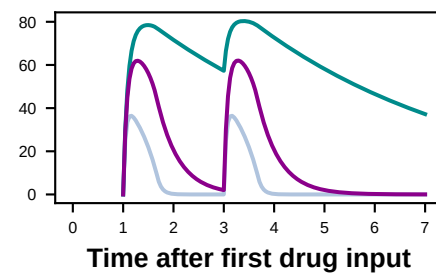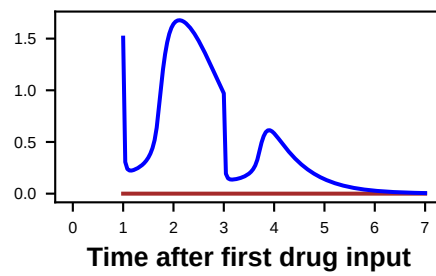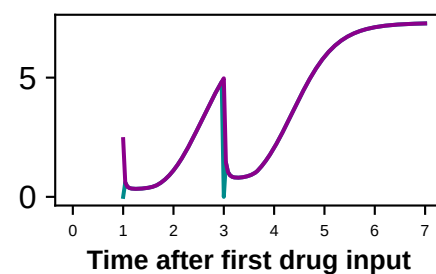

## Sustained

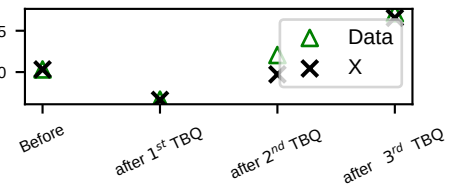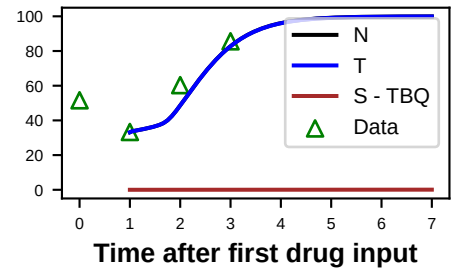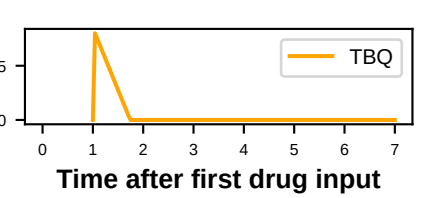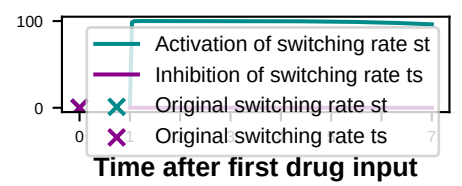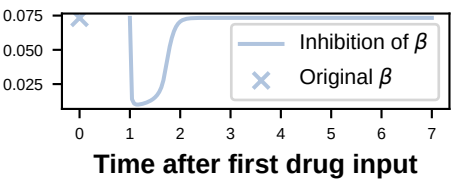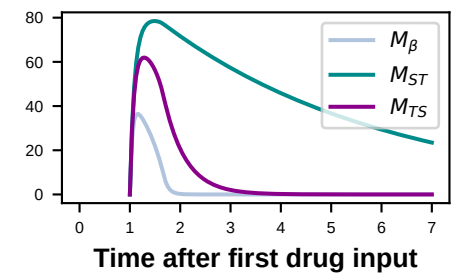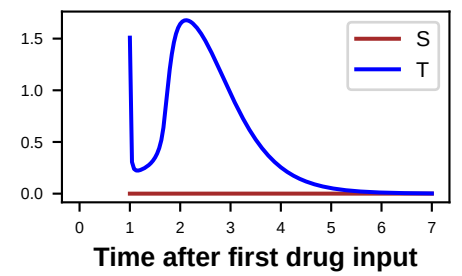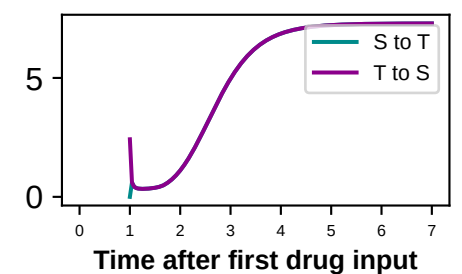

Supplement: Supplementary file 7 — Appendix Simulations Results [file 44320_2025_150_MOESM7_ESM.zip › Appendix_Simulations_Results/PSM1D_Simulations/PSM1_N_1.pdf]

## Model 4 calibrated for pro-necroptotic treatment

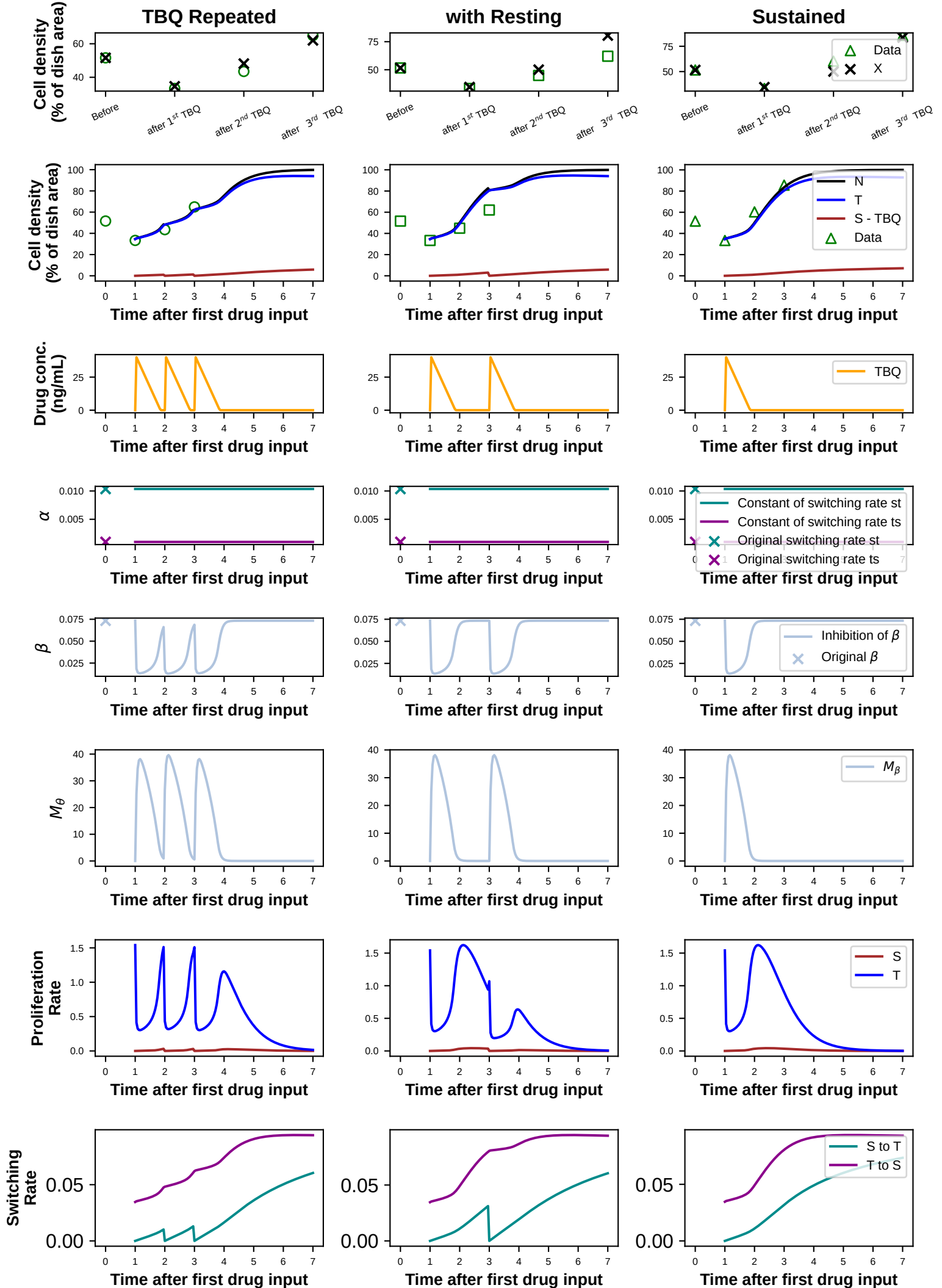

Supplement: Supplementary file 7 — Appendix Simulations Results [file 44320_2025_150_MOESM7_ESM.zip › Appendix_Simulations_Results/PSM1D_Simulations/PSM1_N_4.pdf]

# Model 5 calibrated for pro-necroptotic treatment

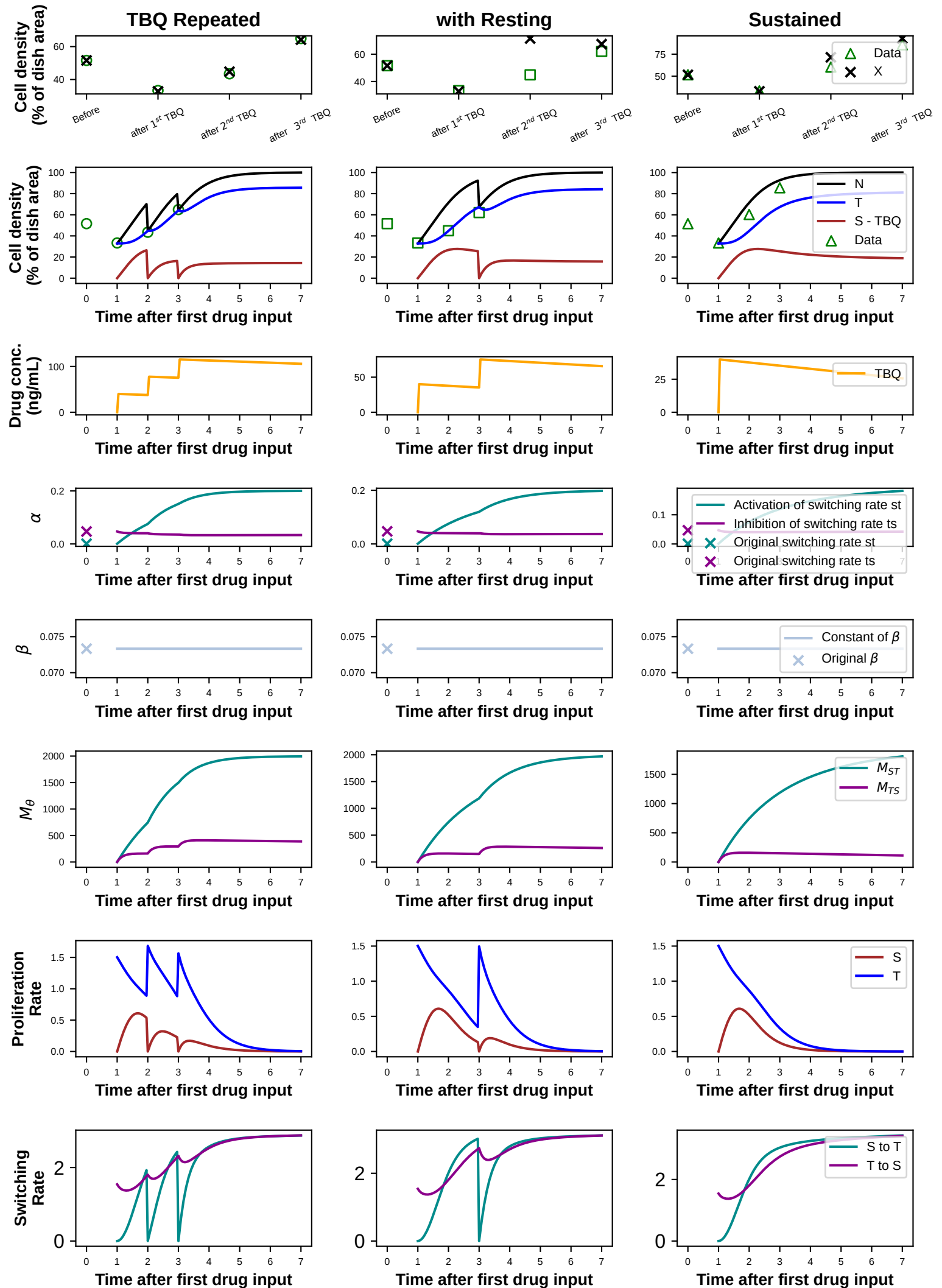

Supplement: Supplementary file 7 — Appendix Simulations Results [file 44320_2025_150_MOESM7_ESM.zip › Appendix_Simulations_Results/PSM1D_Simulations/PSM1_N_5.pdf]

# Model 7 calibrated for pro-necroptotic treatment

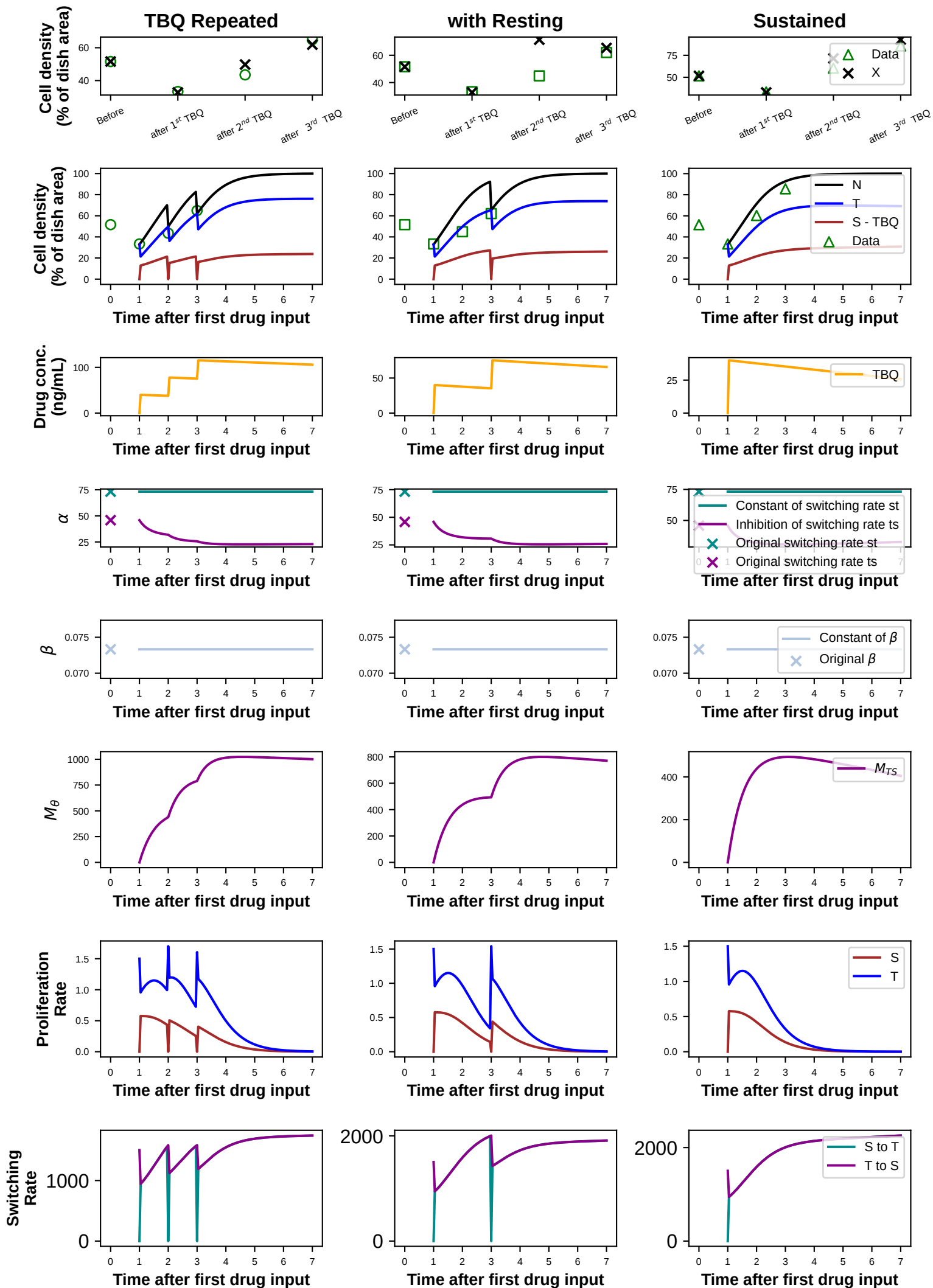

Supplement: Supplementary file 7 — Appendix Simulations Results [file 44320_2025_150_MOESM7_ESM.zip › Appendix_Simulations_Results/PSM1D_Simulations/PSM1_N_7.pdf]

# Model 6 calibrated for pro-necroptotic treatment

## TBQ Repeated

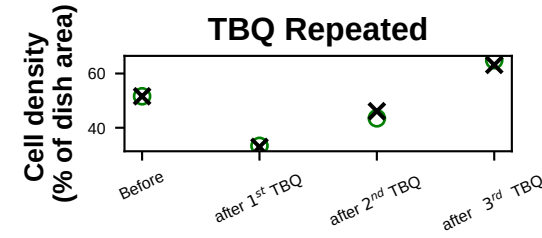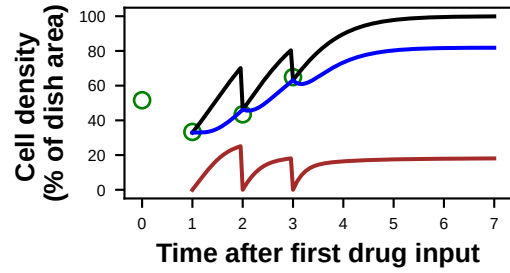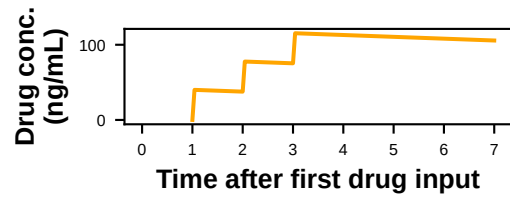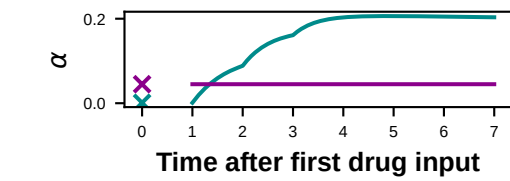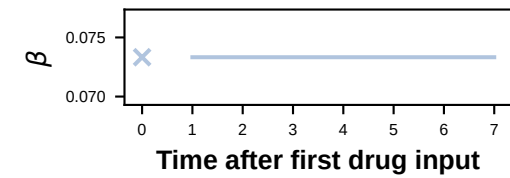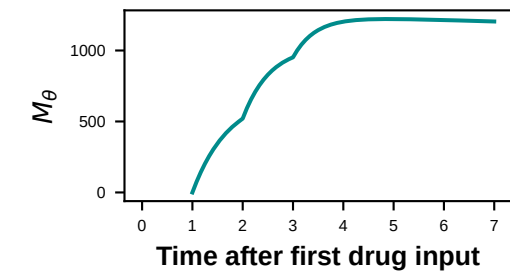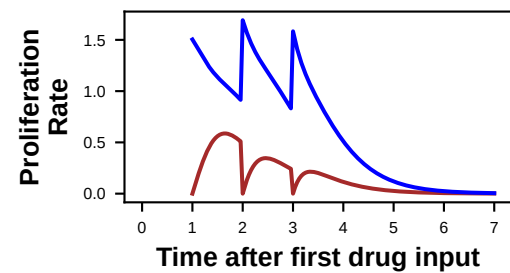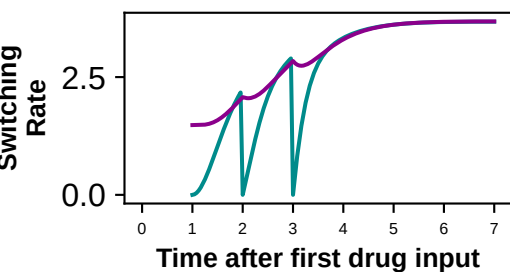

## with Resting

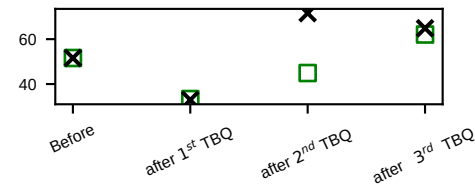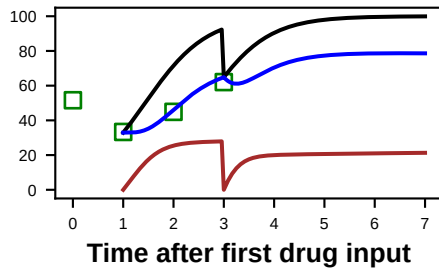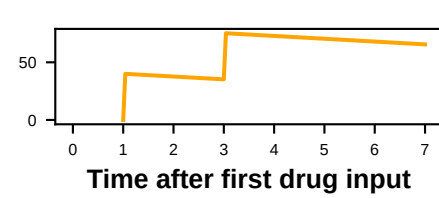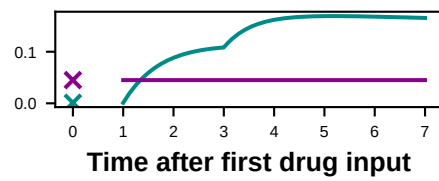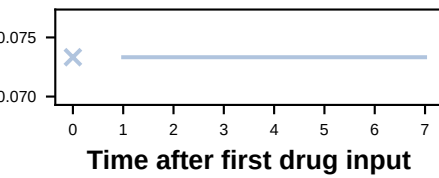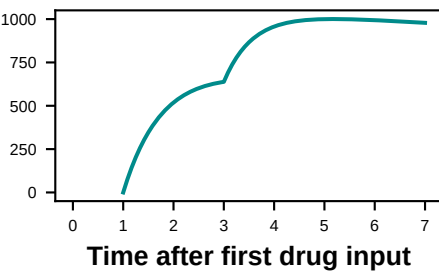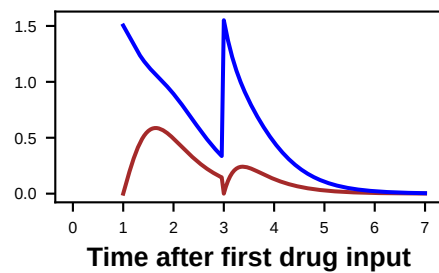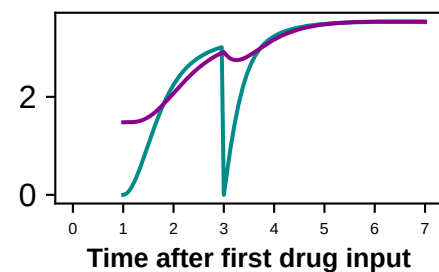

## Sustained

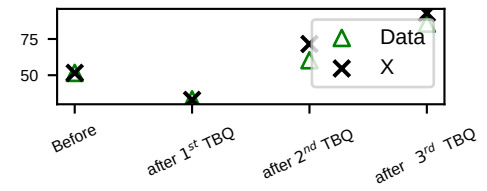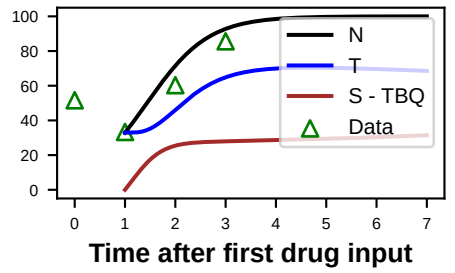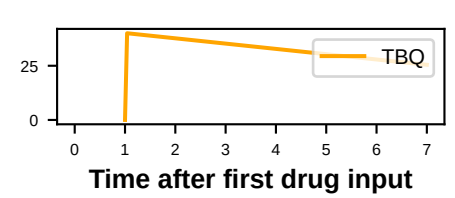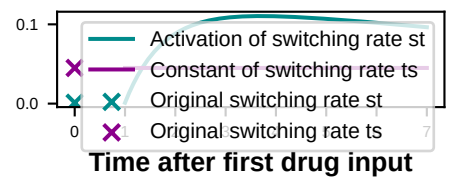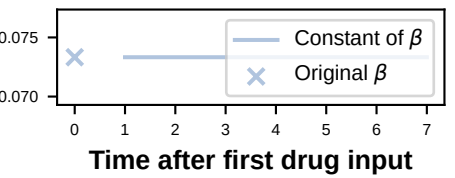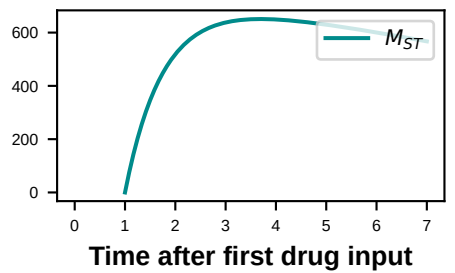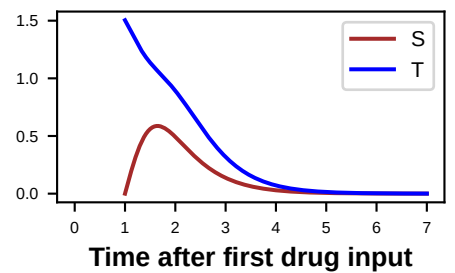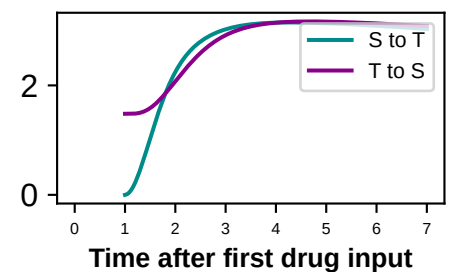

Supplement: Supplementary file 7 — Appendix Simulations Results [file 44320_2025_150_MOESM7_ESM.zip › Appendix_Simulations_Results/PSM1D_Simulations/PSM1_N_6.pdf]
