# Supplementary material for: Transition between cell states of sensitivity reveals molecular vulnerability of drug-tolerant cells
Source: Mol Syst Biol. 2025 Oct 1;21(12):4. doi: 10.1038/s44320-025-00150-0 (PMC12673137; doi:10.1038/s44320-025-00150-0)
Supplement: Supplementary file 8 — Expanded View Figures [file 44320_2025_150_MOESM8_ESM.pdf]

Expanded View Figures

Figure EV1. TRAIL-induced apoptosis and necroptosis (TBQ, TCQ) treatments give rise to drug-tolerant persister cells in HT-29 and HeLa-RIPK3 cell lines.

(Left panels: **A**, **C**, **E**, **G**) Cell density measured as fraction of dish area occupied by HT-29 or HeLa-RIPK3 cells after repeated TRAIL, TBQ, or TCQ treatments (blue dots) or treatment followed by a drug removal ("Resting", red dots) or treatment left in the cell medium for the time of the experiment ("Sustained", green dots), corresponding experimental repeats are shown in the right panels (**B**, **D**, **F**, **H**) (same treatments). (Right panels: **B**, **D**, **F**, **H**) Change in cell density of the population labeled inside each bar, caused by the treatment indicated on the side of each bar, measured as a difference in density before and after treatment. Each bar is an average change in cell density for at least three experimental repeats, data were represented as mean ± SEM, with a $^{**}p$ value of 0.0091 and a $^{***}p$ value of 0.0010 ((**B**), Student's *t*-test), with $^{***}p$ values of 3.2e-06 and 3.1e-05 downward correspondingly ((**D**), Student's *t*-test), with $^{***}p$ values of 3.6e-07 and 1.9e-06 downward correspondingly ((**F**), Student's *t*-test), and with $^{***}p$ values of 7.5e-07, 1.5e-07, 1.5e-09, and 1.7e-06 downward correspondingly ((**H**), Student's *t*-test).

 © The Author(s)

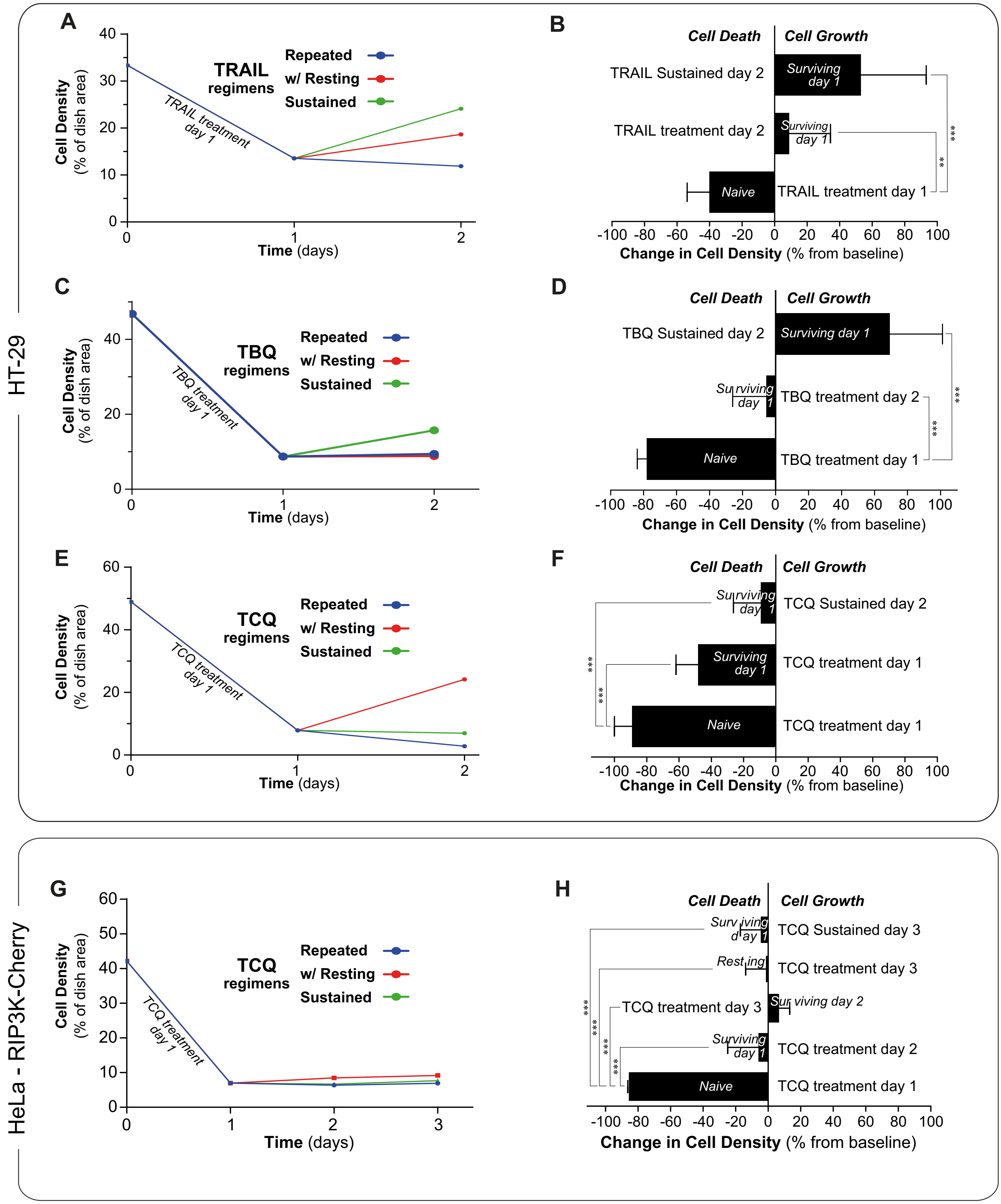
HT-29
A
TRAIL regimens
Repeated
w/ Resting
Sustained
TRAIL treatment day 1
Cell Density (% of dish area)
Time (days)
B
Cell Death
Cell Growth
TRAIL Sustained day 2
TRAIL treatment day 2
TRAIL treatment day 1
Surviving day 1
Naive
Change in Cell Density (% from baseline)
C
TBQ regimens
TBQ treatment day 1
D
TBQ Sustained day 2
TBQ treatment day 2
TBQ treatment day 1
E
TCQ regimens
TCQ treatment day 1
F
TCQ Sustained day 2
TCQ treatment day 1
HeLa - RIP3K-Cherry
G
H
TCQ Sustained day 3
TCQ treatment day 3
Resting
Surviving day 2
TCQ treatment day 2
TCQ treatment day 1

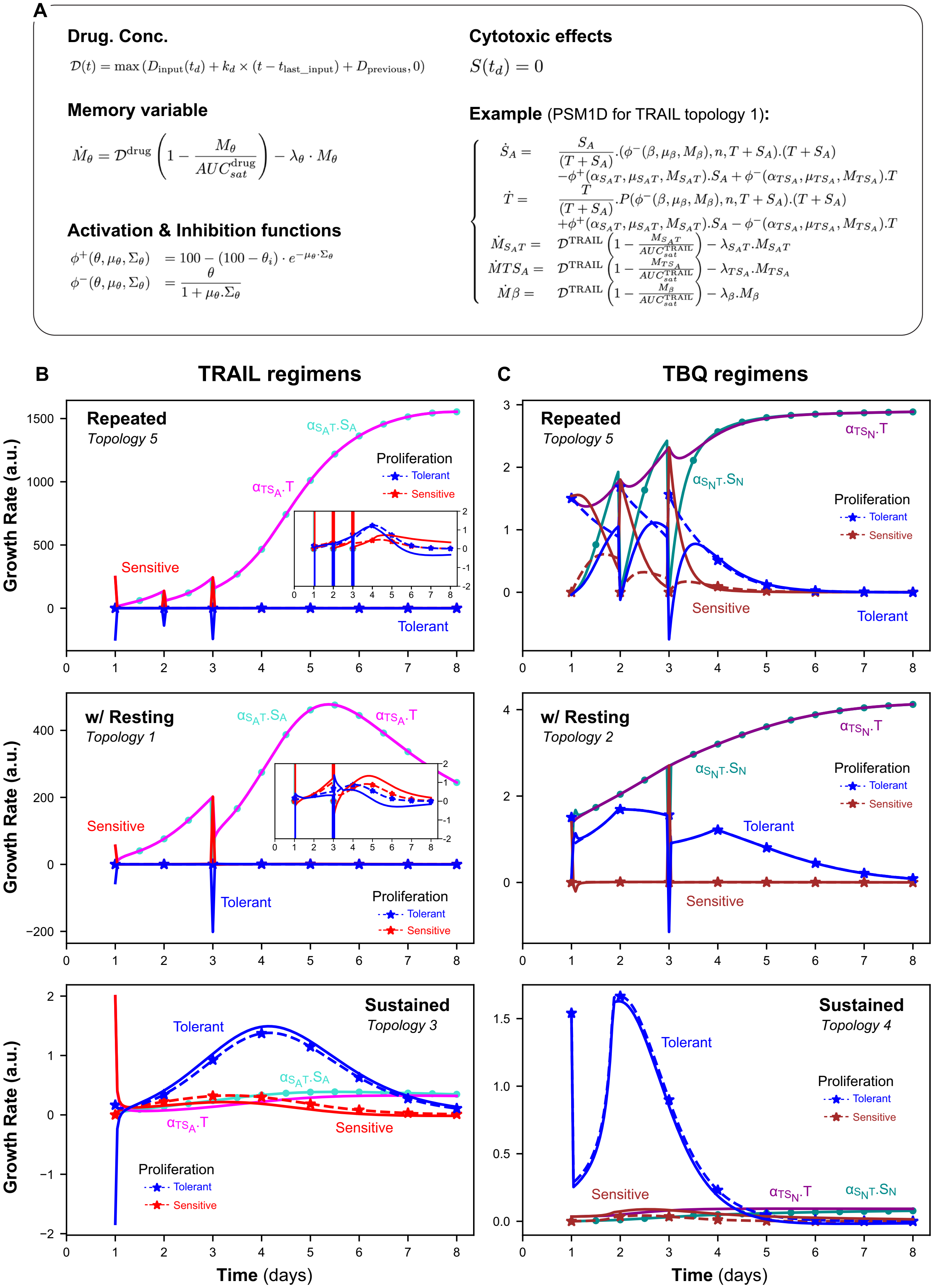
A
Drug. Conc.
$\mathcal{D}(t) = \max\left(D_{\text{input}}(t_d) + k_d \times (t - t_{\text{last\_input}}) + D_{\text{previous}}, 0\right)$
Memory variable
$\dot{M}_\theta = \mathcal{D}^{\text{drug}}\left(1 - \frac{M_\theta}{AUC_{sat}^{\text{drug}}}\right) - \lambda_\theta \cdot M_\theta$
Activation & Inhibition functions
$\phi^+(\theta, \mu_\theta, \Sigma_\theta) = 100 - (100 - \theta_i) \cdot e^{-\mu_\theta \cdot \Sigma_\theta}$
$\phi^-(\theta, \mu_\theta, \Sigma_\theta) = \frac{\theta}{1 + \mu_\theta . \Sigma_\theta}$
Cytotoxic effects
$S(t_d) = 0$
Example (PSM1D for TRAIL topology 1):
$\dot{S}_A = \frac{S_A}{(T+S_A)}.(\phi^-(\beta, \mu_\beta, M_\beta), n, T+S_A).(T+S_A) - \phi^+(\alpha_{S_AT}, \mu_{S_AT}, M_{S_AT}).S_A + \phi^-(\alpha_{TS_A}, \mu_{TS_A}, M_{TS_A}).T$
$\dot{T} = \frac{T}{(T+S_A)}.P(\phi^-(\beta, \mu_\beta, M_\beta), n, T+S_A).(T+S_A) + \phi^+(\alpha_{S_AT}, \mu_{S_AT}, M_{S_AT}).S_A - \phi^-(\alpha_{TS_A}, \mu_{TS_A}, M_{TS_A}).T$
$\dot{M}_{S_AT} = \mathcal{D}^{\text{TRAIL}}\left(1 - \frac{M_{S_AT}}{AUC_{sat}^{\text{TRAIL}}}\right) - \lambda_{S_AT}.M_{S_AT}$
$\dot{M}TS_A = \mathcal{D}^{\text{TRAIL}}\left(1 - \frac{M_{TS_A}}{AUC_{sat}^{\text{TRAIL}}}\right) - \lambda_{TS_A}.M_{TS_A}$
$\dot{M}\beta = \mathcal{D}^{\text{TRAIL}}\left(1 - \frac{M_\beta}{AUC_{sat}^{\text{TRAIL}}}\right) - \lambda_\beta.M_\beta$
B
TRAIL regimens
C
TBQ regimens
Repeated
Topology 5
w/ Resting
Topology 1
Topology 2
Sustained
Topology 3
Topology 4
Growth Rate (a.u.)
Time (days)
Proliferation
Tolerant
Sensitive
$\alpha_{S_AT}.S_A$
$\alpha_{TS_A}.T$
$\alpha_{TS_N}.T$
$\alpha_{S_NT}.S_N$

© The Author(s)

Figure EV2. Phenotypic switch drives growth rate of tolerant population in pro-apoptotic treatments.

(**A**) Equations used to model drug concentration *D* over time (Drug Conc. in Fig. 2), drug long-term retention (Memory variables *M*), activation and inhibition of switching and proliferation parameters by the drug and an example of PSM1D model topology. (**B**, **C**) Contributions of cell proliferation to net growth rate were obtained by computing the product of cell renewal and global proliferation in Eq. (1), using PSM1D solutions from Fig. 2. Phenotypic switch contributions correspond to the phenotypic switch part of Eq. (1). Red lines (TRAIL), Dark red (TBQ): total net growth rate for sensitive population. Blue lines: total net growth rate for the tolerant population. Dashed red lines (TRAIL), dashed dark red lines (TBQ) with stars: Proliferation contribution to growth rate for sensitive. Dashed blue lines with stars: Proliferation contribution to growth rate for sensitive cells. Pink lines (TRAIL), Purple lines (TBQ): Contribution of phenotypic switch from T to S to net growth rate. Dotted turquoise lines (TRAIL), Dark turquoise lines (TBQ): Contribution of phenotypic switch from S to T to net growth rate. Subplots for "Repeated" and "Resting" in panel (**A**) are zoomed versions around 0 of the growth rate dynamics.

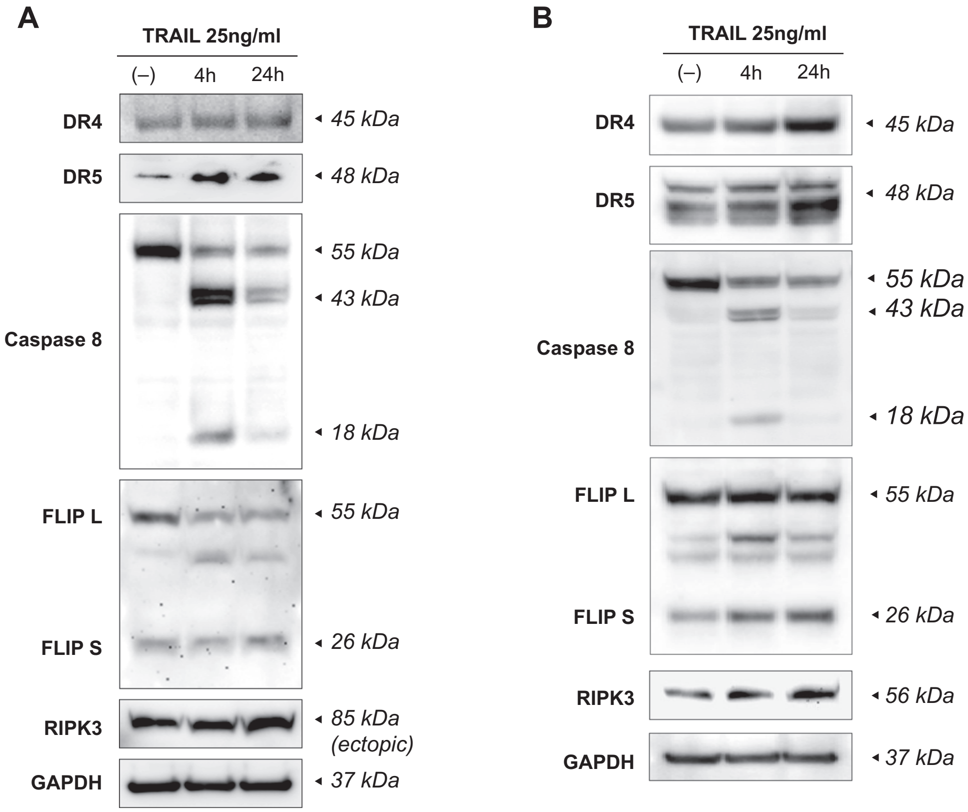

Figure EV3. TRAIL-induced changes in protein expressions in HeLa-RIPK3 and HT-29 cell lines.

Protein expressions of death receptors 4 and 5 (DR4, DR5), Caspase-8, FLIP-L and -S, RIPK3, and GAPDH in control condition (−) and after TRAIL treatments (25 ng/mL, 4 and 24 h), measured by Western blot analyses in HeLa-RIPK3 (**A**) and in HT-29 cells (**B**). Two representative Western blot experiments are shown.

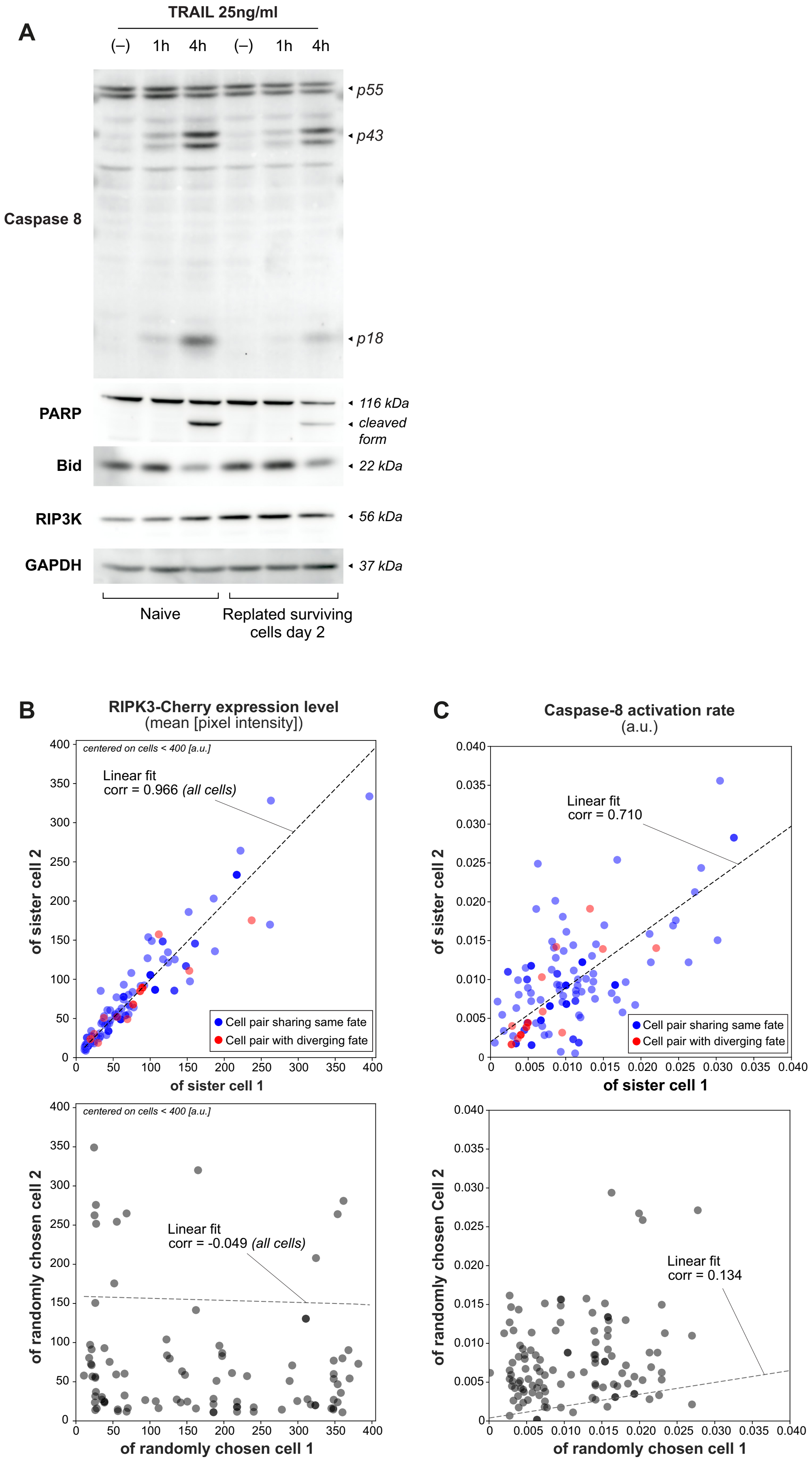
A
TRAIL 25ng/ml
(–) 1h 4h (–) 1h 4h
Caspase 8
p55
p43
p18
PARP
116 kDa
cleaved form
Bid
22 kDa
RIP3K
56 kDa
GAPDH
37 kDa
Naive
Replated surviving cells day 2
B
RIPK3-Cherry expression level
(mean [pixel intensity])
centered on cells < 400 [a.u.]
Linear fit
corr = 0.966 (all cells)
of sister cell 2
of sister cell 1
Cell pair sharing same fate
Cell pair with diverging fate
Linear fit
corr = -0.049 (all cells)
of randomly chosen cell 2
of randomly chosen cell 1
C
Caspase-8 activation rate
(a.u.)
Linear fit
corr = 0.710
of sister cell 2
of sister cell 1
Linear fit
corr = 0.134
of randomly chosen Cell 2
of randomly chosen cell 1

Figure EV4. HT-29 TRAIL-tolerant persisters exhibit molecular features that can confer sensitivity to necroptosis.

Cell division does not participate to the diverging drug-sensitivity cell states. (**A**) Protein expressions of Caspase-8, PARP, cleaved PARP, Bid, RIPK3 and GAPDH in control conditions (−) and after TRAIL (25 ng/ml 1 and 4 h), in treatment-naïve HT-29 cells and in drug-tolerant persisters ("Replated surviving cells day 2", see (**B**) Fig. 4), measured by Western blot analysis (one representative experiment shown). (**B**) Comparison of RIPK3-Cherry expression levels and Caspase-8 activation rates (**C**) in pairs of recently divided sister cells. Clonal HeLa cells were treated with 10 ng/mL of TRAIL (to allow some cell divisions and to trigger some C8 activation) and observed by live-cell microscopy during 24 h. 189 dividing cells were identified (over a total of 654 cells), with 14 cells having a different drug response phenotype (tolerant or sensitive) than their sisters. Linear correlations and regressions are shown for all cells. Caspase-8 activation rates were obtained as the maximum of the time derivative of the FRET ratio between 2 h and 2 h 15 after division. Randomly chosen cell 1 were selected among the dividing cells.

 © The Author(s)

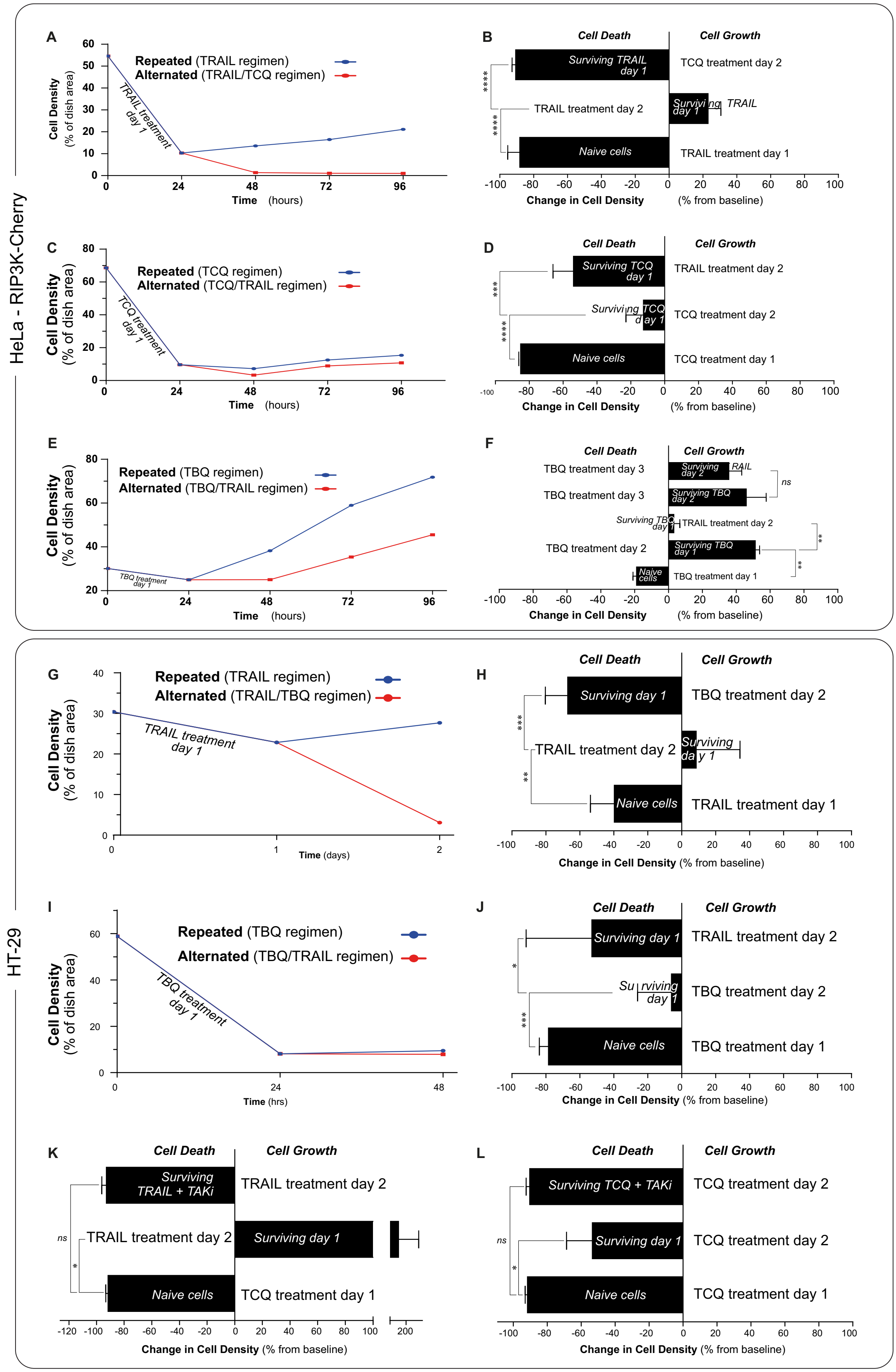
HeLa - RIP3K-Cherry
A
Repeated (TRAIL regimen)
Alternated (TRAIL/TCQ regimen)
Cell Density (% of dish area)
TRAIL treatment day 1
Time (hours)
B
Cell Death
Cell Growth
Surviving TRAIL day 1
TCQ treatment day 2
TRAIL treatment day 2
Naive cells
TRAIL treatment day 1
Change in Cell Density (% from baseline)
C
Repeated (TCQ regimen)
Alternated (TCQ/TRAIL regimen)
TCQ treatment day 1
D
Surviving TCQ day 1
TRAIL treatment day 2
TCQ treatment day 2
TCQ treatment day 1
E
Repeated (TBQ regimen)
Alternated (TBQ/TRAIL regimen)
F
TBQ treatment day 3
TBQ treatment day 2
TRAIL treatment day 2
TBQ treatment day 1
Surviving TBQ day 2
Surviving TBQ day 1
ns
HT-29
G
Repeated (TRAIL regimen)
Alternated (TRAIL/TBQ regimen)
Time (days)
H
Surviving day 1
TBQ treatment day 2
TRAIL treatment day 2
Naive cells
TRAIL treatment day 1
I
Repeated (TBQ regimen)
Alternated (TBQ/TRAIL regimen)
TBQ treatment day 1
Time (hrs)
J
TRAIL treatment day 2
TBQ treatment day 2
TBQ treatment day 1
K
Surviving TRAIL + TAKi
TRAIL treatment day 2
TCQ treatment day 1
L
Surviving TCQ + TAKi
TCQ treatment day 2
TCQ treatment day 1

Figure EV5. Alternating cell death modalities in a specific sequence represents a relevant strategy to limit the emergence of drug-tolerant persisters after TRAIL treatments in HT-29 cells.

(HeLa-RIPK3-Cherry box, Left panels: **A**, **C**, **E**) Cell density measured as fraction of dish area occupied by HeLa-RIPK3 cells after repeated TRAIL, TCQ or TBQ treatments (blue dots) or alternated treatment sequence TRAIL then TCQ or TBQ repeated once (red dots), over 96 h), corresponding experimental repeats are shown in the right panels (**B**, **D**, **F**) (same treatments). (HeLa-RIPK3-Cherry box, Right panels: **B**, **D**, **F**) Change in cell density of the population labeled inside each bar, caused by the treatment indicated on the side of each bar, measured as a difference in density before and after treatment. Each bar is an average change in cell density for three experimental repeats, data are presented as mean ± SEM, with ****p values of 1.0e-06 and 2.0e-08 downward correspondingly ((**B**), Student's t-test), with a ***p value of 9.6e-04 and a ****p value of 2.9e-06 ((**D**), Student's t-test), and with a ns p value of 0.3976 and **p values of 0.0047 and 0.0013 downward correspondingly ((**F**), Student's t-test). HT-29 box panels: (**G**) Cell density measured as fraction of dish area occupied by HT-29 cells after repeated TRAIL (blue dots) or alternated treatment sequence TRAIL then TBQ repeated once (TRAIL 20 ng/mL, then TBQ with TRAIL 10 ng/ml, BV6 200 nM, q-VD 10 µM, red dots), over 48 h. A representative clonogenicity experiment is shown at each time point), corresponding experimental repeats are shown in (**B**) (same treatments). (**H**) Change in Cell Density of the population labeled inside each bar, caused by the treatment indicated on the side of each bar, measured as a difference in density before and after treatment. Each bar is an average change in cell density for three experimental repeats, data were presented as mean ± SEM, a **p value of 0.0042 and a ***p value of 5.4e-05 (Student's t-test). (**I**) Cell density measured as fraction of dish area occupied by HT-29 cells after repeated TBQ treatments (blue dots) or alternated treatment sequence TBQ then TRAIL repeated once (TRAIL 20 ng/mL, then TBQ with TRAIL 10 ng/ml, BV6 200 nM, q-VD 10 uM, red dots), over 48 h. A representative clonogenicity experiment is shown at each time point), corresponding experimental repeats are shown in (**D**) (same treatments). (**J**, **K**, **L**) Change in cell density of the population labeled inside each bar, caused by the treatment indicated on the side of each bar, measured as a difference in density before and after treatment, with or without TAK1 inhibitor, TAKi, 2.5 µM). Each bar is an average change in cell density for three experimental repeats, data were presented as mean ± SEM, with a *p value of 0.0258 and a ***p value of 3.1e-05 ((**J**), Student's t-test), with a ns p value of 0.5452 and a *p value of 0.0273 ((**K**), Student's t-test), and with a ns p value of 0.3317 and a *p value of 0.0119 ((**L**), Student's t-test).

 © The Author(s)

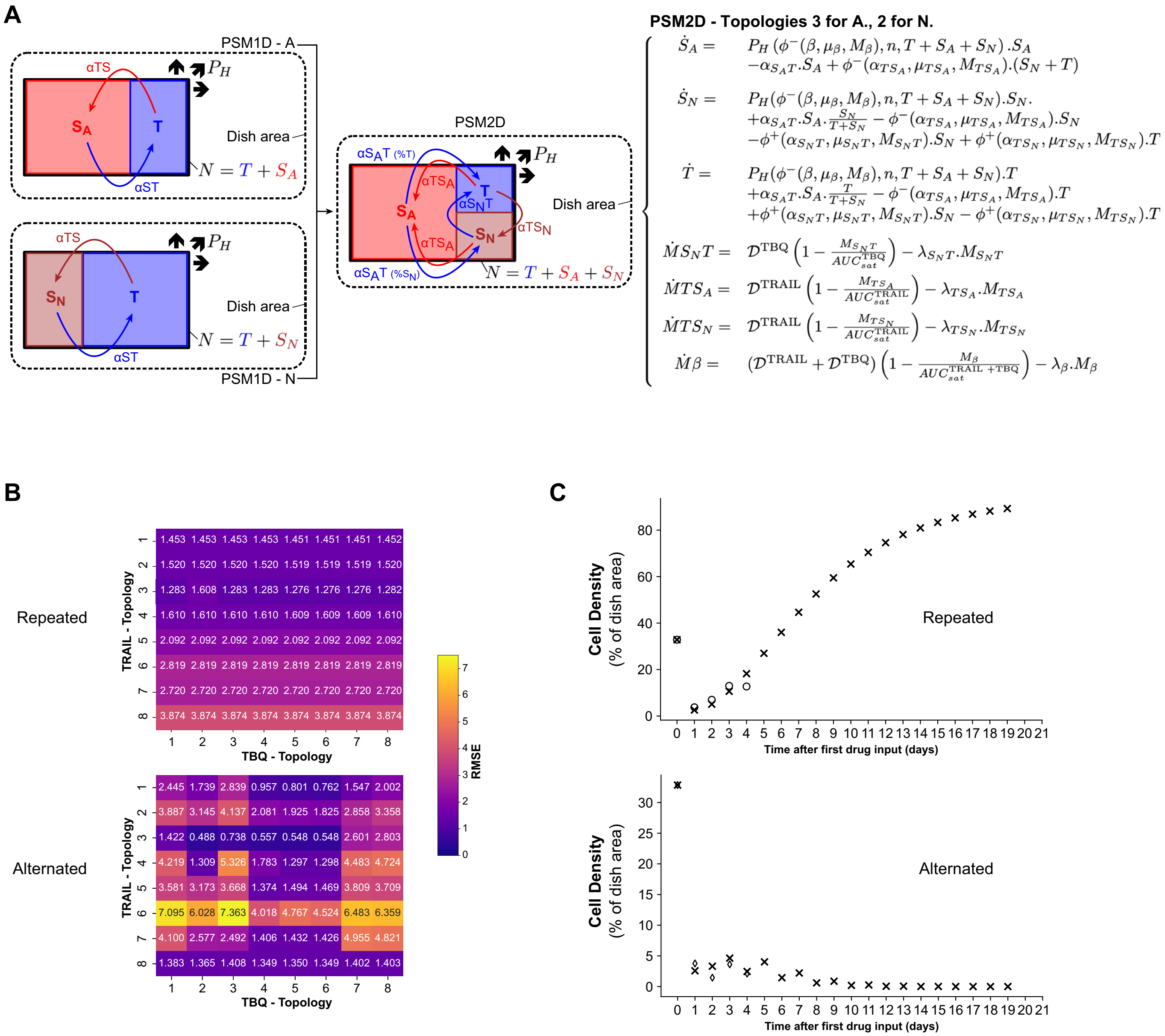

Figure EV6. Two-drug phenotypic switch model (PSM2D) construction and simulations show that an alternated regimen improves long-term cancer treatment efficacy.

(**A**) Phenotypic switch model with one drug (PSM1D) for pro-apoptotic (**A**) and pro-necroptotic (N) treatments are coupled to create PSM2D. The two diagrams on the left show switching rates and initial conditions used during the simulation of PSM1D. PSM1D T and S_m initial conditions are set to experimental values as such: the initial proportion of tolerant cells $T(0)$ is set to the % of area occupied in the dish after the first drug input, while $S_m(0)$ is equal to the difference between the % of area occupied before the first drug input and 24 h after. (Experimentally, the dying cells are the manifestation of the sensitive cell state and therefore represent the sensitive cell population in the model.) Note that $S_A(0)$ is therefore much higher than $S_N(0)$. Figure 5B suggests that a tolerance to TRAIL increases the sensitivity to TBQ. To create PSM2D, we encode this ability by using PSM1D for pro-apoptotic drug (TRAIL) as a starting point and splitting the tolerant compartment in the model into two sub-ones: tolerant T to both drugs and S_N. From S_A perspective, T and S_N are seen as tolerant (right diagram). We therefore used the switching and sensitive rates values obtained after calibration of each PSM1D, along with the same reset speeds and split α_{SAT} into $\alpha_{SASN} = \alpha_{SAT}$. $S_N/(S_N + T)$ (denoted by $\alpha_{SAT}(\%S_N)$ and $\alpha_{SAT} = \alpha_{SAT}$. $T/(S_N + T)$ (denoted by $\alpha_{SAT}(\%T)$ in (**C**). α_{SNT}, α_{TSA} and α_{TSN} conserve their value from the calibration of PSM1D. To simplify the creation of the model, we made two assumptions. First, the effects of the two drugs on proliferation is simply additive, whereas their effects on the switching rate from S_N to T is multiplicative/composed. Finally, we impose the activation of α_{TSN} by TRAIL. Equations correspond to the PSM2D model simulated in Fig. 6 when coupling PSM1D -Topology 3 for pro-apoptotic drug and PSM1D—Topology 2 for pro-necroptotic drugs. (**B**) RMSE of each PSM2D topology were obtained by comparing the N solution ("Total" population, Fig. 5F) with experimental data for each drug regimen (Fig. 5A). (**C**) PSM2D long-term simulations. Topologies combination with the lowest RMSE for alternated regimen is shown for both simulations.

© The Author(s)
